# Estimated effect of increased diagnosis, treatment, and control of diabetes and its associated cardiovascular risk factors among low-income and middle-income countries: a microsimulation model

**DOI:** 10.1016/S2214-109X(21)00340-5

**Published:** 2021-09-22

**Authors:** Sanjay Basu, David Flood, Pascal Geldsetzer, Michaela Theilmann, Maja E Marcus, Cara Ebert, Mary Mayige, Roy Wong-McClure, Farshad Farzadfar, Sahar Saeedi Moghaddam, Kokou Agoudavi, Bolormaa Norov, Corine Houehanou, Glennis Andall-Brereton, Mongal Gurung, Garry Brian, Pascal Bovet, Joao Martins, Rifat Atun, Till Bärnighausen, Sebastian Vollmer, Jen Manne-Goehler, Justine Davies

**Affiliations:** a Center for Primary Care, Harvard Medical School, Boston, MA, USA; b Ariadne Labs, Harvard T H Chan School of Public Health, Brigham and Women's Hospital, Boston, MA, USA; c Department of Global Health and Population, Harvard T H Chan School of Public Health, Brigham and Women's Hospital, Boston, MA, USA; d School of Public Health, Imperial College, London, UK; e Research and Population Health, Collective Health, San Francisco, CA, USA; f Institute of Health Policy, Management and Evaluation, University of Toronto, Toronto, ON, Canada; g Division of Hospital Medicine, Department of Internal Medicine, National Clinician Scholars Program, University of Michigan, Ann Arbor, MI, USA; h Center for Indigenous Health Research, Wuqu' Kawoq, Tecpán, Guatemala; i Research Center for the Prevention of Chronic Diseases, Institute of Nutrition of Central America and Panama, Guatemala City, Guatemala; j Division of Primary Care and Population Health, Department of Medicine, Stanford University, Stanford, CA, USA; k Heidelberg Institute of Global Health, Heidelberg University and University Hospital, Heidelberg, Germany; l Department of Economics and Center for Modern Indian Studies, University of Goettingen, Goettingen, Germany; m Rheinisch-Westfälisches Institut–Leibniz Institute for Economic Research, Essen, Germany; n Epidemiology Department, National Institute for Medical Research, Dar es Salaam, Tanzania; o Office of Epidemiology and Surveillance, Costa Rican Social Security Fund, San José, Costa Rica; p Non-Communicable Diseases Research Center, Endocrinology and Metabolism Population Sciences Institute, Tehran University of Medical Sciences, Tehran, Iran; q Endocrinology and Metabolism Research Center, Endocrinology and Metabolism Clinical Sciences Institute, Tehran University of Medical Sciences, Tehran, Iran; r Tehran University of Medical Sciences, Tehran, Iran; s Togo Ministry of Health, Lome, Togo; t National Center for Public Health, Ulaanbaatar, Mongolia; u National Training School for Senior Technicians in Public Health and Epidemiological Surveillance (ENATSE), University of Parakou, Parakou, Benin; v Non-Communicable Diseases, Caribbean Public Health Agency, Port of Spain, Trinidad and Tobago; w Health Research and Epidemiology Unit, Ministry of Health, Thimphu, Bhutan; x The Fred Hollows Foundation, Sydney, NSW, Australia; y Ministry of Health, Victoria, Seychelles; z Rector of the Univesidade Nacional Timor Lorosae, Dili, Timor-Leste; aa Africa Health Research Institute, Somkhele, South Africa; ab Division of Infectious Diseases, Brigham and Women's Hospital, Boston, MA, USA; ac Medical Practice Evaluation Center, Massachusetts General Hospital, Harvard Medical School, Boston, MA, USA; ad Institute for Applied Health Research, University of Birmingham, Birmingham, UK; ae Centre for Global Surgery, Department of Global Health, Stellenbosch University, Cape Town, South Africa; af Medical Research Council–Wits University Rural Public Health and Health Transitions Research Unit, Faculty of Health Sciences, School of Public Health, University of the Witwatersrand, Johannesburg, South Africa

## Abstract

**Background:**

Given the increasing prevalence of diabetes in low-income and middle-income countries (LMICs), we aimed to estimate the health and cost implications of achieving different targets for diagnosis, treatment, and control of diabetes and its associated cardiovascular risk factors among LMICs.

**Methods:**

We constructed a microsimulation model to estimate disability-adjusted life-years (DALYs) lost and health-care costs of diagnosis, treatment, and control of blood pressure, dyslipidaemia, and glycaemia among people with diabetes in LMICs. We used individual participant data—specifically from the subset of people who were defined as having any type of diabetes by WHO standards—from nationally representative, cross-sectional surveys (2006–18) spanning 15 world regions to estimate the baseline 10-year risk of atherosclerotic cardiovascular disease (defined as fatal and non-fatal myocardial infarction and stroke), heart failure (ejection fraction of <40%, with New York Heart Association class III or IV functional limitations), end-stage renal disease (defined as an estimated glomerular filtration rate <15 mL/min per 1·73 m^2^ or needing dialysis or transplant), retinopathy with severe vision loss (<20/200 visual acuity as measured by the Snellen chart), and neuropathy with pressure sensation loss (assessed by the Semmes-Weinstein 5·07/10 g monofilament exam). We then used data from meta-analyses of randomised controlled trials to estimate the reduction in risk and the WHO OneHealth tool to estimate costs in reaching either 60% or 80% of diagnosis, treatment initiation, and control targets for blood pressure, dyslipidaemia, and glycaemia recommended by WHO guidelines. Costs were updated to 2020 International Dollars, and both costs and DALYs were computed over a 10-year policy planning time horizon at a 3% annual discount rate.

**Findings:**

We obtained data from 23 678 people with diabetes from 67 countries. The median estimated 10-year risk was 10·0% (IQR 4·0–18·0) for cardiovascular events, 7·8% (5·1–11·8) for neuropathy with pressure sensation loss, 7·2% (5·6–9·4) for end-stage renal disease, 6·0% (4·2–8·6) for retinopathy with severe vision loss, and 2·6% (1·2–5·3) for congestive heart failure. A target of 80% diagnosis, 80% treatment, and 80% control would be expected to reduce DALYs lost from diabetes complications from a median population-weighted loss to 1097 DALYs per 1000 population over 10 years (IQR 1051–1155), relative to a baseline of 1161 DALYs, primarily from reduced cardiovascular events (down from a median of 143 to 117 DALYs per 1000 population) due to blood pressure and statin treatment, with comparatively little effect from glycaemic control. The target of 80% diagnosis, 80% treatment, and 80% control would be expected to produce an overall incremental cost-effectiveness ratio of US$1362 per DALY averted (IQR 1304–1409), with the majority of decreased costs from reduced cardiovascular event management, counterbalanced by increased costs for blood pressure and statin treatment, producing an overall incremental cost-effectiveness ratio of $1362 per DALY averted (IQR 1304–1409).

**Interpretation:**

Reducing complications from diabetes in LMICs is likely to require a focus on scaling up blood pressure and statin medication treatment initiation and blood pressure medication titration rather than focusing on increasing screening to increase diabetes diagnosis, or a glycaemic treatment and control among people with diabetes.

**Funding:**

None.

## Introduction

Diabetes is a leading cause of death and disability worldwide, with about 80% of 463 million adults with diabetes residing in low-income and middle-income countries (LMICs).^1^ Diabetes and its associated macrovascular and microvascular complications are a recognised challenge to achieving the Sustainable Development Goal 3.4: “By 2030, reduce by one third premature mortality from non-communicable diseases through prevention and treatment and promote mental health and well-being.”^2^ Prevalence and management has, therefore, been the subject of the 2021 WHO Global Diabetes Compact to support countries with diabetes management,^3^ including dedicated resources for diabetes care.^4^ The diabetes care cascade across 28 LMICs was recently described in a cross-sectional study of nationally representative surveys, which estimated that the total unmet need for diabetes care (defined as the sum of those not screened, screened but not diagnosed, diagnosed but not treated, and treated but not controlled) was 77% of those with diabetes.^5^ A more recent study has shown that fewer than 5% of people with diabetes living in 55 LMICs receive treatment of cardiovascular disease risk factors (eg, diabetes, hypertension, and medication with a statin) as recommended by WHO guidelines.^6^ Individuals in LMICs often have catastrophic spending for diabetes care and do not have appropriate medications to treat diabetes, even when they have health-care insurance.^7^

**Research in context**

**Evidence before this study**

We searched Web of Science and PubMed for primary research literature on June 14, 2021, using the terms “diabetes” and “cost-effectiveness”. We limited our search to studies done since 2010 with no language restrictions. We found 3785 studies, of which 3710 (98%) focused on cost-effectiveness of individual care components for diabetes (ie, specific medication choices) or were done in high-income countries. A systematic review done in 2020 found strong evidence for regular screening to detect diabetes and for blood pressure control. Among studies related to low-income and middle-income countries (LMICs), many described low levels of diagnosis, treatment, and control. One study reported that a risk-based approach to treatment (treating glucose, blood pressure, and lipids until calculated risk reduced below a threshold) was more cost-effective than treating to specific laboratory or clinical measures (eg, until reaching a certain blood glucose concentration), and another study using a similar approach assessed the cost-effectiveness of meeting management recommendations for cardiovascular disease risk factors among people with diabetes in South Africa. Several studies assessed the cost-effectiveness of implementing the Diabetes Prevention Program in LMICs, but we did not identify a previous primary research study that assessed the cost-effectiveness of achieving targets for diabetes screening, treatment, or control in LMICs. Additionally, we did not find organised, population-representative data for risk factors for renal, ophthalmic, and neuropathic complications of diabetes in LMICs.

**Added value of this study**

In this study, we evaluated how the health consequences and management costs of diabetes and its complications would be expected to change if LMICs achieved different targets for diabetes diagnosis, treatment, and control. We collected individual participant level data from nationally representative population-based cross-sectional surveys done in LMICs and used risk equations to provide estimates of cardiovascular, renal, ophthalmic, and neuropathic complications of diabetes in these countries. The study addressed the important unanswered question of which targets for diabetes treatment would be most beneficial at a population level for overall reduction of disability-adjusted life-years (DALYs) attributed to diabetes complications. We observed that the major incremental benefits of increased diagnosis, treatment, or control were to reduce cardiovascular events, despite the large baseline burden of end-stage renal disease. The greatest reductions in cardiovascular events were achieved through increased treatment with blood pressure and statin medicines, and increased titration of blood pressure medicines to achieve blood pressure targets.

**Implications of all the available evidence**

When considered altogether, the available evidence points to increasing the treatment and control of blood pressure and increasing treatment with statin medications as among the most important strategies for reducing DALYs attributable to diabetes complications in LMICs.

As WHO and other entities address improvement to diabetes care, lessons from other disease control efforts might be pertinent. For example, in 2014 the UN set the 95-95-95 HIV management targets for countries—ie, 95% of people with HIV would be diagnosed, 95% of those diagnosed would be treated, and 95% of those treated would achieve viral suppression. Despite the numerous challenges acknowledged in achieving these targets, they are now credited with driving cross-country efforts to improve health services for patients with HIV.^8^, ^9^, ^10^ Similar targets have been adopted for other conditions.^11^ For type 1 diabetes, which is rapidly fatal without simple treatment, many argue that 100% of patients should be diagnosed and treated.^4^ However, whether or not targets should be put forward for patients with other forms of diabetes—and if so, what those should be—remains unclear. Estimates of the potential benefits and costs of different scale-up activities are needed to help prioritise strategies for health systems.

In this study, we therefore aimed to estimate the costs and benefits of achieving targets for diagnosis, treatment, and control of diabetes and its associated cardiovascular risk factors of hypertension and dyslipidaemia among LMICs.

## Methods

### Model overview

**Figure 1:**
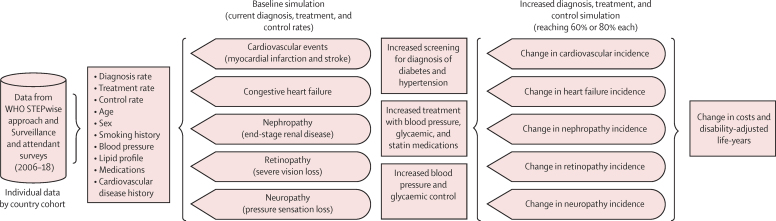
Model diagram Individual level data from survey respondents with diabetes mellitus in the WHO STEPwise approach to Surveillance and attendant surveys (2006–18)^12^ were used to estimate the baseline risk of macrovascular and microvascular complications of diabetes. Data from randomised controlled trials were then used to estimate the effect of increased blood pressure, glycaemia, and statin treatment, and increased blood pressure and glucose control, with or without new screening to increase the overall rates of diagnosis of diabetes and hypertension.

We constructed a microsimulation to estimate the disability-adjusted life-years (DALYs) lost to the macrovascular and microvascular complications of diabetes, and health-care costs including prevention and treatment of these complications, among people with diabetes in LMICs. We estimated the effect of the increased diagnosis, treatment, and control measures for glycaemia, blood pressure, and dyslipidaemia following WHO guidelines. A microsimulation simulates individual people, their demographics, health-related risk factors, and outcomes, and then aggregates the individual events they experience over the life-course to estimate health outcomes and costs for the overall population (figure 1; appendix pp 54–55).^12^, ^13^

### Target populations and data source

We simulated each country's population with diabetes by sampling from the individual patient data in the WHO STEPwise approach to Surveillance (STEPS) and other similar attendant surveys (2006–18),^14^ specifically from the subset of people who were defined as having any type of diabetes by WHO standards (fasting blood glucose >7 mmol/L, non-fasting glucose >11·1 mmol/L, glycated haemoglobin A_1c_ [HbA_1c_] ≥6·5% [48 mmol/mol], or taking a glycaemic control medicine including insulin) across 67 countries spanning 15 world regions.^15^ Details about the surveys included are shown in the appendix (pp 3–53), and included sampling weights to adjust samples to be representative to the World Population Prospects estimates of the overall country population by age and sex.^16^ To be included in the analysis, surveys needed to have collected data allowing calculation of individual presence of diabetes or hypertension, and whether these conditions had been previously diagnosed, or were treated or controlled, and whether the individual was on a statin medication. We describe the survey data both at the regional level and at the individual country level. Missing data were imputed using multiple imputation with chained equations plus a classification and regression tree algorithm to account for the complex covariation among data elements.^17^

### Outcomes and simulated scenarios

We calculated each individual's baseline 10-year risk of cardiovascular disease (defined as fatal and non-fatal myocardial infarction and stroke); heart failure with reduced ejection fraction (ejection fraction of <40%, with New York Heart Association class III or IV functional limitations); end-stage renal disease (defined as an estimated glomerular filtration rate <15 mL/min per 1·73 m^2^ or needing dialysis or transplant); retinopathy with severe vision loss (<20/200 visual acuity as measured by the Snellen chart); neuropathy with pressure sensation loss (assessed by the Semmes-Weinstein 5·07/10 g monofilament exam); or DALYs (computed as the sum of years of life with disability and years of life lost because of mortality from each outcome). Baseline cardiovascular disease risk was estimated by the 2019 WHO cardiovascular disease risk equations by region (using laboratory-based equations where lipid data were available, and clinically based equations otherwise),^18^ and the risk of other outcomes were estimated using the Risk Equations for Complications of type 2 Diabetes (appendix pp 64–65).^19^, ^20^ The disability weights used in the DALY calculations were obtained from a multi-country survey assessment (appendix p 66).^21^ In the baseline simulation, we estimated the risk of each outcome given the current levels of diagnosis and treatment observed in the survey data. We simulated combinations of increased diagnosis, treatment, and control, to 60% of each or 80% of each activity, individually and in combination (eg, to achieve 60% treatment and 60% control, or 80% screening, 60% treatment, and 60% control). We note that each element of the cascade affected all downstream elements, such that increased screening would increase the absolute number of people being treated and controlled (even if the percentage treated or controlled remained unchanged), and increased treatment would increase the absolute number of people controlled (even if the percentage controlled remained unchanged). During the simulation, we computed the probability of cause-specific mortality and all-cause mortality based on country-specific data from the Institute for Health Metrics and Evaluation,^22^ and computed the overall DALYs lost by summing the disutility-weighted years of life lived in disability and years of life lost.

For diagnosis, we randomly sampled among those undiagnosed to bring the proportion of people with diabetes who were diagnosed up to 60% or up to 80% within each country's population, and the portion of those with diabetes and hypertension who were diagnosed with hypertension up to 60% or up to 80%, leaving unaltered those countries with a baseline level above these proportions (appendix pp 56–63). For increased treatment, we simulated the initiation of the first stage of treatment for up to 60% or up to 80% of those diagnosed; treatment initiation followed the 2020 WHO Package of Essential Non-communicable Disease (PEN) interventions,^23^ which included enalapril 20 mg once per day for a systolic blood pressure of 130 mm Hg or higher or a diastolic blood pressure of 80 mm Hg or higher, simvastatin 20 mg once per day for those aged 40 years or older or having an estimated 10-year cardiovascular risk of more than 20%, and metformin 500 mg once per day for those with fasting plasma glucose of 7 mmol/L (126 mg/dL) or more and less than 18 mmol/L (325 mg/dL) or a random plasma glucose of 11·1 mmol/L (200 mg/dL) or more and <18 mmol/L (325 mg/dL) or gliclazide 80 mg twice per day for those with a fasting or random plasma glucose of 18 mmol/L (325 mg/dL) or more.^23^ For increased control, we simulated the continuation of the medication titration algorithms of the PEN guidelines for up to 60% or up to 80% of those treated, to achieve the WHO targets for blood pressure (systolic blood pressure <130 mm Hg or diastolic <80 mm Hg) and glycaemic control (HbA_1c_ ≤7% [53·0 mmol/mol] or fasting plasma glucose <7 mmol/L [126 mg/dL]). We did not simulate titration of statin treatment to a specific lipid biomarker concentration, given current evidence favouring risk-based treatment rather than target-based treatment.^24^, ^25^ We estimated the effect of reduced blood pressure, reduced glycaemia, or initiation of a statin on the risk reduction for each outcome for each individual on the basis of meta-analyses of randomised controlled trials (appendix [pp 2–3]).

### Cost estimates

Cost estimates for clinical management of conditions were derived using the WHO OneHealth Tool, a standardised spreadsheet-based tool estimating the costs for the clinical visits at primary, secondary, or tertiary facilities, and costs of common diagnostic tests (eg, laboratory tests or x-rays; appendix pp 67–71). Pharmaceutical costs were based on international drug prices from the UN, Management Sciences for Health, and International Dispensary Association. Costs of screening to make new diagnoses were based on a previous estimate of costs for random plasma glucose testing via community-based health workers or primary care clinics,^26^ adjusted for local labour and material costs in each country to include both initial screening and confirmatory testing with fasting plasma glucose or HbA_1c_, or both. Costs were updated to 2020 International Dollars. Costs and DALYs were computed over a 10-year policy planning time horizon at a 3% annual discount rate.^27^

### Role of the funding source

There was no funding source for this study.

## Results

We obtained data from 23 678 people with diabetes from surveys across 67 countries spanning 15 world regions (tables 1, 2). The median age was 53·0 years (IQR 42·0–61·0). Of the 23 678 people with diabetes, 14 164 (59·8%) were female, 11 967 (50·5%) reported being previously diagnosed with diabetes before the survey, and 9288 (39·2%) reported being previously diagnosed with hypertension. The overall sample population had a median systolic blood pressure of 134·0 mm Hg (IQR 121·0–150·7) and a median HbA_1c_ of 7·4% (IQR 6·6–9·2; 57·4 mmol/mol [IQR 48·6–77·1]).

**Figure 2:**
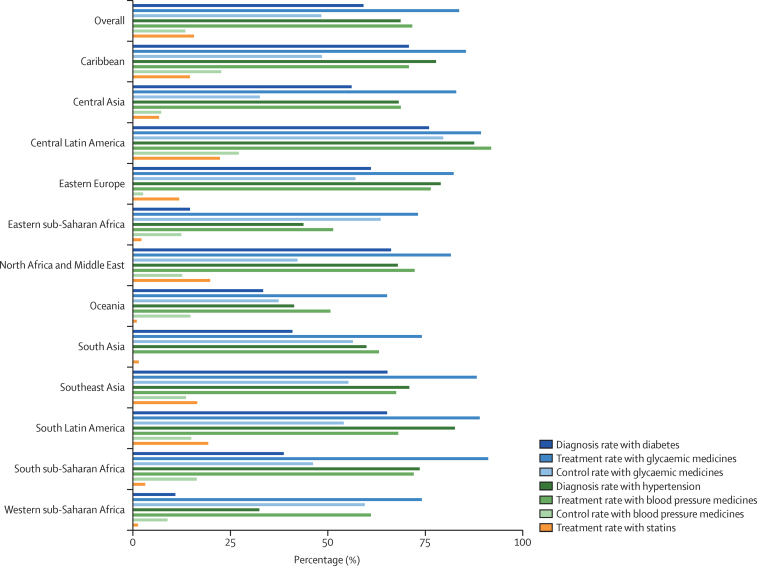
Treatment cascade for people with diabetes, from the WHO STEPwise approach to Surveillance and attendant surveys (2006–18)^15^ Diagnosis rate with diabetes mellitus is defined as the proportion of those reporting a previous diagnosis of diabetes, among those with clinical diabetes (defined as fasting blood glucose >7 mmol/L, or non-fasting blood glucose >11·1 mmol/L, HbA_1c_ ≥6·5% [48 mmol/mol] or taking a glycaemic control medicine including insulin). Diagnosis rate with hypertension is defined as the proportion of those reporting a previous diagnosis of hypertension, among those with clinical hypertension (defined as previous diagnosis, a systolic blood pressure ≥140 mm Hg or a diastolic blood pressure ≥90 mm Hg at the time of the survey, or taking blood pressure medicines). Treatment rate with glycaemic medicines is defined as those treated among those diagnosed with diabetes. Treatment rate with blood pressure medicines is defined as those treated among those diagnosed with hypertension. Treatment rate with statins is defined as those treated among individuals who are 40 years or older or having an estimated 10-year pre-treatment cardiovascular risk greater than 20%. The control rate with glycaemic medicines is the proportion of people diagnosed and treated with glycaemic medicines who achieved glycaemic control (HbA_1c_ ≤7% [53 mmol/mol] or a fasting plasma glucose <7 mmol/L [126 mg/dL]). The control rate with blood pressure medicines is the proportion of people diagnosed and treated for hypertension who achieved blood pressure control (systolic blood pressure <130 mm Hg and diastolic blood pressure <80 mm Hg). HbA_1c_=glycated haemoglobin A_1c_.

Figure 2 summarises the cascade of diagnosis, treatment, and control of diabetes, hypertension, and dyslipidaemia. We observed wide variations across diagnosis, treatment, and control indicators at the county level and at the regional level (table 1; appendix pp 56–63).

**Table 1:** Descriptive statistics on the survey respondents included in the study, WHO STEPwise approach to Surveillance, and attendant surveys (2006–18)^15^

	**Oceania**	**Andean Latin America**	**Central Latin America**	**Southern Latin America**	**Caribbean**	**Central Europe**	**Eastern Europe**
**Demographic**s							
Total	3127	341	3075	538	493	256	586
Females	1706 (54·6%)	194 (56·9%)	1986 (64·6%)	317 (58·9%)	358 (72·6%)	107 (41·8%)	380 (64·8%)
Males	1421 (45·4%)	147 (43·1%)	1089 (35·4%)	221 (41·1%)	135 (27·4%)	149 (58·2%)	206 (35·2%)
Age (years)	48·0 (38·0–56·0)	52·0 (43·0–60·0)	57·0 (47·0–67·0)	61·0 (51·3–70·0)	55·0 (46·0–65·0)	63·0 (53·0–70·0)	58·0 (51·0–64·0)
**Clinical measurements**							
BMI (kg/m^2^)	29·34 (25·4–34·2)	28·9 (25·3–32·5)	29·4 (26·2–33·3)	29·8 (26·3–34·1)	29·3 (25·8–33·5)	30·8 (28·0–34·6)	31·2 (27·0–35·5)
Currently smokes tobacco	785 (25·1%)	44 (12·9%)	314 (10·2%)	117 (21·7%)	23 (4·7%)	47 (18·4%)	85 (14·5%)
History of heart attack	282 (9·0%)	38 (11·1%)	218 (7·1%)	68 (12·6%)	58 (11·8%)	13 (5·1%)	147 (25·1%)
Systolic blood pressure (mm Hg)	131·0 (119·0–146·3)	125·3 (116·0–137·0)	132·0 (119·0–151·0)	137·5 (125·5–154·5)	134·0 (119·0–150·0)	138·0 (125·4–151·0)	150·7 (134·3–170·3)
Diastolic blood pressure (mm Hg)	81·5 (73·0–89·7)	77·7 (71·3–84·3)	79·0 (70·0–88·0)	78·0 (70·5–87·5)	80·0 (71·5–87·0)	80·5 (74·5–87·5)	91·3 (82·1–99·0)
Fasting blood glucose (mmol/L)	8·6 (7·5–11·8)	8·1 (7·1–11·5)	8·1 (6·6–11·9)	7·9 (7·2–10·9)	8·6 (7·1–11·9)	7·8 (7·1–9·3)	7·7 (7·0–9·8)
HbA_1c_ (mmol/mol)	57·4 (48·6–74·9)	53·2 (48·6–72·7)	61·8 (48·6–82·5)	57·4 (48·6–75·8)	58·5 (48·6–78·1)	48·1 (38·8–59·6)	58·5 (48·6–76·0)
HbA_1c_ (%)	7·4 (6·6–9·0)	7·0 (6·6–8·8)	7·8 (6·6–9·7)	7·4 (6·6–9·1)	7·5 (6·6–9·3)	6·6 (5·7–7·6)	7·5 (6·6–9·1)
Total cholesterol (mmol/L)	4·6 (3·9–5·4)	4·9 (4·1–5·8)	4·9 (4·2–5·6)	5·0 (4·2–5·8)	4·8 (4·0–5·7)	5·0 (4·2–5·8)	5·0 (4·3–5·8)
Total cholesterol (mg/dL)	177·5 (151·6–206·9)	190·0 (157·0–226·0)	189·0 (163·0–215·0)	193·1 (164·0–222·8)	186·0 (154·0–220·0)	193·5 (161·8–225·1)	193·34 (166·0–224·3)
HDL cholesterol (mmol/L)	1·0 (0·8–1·3)	1·0 (0·8–1·3)	1·0 (0·9–1·2)	1·1 (0·9–1·3)	1·2 (0·9–1·5)	1·2 (1·0–1·4)	1·3 (1·0–1·6)
HDL cholesterol (mg/dL)	39·1 (29·8–50·7)	39·1 (32·0–49·9)	40·0 (34·0–47·0)	41·0 (35·0–50·0)	46·4 (36·4–58·8)	47·0 (38·0–55·3)	48·3 (39·1–61·4)
LDL cholesterol (mmol/L)	2·6 (2·1–3·3)	2·9 (2·1–3·7)	2·8 (2·2–3·4)	2·9 (2·3–3·6)	2·8 (2·1–3·6)	3·0 (2·3–3·7)	2·8 (2·2–3·5)
LDL cholesterol (mg/dL)	101·2 (80·0–127·3)	111·6 (81·6–141·6)	107·4 (85·2–130·5)	113·8 (90·7–140·1)	108·5 (82·2–137·2)	116·1 (88·0–142·5)	109·5 (84·9–135·0)
**Diabetes cascade**							
Clinical diabetes	3127 (100%)	341 (100%)	3075 (100%)	538 (100%)	493 (100%)	256 (100%)	586 (100%)
Diabetes diagnosis rate	1012 (32·4%)	161 (47·2%)	2108 (68·6%)	331 (61·5%)	349 (70·8%)	198 (77·3%)	356 (60·8%)
On treatment for diabetes	501 (16·0%)	144 (42·2%)	1741 (56·6%)	244 (45·4%)	299 (60·6%)	37 (14·5%)	296 (50·5%)
Diabetes treatment rate*	49·5%	89·4%	82·6%	73·7%	85·7%	18·7%	83·1%
Current insulin use	402 (12·9%)	37 (10·9%)	1071 (34·8%)	46 (8·6%)	49 (9·9%)	12 (4·7%)	94 (16·0%)
HbA_1c_ ≤7% or fasting blood glucose <7 mmol/L	1284 (41·1%)	198 (58·1%)	1612 (52·4%)	272 (50·6%)	241 (48·9%)	162 (63·3%)	293 (50·0%)
Diabetes control rate†	31·5%	58·3%	55·8%	55·3%	53·2%	97·3%	58·8%
**Hypertension cascade**							
Hypertension	1364 (43·6%)	126 (37·0%)	1870 (60·8%)	335 (62·3%)	298 (60·4%)	162 (63·3%)	471 (80·4%)
Previous diagnosis of hypertension‡	675 (49·5%)	121 (96·0%)	1440 (77·0%)	316 (94·3%)	262 (87·9%)	162 (100%)	389 (82·6%)
Medication for raised blood pressure	332 (10·6%)	71 (20·8%)	1212 (39·4%)	166 (30·9%)	186 (37·7%)	0	298 (50·9%)
Hypertension treatment rate*	49·2%	58·7%	84·2%	52·5%	71·0%	0%	76·6%
SBP <130 mm Hg and DBP <80 mm Hg	1065 (34·1%)	160 (46·9%)	1022 (33·2%)	152 (28·3%)	164 (33·3%)	67 (26·2%)	54 (9·2%)
Hypertension control rate†	17·2%	32·4%	26·2%	21·1%	23·7%	0%	2·7%
**Statin cascade**							
Statin treatment indicated as aged >40 years or 10-year cardiovascular event risk >20%	2182 (69·8%)	278 (81·5%)	2687 (87·4%)	496 (92·2%)	423 (85·8%)	228 (89·1%)	520 (88·7%)
Current statin use	101 (3·2%)	24 (7·0%)	359 (11·7%)	64 (11·9%)	34 (6·9%)	13 (5·1%)	63 (10·8%)
Statin treatment rate*	4·6%	8·6%	13·4%	12·9%	8·0%	5·7%	12·1%

Data are n, n (%), n/N (%), or median (IQR). We included the subset of people with diabetes mellitus (defined as fasting blood glucose >7 mmol/L, or non-fasting blood glucose >11·1 mmol/L, HbA_1c_ ≥6·5% [48 mmol/mol] or taking a glycaemic control medicine including insulin) across 67 countries spanning 15 world regions. For data related to blood pressure, glycaemia, and statin medicine cascades, participants had to fulfil criteria for the preceding step to be included in the denominator for the next step (eg, a person had to be diagnosed to be in the denominator of the percentage treated, or had to be treated to be in the denominator of the percentage controlled). Hypertension was defined by self-reported diagnosis of hypertension (SBP ≥140 mm Hg or DBP ≥90 mm Hg) on hypertensive treatment. BMI=body-mass index. DBP=diastolic blood pressure. HbA_1c_=glycated haemoglobin A_1c_. SBP=systolic blood pressure.

* For diabetes and hypertension, calculated as those diagnosed and treated, divided by those diagnosed; for statins, calculated as those indicated for treatment and treated, divided by those indicated for treatment.

† Calculated as those who have their condition controlled divided by the sum of those diagnosed and treated.

‡ Percentage of those with hypertension.

At the current levels of diagnosis, treatment, and control observed in individuals with diabetes, we estimated the highest future risks were for cardiovascular events and neuropathy, followed by end-stage renal disease, severe retinopathy, and heart failure (table 3). Risks are expressed in terms of the median 10-year risk, which can be interpreted as the proportion of the population who would be expected to newly experience the outcome within 10 years. The median estimated 10-year risk was 10·0% (IQR 4·0–18·0) for cardiovascular events, 7·8% (5·1–11·8) for neuropathy with pressure sensation loss, 7·2% (5·6–9·4) for end-stage renal disease, 6·0% (4·2–8·6) for retinopathy with severe vision loss, and 2·6% (1·2–5·3) for congestive heart failure.

**Table 3:** Modelled effect of increased hypertension and diabetes diagnosis; increased blood pressure, glycaemia, and statin treatment; and increased blood pressure and glucose control on the risk of diabetes complications

		**Cardiovascular events**		**Congestive heart failure**		**Neuropathy**		**End-stage renal disease**		**Retinopathy**	
		Risk	Decrease in risk from baseline	Risk	Decrease in risk from baseline	Risk	Decrease in risk from baseline	Risk	Decrease in risk from baseline	Risk	Decrease in risk from baseline
Baseline		10·0% (4·0–18·0)	NA	2·6% (1·2–5·3)	NA	7·8% (5·1–11·8)	NA	7·2% (5·6–9·4)	NA	6·0% (4·2–8·6)	NA
Diagnosis											
	Increase diagnosis of diabetes	NA	NA	NA	NA	7·2% (4·7–10·9)	0·6%	6·7% (5·0–8·9)	0·5%	5·0% (3·5–7·1)	1·0%
	Increase diagnosis of hypertension	9·0% (4·0–15·0)	1·0%	2·4% (1·2–4·7)	0·2%	NA	NA	6·7% (5·0–9·0)	0·5%	5·0% (3·5–7·1)	1·0%
Treatment											
	Increase in treatment of diabetes	NA	NA	NA	NA	7·2% (4·7–10·9)	0·6%	6·7% (5·0–8·9)	0·5%	5·0% (3·5–7·1)	1·0%
	Increase in treatment of hypertension	9·0% (4·0–15·0)	1·0%	2·4% (1·2–4·7)	0·2%	NA	NA	6·7% (5·0–9·0)	0·5%	5·0% (3·5–7·1)	1·0%
	Increase in treatment with statins	9·0% (4·0–15·0)	1·0%	NA	NA	NA	NA	NA	NA	NA	NA
Control											
	Increase in glycaemic control	NA	NA	NA	NA	7·3% (4·8–11·0)	0·5%	6·7% (5·1–9·0)	0·5%	5·1% (3·6–7·2)	0·9%
	Increase in blood pressure control	9·0% (4·0–16·0)	1·0%	2·4% (1·2–4·7)	0·2%	NA	NA	6·6% (4·9–8·9)	0·8%	4·9% (3·4–7·1)	1·1%

Data are median (IQR) or percentage points. We simulated a 10 percentage point increase in each of several potential activities: an increase in diagnosing diabetes through screening, an increase in treatment with blood pressure or glycaemia or statin medicines, and an increase in control of blood pressure or glycaemia (defined as a systolic blood pressure <130 mm Hg and diastolic blood pressure <80 mm Hg for blood pressure, and a glycated haemoglobin A_1c_ ≤7% [53 mmol/mol] or a fasting plasma glucose <7 mmol/L [126 mg/dL]). The control rate with blood pressure medicines is the proportion of people diagnosed and treated for hypertension who achieved blood pressure control (systolic blood pressure <130 mm Hg and diastolic blood pressure <80 mm Hg). Each element of the treatment cascade affects each subsequent element, such that a targeted percentage of those diagnosed are treated, and a targeted percentage of those treated are controlled. Hence, an increase in diagnosis results in an overall increase in the absolute number of people treated, which results in an overall increase in the absolute number of people controlled. NA=not applicable.

When we compared the relative effect of increased diagnosis, increased treatment, and increased control, we found that the estimates of incremental risk reduction were largely overlapping between the three activities. Although increased diagnosis implied a larger absolute number of people treated and controlled (ie, multiplying a larger number of people diagnosed by the same proportions treated and controlled), and similarly increased treatment implied a larger absolute number of people controlled, the risk levels of those already diagnosed or already treated were higher than those newly diagnosed through screening or treated for the first time. As a result, increased diagnosis through screening did not necessarily result in a larger population shift in risk than focusing on increased treatment or control of the population already having a diagnosis; in fact, the largest declines in end-stage renal disease and retinopathy occurred when focusing on increased blood pressure control rather than increased screening or treatment of diabetes or hypertension (table 2; appendix pp 64–65).

**Table 2:** Descriptive statistics on the survey respondents included in the study, WHO STEPwise approach to Surveillance, and attendant surveys (2006–18)^15^

	**Central Asia**	**East Asia**	**South Asia**	**Southeast Asia**	**North Africa and Middle East**	**Eastern sub-Saharan Africa**	**Western sub-Saharan Africa**	**Southern sub-Saharan Africa**
**Demographics**								
Total	1740	648	1018	2315	5777	1274	1526	964
Females	1025 (58·9%)	300 (46·3%)	543 (53·3%)	1538 (66·4%)	3429 (59·4%)	768 (60·3%)	810 (53·1%)	703 (72·9%)
Males	715 (41·1%)	348 (53·7%)	475 (46·7%)	777 (33·6%)	2348 (40·6%)	506 (39·7%)	716 (46·9%)	261 (27·1%)
Age (years)	54·0 (43·0–61·0)	59·8 (50·2–69·0)	47·0 (37·0–55·0)	53·0 (44·0–60·0)	55·0 (45·0–63·0)	48·0 (36·0–58·0)	40·0 (30·0–51·0)	56·0 (47·0–63·0)
**Clinical measurements**								
BMI (kg/m^2^)	29·3 (25·7–33·6)	25·1 (22·7–27·8)	24·6 (22·0–27·5)	24·8 (21·9–27·9)	28·5 (25·2–32·3)	23·5 (20·3–27·6)	22·9 (20·2–27·4)	30·1 (25·5–34·5)
Currently smokes tobacco	297 (17·1%)	173 (26·7%)	195 (19·2%)	363 (15·7%)	760 (13·2%)	122 (9·6%)	101 (6·6%)	87 (9·0%)
History of heart attack	402 (23·1%)	44 (6·8%)	113 (11·1%)	281 (12·1%)	576 (10·0%)	93 (7·3%)	85 (5·6%)	104 (10·8%)
Systolic blood pressure (mm Hg)	136·4 (123·0–155·1)	131·0 (121·0–148·0)	128·7 (118·3–143·0)	133·0 (120·7–150·0)	136·5 (123·0–151·7)	130·7 (117·0–148·3)	130·0 (118·5–147·0)	143·4 (127·9–163·5)
Diastolic blood pressure (mm Hg)	87·0 (78·5–95·5)	82·0 (79·0–90·0)	85·0 (77·3–92·3)	83·7 (76·0–92·0)	82·5 (75·0–90·0)	82·0 (74·0–91·5)	82·0 (74·5–90·3)	83·5 (76·5–93·5)
Fasting blood glucose (mmol/L)	8·2 (7·2–11·1)	8·0 (7·3–10·2)	8·4 (7·3–11·1)	8·3 (7·1–11·1)	8·3 (7·1–11·2)	7·9 (7·2–9·8)	9·0 (7·5–10·4)	8·0 (7·1–10·6)
HbA_1c_ (mmol/mol)	59·6 (49·7–78·1)	51·9 (41·0–68·6)	56·3 (48·6–77·0)	59·6 (49·7–80·3)	59·6 (48·6–78·1)	56·3 (48·6–77·1)	51·9 (44·3–68·3)	55·7 (48·6–74·0)
HbA_1c_ (%)	7·60 (6·7–9·3)	6·90 (5·9–8·4)	7·30 (6·6–9·2)	7·6 (6·7–9·5)	7·6 (6·6–9·3)	7·3 (6·6–9·2)	6·9 (6·2–8·4)	7·2 (6·6–8·9)
Total cholesterol (mmol/L)	4·8 (4·0–5·6)	5·1 (4·4–5·9)	4·7 (4·0–5·5)	4·6 (3·8–5·5)	4·3 (3·5–5·2)	4·3 (3·6–5·2)	4·3 (3·6–5·3)	4·5 (3·7–5·4)
Total cholesterol (mg/dL)	185·2 (154·7–216·2)	198·4 (170·9–226·2)	183·0 (155·0–213·0)	179·3 (148·0–211·9)	166·9 (136·1–199·2)	166·3 (138·3–199·0)	166·1 (140·0–202·9)	172·0 (144·0–209·0)
HDL cholesterol (mmol/L)	1·1 (0·9–1·4)	1·2 (1·0–1·5)	1·0 (0·8–1·1)	1·1 (0·9–1·3)	1·0 (0·8–1·2)	1·1 (0·8–1·4)	1·1 (0·9–1·4)	1·1 (0·9–1·4)
HDL cholesterol (mg/dL)	43·7 (36·4–54·1)	47·8 (40·2–57·7)	37·0 (32·0–43·9)	41·0 (33·3–50·7)	39·1 (32·0–48·0)	41·4 (32·5–52·9)	42·0 (33·3–54·5)	43·0 (35·0–54·0)
LDL cholesterol (mmol/L)	2·7 (2·0–3·4)	2·9 (2·4–3·7)	2·8 (2·2–3·4)	2·6 (1·9–3·4)	2·5 (1·8–3·2)	2·4 (1·8–3·1)	2·4 (1·8–3·1)	2·5 (1·8–3·3)
LDL cholesterol (mg/dL)	103·7 (77·8–129·9)	113·7 (91·5–142·7)	108·9 (84·7–133·2)	102·1 (75·0–132·5)	94·8 (70·2–122·2)	93·6 (69·3–120·6)	92·5 (70·2–120·7)	97·4 (69·6–126·9)
**Diabetes cascade**								
Clinical diabetes	1740 (100%)	648 (100%)	1018 (100%)	2315 (100%)	5777 (100%)	1274 (100%)	1526 (100%)	964 (100%)
Diabetes diagnosis rate	765 (44·0%)	232 (35·8%)	437 (42·9%)	1255 (54·2%)	3790 (65·6%)	417 (32·7%)	120 (7·9%)	436 (45·2%)
On treatment for diabetes	617 (35·5%)	213 (32·9%)	329 (32·3%)	1074 (46·4%)	3144 (54·4%)	297 (23·3%)	89 (5·8%)	395 (41·0%)
Diabetes treatment rate*	80·7%	91·8%	75·3%	85·6%	83·0%	71·2%	74·2%	90·6%
Current insulin use	209 (12·0%)	54 (8·3%)	105 (10·3%)	191 (8·3%)	962 (16·7%)	152 (11·9%)	55 (3·6%)	160 (16·6%)
HbA_1c_ ≤7% or fasting blood glucose <7 mmol/L	759 (43·6%)	382 (59·0%)	477 (46·9%)	1080 (46·7%)	2643 (45·8%)	620 (48·7%)	817 (53·5%)	517 (53·6%)
Diabetes control rate†	44·9%	59·2%	50·5%	56·9%	51·3%	63·0%	57·3%	52·4%
**Hypertension cascade**								
Hypertension	1103 (63·4%)	358 (55·2%)	500 (49·1%)	1263 (54·6%)	3510 (60·8%)	567 (44·5%)	639 (41·9%)	693 (71·9%)
Previous diagnosis of hypertension‡	834 (75·6%)	211 (58·9%)	337 (67·4%)	963 (76·2%)	2618 (74·6%)	288 (50·8%)	181 (28·3%)	491 (70·9%)
Medication for raised blood pressure	614 (35·3%)	181 (27·9%)	204 (20·0%)	551 (23·8%)	1862 (32·2%)	143 (11·2%)	107 (7·0%)	395 (41·0%)
Hypertension treatment rate*	73·6%	85·8%	60·5%	57·2%	71·1%	49·7%	59·1%	80·4%
SBP <130 mm Hg and DBP <80 mm Hg	385 (22·1%)	111 (17·1%)	296 (29·1%)	659 (28·5%)	1371 (23·7%)	443 (34·8%)	516 (33·8%)	181 (18·8%)
Hypertension control rate†	11·4%	3·3%	18·6%	15·6%	14·7%	11·9%	10·3%	11·6%
**Statin cascade**								
Statin treatment indicated as aged >40 years or 10-year cardiovascular event risk >20%	1378 (79·2%)	602 (92·9%)	661 (64·9%)	1905 (82·3%)	4780 (82·7%)	830 (65·1%)	728 (47·7%)	817 (84·8%)
Current statin use	133 (7·6%)	38 (5·9%)	55 (5·4%)	261 (11·3%)	981 (17·0%)	27 (2·1%)	38 (2·5%)	94 (9·8%)
Statin treatment rate*	9·7%	6·3%	8·3%	13·7%	20·5%	3·3%	5·2%	11·5%

Data are n, n (%), n/N (%), or median (IQR). We included the subset of people with diabetes mellitus (defined as fasting blood glucose >7 mmol/L, or non-fasting blood glucose >11·1 mmol/L, HbA_1c_≥6·5% [48 mmol/mol] or taking a glycaemic control medicine including insulin) across 67 countries spanning 15 world regions. For data related to blood pressure, glycaemia, and statin medicine cascades, participants had to fulfil criteria for the preceding step to be included in the denominator for the next step (eg, a person had to be diagnosed to be in the denominator of the percent treated, or had to be treated to be in the denominator of the percent controlled). Hypertension was defined by self-reported diagnosis of hypertension (SBP ≥140 mm Hg or DBP ≥90 mm Hg) on hypertensive treatment. BMI=body-mass index. DBP=diastolic blood pressure. HbA_1c_=glycated haemoglobin A_1c_. SBP=systolic blood pressure.

* For diabetes and hypertension, calculated as those diagnosed and treated, divided by those diagnosed; for statins, calculated as those indicated for treatment and treated, divided by those indicated for treatment.

† Calculated as those who have their condition controlled divided by the sum of those diagnosed and treated.

‡ Percentage of those with hypertension.

Table 4, Table 5 summarises the estimated combined effect of increasing diagnosis, treatment, and control of hypertension and diabetes on DALYs, cost, and incremental cost-effectiveness; the effect on each individual macrovascular or microvascular complication is provided in the appendix (pp 73–98, 104–81). The estimates reveal that DALYs attributable to cardiovascular events could be substantially reduced through improvements in treatment and control, whereas microvascular complications (ie, nephropathy, retinopathy, and neuropathy) were less affected by such changes.

**Table 4:** Modelled combined effect of increased hypertension and diabetes diagnosis to 60% or 80%; increased blood pressure, glycaemia, and statin treatment to 60% or 80%; and increased blood pressure and glucose control to 60% or 80%, on risk of diabetes complications

	**Oceania**	**Andean Latin America**	**Central Latin America**	**Southern Latin America**	**Caribbean**	**Central Europe**	**Eastern Europe**
**Baseline risk**							
10-year estimated cardiovascular event risk	8·00% (0·00 to 12·00)	6·00% (3·00 to 9·00)	9·00% (4·00 to 14·00)	14·00% (7·00 to 21·00)	9·00% (5·00 to 15·00)	21·00% (11·75 to 31·00)	23·00% (14·00 to 31·00)
10-year estimated heart failure risk	1·85% (0·97 to 3·37)	2·33% (1·26 to 4·57)	3·71% (1·88 to 7·68)	4·28% (2·24 to 8·21)	2·82% (1·51 to 5·49)	2·58% (1·35 to 4·02)	3·87% (1·87 to 8·35)
10-year estimated end-stage renal disease risk	7·89% (6·37 to 9·91)	8·25% (6·43 to 10·57)	7·79% (5·90 to 10·49)	7·57% (5·78 to 9·74)	5·74% (4·59 to 7·84)	5·55% (4·66 to 7·15)	6·72% (5·36 to 8·82)
10-year estimated severe vision loss risk	5·41% (3·92 to 7·53)	5·42% (4·02 to 7·22)	7·02% (5·00 to 10·15)	7·65% (5·37 to 10·79)	5·85% (4·04 to 7·86)	7·20% (4·84 to 9·48)	8·32% (5·94 to 11·19)
10-year estimated pressure sensation loss risk	7·00% (4·60 to 10·18)	7·33% (5·13 to 10·63)	9·60% (6·43 to 14·19)	10·69% (7·20 to 15·97)	7·38% (4·98 to 11·00)	9·19% (6·28 to 13·20)	10·02% (6·86 to 14·42)
**DALYs per 1000 population over 10 years**							
Baseline DALYs	12 309 (11 856 to 12 779)	1113 (1077 to 1147)	2872 (2806 to 2937)	919 (899 to 941)	2646 (2509 to 2786)	776 (746 to 806)	2017 (1953 to 2081)
60% treatment, 60% control	12 137 (11 686 to 12 605)	1101 (1065 to 1134)	2793 (2728 to 2858)	881 (861 to 903)	2548 (2412 to 2685)	710 (681 to 740)	1835 (1772 to 1898)
60% treatment, 80% control	12 137 (11 686 to 12 605)	1101 (1065 to 1134)	2793 (2728 to 2858)	881 (861 to 903)	2548 (2412 to 2685)	710 (681 to 740)	1835 (1772 to 1898)
80% treatment, 60% control	12 076 (11 627 to 12 545)	1096 (1060 to 1129)	2750 (2686 to 2814)	869 (849 to 891)	2512 (2379 to 2649)	693 (665 to 723)	1796 (1733 to 1859)
80% treatment, 80% control	12 059 (11 611 to 12 528)	1095 (1059 to 1128)	2734 (2670 to 2798)	865 (845 to 887)	2502 (2369 to 2639)	687 (658 to 716)	1773 (1710 to 1836)
60% diagnosis, 60% treatment, 60% control	12 054 (11 604 to 12 521)	1099 (1063 to 1132)	2793 (2728 to 2858)	881 (861 to 903)	2548 (2412 to 2685)	710 (681 to 740)	1831 (1768 to 1895)
60% diagnosis, 60% treatment, 80% control	12 054 (11 604 to 12 521)	1099 (1063 to 1132)	2793 (2728 to 2858)	881 (861 to 903)	2548 (2412 to 2685)	710 (681 to 740)	1831 (1768 to 1895)
60% diagnosis, 80% treatment, 60% control	11 957 (11 516 to 12 426)	1093 (1057 to 1126)	2750 (2686 to 2814)	869 (849 to 891)	2512 (2379 to 2649)	693 (665 to 723)	1792 (1728 to 1855)
60% diagnosis, 80% treatment, 80% control	11 930 (11 487 to 12 397)	1092 (1056 to 1125)	2734 (2670 to 2798)	865 (845 to 887)	2502 (2369 to 2639)	687 (658 to 716)	1767 (1704 to 1830)
80% diagnosis, 60% treatment, 60% control	11 995 (11 547 to 12 461)	1096 (1060 to 1130)	2789 (2724 to 2854)	875 (855 to 897)	2544 (2408 to 2681)	710 (680 to 739)	1817 (1754 to 1881)
80% diagnosis, 60% treatment, 80% control	11 995 (11 547 to 12 461)	1096 (1060 to 1130)	2789 (2724 to 2854)	875 (855 to 897)	2544 (2408 to 2681)	710 (680 to 739)	1817 (1754 to 1881)
80% diagnosis, 80% treatment, 60% control	11 881 (11 440 to 12 348)	1090 (1054 to 1122)	2743 (2679 to 2807)	861 (841 to 882)	2507 (2374 to 2644)	693 (664 to 722)	1773 (1709 to 1836)
80% diagnosis, 80% treatment, 80% control	11 852 (11 411 to 12 318)	1087 (1051 to 1120)	2722 (2658 to 2786)	853 (833 to 874)	2496 (2364 to 2633)	687 (658 to 716)	1748 (1684 to 1810)
Costs ($1000 per 1000 population over 10 years)*							
Baseline costs	20 751 (20 160 to 21 319)	2581 (2525 to 2640)	8412 (8275 to 8540)	3156 (3104 to 3209)	6109 (5879 to 6347)	2400 (2332 to 2471)	4505 (4383 to 4611)
60% treatment, 60% control	21 286 (20 663 to 21 841)	2679 (2616 to 2735)	8709 (8542 to 8817)	3128 (3068 to 3173)	6439 (6183 to 6666)	2399 (2321 to 2461)	4431 (4300 to 4529)
60% treatment, 80% control	21 286 (20 663 to 21 841)	2679 (2616 to 2735)	8709 (8542 to 8817)	3128 (3068 to 3173)	6439 (6183 to 6666)	2399 (2321 to 2461)	4431 (4300 to 4529)
80% treatment, 60% control	21 388 (20 760 to 21 945)	2710 (2647 to 2766)	8768 (8601 to 8873)	3112 (3054 to 3156)	6544 (6289 to 6761)	2379 (2303 to 2442)	4418 (4288 to 4516)
80% treatment, 80% control	21 538 (2 0911 to 22 096)	2739 (2677 to 2795)	8887 (8723 to 8993)	3125 (3066 to 3169)	6672 (6415 to 6888)	2397 (2320 to 2458)	4425 (4295 to 4522)
60% diagnosis, 60% treatment, 60% control	21 581 (20 947 to 22 126)	2707 (2644 to 2763)	8709 (8542 to 8817)	3128 (3068 to 3173)	6439 (6183 to 6666)	2399 (2321 to 2461)	4432 (4301 to 4530)
60% diagnosis, 60% treatment, 80% control	21 581 (20 947 to 22 126)	2707 (2644 to 2763)	8709 (8542 to 8817)	3128 (3068 to 3173)	6439 (6183 to 6666)	2399 (2321 to 2461)	4432 (4301 to 4530)
60% diagnosis, 80% treatment, 60% control	21 707 (21 077 to 22 256)	2742 (2677 to 2795)	8768 (8601 to 8873)	3112 (3054 to 3156)	6544 (6289 to 6761)	2379 (2303 to 2442)	4419 (4289 to 4516)
60% diagnosis, 80% treatment, 80% control	21 913 (21 276 to 22 462)	2774 (2711 to 2830)	8887 (8723 to 8993)	3125 (3066 to 3169)	6672 (6415 to 6888)	2397 (2320 to 2458)	4427 (4296 to 4524)
80% diagnosis, 60% treatment, 60% control	21 808 (21 167 to 22 346)	2752 (2687 to 2807)	8730 (8562 to 8837)	3133 (3073 to 3177)	6454 (6198 to 6681)	2399 (2321 to 2461)	4437 (4305 to 4534)
80% diagnosis, 60% treatment, 80% control	21 808 (21 167 to 22 346)	2752 (2687 to 2807)	8730 (8562 to 8837)	3133 (3073 to 3177)	6454 (6198 to 6681)	2399 (2321 to 2461)	4437 (4305 to 4534)
80% diagnosis, 80% treatment, 60% control	21 964 (21 319 to 22 505)	2791 (2727 to 2846)	8787 (8621 to 8889)	3113 (3053 to 3157)	6561 (6305 to 6778)	2379 (2303 to 2442)	4422 (4291 to 4520)
80% diagnosis, 80% treatment, 80% control	22 213 (21 569 to 22 756)	2832 (2769 to 2885)	8906 (8743 to 9010)	3122 (3061 to 3167)	6691 (6435 to 6907)	2397 (2320 to 2458)	4431 (4300 to 4528)
Incremental cost-effectiveness ratio (change in $ per change in DALYS)*							
60% treatment 60% control	3071 (2885 to 3122)	7503 (6967 to 7662)	3543 (3372 to 3763)	−963 (−963 to −755)	3283 (3031–3334)	−144 (−162 to −14)	−454 (−456 to −404)
60% treatment 80% control	3071 (2885 to 3122)	7503 (6967 to 7662)	3543 (3372 to 3763)	−963 (−963 to −755)	3283 (3031–3334)	−144 (−162 to −14)	−454 (−456 to −404)
80% treatment, 60% control	2735 (2561 to 2735)	7314 (6828 to 7470)	2754 (2659 to 2935)	−1020 (−1065 to −883)	3190 (3006–3235)	−352 (−360 to −252)	−431 (−434 to −392)
80% treatment, 80% control	3158 (2986 to 3170)	8722 (8172 to 8831)	3327 (3222 to 3461)	−719 (−744 to −574)	3865 (3650–3900)	−132 (−144 to −37)	−360 (−365 to −325)
60% diagnosis, 60% treatment, 60% control	3202 (3045 to 3257)	8564 (8013 to 8747)	3543 (3372 to 3763)	−963 (−963 to −755)	3283 (3031–3334)	−144 (−162 to −14)	−440 (−442 to −390)
60% diagnosis, 60% treatment, 80% control	3202 (3045 to 3257)	8564 (8013 to 8747)	3543 (3372 to 3763)	−963 (−963 to −755)	3283 (3031–3334)	−144 (−162 to −14)	−440 (−442 to −390)
60% diagnosis, 80% treatment, 60% control	2716 (2593 to 2757)	7781 (7170 to 8101)	2754 (2659 to 2935)	−1020 (−1065 to −883)	3190 (3006 to 3235)	−352 (−360 to −252)	−418 (−422 to −379)
60% diagnosis, 80% treatment, 80% control	3069 (2921 to 3097)	8721 (8340 to 8866)	3327 (3222 to 3461)	−719 (−744 to −574)	3865 (3650 to 3900)	−132 (−144 to −37)	−350 (−352 to −313)
80% diagnosis, 60% treatment, 60% control	3327 (3161 to 3375)	9870 (9391 to 10 024)	3626 (3466 to 3841)	−724 (−738 to −539)	3302 (3051 to 3351)	−145 (−157 to −9)	−389 (−392 to −336)
80% diagnosis, 60% treatment, 80% control	3327 (3161 to 3375)	9870 (9391 to 10 024)	3626 (3466 to 3841)	−724 (−738 to −539)	3302 (3051 to 3351)	−145 (−157 to −9)	−389 (−392 to −336)
80% diagnosis, 80% treatment, 60% control	2833 (2689 to 2849)	8761 (8269 to 8826)	2742 (2670 to 2926)	−885 (−899 to −742)	3193 (3011 to 3240)	−349 (−356 to −253)	−375 (−376 to −339)
80% diagnosis, 80% treatment, 80% control	3200 (3053 to 3224)	9385 (9049 to 9461)	3171 (3097 to 3303)	−631 (−657 to −520)	3854 (3634 to 3878)	−132 (−145 to −33)	−307 (−308 to −274)

Data are median (IQR). Control for blood pressure was defined as a systolic blood pressure of less than 130 mm Hg and a diastolic blood pressure of less than 80 mm Hg. Control for glycaemia was defined as a glycated haemoglobin of 7% or less (53 mmol/mol, or a fasting plasma glucose of less than 7 mmol/L (126 mg/dL). We estimated the DALY effect of cardiovascular diseases (defined as fatal and non-fatal myocardial infarction and stroke), congestive heart failure (ejection fraction of <40%, with New York Heart Association class III or IV functional limitations), end-stage renal disease (defined as estimated glomerular filtration rate <15 mL/min per 1·73 m^2^ or needing dialysis or transplant), retinopathy with severe vision loss (<20/200 visual acuity as measured by the Snellen chart), neuropathy (as measured by pressure sensation loss via the Semmes-Weinstein 5·07/10 g monofilament examination). Costs and DALYs were computed over a 10-year policy planning time horizon, simulating all persons alive or born within the next 10 years, at a 3% annual discount rate. Negative values for the incremental cost-effectiveness ratio indicate cost-savings. Overall estimates are population weighted. We note that in some cases, the incremental effect of changing diagnosis, treatment, or control rates are sufficiently small that some rows are the same as others, when subject to rounding. Costs are rounded to the nearest $1000 per 1000 population. DALYs=disability-adjusted life-years.

* 2020 International Dollars.

**Table 5:** Modelled combined effect of increased hypertension and diabetes diagnosis to 60% or 80%; increased blood pressure, glycaemia, and statin treatment to 60% or 80%; and increased blood pressure and glucose control to 60% or 80%, on risk of diabetes complications

	**Central Asia**	**East Asia**	**South Asia**	**Southeast Asia**	**North Africa and Middle East**	**Eastern sub-Saharan Africa**	**Western sub-Saharan Africa**	**Southern sub-Saharan Africa**
**Baseline risk**								
10-year estimated cardiovascular event risk	13·00% (5·00 to 26·00)	17·00% (11·00 to 25·00)	6·00% (0·00 to 11·00)	8·00% (4·00 to 13·00)	17·00% (9·00 to 25·00)	6·00% (0·00 to 11·00)	3·00% (0·00 to 8·00)	10·00% (6·00 to 17·00)
10-year estimated heart failure risk	2·95% (1·35 to 6·31)	2·84% (1·30 to 5·54)	1·86% (1·04 to 3·45)	2·63% (1·41 to 5·04)	3·06% (1·55 to 6·15)	1·69% (0·81 to 3·19)	0·93% (0·48 to 2·01)	3·20% (1·52 to 6·60)
10-year estimated end-stage renal disease risk	7·26% (5·75 to 9·33)	5·69% (4·52 to 7·38)	7·68% (5·89 to 9·66)	7·12% (5·61 to 9·34)	6·67% (5·18 to 8·81)	7·41% (5·64 to 9·56)	7·79% (6·27 to 9·83)	6·11% (4·78 to 7·91)
10-year estimated severe vision loss risk	6·67% (4·61 to 9·48)	6·66% (4·84 to 9·27)	4·94% (3·69 to 6·68)	6·17% (4·46 to 8·73)	6·32% (4·49 to 8·81)	4·70% (3·22 to 6·90)	3·81% (2·72 to 5·55)	6·45% (4·39 to 9·20)
10-year estimated pressure sensation loss risk	8·57% (5·54 to 12·67)	8·91% (5·94 to 13·19)	6·70% (4·56 to 9·52)	7·75% (5·21 to 11·46)	8·13% (5·48 to 12·04)	6·23% (3·81 to 9·18)	4·69% (2·93 to 7·46)	8·21% (5·35 to 12·13)
**DALYs per 1000 population over 10 years**								
Baseline DALYs	8197 (7872 to 8527)	804 (786 to 822)	2887 (2812 to 2964)	8740 (8340 to 9130)	11 079 (10 797 to 11 353)	12 152 (11 463 to 12 880)	10 798 (10 287 to 11 321)	4058 (3855 to 4276)
60% treatment, 60% control	7794 (7472 to 8121)	778 (760 to 797)	2850 (2776 to 2926)	8555 (8158 to 8943)	10 572 (10 294 to 10 843)	11 992 (11 306 to 12 715)	10 755 (10 245 to 11 275)	3952 (3751 to 4169)
60% treatment, 80% control	7794 (7472 to 8121)	778 (760 to 797)	2850 (2776 to 2926)	8555 (8158 to 8943)	10 572 (10 294 to 10 843)	11 992 (11 306 to 12 715)	10 755 (10 245 to 11 275)	3952 (3751 to 4169)
80% treatment, 60% control	7686 (7364 to 8010)	771 (753 to 789)	2838 (2765 to 2915)	8495 (8096 to 8883)	10 415 (10140 to 10686)	11 941 (11 254 to 12 662)	10 735 (10 227 to 11 254)	3917 (3718 to 4137)
80% treatment, 80% control	7636 (7315 to 7959)	768 (750 to 787)	2835 (2761 to 2911)	8475 (8078 to 8862)	10 361 (10 085 to 10 631)	11 921 (11 234 to 12 643)	10 730 (10 222 to 11 249)	3904 (3705 to 4123)
60% diagnosis, 60% treatment, 60% control	7746 (7425 to 8073)	767 (749 to 785)	2838 (2764 to 2914)	8516 (8120 to 8901)	10 558 (10 280 to 10 829)	11 925 (11242 to 12648)	10 653 (10 147 to 11 173)	3934 (3734 to 4151)
60% diagnosis, 60% treatment, 80% control	7746 (7425 to 8073)	767 (749 to 785)	2838 (2764 to 2914)	8516 (8120 to 8901)	10 558 (10 280 to 10 829)	11 925 (11242 to 12648)	10 653 (10 147 to 11 173)	3934 (3734 to 4151)
60% diagnosis, 80% treatment, 60% control	7620 (7299 to 7945)	756 (738 to 775)	2815 (2742 to 2891)	8405 (8018 to 8793)	10 395 (10 119 to 10 664)	11 833 (11 150 to 12 549)	10 581 (10 082 to 11 097)	3895 (3696 to 4114)
60% diagnosis, 80% treatment, 80% control	7559 (7240 to 7884)	753 (735 to 771)	2805 (2733 to 2882)	8370 (7986 to 8758)	10 334 (10 059 to 10 604)	11 776 (11 095 to 12 489)	10 557 (10 059 to 11 073)	3880 (3682 to 4098)
80% diagnosis, 60% treatment, 60% control	7687 (7366 to 8013)	759 (741 to 777)	2828 (2755 to 2905)	8480 (8085 to 8865)	10 504 (10 227 to 10 774)	11 882 (11 200 to 12 603)	10 615 (10 110 to 11 132)	3912 (3711 to 4127)
80% diagnosis, 60% treatment, 80% control	7687 (7366 to 8013)	759 (741 to 777)	2828 (2755 to 2905)	8480 (8085 to 8865)	10 504 (10 227 to 10 774)	11 882 (11 200 to 12 603)	10 615 (10 110 to 11 132)	3912 (3711 to 4127)
80% diagnosis, 80% treatment, 60% control	7545 (7223 to 7867)	745 (728 to 764)	2799 (2727 to 2876)	8354 (7967 to 8741)	10 316 (10 043 to 10 586)	11 769 (11 084 to 12 480)	10 530 (10 033 to 11 043)	3867 (3669 to 4084)
80% diagnosis, 80% treatment, 80% control	7476 (7156 to 7799)	741 (724 to 760)	2792 (2719 to 2868)	8318 (7934 to 8705)	10 247 (9974 to 10 517)	11 714 (11 033 to 12 426)	10 503 (10 007 to 11 017)	3850 (3652 to 4066)
Costs ($1000 per 1000 population over 10 years)*								
Baseline costs	16 204 (15 679 to 16 705)	2023 (1988 to 2061)	4406 (4317 to 4495)	17 914 (17 349 to 18 526)	19 718 (19 322 to 20 108)	19 323 (18 481 to 20 199)	15 170 (14 581 to 15 765)	8088 (7776 to 8382)
60% treatment, 60% control	16 135 (15 579 to 1 6613)	2080 (2041 to 2117)	4437 (4344 to 4524)	18 272 (17 663 to 18 864)	19 779 (19 343 to 20 135)	19 488 (18 621 to 20 350)	15 266 (14 668 to 15 859)	8101 (7774 to 8382)
60% treatment, 80% control	16 135 (15 579 to 16 613)	2080 (2041 to 2117)	4437 (4344 to 4524)	18 272 (17 663 to 1 8864)	19 779 (19 343 to 20 135)	19 488 (18 621 to 20 350)	15 266 (14 668 to 15 859)	8101 (7774 to 8382)
80% treatment, 60% control	16 113 (15 557 to 16 591)	2090 (2051 to 2127)	4444 (4352 to 4530)	18 366 (17 756 to 18 954)	19 784 (19 352 to 20 139)	19 518 (18 648 to 20 381)	15 280 (14 683 to 15 873)	8098 (7771 to 8377)
80% treatment, 80% control	16 155 (15 599 to 16 631)	2115 (2076 to 2152)	4454 (4361 to 4539)	18 509 (17 899 to 19 096)	19 874 (19 440 to 20 227)	19 563 (18 694 to 20 425)	15 306 (14 709 to 15 899)	8123 (7796 to 8401)
60% diagnosis, 60% treatment, 60% control	16 219 (15 659 to 16 693)	2132 (2092 to 2168)	4460 (4365 to 4544)	18 579 (17 964 to 19 168)	19 793 (19 356 to 20 148)	19 647 (18 765 to 20 494)	15 630 (15 019 to 16 213)	8126 (7797 to 8405)
60% diagnosis, 60% treatment, 80% control	16 219 (15 659 to 16 693)	2132 (2092 to 2168)	4460 (4365 to 4544)	18 579 (17 964 to 19 168)	19 793 (19 356 to 20 148)	19 647 (18 765 to 20 494)	15 630 (15 019 to 16 213)	8126 (7797 to 8405)
60% diagnosis, 80% treatment, 60% control	16 194 (15 637 to 16 667)	2147 (2107 to 2183)	4463 (4369 to 4544)	18 675 (18 061 to 19 261)	19 797 (19 367 to 20 151)	19 669 (18 787 to 20 508)	15 677 (15 066 to 16 250)	8126 (7796 to 8403)
60% diagnosis, 80% treatment, 80% control	16 252 (15 693 to 16 720)	2183 (2144 to 2219)	4474 (4377 to 4555)	18 864 (18 251 to 19 451)	19 882 (19 448 to 20 234)	19 725 (18 837 to 20 565)	15 759 (15 149 to 16 333)	8155 (7826 to 8431)
80% diagnosis, 60% treatment, 60% control	16 286 (15 719 to 16 753)	2177 (2136 to 2212)	4478 (4382 to 4562)	18 814 (18 192 to 19 399)	19 846 (19 406 to 20 197)	19 756 (18 869 to 20 596)	15 789 (15 171 to 16 368)	8159 (7827 to 8435)
80% diagnosis, 60% treatment, 80% control	16 286 (15 719 to 16 753)	2177 (2136 to 2212)	4478 (4382 to 4562)	18 814 (18 192 to 19 399)	19 846 (19 406 to 20 197)	19 756 (18 869 to 20 596)	15 789 (15 171 to 16 368)	8159 (7827 to 8435)
80% diagnosis, 80% treatment, 60% control	16 260 (15 698 to 16 727)	2196 (2155 to 2231)	4482 (4386 to 4561)	18 931 (18 317 to 19 519)	19 844 (19 412 to 20 196)	19 783 (18 892 to 20 621)	15 859 (15 239 to 16 426)	8160 (7829 to 8434)
80% diagnosis, 80% treatment, 80% control	16 331 (15 769 to 16 796)	2242 (2201 to 2276)	4497 (4398 to 4577)	19 167 (18 552 to 19 752)	19 933 (19 502 to 20 288)	19 869 (18 973 to 20 692)	15 967 (15 349 to 16 535)	8197 (7864 to 8469)
Incremental cost-effectiveness ratio (change in $ per change in DALYS)*								
60% treatment 60% control	−230 (−246 to −171)	2162 (2064 to 2229)	773 (725 to 846)	1854 (1675 to 1935)	54 (42 to 120)	963 (852 to 1031)	2188 (1882 to 2236)	4 (−20 to 122)
60% treatment 80% control	−230 (−246 to −171)	2162 (2064 to 2229)	773 (725 to 846)	1854 (1675 to 1935)	54 (42 to 120)	963 (852 to 1031)	2188 (1882 to 2236)	4 (−20 to 122)
80% treatment, 60% control	−225 (−236 to −179)	1991 (1909 to 2038)	722 (718 to 793)	1751 (1645 to 1843)	47 (46 to 101)	870 (764 to 924)	1734 (1511 to 1790)	−34 (−40 to 71)
80% treatment, 80% control	−133 (−140 to −88)	2556 (2477 to 2602)	857 (847 to 916)	2171 (2051 to 2238)	168 (163 to 217)	990 (896 to 1041)	1988 (1763 to 2045)	126 (126 to 224)
60% diagnosis, 60% treatment, 60% control	−27 (−43 to 33)	2909 (2810 to 2983)	1014 (967 to 1090)	2914 (2690 to 2971)	78 (65 to 144)	1340 (1225 to 1428)	3171 (2951 to 3200)	191 (167 to 304)
60% diagnosis, 60% treatment, 80% control	−27 (−43 to 33)	2909 (2810 to 2983)	1014 (967 to 1090)	2914 (2690 to 2971)	78 (65 to 144)	1340 (1225 to 1428)	3171 (2951 to 3200)	191 (167 to 304)
60% diagnosis, 80% treatment, 60% control	−67 (−73 to −18)	2571 (2501 to 2622)	727 (694 to 798)	2271 (2112 to 2280)	66 (64 to 117)	987 (923 to 1083)	2334 (2159 to 2365)	130 (123 to 232)
60% diagnosis, 80% treatment, 80% control	24 (22 to 75)	3117 (3051 to 3167)	747 (737 to 826)	2565 (2424 to 2609)	171 (169 to 221)	996 (910 to 1067)	2445 (2290 to 2488)	282 (278 to 374)
80% diagnosis, 60% treatment, 60% control	94 (78 to 160)	3336 (3244 to 3423)	1161 (1111 to 1234)	3415 (3175 to 3461)	157 (145 to 223)	1511 (1399 to 1600)	3370 (3124 to 3408)	366 (341 to 481)
80% diagnosis, 60% treatment, 80% control	94 (78 to 160)	3336 (3244 to 3423)	1161 (1111 to 1234)	3415 (3175 to 3461)	157 (145 to 223)	1511 (1399 to 1600)	3370 (3124 to 3408)	366 (341 to 481)
80% diagnosis, 80% treatment, 60% control	34 (30 to 86)	2913 (2858 to 2986)	793 (771 to 862)	2631 (2490 to 2662)	118 (116 to 166)	1115 (1027 to 1198)	2570 (2367 to 2605)	281 (273 to 378)
80% diagnosis, 80% treatment, 80% control	127 (124 to 176)	3468 (3411 to 3528)	873 (854 to 951)	2966 (2828 to 3020)	219 (216 to 259)	1147 (1083 to 1246)	2700 (2522 to 2752)	429 (420 to 523)

Data are median (IQR). Control for blood pressure was defined as a systolic blood pressure of less than 130 mm Hg and a diastolic blood pressure of less than 80 mm Hg. Control for glycaemia was defined as a glycated haemoglobin of 7% or less (53 mmol/mol), or a fasting plasma glucose of less than 7 mmol/L (126 mg/dL). We estimated the DALY effect of cardiovascular diseases (defined as fatal and non-fatal myocardial infarction and stroke), congestive heart failure (ejection fraction of <40%, with New York Heart Association class III or IV functional limitations), end-stage renal disease (defined as estimated glomerular filtration rate <15 mL/min per 1·73 m^2^ or needing dialysis or transplant), retinopathy with severe vision loss (<20/200 visual acuity as measured by the Snellen chart), neuropathy (as measured by pressure sensation loss via the Semmes-Weinstein 5·07/10 g monofilament examination). Costs and DALYs were computed over a 10-year policy planning time horizon, simulating all persons alive or born within the next 10 years, at a 3% annual discount rate. Negative values for the incremental cost-effectiveness ratio indicate cost-savings. Overall estimates are population weighted. We note that in some cases, the incremental effect of changing diagnosis, treatment, or control rates are sufficiently small that some rows are the same as others, when subject to rounding. Costs are rounded to the nearest $1000 per 1000 population. DALYs=disability-adjusted life-years.

* 2020 International Dollars.

At the baseline levels of diagnosis, treatment, and control observed in the survey, we estimated a population-weighted median loss of 1161 DALYs per 1000 population over 10 years (IQR 1103–1218) from the simulated outcomes (table 3). When increasing treatment across all countries to 60% for blood pressure, glycaemia, and statin medicines, and increasing control across all countries for blood pressure and glycaemia to 60% (no additional screening), we estimated that the populations would experience a median loss of 1128 DALYs per 1000 population over 10 years (IQR 1069–1182)—a 2·8% reduction from the baseline of 1161 DALYs per 1000 population—with most of the reduction from baseline occurring from reduced cardiovascular events (down from 143 to 124 DALYs per 1000 population; appendix p 73). Increasing screening in this scenario to achieve 60% diagnosis across all countries (for 60% diagnosis, 60% treatment, and 60% control) reduced the median DALYs lost by five DALYs per 1000 population (ie, 1123 DALYs per 1000 population over 10 years [IQR 1066–1182], relative to 60% treatment and control with no additional screening). Alternatively, increasing control levels to 80% for blood pressure and glycaemia medicines (ie, no additional screening, 60% treatment, and 80% control), reduced the median DALYs by less than one per 1000 population, relative to the 60% treatment and control (no additional screening) data (table 3).

When increasing treatment across all countries to 80% for blood pressure, glycaemia, and statin medicines, and increasing control across all countries for blood pressure and glycaemia to 80% (no additional screening), we estimated a median population-weighted loss of 1115 DALYs per 1000 population over 10 years (IQR 1059–1170)—a 4·0% reduction from the baseline of 1161 DALYs per 1000 population—with most of the reduction from baseline occurring from reduced cardiovascular events (down from a median of 143 to 117 DALYs per 1000 population; appendix p 73). Increasing screening in this scenario to 80% across all countries (for 80% screening, 80% treatment, and 80% control) reduced the total DALYs lost to 1097 DALYs per 1000 population over 10 years (IQR 1051–1155), primarily from reduced cardiovascular events when the newly diagnosed individuals received blood pressure and statin treatment (reducing cardiovascular event DALYs from 143 to 100 DALYs per 1000 population; appendix p 77).

At the baseline levels of diagnosis, treatment, and control observed in the survey, if patients were to receive recommended management for complications, we estimated the median population-weighted total treatment costs (ie, costs of treating and controlling risk factors and managing adverse outcomes) would be about $2 223 000 per 1000 people with diabetes over 10 years (IQR 2 142 000–2 280 000; table 3).

When we simulated the effect of increasing treatment and control with or without increased screening, the majority of decreased costs were from reduced cardiovascular event management costs, whereas the majority of increased costs were from increased blood pressure treatment—which, although low on an individual level, was large when applied to the population—resulting in an overall slight increase in net total cost. For example, when increasing treatment across all countries to 60% for blood pressure, glycaemia, and statin medicines, and increasing control across all countries for blood pressure and glycaemia to 60% (no new screening), we estimated that the populations would experience a median cost of $2 678 589 516 per 1000 over 10 years (IQR 2 616 089–2 735 027; a 20·5% increase from the baseline of $2 222 882) from the simulated outcomes, with the largest decrease from baseline from reduced cardiovascular events (down from $79 258 to $65 327 per 1000) offset by an increase in costs of medications for risk factors (up from $24 929 to $59 662 per 1000; table 3). When computing the individual country ratios of incremental cost to incremental DALYs, we arrived at a population-weighted international median incremental cost-effectiveness ratio of $1206 per DALY averted for achieving the 60% diagnosis, 60% treatment, and 60% control target (IQR 1130–1281). The incremental cost-effectiveness ratio varied across regions due to differences in the baseline levels of diagnosis, treatment, and control (table 3), and therefore the added value of screening, diagnosis, and treatment differed across regions such that those with higher baseline levels had less incremental benefits (diminishing returns).

By contrast, we arrived at a population-weighted international median incremental cost-effectiveness ratio of $1362 per DALY averted for achieving the 80% diagnosis, 80% treatment, and 80% control target (IQR 1304–1409; table 3). Detailed costs by region, cost subitem, and screening, treatment, and control scenario are provided in the appendix (pp 72–98).

## Discussion

In this study of evidence-based targets to increase comprehensive diagnosis, treatment, and control of diabetes and its associated cardiovascular risk factors in LMICs, we found that—despite marked variations across regions—the baseline rate of treatment and control was generally much lower than the rate of diagnosis for both diabetes and hypertension, and the use of statins for those indicated for statin treatment was particularly low. In this model-based analysis, the greatest reductions in cardiovascular events were achieved through increased treatment with blood pressure and statin medicines, and increased titration of blood pressure medicines to achieve blood pressure targets. However, the largest effect on end-stage renal disease came from increasing treatment with glycaemic medicines, followed by increasing diagnosis of diabetes with existing rates of treatment and control, and finally from increasing rates of blood pressure control. Hence, when considered altogether, the treatment and control of blood pressure was among the most important strategies for reducing DALYs attributable to diabetes complications.

As WHO contemplates setting global targets for diagnosis, treatment, and control, we estimated that a target for 80% diagnosis, 80% treatment, and 80% control would be expected to reduce the DALYs lost from diabetes complications primarily from reduced cardiovascular events, while increasing the cost for treatment and control resulting in an incremental cost-effectiveness ratio of $1362 per DALY averted. The increased cost of blood pressure, glycaemic, and statin medicines was partially but not fully offset by the decreased cost of managing cardiovascular events. Increasing screening had only a small incremental benefit over diagnosis and control, given that although it increased numbers of those treated and controlled, many people who were at high risk had already progressed through the cascade beyond screening.

Our analyses are subject to several important limitations. Diagnosis of diabetes and hypertension was based on criteria that are accepted in epidemiology studies, but these methods might overestimate or underestimate the numbers that would be diagnosed in a clinical setting.^28^ In LMICs in particular, cross-sectional data might not reveal systematically underdiagnosed conditions. Additionally, our microvascular risk equations were derived among cohorts and trials based largely in the USA, and despite having coefficients to account for Latino or African heritage, it would be helpful to develop longitudinal cohort data from LMICs to account for further potential racial, ethnic, geographical, or other unmeasured covariates that might recalibrate the microvascular equations for other settings. We also did not account for any behavioural change that can occur at the individual level on receiving a diagnosis of hypertension or diabetes. Furthermore, we are unable to extract age-specific disability weights from our data sources, and so we could not fully capture how disutility of complications worsens with older age. Also, we did not simulate targeting of a specific LDL concentration for statin treatment, given current evidence favouring risk-based treatment rather than target-based treatment.^24^, ^25^ Future changes to statin therapy might switch back to a target-based approach that would require further analysis. Moreover, we have assumed in this study that most participants have type 2 diabetes given their age. The survey results do not enable us to distinguish between the types of diabetes, and we might have inadvertently included a small number of people with type 1 diabetes. Finally, data limitations exist for cost estimates in that they are often approximations with widely varying quality and geographical representation, and the actual cost that the health system experiences from reaching a target such as 80% diagnosis, 80% treatment, and 80% control might not be the costs that would be experienced if guidelines were being perfectly adhered to.

Our findings have important implications, such as emphasising the need for scale-up of blood pressure and statin medication treatment initiation and blood pressure medication titration to reduce the cardiovascular event rate from diabetes. In the future, we aim to understand what factors specifically contribute to the improvement of screening, treatment, and control of risk factors for diabetes complications across LMICs. Although the data used here are cross-sectional, efforts to repeat these analyses are underway, and, if augmented by cost and disability assessments, might help to enhance the field's understanding of what targets to set and how to maximise the potential for strategic investments to improve the population health of those with diabetes.

## Data sharing

Individual de-identified participant data including data dictionaries are available alongside statistical code for researchers to do non-commercial academic studies as detailed in the appendix (p 1).

## Declaration of interests

SB reports grants from the US National Institutes of Health (NIH) and US Centers for Disease Control and Prevention; consulting fees from the Clinton Health Access Initiative and University of California San Francisco; patents pending for a multi-model patient outreach system; unpaid leadership roles at La Scuola International School and Columbia University Global Research Analytics for Population Health; and stock options at Collective Health, outside the submitted work. DF reports volunteer affiliations with Wuqu' Kawoq and GlucoSalud, outside the submitted work. RA reports contracts with Novo Nordisk, outside the submitted work. TB reports grants from the NIH–National Institute of Allergy and Infectious Diseases, NIH–National Institute on Aging, NIH, National Institute of Child Health and Human Development, Wellcome, Alexander von Humboldt Foundation, UNAIDS, German Research Foundation, European Union, German Federal Ministry of Education and Research, German Federal Ministry of Environment, Nature Conservation and Nuclear Safety, German Federal Ministry of Health, KfW, Else Kröner Foundation, African Academy of Science, European and Developing Countries Clinical Trials Partnership, and the Bill & Melinda Gates Foundation. All other authors declare no competing interests.
