# Correction to *Lancet Glob Health* 2021; 9: e1539–52

**DOI:** 10.1016/S2214-109X(21)00585-4

**Published:** 2021-12-08

*Basu S, Flood D, Geldsetzer P, et al. Estimated effect of increased diagnosis, treatment, and control of diabetes and its associated cardiovascular risk factors among low-income and middle-income countries: a microsimulation model.* Lancet Glob Health *2021; **9:** e1539–52*—In this Article, Cara Ebert's affiliation should have been RWI – Leibniz Institute for Economic Research, Essen (Berlin Office), Germany. This correction has been made as of Dec 8, 2021.

