# Supplementary material for: Estimated effect of increased diagnosis, treatment, and control of diabetes and its associated cardiovascular risk factors among low-income and middle-income countries: a microsimulation model
Source: Lancet Glob Health. 2021 Sep 22;9(11):e1539–52. doi: 10.1016/S2214-109X(21)00340-5 (PMC8526364; doi:10.1016/S2214-109X(21)00340-5)
Supplement: Supplementary appendix [file mmc1.pdf]

THE LANCET
Global Health

Supplementary appendix

This appendix formed part of the original submission and has been peer reviewed. We post it as supplied by the authors.

Supplement to: Basu S, Flood D, Geldsetzer P, et al. Estimated effect of increased diagnosis, treatment, and control of diabetes and its associated cardiovascular risk factors among low-income and middle-income countries: a microsimulation model. *Lancet Glob Health* 2021; published online Sept 22. http://dx.doi.org/10.1016/S2214-109X(21)00340-5.

Appendix

Model code for reproduction and extension of the analysis is posted at: https://github.com/sanjaybasu/global-diabetes-targets.

Baseline absolute risk estimation

Baseline cardiovascular disease risk was estimated by the 2019 laboratory-based WHO cardiovascular disease risk estimates by region,[1] using the laboratory or office-based estimates for future cardiovascular event risk. Where it was possible to use laboratory (in countries with cholesterol measurements reported), this was preferred over office as the laboratory-based equations have been shown to be more reliable than use of office-based measures in people with diabetes.[1] When cholesterol values were not available, the office-based equations were used. The baseline risk of all other outcomes (congestive heart failure exacerbations; renal failure/end-stage renal disease (ESRD) due to hypertensive or diabetic nephropathy; severe vision loss attributable to diabetic retinopathy; and pressure sensation loss or further severe diabetic neuropathy) were estimated using the Risk Equations for Complications of Type 2 Diabetes (RECODe).[2,3] Case fatality rates following events and all-cause mortality was obtained from the Institute for Health Metrics and Evaluation.[4] The equations are provided in **Appendix Table 3**. We note that a recent evaluation of the WHO cardiovascular equations highlights the importance of their calibration to LMIC settings,[1,5] while a recent head-to-head comparison favored the RECODe equation set including for heart failure, due to higher predictive accuracy across international datasets.[5] Ten-year risks were chosen because this was the time period against which the risk equations were validated.

Risk estimates for individuals (with inputs of age, sex, race/ethnicity, tobacco smoking status, systolic blood pressure, cardiovascular disease history, medication treatment with blood pressure agents, treatment with statins, hemoglobin A1c, total and high-density lipoprotein cholesterol, serum creatinine, and urine microalbumin/creatinine ratio), can be obtained using an online calculator and associated open source code in *R*

(https://sanjaybasu.shinyapps.io/recode and https://github.com/sanjaybasu/t2dmriskeqns).

Relative risk reduction equations

With blood pressure treatment, the relative risk reduction for cardiovascular disease events was estimated using the Smith-Spangler equation[6] calculating relative risk (RR) as a function of age (in years) and change in systolic blood pressure (ΔSBP) :

[1] $$RR = 2^{\Delta SBP(-0.0000184775 \times age^2 + 0.001584 \times age + 0.028672}$$

where the change in systolic blood pressure by medication class and dosage was obtained from a meta-analysis of 354 randomized trials.[7]

With angiotensin-converting-enzyme inhibitor therapy, the relative risk reduction in congestive heart failure exacerbations was estimated as 22% (95% CI: 6%, 35%).[8] With blood pressure treatment, the relative risk for renal failure conditional on baseline systolic blood pressure (SBP) and reduction in systolic blood pressure (ΔSBP) was estimated as[9]:

[2] $$RR = \left(\frac{SBP - \Delta SBP}{SBP}\right)^{2.5640}$$

and the relative risk for retinopathy among those with diabetes was estimated as[9]:

[3] $$RR = \left(\frac{SBP - \Delta SBP}{SBP}\right)^{6.4249}$$

With statin treatment, the relative risk reduction for myocardial infarction and stroke from statin treatment was estimated as 21% (95% CI: 19%, 23%).[10]

With glycemic treatment of type 2 diabetes, the relative risk for nephropathy conditional on baseline hemoglobin A1c (A1c) and reduction in systolic blood pressure (ΔA1c) was estimated as[9]:

[4] $$RR = \left(\frac{A1c - \Delta A1c}{A1c}\right)^{1.1025}$$

while the relative risk for neuropathy was estimated as[9]:

[5] $$RR = \left(\frac{A1c - \Delta A1c}{A1c}\right)^{1.4325}$$

and the relative risk for retinopathy as[9]:

[6] $$RR = \left(\frac{A1c - \Delta A1c}{A1c}\right)^{2.5144}$$

The reduction in baseline hemoglobin A1c by medication class and dosage was obtained from a review of treatment agents, and estimated a 1.3% reduction from metformin (95% CI: 1.0%, 1.5%), and 1.5% from sulfonylureas (95% CI: 1.0%, 2.0%).[11]

DALY and cost calculations

The Consolidated Health Economic Evaluation Reporting Standards (CHEERS) checklist is provided in **Appendix Table 1**.[12] DALY disutility weight estimates for each outcome are provided in **Appendix Table 4.**[13] The weight estimates were elicited through international standardized preference elicitation surveys.[13] The method of calculating DALYs from disutilities specified by Rushby and Hanson was used, to include the sum of years of life lost and years of life lived in disability from each outcome.[14] For cost estimation, the care components used in the microsimulation model are as detailed in **Appendix Table 5.** The face facility of the cost estimates was established by ensuring that in every case, the uncertainty ranges of the cost estimates included the point estimates from country-specific costing exercises found in the literature.[15–18]

Survey Data Details

In this Appendix, we provide further details of the STEPS and other nationally-representative surveys used to populate our microsimulation model. In particular, we highlight the survey response rates in **Appendix Table 7**, the survey flow chart in **Appendix Figure 43**, the diabetes biomarker devices used by country in **Appendix Table 8**, the blood pressure measurement devices used by country in **Appendix Table 9**, and the lipid measurement devices used by country in **Appendix Table 10.**

The generic versions of the World Health Organization STEPwise approach to noncommunicable disease surveillance (WHO STEPS) instrument are available online (accessed March 10, 2020) at the following links:
Version 2.1:
https://www.who.int/ncds/surveillance/steps/STEPS_Instrument_v2.1.pdf
Version 3.2:
https://www.who.int/ncds/surveillance/steps/instrument/STEPS_Instrument_V3.2.pdf

Data included in this study are publicly available for 28 of the 33 included country surveys. Microdata can be downloaded (upon free registration) at the following links:
Chile: https://www.minsal.cl/estudios_encuestas_salud/
STEPS Microdata repository:
https://extranet.who.int/ncdsmicrodata/index.php/catalog/STEPS)
For countries without publically available microdata (Belize, Burkina Faso, Costa Rica, Seychelles, and St. Vincent & the Grenadines), please contact Paul Martin at pmartin@hsph.harvard.edu. For Guyana and St. Vincent and the Grenadines, which are member countries of the Caribbean Public Health Agency (CARPHA): Data were originally shared through a Data Use Agreement signed with the Executive Director of CARPHA. The Senior Technical Officer for NCDs (Dr. Glennis Andall- Brereton, andallgl@carpha.org) can be contacted, if necessary. After our Data Use Agreement was signed, microdata for the Guyana 2016 STEPS survey also became publicly available on the WHO STEPS Microdata repository.ch

For the STEPS surveys, the inclusion criteria were as follows:

Inclusion criteria for a survey:

(1) The survey was conducted during or after 2006; in cases where two surveys were available for a particular country, the most recent survey was used;
(2) The survey data were made available at the individual level;
(3) The survey was conducted in an upper-middle, lower-middle or low-income country according to the World Bank in the year the survey was conducted;[1]
(4) The survey was nationally representative;
(5) The survey had a response rate ≥50%;
(6) The survey contained a biomarker for diabetes (either a glucose measurement or HbA1c) and cholesterol;
(7) The survey contained blood pressure measurements;
(8) The surveys contained data the health service indicators of diabetes care defined in this paper

We first identified all countries in which a World Health Organization (WHO) Stepwise Approach to Surveillance (STEPS) survey had been conducted during a year in which the

country fell into an eligible World Bank country income category of low-income or middle-income. Prior to the STEPS surveys being made available in the WHO STEPS survey Central Data Catalog in 2019, we systematically requested each eligible STEPS survey from a list of these surveys that the WHO maintains on their website. The research team contacted the responsible party for each survey, based on the information provided on this website. If the contact information was outdated or unavailable, the authors relied on publications utilizing STEPS data and electronic searches of the survey or contact name. For the Caribbean region, country involvement was facilitated by the Caribbean Public Health Agency (CARPHA).

In 2019-2020, additional eligible surveys were downloaded from the Central Data Catalog. The search words used in the WHO Central Data Catalog were: (1) STEPS collection, (2) surveys conducted ≥2008, (3) low-and middle-income countries. The final date for inclusion of a STEPS survey was April 8, 2020.

Afghanistan STEPS 2018

In the sampling methodology districts are used as primary sampling units (PSUs), villages/blocks are the SSUs, and households within districts serves as TSUs. Based on the guidelines of the WHO, the total number of the PSUs within a sampling frame should be greater than 100 among which 50-100 PSUs should be randomly selected. The total number of districts in 34 provinces of Afghanistan is 417. From 417 districts 55districts were selected based on the available resources using Stepwise-Approach XLs form.

The total sample size was distributed proportionate to the size of the districts, then the sample size of the districts was divided by 15 (maximum number of the household to interviewed within an EA) and number of EAs within each district was calculated. Using the EPI sampling frame EAs were selected within each district. Within each EA the total number of the households were calculated and it was divided to calculate the sampling interval. The household with each randomly selected, within each household interview with a randomly selected male or female members was conducted.

Age range of participants included: 18-69 years

Source: Afghanistan STEPS 2018 Report. Available at:
https://extranet.who.int/ncdsmicrodata/index.php/catalog/782

Algeria: STEPS 2016-2017

A multi-stage cluster sample of households. One individual within the age range of the survey was selected per household. Analysis weights were calculated by taking the inverse of the probability of selection of each participant. These weights were adjusted for differences in the age-sex composition of the sample population as compared to the target population.
Different weight variables are available per Step:
wStep1 - for interview data
wStep2 - for physical measures
wStep3 - for biochemical measures
This allows for differences in the weight calculation for each Step of the survey as the age-sex composition of the respondents to each Step can differ slightly due to refusal or drop out. Additionally, some countries perform subsampling for Step 2 and/or Step 3. When no subsampling is done and response rates do not differ across Steps of the survey, the 3 weight variables will be the same.
Age range of participants included: 18-69 years
Source: no report or fact sheet available. Sampling information obtained from:
https://extranet.who.int/ncdsmicrodata/index.php/catalog/91/study-description

Armenia: STEPS 2016

The STEPS survey of non-communicable disease (NCD) risk factors in Republic of Armenia was carried out from September 2016to December 2016. The Republic of Armenia carried out Step 1, Step 2, and Step3. Socio demographic and behavioral information was collected in Step 1. Physical measurements such as height, weight and blood pressure were collected in Step 2. Biochemical measurements were collected to assess blood glucose and cholesterol levels and urine analyze to assess salt intake levels in Step 3. The survey was a population-based survey of adults aged 18-69A cluster sample design was used to produce representative data for that age range in Armenia. A total of2349adults participated in the survey. The overall response rate was42%.
Age range of participants included: 18-69 years
Source: Armenia STEPS Fact Sheet. Available at:
https://extranet.who.int/ncdsmicrodata/index.php/catalog/102

Azerbaijan: STEPS 2017

A multi-stage cluster sample of households. One individual within the age range of the survey was selected per household. Analysis weights were calculated by taking the inverse of the probability of selection of each participant. These weights were adjusted for differences in the age-sex composition of the sample population as compared to the target population.

Different weight variables are available per Step:

wStep1 - for interview data

wStep2 - for physical measures

wStep3 - for biochemical measures

This allows for differences in the weight calculation for each Step of the survey as the age-sex composition of the respondents to each Step can differ slightly due to refusal or drop out. Additionally, some countries perform subsampling for Step 2 and/or Step 3. When no subsampling is done and response rates do not differ across Steps of the survey, the 3 weight variables will be the same.

Age range of participants included: 18-69 years

Source: no report or fact sheet available. Sampling information obtained from: https://extranet.who.int/ncdsmicrodata/index.php/catalog/127/studydescription#page=overview&tab=study-desc

Bangladesh: STEPS 2018

Sampling Procedure

A multistage complex sampling design was used to produce representative data for that age range in Bangladesh.

Response Rate

The overall response rate was 83.8%.

Weighting

Analysis weights were calculated by taking the inverse of the probability of selection of each participant. These weights were adjusted for differences in the age-sex composition of the sample population as compared to the target population.

Different weight variables are available per Step:

wStep1 - for interview data

wStep2 - for physical measures
wStep3 - for biochemical measures
This allows for differences in the weight calculation for each Step of the survey as the age-sex composition of the respondents to each Step can differ slightly due to refusal or drop out. Additionally, some countries perform subsampling for Step 2 and/or Step 3. When no subsampling is done and response rates do not differ across Steps of the survey, the 3 weight variables will be the same."Age range of participants included: 25 to 69 years
Source: https://extranet.who.int/ncdsmicrodata/index.php/catalog/770/study-description#page=overview&tab=study-desc

Belarus: STEPS 2015
The sampling frame is a collection of data and materials from which are selected for the survey. The optimal sampling frame should be complete, accurate and current. Best of all, the above criteria are met by the results of the population census, which became the basis for constructing the sample for the STEPS study. Census population represents a representative territorial sampling frame in the form a hierarchical set of parcels grouped in a certain way. Plots censuses are, on average, about the same size. For each site there is a schematic map that provides a clear, non-overlapping demarcation of geographic districts, as well as information on the population and the number of households.
The largest in size is the census area, which includes several instructor sites. The smallest unit in the hierarchical structure of parcels by censuses - enumeration areas.A positive aspect of using enumeration areas as primary sampling units (PSUs) is that they have a small and approximately the same size (each includes about 100 HHs on average). Consequently this, the PSU is a territory within which it is possible to effectively organize field work. To conduct a population census, the territory of the Republic of Belarus was divided into almost 32 thousand enumeration areas. Due to the fact that the last population census in the Republic of Belarus was carried out in 2009, to update the sample, the current data of polyclinics were used, medical outpatient clinics, FAPs and rural Soviet accounting in rural areas.
Age range of participants included: 18-69 years
Source: Translated directly from the Belarus STEPS 2016 report. Available at: https://extranet.who.int/ncdsmicrodata/index.php/catalog/100/related_materials

Belize CAMDI 2005-06

In this cross-sectional descriptive study, 10,822 men and women of six Central American populations (urban areas of San José, Costa Rica; Santa Tecla, San Salvador, El Salvador; Villanueva, Guatemala City, Guatemala; Tegucigalpa, Honduras; and Managua, Nicaragua; and the national population of Belize) were surveyed out of a total of 13,138 pre-selected people. Prospective participants were selected by multi stage random sampling stratified into three age groups (20 to39 years, 40 to 64 years, and 65 or more years)
In each population, census segments were the Primary Sampling Units (PSU). A list of the census segments and the number of dwellings of each urban area of interest was prepared. Primary sampling units (PSU) were selected systematically with a probability proportional to population size, as measured by the respective number of dwellings. Out of the 6,708 census segments into which the target populations were divided, 212 were selected. Maps of the selected segments were updated because the information obtained was outdated.
Once the map of the selected census segments was updated, it was divided into compact segments (CS), or groups of 11 to 12 dwellings. Two CSs of each segment where then randomly selected. In each country approximately 100 CSs were selected. A census was taken of the dwellings in all the CSs. All individuals in the CSs who met the selection criteria were included in the sample, independent of whether or not the dwelling was inhabited by more than one family. For the purposes of this study, a family was defined as a group of cohabitants who eat at the same table. All family members were visited to be informed about the survey and the importance of participating. Those who agreed to participate signed a consent form after having read it with the interviewer. No substitutions of any sector, sub-unit, dwelling, or interviewee were allowed.
Age range of participants included: ≥20 years
Source: https://www.paho.org/hq/dmdocuments/2012/PAHO-CAMDI-English2-2012.pdf

Benin: STEPS 2015
"The STEPS survey on risk factors for non-communicable diseases in Benin was conducted from October to December 2015. It was a population-based survey of adults aged 18 to 69 years. A 3-stage sampling frame was used to produce representative data for this age group in Benin. The information required for the investigation was collected electronically using a manual device. The survey was implemented by the National Program for the Fight against Non-Communicable Diseases (PNLMNT) of the Ministry of Health of Benin. A total of 5,126 adults participated in the STEPS survey conducted in Benin. The overall response rate was 98.6%. The 1st survey took place in 2008. A third survey is planned for 2020 if the financial situation allows it."

Age range of participants included: 18-69 years

Source: Translated directly from the Benin STEPS 2015 report. Available at:

https://extranet.who.int/ncdsmicrodata/index.php/catalog/107/download/1044

Botswana: STEPS 2014

Botswana has a population of over 2 million with 27 districts and 4,845 enumeration areas and sample size of 300 enumeration areas with a target population of 6,400 people was systematically drawn from a pool of the whole enumeration areas. Against the identified enumeration areas numbers of households were listed and proportion of participants was calculated from the total sample size required for the country. Finally a computer generated random number was drawn to go into specific households in that specific enumeration area and at the end eligible participants residing in the household were listed into the electronic hand held data assistant (PDA) and at the end a name was picked automatically to participate in the survey.

Age range of participants included: 15-69 years

Source: Botswana STEPS report. Available at:

https://extranet.who.int/ncdsmicrodata/index.php/catalog/318

Burkina Faso: STEPS 2013

"Sampling methodology: The study was conducted on a sample obtained from a three-stage cluster stratified as recommended by the WHO for STEPS screening surveys. risk factors for noncommunicable diseases. The sampling frame used was that derived from the general census of the population and habitat 2006 (RGPH 2006) and updated in 2010 during the survey Demographic and Health Survey of Burkina Faso (EDS-BF, 2010). This update concerned the enumeration areas (EAs) that correspond to the cluster as part of this study.

Selection of clusters: The choice of clusters was made according to a systematic random selection proportional to their size (in number of households) within strata (regions). To do this clusters were organized by stratum and place of residence (urban / rural). A total of 240 clusters of which 185 were in rural areas and 55 in urban areas were selected for the investigation.

Selection of households: Households were randomly drawn after an enumeration exhaustive list of all households in the cluster. A draw tool designed on Excel by the team. The technique was used in the field for selecting households to investigate. In total, 20 households in clusters were selected to participate in the study.

Selection of individuals: The choice of individuals was made randomly using Kish's method. In total, an individual aged 25 to 64 living in a selected household was fired for participate in the survey."
Age range of participants included: 25-64 years
Source, translated from: Rapport de l'enquete national sur la prevalence des principaux facteurs de risques communs aux maladies non transmissibles au Burkina Faso Enquete STEPS 2013. Available at: http://www.who.int/chp/steps/burkina_faso/en/.

Cabo Verde STEPS 2007

A multi-stage cluster sample of households. One individual within the age range of the survey was selected per household. Analysis weights were calculated by taking the inverse of the probability of selection of each participant. These weights were adjusted for differences in the age-sex composition of the sample population as compared to the target population.
Different weight variables are available per Step:
wStep1 - for interview data
wStep2 - for physical measures
wStep3 - for biochemical measures
This allows for differences in the weight calculation for each Step of the survey as the age-sex composition of the respondents to each Step can differ slightly due to refusal or drop out. Additionally, some countries perform subsampling for Step 2 and/or Step 3. When no subsampling is done and response rates do not differ across Steps of the survey, the 3 weight variables will be the same.
Age range of participants included: 25-64 years
Source: No report available. Sampling information obtained from: https://extranet.who.int/ncdsmicrodata/index.php/catalog/319/related_materials

Cambodia: STEPS 2010

"The initial planned sample size was designed to involve 5,760 persons in accordance with the NCD multi-stage cluster survey method (1.5 design effect, 95% confidence interval, 5% margin or error, and 50% baseline levels of the indicators) in order to provide an equivalent distribution of the participants in regards to age groups and gender after taking into consideration that the estimated potential rate for non-response in each group and refusals in the nest stages would equal to 20%. Estimates were obtained for each of the following eight age/sex groups: men ahed 25-34 years, 35-

44 years, 45-54 years, and 55-64 years; and women aged 25-34 years, 35-44 years, 45-54 years, and 55-64 years.

The survey was designed to cover all geographical areas of Cambodia and a 3-stage sampling process as part of the multi-stage cluster sampling was carried out to randomly select the target population: random selection of communes (Khum in rural areas and its equivalent Sangkat in urban area) as primary sampling unit (PSU), followed by villages (Phum) for the second sampling unit (SSU), and by households for the elementary units (EU). Finally, all members of the randomly chose households aged 25-64 years were invited to participate in this survey. The selection process was performed identically for urban and rural areas in order to get a self-weighted estimate for the whole population of the country. A total of 180 clusters with 34 clusters from the urban area and 146 clusters from the rural area were randomly selected."

Age range of participants included: 25-64 years

Source: Cambodia STEPS 2010 survey report. Available at: https://www.who.int/ncds/surveillance/steps/cambodia/en/

Chile: NHS 2009-10

"The sampling frame was constituted from the Population and Housing Census 2002. The design of the study was transversal, with a random sample of complex type households (stratified and multi-stage by clusters) with national, regional and area representation rural / urban. The target population was adults older than or equal to 15 years. The survey had a response rate in the eligible population of 85%. The refusal rate was of 12%. 5,434 people were interviewed. A nurse performed clinical and examinations to 5,043 participants and 4,956 accepted laboratory tests (blood and urine). The total sample loss of the oversized sample was 28% (this including rejection, non-contact and other causes of random loss). The raw sample was designed with overrepresentation of some population groups (older adults, regions other than the Metropolitan Region and rural areas) to increase sample efficiency and homogenize the accuracy of the estimators. The expansion of the sample data is because it grants each participant the weight that corresponds to it according to the design sample and at the same time corrects the distortion of the raw sample, making it coincide with the census population projection for January 2010 for Chilean adults over 15 years of age"

Age range of participants included: 15 years or older

Source, translated from: Resumen Ejecutivo: Encuesta Nacional de Salud ENS Chile 2009-10. Available at: http://epi.minsal.cl/encuesta-ens-anteriores/.

China: CHNS 2009

"The China Health and Nutrition Survey is a longitudinal study across 228 communities within nine provinces of China. Surveys began in 1989, with subsequent surveys every 2–4 years, for a total of nine rounds between 1989 and 2011. The China Health and Nutrition Survey was designed to provide representation of rural, urban and suburban areas varying substantially in geography, economic development, public resources and health indicators,[13] and it is the only large-scale, longitudinal study of its kind in China. The original survey in 1989 used a multistage, random cluster design in eight provinces (Liaoning, Jiangsu, Shandong, Henan, Hubei, Hunan, Guangxi and Guizhou) to select a stratified probability sample; a ninth province, Heilongjiang, was added in 1997 using a similar sampling strategy. Essentially, two cities (one large and one small city—usually the provincial capital and a lower income city) and four counties (stratified by income: one high, one low and two middle income counties) were selected in each province. Within cities, two urban and two suburban communities were selected; within counties, one community in the capital city and three rural villages were chosen. Twenty households per community were then selected for participation. The study met the standards for the ethical treatment of participants and was approved by the Institutional Review Boards of the University of North Carolina at Chapel Hill and the Institute of Nutrition and Food Safety, Chinese Center for Disease Control and Prevention."

Age range of participants included: all ages

Source: Attard, Samantha M.; Herring, Amy H.; Wang, Huiling; Howard, Annie Green; Thompson, Amanda L.; Adair, Linda S.; Mayer-Davis, Elizabeth J.; & Gordon-Larsen, Penny. (2015). Implications of Iron Deficiency/Anemia on the Classification of Diabetes Using HbA1c. Nutrition & Diabetes, 5, e166.

Comoros: STEPS 2011

"The STEPS survey on risk factors for chronic diseases in the Union of the Comoros took place from January to March 2011. This study has undertaken Step 1, Step 2 and Step 3. Indeed, socio-demographic and behavioral measures were collected in Step 1. Physical measures such as height, weight and tension were collected in Step 2 and biochemical measurements were collected to assess the levels of blood glucose and cholesterol levels in Step 3. The STEPS survey conducted in Comoros Union is a survey of general population, targeting adults aged 25 to 64 years. A stratified survey was used to produce representative data for this age group. A total of 5556 adults aged 25 to 64 participated in the STEPS survey on a sample of 5760 people representing an overall response rate of 96.5%."

Age range of participants included: 25-64 years

Source, translated from Union des Comores STEPS 2011 Note de synthèse.

Available at: http://www.who.int/chp/steps/comoros/en/.

Costa Rica: STEPS 2010

"The Costa Rican NCRFSS survey was a cross-sectional survey based on a probabilistic cluster sampling design. The NCRFSS survey was conducted during 2010 under the supervision of the Caja Costarricense de Seguro Social, a government public healthcare provider, and covers the overall adult population aged ≥20 years. Multistage cluster sampling was performed stratified by geographical areas, age groups (20–39, 40–64, and ≥65 years) and gender. The first sample stage was the randomized selection of the country's geographical areas as primary sample units followed by the random selection of sectors in selected areas as secondary sample units. The random selection of areas and sectors was performed with probability proportional to size; the area or sector size was determined by the population >20 years during 2009, as estimated by the Costa Rican Census and Statistics National Institute (INEC). Households were chosen through a random number generator using dwelling lists obtained from the health technician assistant in every community until all age group and gender strata sample sizes were achieved. A family dwelling was defined as a group of people who share the same table to eat. Survey participants were selected by the Kish method, which samples participants within a household with equal probability of selection, as recommended by the WHO STEPwise methodology. To be eligible for inclusion in the study, subjects had to be ≥20 years of age, permanently residing in the selected homes, and to have provided written consent. Pregnant or lactating mothers and those who were within 6 months postpartum were excluded from the study. Each participant selected for the study was informed of the study objectives and details before agreeing to participate in the investigation. In all, 3653 noninstitutionalized adults were surveyed, with an 87.8% response rate of the eligible population."

Age range of participants included: 20 years or older

Source: Wong-McClure R, Gregg EW, Barcelo A, Sanabria-Lopez L, Lee K, Abarca-Gomez L, Cervantes-Loaiza M, Luman ET. Prevalence of diabetes and impaired fasting glucose in Costa Rica: Costa Rican National Cardiovascular Risk Factors Survey, 2010. J Diabetes. 2016 Sep;8(5):686-92.

Ecuador STEPS 2018

Type and stages of the sample design. The STEPS sample was selected following an element probability sampling scheme with the following three stages of selection: i) first stage: selection of

Primary Sampling Units (PSU) per stratum; ii) second stage: selection of 12 occupied households within each PSU selected in the first stage; and, iii) third stage: selection of 1 person between 18 and 69 years old per household. Study domains. Men and women between 18 and 69 years of age at the national level, with the exception of Galapagos.

Sample selection. The selection of the PSUs, according to the established size, was carried out independently in a random manner in each of the strata. Twelve households were also randomly selected from each previously selected cluster. From the second survey period onwards, given the high rates of occupancy change, 16 dwellings per conglomerate were selected to counteract this effect. The change affected the remaining 230 clusters, giving a total of 6,680 dwellings to be surveyed. Finally, a list was made of the persons eligible for selection within each dwelling, randomly selecting one of them.
Age range of participants included: 18-69 years
Source: Ecuador STEPS 2019 Report [Translated]. Available at: https://extranet.who.int/ncdsmicrodata/index.php/catalog/774/study-description#page=sampling&tab=study-desc

El Salvador 2014-15
The sample selection was carried out in a two-stage and probabilistic manner; the sample framework was the population census conducted in El Salvador in 2007. A cartographic update of the census segments conducted by Digestyc in 2015 was carried out and these were divided into clusters, which were composed of 12 to 25 dwellings and finally to all persons in the dwellings that met the inclusion criteria.

The data collection process was carried out in two stages: in the first stage, each of the selected houses was visited, where all the members of the household who met the inclusion criteria were listed in a family file. The objective of the study was explained to the eligible persons and they were given the consent form to read it; the document was read to those who had difficulty reading and it was explained to them that they could withdraw from the study at any time if they chose to do so. Once the reading was finished, they were invited to participate in the study; those who accepted signed the informed consent form or placed their fingerprint, and then proceeded to conduct the survey.

If a person was ill at the time of the survey or had been diagnosed during the application of the survey, he/she was referred to a health facility. The actual fieldwork was conducted from October 2014 to March 2015. The second measurement was performed with a minimum interval of three months after the first one, in order to confirm the CKD. Thus in January 2015, the remeasurement was carried out, ending in March 2015.Out of a total of 1032 persons to be remeasured, 725 underwent such remeasurement. After the study, 4817 questionnaires that met all the required methodological conditions were completed. These were used to form the database for the analysis of the results. Estimates were made according to sex, 3 age groups (20 to 40, 41 to 60 and 60 and over), urban and rural area of residence and Minsal health regions.

Age range of participants included: ≥20 years

Source: Ministerio de Salud, 2015. Encuesta Nacional de Enfermedades Crónicas no transimibles en Población Adulta de El Salvador. San Salvador.[Translated]

Eritrea: STEPS 2010

"A multi-stage cluster sample of households. One individual within the age range of the survey was selected per household.

Analysis weights were calculated by taking the inverse of the probability of selection of each participant. These weights were adjusted for differences in the age-sex composition of the sample population as compared to the target population.

Different weight variables are available per Step:

wStep1 - for interview data

wStep2 - for physical measures

wStep3 - for biochemical measures

This allows for differences in the weight calculation for each Step of the survey as the age-sex composition of the respondents to each Step can differ slightly due to refusal or drop out.

Additionally, some countries perform subsampling for Step 2 and/or Step 3. When no subsampling is done and response rates do not differ across Steps of the survey, the 3 weight variables will be the same."

Age range of participants included: 25-74 years

Source: no report available. Sampling information obtained from: https://extranet.who.int/ncdsmicrodata/index.php/catalog/589/study-description#page=sampling&tab=study-desc

Eswatini: STEPS 2014

"A Multi-stage cluster sampling design was applied. The survey covered all the four regions of the country. The size of the country and the distances between the regions and communities made it possible for the survey to sample a population representing all the 4 regions. The Multi-stage sampling procedure was implemented in the following procedural steps:

Stage 1: All four regions were included as a sampling frame of our Primary Sampling Unit (PSU).The number of the PSUs at this stage ensured precision in the survey estimates and as a result 216 PSUs were selected using probability proportional to size sampling.

Stage 2: The second stage of cluster sampling procedure entailed listing, sorting and random systematic sampling of the Secondary Sampling Units (Households) within the PSUs selected in stage1 where 20 households were selected from each PSU. Based on census data, only households with eligible participants were systematically sampled through random systematic sampling.

Stage 3: At this level, all the eligible participants within a household were sequentially listed into the PDAs and only one participant per household was randomly sampled using KISH method built into the PDAs. The KISH method is a widely used technique that uses a pre-assigned table of random numbers to identify the person to be interviewed."

Age range of participants included: 15 to 69 years

Source: WHO STEPS: Noncommunicable Disease Risk Factor Surveillance Report Swaziland 2014. Available at: http://www.who.int/chp/steps/swaziland/en/.

Ethiopia STEPS 2015:

According to the WHO step-wise approach to the surveillance of NCD risk factors, a community-based cross sectional study was carried out.

The target population for this survey included all men and women age15-69 years old who have been living at their place of residence for at least six months. This target population included all people who consider Ethiopia to be their primary place of residence. This definition included those individuals residing in Ethiopia regardless of their citizenship status. . People with the following characteristics were not included: those who were not a permanent resident of Ethiopia, and those who were institutionalized including people residing in hospitals, prisons, nursing homes, and other similar institutions or residents whose primary residences are military camps or dormitories. Furthermore, critically ill, mentally disabled and those with some type of physical disability that is not suitable for physical measurement were excluded from this study. In general, the target

population of the study included individuals 15-69 years old and residing in all geographic areas of the country.

Age range of participants included: 15 to 69 years

Source: Ethiopia STEPS 2015 Report. Available at:

https://extranet.who.int/ncdsmicrodata/index.php/catalog/794

Fiji: STEPS 2011

"Sampling Procedure

The survey used a multi cluster stage sample design to produce representative data for adults aged 25-64 years.

Response Rate

A total of 2,586 people participated in the 2011 survey (response rate 2,586/4,850= 53.3%).

Weighting

Analysis weights were calculated by taking the inverse of the probability of selection of each participant. These weights were adjusted for differences in the age-sex composition of the sample population as compared to the target population.

Different weight variables are available per Step:

wStep1 - for interview data

wStep2 - for physical measures

wStep3 - for biochemical measures

This allows for differences in the weight calculation for each Step of the survey as the age-sex composition of the respondents to each Step can differ slightly due to refusal or drop out. Additionally, some countries perform subsampling for Step 2 and/or Step 3. When no subsampling is done and response rates do not differ across Steps of the survey, the 3 weight variables will be the same."

Age range of participants included: 25 to 64 years

Source:

https://extranet.who.int/ncdsmicrodata/index.php/catalog/636/study-description#page=sampling&tab=study-desc

Georgia: STEPS 2016

"The STEPS survey of noncommunicable disease (NCD) risk factors in Georgia was carried out from June 2016 to September 2016. Georgia carried out Step 1, Step 2 and Step 3. Socio demographic and

behavioural information was collected in Step 1. Physical measurements such as height, weight and blood pressure were collected in Step 2. Biochemical measurements were collected to assess blood glucose and cholesterol levels in Step 3. The survey was a population-based survey of adults aged 18-69. A Multi-stage cluster sampling design was used to produce representative data for that age range in Georgia. A total of 5554 adults participated in the survey. The overall response rate was 75.7%."

Age range of participants included: 18 to 69 years

Source: Georgia STEPS Survey 2016 Fact Sheet.

Available at: http://www.who.int/chp/steps/georgia/en/.

Guyana: STEPS 2016

"A response rate of 66.68% will be selected based on the experience and response rates of other surveys over the years such as the recent Demographic Health Survey 2009. [...] STEPS 3 involve taking blood samples from a proportion of the sample, in this case 50% of the sample, in order to measure raised blood glucose levels and abnormal blood lipids. [...] The STEPS sample will be prepared by the Bureau of Statistics Guyana following the recommended STEPS sample methodology. A multi-stage cluster sampling design will be used. Guyana is divided into 10 administrative regions and within the administrative regions there are seven towns and each region is further divided into enumeration districts. For the STEPS survey 288 enumeration districts will be selected using the population probability sampling method and from each enumeration district 12 households will be selected giving a total sample size of 3456. Further at the household level each participant will be randomly selected by the electronic tablet. For STEP 3 50% of the sample will be randomly selected to participate. A re-listing of some households may also be necessary, such as those interior region locations, in which case in addition to household listings, enumeration districts maps will also be provided so that a re-listing can be done where required."

Age range of participants included: 18 to 69 years

Source: STEPwise Approach to Chronic Disease risk factor surveillance (STEPS): Guyana's Implementation Plan. June 20, 2016. Ministry of Public Health, Guyana.

Indonesia: IFLS 2014-15

"Because it is a longitudinal survey, IFLS5 drew its sample from IFLS1, IFLS2, IFLS2+, IFLS3 and IFLS4. The IFLS1 sampling scheme stratified on provinces and urban/rural location, then randomly

sampled within these strata (see Frankenberg and Karoly, 1995, for a detailed description). Provinces were selected to maximize representation of the population, capture the cultural and socioeconomic diversity of Indonesia, and be cost effective to survey given the size and terrain of the country. For mainly cost-effectiveness reasons, 14 of the then existing 27 provinces were excluded.3 The resulting sample included 13 of Indonesia's 27 provinces containing 83% of the population: four provinces on Sumatra (North Sumatra, West Sumatra, South Sumatra, and Lampung), all five of the Javanese provinces (DKI Jakarta, West Java, Central Java, DI Yogyakarta, and East Java), and four provinces covering the remaining major island groups (Bali, West Nusa Tenggara, South Kalimantan, and South Sulawesi).

Within each of the 13 provinces, enumeration areas (EAs) were randomly chosen from a nationally representative sample frame used in the 1993 SUSENAS, a socioeconomic survey of about 60,000 households. The IFLS randomly selected 321 enumeration areas in the 13 provinces, over-sampling urban EAs and EAs in smaller provinces to facilitate urban-rural and Javanese–non-Javanese comparisons.

Within a selected EA, households were randomly selected based upon 1993 SUSENAS listings obtained from regional BPS office. A household was defined as a group of people whose members reside in the same dwelling and share food from the same cooking pot (the standard BPS definition). Twenty households were selected from each urban EA, and 30 households were selected from each rural EA. This strategy minimized expensive travel between rural EAs while balancing the costs of correlations among households. For IFLS1 a total of 7,730 households were sampled to obtain a final sample size goal of 7,000 completed households. This strategy was based on BPS experience of about 90% completion rates. In fact, IFLS1 exceeded that target and interviews were conducted with 7,224 households in late 1993 and early 1994. In IFLS1 it was determined to be too costly to interview all household members, so a sampling scheme was used to randomly select several members within a household to provide detailed individual information."

Age range of participants included: all ages

Source: Strauss, J., F. Witoelar, and B. Sikoki. "The Fifth Wave of the Indonesia Family Life Survey (IFLS5): Overview and Field Report". March 2016. WR-1143/1-NIA/NICHD.

Iran: STEPS 2016

"The sampling part, which includes determining the sample size and the cluster head, belongs to the pre-study phase and was planned in the form of a specific protocol for sample size and statistical

sampling. All experts in the quality control team supervised the finding of samples and cluster heads.

In order to estimate the prevalence rate of the risk factors for non-communicable diseases in the country in 1395, a sampling method proportionate to the population was used, which is a common approach in survey studies. Therefore, the selected sample size was proportionated to the population of that province. On the other hand, for estimating the prevalence of the risk factors in the province, in order to be on the safe side, the smallest sample size for achieving the predicted rates was calculated at 95%. This rate was equal to 384 samples, which was selected as the smallest sample size in the least populated province, Ilam. The required sample size for other provinces was therefore calculated according to the population of that province proportionate to the population of the reference province, Ilam. Besides, to control the non-response error, 10% was added to the calculated sample size in each province. In order to decrease costs and increase efficiency, for provinces with 800 samples or more, weights were given to their samples. Weight-giving is an effective method used in surveys in order to decrease the sample size. This was achieved in the selected provinces by considering the calculated sample size as half and the sampling weight as double. The total sample size was calculated to be 30150 and to achieve this sample size, sampling from 3015 clusters was required."

Age range of participants included: 18 and older

Source: Iran STEPS 2015 report.

Available at: https://www.who.int/ncds/surveillance/steps/STEPS_2016_Atlas_EN.pdf?ua=1

Iraq: STEPS 2015

"The sample frame consisted of the population of Iraq of (18+) years for both sexes residing in the urban and rural area. It was based on the results of listing and numbering operation for the year 2009 that covered all governorates. Due to the unstable conditions at the time of the survey three governorates (Naynawa, Salahaddin and Al-Anbar) were excluded. A major challenge confronted was the late demographic change due to population movement, displacement and migration.All permanent residents of (18+) years of age, who were resident in Iraq within one month at the time of implementation of the survey were considered eligible.

A cross-sectional community based survey covering 15 governorates in Iraq. A Multi-stage cluster sampling technique was depended to select the minimum representative sample size to estimate the prevalence of the risk factors of noncommunicable disease through direct interview, physical examination and laboratory examination of blood samples of study participants. A total of 412

clusters were randomly selected each contain ten households. One subject from each household was randomly selected using KISH table to participate in the survey with a total sample size of 4120. The Sample was designed to provide estimates on a number of indicators on the situation of Noncommunicable diseases risk factors in Iraq at the national level. A national based rather than a governorate based sample is selected. A multi stage cluster sampling was used with stratification to urban and rural areas. Primary sampling units (PSUs) were the blocks, which consisted of 70 households or more before selection."

Age range of participants included: 18 years and older

Source: Iraq STEPS 2015 report.

Available at: https://www.who.int/ncds/surveillance/steps/Iraq_2015_STEPS_Report.pdf

Jordan STEPS 2019

A national cross-sectional survey was conducted adopting a two-stage stratified-cluster sampling design. The margin error was (5%) and the confidence level was set at 95%. The Jordan Population and Housing Census 2015 was used as a sampling frame for Jordanians. A sample of 3000 households was randomly drawn to represent the Jordanian population. It was designed in a probability proportional to size (PPS) way to provide valid and reliable survey estimates across the entire Kingdom of Jordan - rural and urban areas, the twelve governorates and the smaller communities within. The sample also ensured reliable estimates in terms of geographical distribution, where Jordan was divided into three regions; north, centre, and south, also at governorate level. The north of Jordan covered Ajloun, Irbid, Jerash, and Mafraq, the centre region covered Amman, Balqa, Madaba, and Zarqa, and the south region covered Aqaba, Karak, Ma'an, and Tafieleh. Furthermore, each governorate was subdivided into area units called census blocks, which were the Primary Sampling Units (PSU-Blocks) for this survey (on average a PSU comprises 50-70 households). The PSU-Blocks were then regrouped to form clusters. From each PSU, eight households were randomly drawn with an equal probability systematic selection. A household was defined as a group of people living in the same dwelling space who eat meals together, acknowledging the authority of a man or a woman as the head of the household. After the household selection and obtaining the permission of household residents to participate in the survey, all the eligible household members were entered into the STEPS program, which ran a random selection to choose one member household.

Age range of participants included: 18 to 69 years

Source: Jordan STEPS 2019 Report. Available at: https://extranet.who.int/ncdsmicrodata/index.php/catalog/853

Kenya: STEPS 2015

"The 2015 Kenya STEPs survey was a national cross-sectional household survey designed to provide estimates for indicators on risk factors for non-communicable diseases for persons age 18 – 69 years. The sample was designed with a sample size of 6,000 individuals to allow national estimates by sex (male and female) and residence (urban and rural areas). The survey used the fifth National Sample Surveys and Evaluation Programme (NASSEP V) master sample frame that was developed and maintained by KNBS. The frame was developed using the Enumeration Areas (EAs) generated from the 2009 Kenya Population and Housing Census to form 5,360 clusters split into four equal sub-samples. A three-stage cluster sample design was adopted for the survey involving selection of clusters, households and eligible individuals. In the first stage, 200 clusters (100 urban and 100 rural) were selected from one sub-sample of NASSEP V frame. A uniform sample of 30 households from the listed households in each cluster was selected in the second stage of sampling. The last stage of sampling was done using Personal Digital Assistants (PDAs) at the time of survey, where one individual was randomly selected from all eligible listed household members using a programmed KISH method of sampling."

Age range of participants included: 18 to 69 years

Source: WHO: Kenya STEPwise Survey for Non Communicable Diseases Risk Factors 2015 Report. Available at: http://www.who.int/chp/steps/Kenya_2015_STEPS_Report.pdf?ua=1.

Kiribati: STEPS 2015

The second Kiribati STEPS Survey was a population-based survey of 18-69 year olds. The decision was to use three age groups: 18-29, 30-44, 45-69 years for men and women using the following corrections:

- Design Effect of 1.0 (clustering at village and household level)
- 95% confidence interval; p value .05
- 0.7% response rate
- Baseline prevalence percentage indicator: 0.5
- FPC – not applicable
- 6 age-sex groups (18-29 years, 30-44 years, 45-69 years)

As STEPS is intended to be nationally representative, a multi-stage cluster sampling method was used. The STEPS sampling spreadsheet was completed using the most recent census information (2012). The sample was selected in two stages assuming no replacement. At the first stage, a sample of Enumeration Areas (Islands and villages) from each stratum using probability proportional to size (PPS) sampling was selected. In the second stage, a fixed number of households from each selected Enumeration Area using systematic sampling was se-lected. The third stage of sampling selection was done at the household level using the KISH method.

The sampling identified that data collection would be needed on the following islands: Makin, Butaritari, Mara-kei, Abaiang, North Tarawa, South Tarawa,Betio, Maiana, Abemama, Kuria, Aranuka, Nonouti, Tabiteuea North, Tabiteuea South, Arorae, Tabuaeran and Kiritimati. Further details in Annex 3."

Age range of participants included: 18 to 69 years

Source: Kiribati STEPS 2015 report. Available at: https://extranet.who.int/ncdsmicrodata/index.php/catalog/724

Kyrgyzstan: STEPS 2013

A multi-stage cluster sample of households. One individual within the age range of the survey was selected per household.

Analysis weights were calculated by taking the inverse of the probability of selection of each participant. These weights were adjusted for differences in the age-sex composition of the sample population as compared to the target population.

Different weight variables are available per Step:

wStep1 - for interview data

wStep2 - for physical measures

wStep3 - for biochemical measures

This allows for differences in the weight calculation for each Step of the survey as the age-sex composition of the respondents to each Step can differ slightly due to refusal or drop out.

Age range of participants included: 25 to 64 years

Source: no report or fact sheet available. Sampling information obtained from:https://extranet.who.int/ncdsmicrodata/index.php/catalog/271/study-description#page=overview&tab=study-desc

Lao People's Democratic Republic: STEPS 2013

A multi-stage cluster sample of households. One individual within the age range of the survey was selected per household. Analysis weights were calculated by taking the inverse of the probability of selection of each participant. These weights were adjusted for differences in the age-sex composition of the sample population as compared to the target population.

Different weight variables are available per Step:

wStep1 - for interview data

wStep2 - for physical measures

wStep3 - for biochemical measures

This allows for differences in the weight calculation for each Step of the survey as the age-sex composition of the respondents to each Step can differ slightly due to refusal or drop out. Additionally, some countries perform subsampling for Step 2 and/or Step 3. When no subsampling is done and response rates do not differ across Steps of the survey, the 3 weight variables will be the same.

Age range of participants included: 18 to 64 years

Source: no report or fact sheet available. Sampling information obtained from: https://extranet.who.int/ncdsmicrodata/index.php/catalog/588/study-description#page=sampling&tab=study-desc

Lebanon: STEPS 2017

"A national cross-sectional survey adopting a two-stage cluster sampling design was conducted for Steps 1, 2 and 3. The sampling frames references used were the population distribution in Lebanon 2014, retrieved from the Central Administration for Statistics (CAS) and the Syrian population distribution data 2015, retrieved from UNHCR. 144 clusters were selected for the Lebanese sample and 144 clusters for the Syrian sample. The Primary Sampling Units (PSUs) were cadastral areas (cadasters) and the Secondary Sampling Units (SSUs) were the households. Twenty participants were recruited from each cluster. The latest available population estimates (cadastral data) were used, to randomly recruit PSUs by Probability Proportionate to Size (PPS). To account for the issue of the variability in the cadasters' sizes, very small cadasters (<200 individuals) were combined with neighboring PSUs before selecting the sample, to enhance the likelihood of finding 20 target participants. On the other hand, cadasters with a large population size that were guaranteed to be sampled at least twice were handled as strata and each stratum were assigned a fixed number of random starting points based on how often it was selected with certainty. This was done using

satellite images divided into grids, previously obtained from the Centers for Disease Control and Prevention (CDC) for all Lebanese cadasters.

For the Lebanese sample, the research team relied on the standard Expanded Program for Immunization (EPI) method for a systematic random selection of the households. Accordingly, within each selected PSU, households were identified using a systematic random approach following the WHO-UNICEF-EPI cluster method. The fieldworkers started with the highest floor on the right side of a building. If the household hosted an eligible participant, they proceeded with data collection, if not, they visited a second household which is selected by skipping 5 households. If during sampling, non-Lebanese households were selected, the fieldworker skipped them in a straight line until a Lebanese household was identified. This method has been previously used for national surveys in Lebanon. One participant was randomly selected within each household, using the eSTEPS application. Households were chosen until the target of 20 participants was reached.

The PSUs for the Syrian refugees' sample were identified, using the most recent available refugee estimates to randomly recruit PSUs by PPS. The same measures aforementioned were done to account for the variation in the cadasters' sizes. The WHO-UNICEF- EPI cluster method was employed to select households. The fieldworkers targeted Syrian households; accordingly, when during sampling, non-Syrian households were selected, the fieldworker skipped them in a straight line until a Syrian household was identified. One participant was randomly selected within each household, using the eSTEPS application.

For both samples, following STEPS' team recommendations, sampling of participants was done without replacement, i.e. once a person was selected that person was not replaced with another one. Efforts were made to include all selected households. If the house was unoccupied at the time of the visit or if an adult was not available for an interview at the time of the visit, that house was revisited up to 4 times, with different visiting times. The number of refusals and non-responses was recorded."
Age range of participants included: 18 to 69 years
Source: Lebanon STEPS 2016-2017 report. Available at:
https://www.who.int/ncds/surveillance/steps/Lebanon_STEPS_report_2016-2017.pdf?ua=1

Lesotho: STEPS 2012

A multi-stage cluster sample of households. One individual within the age range of the survey was selected per household. Analysis weights were calculated by taking the inverse of the probability of selection of each participant. These weights were adjusted for differences in the age-sex composition of the sample population as compared to the target population.
Different weight variables are available per Step:
wStep1 - for interview data
wStep2 - for physical measures
wStep3 - for biochemical measures
This allows for differences in the weight calculation for each Step of the survey as the age-sex composition of the respondents to each Step can differ slightly due to refusal or drop out. Additionally, some countries perform subsampling for Step 2 and/or Step 3. When no subsampling is done and response rates do not differ across Steps of the survey, the 3 weight variables will be the same.
Age range of participants included: 25-64 years
Source: Source: no report available. Sampling information obtained from: https://extranet.who.int/ncdsmicrodata/index.php/catalog/491/study-description#page=sampling&tab=study-desc

Liberia: STEPS 2011
"Random multi-cluster sampling method was used to collect data during this survey in 5 of the 15 counties of Liberia with the district serving as the primary sampling unit. Different sampling frames were designed and used at the district (Primary Sampling Unit-PSU), Chiefdoms (Secondary Sampling Unit-SSU) and household levels. Households listing generated from the 2008 National Population Census was used, and in each household, the list of individuals' resident was obtained and the Kish Method was used. Kish Method is a household sampling technique developed by WHO for STEPS. The field team selected households by using nutrition sampling method (throwing a pencil to get a selected direction). When the household enumeration sampling point is established, the interviewer counts all the households and using interval sample to get the household number. In each household, one person was selected using the Kish method."
Age range of participants included: 25 to 64 years
Source: WHO: The Final Report on the Liberia STEPS Survey 2011. Available at: http://www.who.int/chp/steps/Liberia_2011_STEPS_Report.pdf?ua=1.

Libya STEPS 2009

The STEPS survey of chronic disease risk factors in Libya was carried out from Feb 2009 to Nov 2009. Libya carried out Step 1, Step 2 and Step 3. Socio demographic and behavioural information was collected in Step 1. Physical measurements such as height, weight and blood pressure were collected in Step 2. Biochemical measurements were collected to assess blood glucose and cholesterol levels in Step 3. The STEPS survey in Libya was a population-based survey of adults aged 25-64. A multi-stage cluster sample design was used to produce representative data for that age range in Libya. A total of 3,590 adults participated in the Libya STEPS survey

Age range of participants included: 25 to 64 years

Source: Libya STEPS 2009 Survey Fact Sheet. Available at:

https://extranet.who.int/ncdsmicrodata/index.php/catalog/248

Malawi STEPS 2009

This was a national community based cross-sectional survey, using WHO STEPwise approach for assessing risk factors for chronic non-communicable diseases. The approach includes the use of a questionnaire for gathering demographic and behavioural information (Step 1), then moving to physical measurements (Step 2) and then biochemistry tests (Step 3). In addition, there are three modules of risk factor assessment, namely core, expanded and optional. The STEPS Survey instrument was adapted and tested by the core team and data collectors.

Age range of participants included: 18 to 69 years

Source: Malawi Steps 2009 Report. Available at:

https://extranet.who.int/ncdsmicrodata/index.php/catalog/629

Marshall Islands Hybrid Survey 2017

Stage 1: Households were identified at random according to geographical stratification in Majuro and Ebeye. The country was stratified into two major groups, Urban (Majuro and Ebeye) and Rural (all outer islands). In Majuro and Ebeye, household cluster sampling was used to randomly select households in these areas. Stage 2: In Majuro and Ebeye, one individual was selected at random from each household using the KISH table method. All adults in Kili, Arno, Wotje, and Jabwor, Jaluit atolls were included in the sample because the adult populations are about 200 each on these atolls.

Age range of participants included: ≥18 years

Source Marshall Islands STEPS 2017 Report. Available at:https://extranet.who.int/ncdsmicrodata/index.php/catalog/742/study-description#page=sampling&tab=study-desc

Mexico: ENSANUT 2018

The ENSANUT 2018-19 is a national, urban and rural probabilistic survey. The units of analysis defined for the survey are the following: - Household is the set of people related by some kinship or not who usually sleep in a dwelling under the same roof, benefiting from a common income contributed by one or more of the household members. - Population aged 0 to 4 years (preschoolers)- Population aged 5 to 9 years (schoolchildren)- Population aged 10 to 19 years (adolescents)- Population aged 20 years and older (adults)- Utilizers

Once the PSUs and strata were constructed, the PSUs for the 2018-19 ENSANUT were selected in two stages: first, INEGI selected a master sample of PSUs with probability proportional to their number of dwellings in the year 2012, then, for the 2018-19 ENSANUT, a subsample of PSUs with equal probability was selected within each stratum. Finally, in each PSU, dwellings were selected with equal probability; on average, five dwellings were selected in each PSU of the high urban stratum and 20 dwellings were selected in the PSUs of the rural and urban complement strata.

Whenever possible, one adult, one adolescent, one schoolchild and one preschooler were selected from each household with equal probability. Also, whenever possible, up to two users of medical services during the last 15 days were selected in 40% of the dwellings, and in the remaining 60% of the dwellings, up to one user was selected.

Age range of participants included: All ages

Source: ENSANUT Report. Available at: https://ensanut.insp.mx/encuestas/ensanut2018/informes.php [Translated]

Moldova: STEPS 2013

"A total of 4807 randomly selected respondents participated in the survey. They were all aged 18–69 years, and the group comprised both sexes, as well as residents of all districts and the territorial administrative unit "Gagauz-Yeri", along with Chişinău and Balti municipalities. The survey did not cover the districts from the left bank of the Nistru River and the municipality of Bender. A two-stage

cluster sampling procedure was carried out to select randomly participants from among the target population. Cluster sectors from the 2004 Moldova Population Census were used as a basic unit. Given the differences in lifestyle and disease status between populations in urban and rural areas, the target population was stratified into urban and rural areas of residence for the STEPS survey. At the first stage, within each stratum, primary sampling units (PSUs) (enumeration areas (EAs)) were selected systematically with probability proportional to the 2004 Population Census EAs (measure of size equal to the number of population in the EAs, provided by the census). Before selection, the census sectors were sorted geographically from north to south within each stratum, in order to ensure additional implicit stratification according to geographical criteria. A total of 400 clusters representing 400 EAs were selected from the 10 991 census EAs. These probabilistically selected clusters were used also in Moldova's DHS conducted in 2005, and the Multiple Indicator Cluster Surveys (MICS) conducted in 2012. Cartographic materials from the Population Census conducted in Moldova in 2004 were not available, thus it was not possible to use them for the STEPS survey. Therefore, for the first stage the probabilistic samples from the abovementioned surveys were used.

Out of the 400 selected clusters, 167 were rural and 233 were urban. The distribution of the sample of 400 PSUs (EAs) for the DHS/MICS surveys was inversely proportional to the number of population within each stratum, taking into account that the response rate is lower in urban areas than rural owing to the smaller average size of the households in urban areas compared with rural areas. Thus, disproportional allocation with oversampling for urban areas was applied in the STEPS survey. A final weighting adjustment procedure was carried out to enable estimates at national and urban/rural levels.

At the second stage, 15 households (secondary sampling units (SSUs)) were selected within each of the 400 PSUs. From the updated list of households used for the MICS 2012 survey, 15 households were selected randomly per cluster, using the Microsoft Excel® random sample tool. A total of 6000 individuals were selected from among the 400 clusters. The Kish method *(17)* was applied for the random selection of one individual aged 18–69 years from each household.
Age of participants included: 18-69 years
Source: Republic of Moldova STEPS 2013 report. Available at: https://www.who.int/ncds/surveillance/steps/Moldova_2013_STEPS_Report.pdf

Mongolia: STEPS 2019

A multistage stratified sampling design was used to produce representative data for that age range in Mongolia. A total of 6654 adults participated in the survey. Analysis weights were calculated by taking the inverse of the probability of selection of each participant. These weights were adjusted for differences in the age-sex composition of the sample population as compared to the target population.

Different weight variables are available per Step:

wStep1 - for interview data

wStep2 - for physical measures

wStep3 - for biochemical measures

This allows for differences in the weight calculation for each Step of the survey as the age-sex composition of the respondents to each Step can differ slightly due to refusal or drop out. Additionally, some countries perform subsampling for Step 2 and/or Step 3. When no subsampling is done and response rates do not differ across Steps of the survey, the 3 weight variables will be the same.

Source: No report available. Sampling information obtained from https://extranet.who.int/ncdsmicrodata/index.php/catalog/836/study-description#page=sampling&tab=study-desc

Morocco: STEPS 2017

One of the essential elements for establishing a probability sampling plan is the constitution an adequate sampling frame. For the purpose of the STEPS survey, the sampling frame used to meet the sampling need was the 2014 master sample, developed by the HCP based on data from the 2014 population and housing census. It has the advantage extrapolate the sample results to the target population and estimate the accuracy desired. The stratification of observation units belonging to any sampling frame makes it possible to design sampling plans ensuring optimal sample size; a significant reduction in costs and a substantial improvement in the accuracy of expected estimators. However, the choice of criteria allowing the population to be divided into homogeneous groups (strata) and having recent and reliable data on these criteria is a task that requires generally considerable efforts (updating the sampling frame) both in terms of methodological than that of data collection.

In Morocco, the particularity of cities containing several social categories for which, synthesizing the vector of heterogeneous demographic and socioeconomic behavior into a representative characteristic makes stratification a difficult task. The stratification adopted was geographical for the two environments according to the weight in terms of households, each of which has a specific stratification: For urban units, the criteria used were the administrative division into regions, provinces / prefectures and the dominant habitat type. As for the rural environment, the primary units were stratified according to the geographical criterion, and the type of relief dominant at the municipal level.
Age range of participants included: 18 years and older
Source: Morocco STEPS report [translated online]:
https://extranet.who.int/ncdsmicrodata/index.php/catalog/544/study-description

Myanmar: STEPS 2014
To achieve a nationally representative sample, a multi-stage sampling method was used to select townships, wards and villages, households and eligible participants at each of the selected households.
Stage 1: Selection of primary sampling units (PSUs)
Administratively, Myanmar is divided into 330 townships. A township is subdivided into wards for urban settings and village tracts and then villages for rural settings. The list of townships has been used as the sampling frame at the first stage of sampling. Townships form the Primary Sampling Units (PSUs). Out of the total 330 PSUs, 52 PSUs were selected using Probability Proportionate to Size of population in each PSU (PPS).
Stage 2: Selection of Secondary Sampling Units (SSUs)
From each selected PSU (township), 6 SSUs (wards and villages) were chosen using probability proportionate to population size, totaling 312 SSUs for the whole country.
Stage 3: Selection of eligible participants at household level
From each selected SSU (ward/village), 30 households were selected using systematic random sampling. The sampling frame for this sampling is the list of households with unique identification number (ID) developed from a recent listing of households available from the Basic Health Staff.
Stage 4: Selection of eligible participants at household level
One eligible participant (aged between 25 and 64 years) in the selected households was recruited for the survey. The Kish sampling method was used to randomly select one eligible member of the household. Using the Kish Method, eligible participants (adults aged 25

to 64 years) in each household were ranked in order of 8 decreasing age, starting with males then females, then randomly selected using the automated program for Kish selection in the handheld PDA. Each PSU (township) was estimated to contribute 180 participants, totaling **9,360**participants for 52 selected townships for the whole country. In actual study, the total sample size was 8757 participants.
Age range of participants included: 18 years and older
Source: STEPwise approach to chronic disease risk factor surveillance report 2014. Available at: https://www.who.int/ncds/surveillance/steps/myanmar/en/

Naura STEPS 2015
As STEPS is intended to be nationally representative, a simple random sample of individuals was identified, based on the most recent census survey. As STEPS is intended to be nationally representative, a simple random sample of individuals was identified, based on the most recent census survey.
Source: No report available. Sampling information obtained from https://extranet.who.int/ncdsmicrodata/index.php/catalog/836/study-description#page=sampling&tab=study-desc

Nepal: STEPS 2019
STEPS-2019 is national cross-sectional population-based household survey that used multi-stage cluster sampling design to sample households and eligible adult men and women (15-69 years of age) for questionnaire interview and physical examination (anthropometry, blood pressure measurement, blood glucose and cholesterol and urine sample for salt).
Survey population included men and women aged 15-69 years who have been the usual residents of the household for at least six months and have stayed in the household the night before the survey. People with the follow characteristics were not included: Those whose primary place of residence was in military base or group quarters, Those residing in hospitals, prisons, nursing homes and other institutions, Those too frail and mentally unfit to participate in the study, Those with any physical disability, Those unable or unwilling to give informed consent.
Sampling of Primary units (clusters):
This national representative sample was selected through multistage cluster sampling. Sampling frame consisting of the distribution of oldwards as in census 2011 was obtained from Central Bureau of Statistics (CBS). Then, in each of the province, the oldwards were compared with current

classification of metropolitan, sub metropolitan, municipality, and rural municipalities and recorded as per new classification which has been recently updated by the government of Nepal. The location of the new classifications were matched with the oldwards and, finally, used as the sampling frame for selecting Primary Sampling Units (PSUs) for 2019 STEPS survey.

As a trade-off between survey costs and reducing the standard error, it was decided to sample 25 survey participants from each cluster, requiring sampling of 36.12 ~37 clusters in each of 7 provinces i.e. 259 clusters at national level.

Within each Province, the numbers of clusters were assigned to the three sub-strata in metropolitan, sub-metropolitan, municipality and rural municipality in proportion to the share of population in each of these 3 substrata in the total Province population.

Sampling of households and individuals from clusters:

A total of 25 households were sampled from each of the cluster. A sampling frame of the all households in the sampled PSUs was obtained through a complete household listing and mapping carried out in the sampled PSUs in September 6 to December 6 2018.

Sampling frame for selection of households from each PSU was prepared by conducting household listing and mapping. The team of enumerators visited the sampling PSUs and carried out a complete mapping of all the households in the PSU. If the sampled cluster were large, (if the population exceeds 300), cluster was segmented. In that case, field team started from northeast corner of each PSU and prepared an enumeration area of 300 household's with at least one person aged 15 years or more. Household listing questionnaire was used to list all of the household's members in selected PSUs. The listing was carried out electronically using Android ODK software. Mapping was done along with household listing. Drawing a location map of the cluster as well a detailed sketch map of all structures residing in the cluster was done These materials guided the interviewers to return to the pre-selected households for interview.

This lists of the households so prepared from all sampled PSUs served as the sampling frame for the selection of households in the next stage. From the prepare list, 25 households per PSU were sampled using equal systematic random sampling after determining the sampling interval by dividing the number of listed household by 25 and by randomly selecting the starting number between 0 and the sampling interval. From each of the selected, one adult member was sampled randomly for participation in the survey using the android tablet.

Age range of participants included: 15 to 69 years

Source: Nepal STEPS 2019 Report. Available at: https://extranet.who.int/ncdsmicrodata/index.php/catalog/771

Niger STEPS 2007

The STEPS survey of chronic disease risk factors in Niger was carried out from the 13th to the 27th of December, 2007 in all the regions except Bilma. Niger carried out Step 1, Step 2 and Step 3. Socio demographic and behavioural information was collected in Step 1. Physical measurements such as height, weight and blood pressure were collected in Step 2. Biochemical measurements were collected to assess blood glucose levels in Step 3. The STEPS survey in Niger was a population-based survey of adults aged 15-64. A multi-stage cluster sample design was used to produce representative data for that age range in Niger. A total of 2,760 adults participated in the Niger STEPS survey. The overall response rate was 91.3%

Age range of participants included: 15to 64 years

Source: Niger STEPS Survey 2007. Available at:https://extranet.who.int/ncdsmicrodata/index.php/catalog/736/related_materials

Romania: SEPHAR II

"Sampling was performed by a multi-stratified procedure, leading to the selection of a representative sample of 1942 adults. Subject selection followed the principle of equality of chances of being enrolled in the study, regardless of the size of the place of residency.

Stratification criteria for sample selection were:

- territorial regions (Romania's territory was divided into 7 regions plus the capital city Bucharest, based on the National Statistics Institute recommendations: the North-East region, the South-East region, the South region, the South-West region, the West region, the North-West region, the Central region and the Bucharest region);
- locality type (cities with over 200 000 inhabitants, cities with 50 000–200 000 inhabitants, cities with less than 50 000 inhabitants, Commune);
- gender (male and female);
- age groups (18–24 years, 25–34 years, 35–44 years, 45–54 years, 55–64 years, 65–80 years).

In the first stage of selection, the adult population weighted average was calculated for each region and each district, and, based on this, the number of adult persons from each region/district was calculated from the working sample of 1942 subjects.

In the second stage of selection, the number of localities of a certain size from which the subjects were later selected was established for each district. This number was directly proportional to the population in the respective district. A random selection of a certain locality in a certain category was done using a computer software (generation of random numbers). The selected localities represent the interview centers where the study was to take place. The weighted average of the specific locality population in the district was calculated, and, based on this, the number of people selected to participate in the study.

The third stage of selection consisted of distribution by gender of adult people selected from each locality, using Romania's population gender distribution according to the 2002 census (F : M = 51.25% vs. 48.75%) and the fourth stage of selection consisted of distribution by age of male and female adult people selected from each locality, using Romania's population age distribution according to the 2002 census."

Age range of participants included: 18 to 80 years

Source: Dorobantu M, Tautu OF, Darabont R, Ghiorghe S, Badila E, Dana M, Dobreanu M, Baila I, Rutkowski M, Zdrojewski T. Objectives and methodology of Romanian SEPHAR II Survey. Project for comparing the prevalence and control of cardiovascular risk factors in two East-European countries: Romania and Poland. Arch Med Sci. 2015 Aug 12;11(4):715-23.

Additional reference: Dorobantu M, Tautu O-F, Dimulescu D, Sinescu C, Gusbeth-Tatomir P, Arsenescu-Georgescu C, et al. Perspectives on hypertension's prevalence, treatment and control in a high cardiovascular risk East European country: data from the SEPHAR III survey. J Hypertens. 2018;36(3):690–700.

Rwanda: STEPS 2012-2013

Participants were Rwandan residents aged 15-64 years. Because it was not feasible to conduct a census on the whole population, a representative random sample of participants was selected. To detect statistically significant differences between categories, the WHO STEPwise methodology suggests a minimum sample of 384 people for every age, sex rural/urban or province category the results will be stratified by. For the Rwandan survey the MOH was interested in looking at both males and females across five age groups (15-24 years, 25-34 years, 35-44 years, 45-54 years and 55-64 years), yielding a minimum required sample size of 3840. This was multiplied by 1.5 to account conservatively for the likelihood of a selected participant having the risk factor of interest

and then divided by 0.80 assuming that only 80% of those invited to participate would actually participate. This yielded a required sample size of 7200 participants.

Multistage cluster sampling was used to select these participants from the population based on information from the last census. The three levels of clustering were: 1. Random selection of a statistical enumeration area (as defined by NISR) 2. Random selection of a household within the enumeration area 3. Random selection of an individual within the household.

Administratively, Rwanda is divided into thirty districts. In turn, each district is subdivided into sectors. Each sector is sub-divided into cells and then into villages. Villages are synonymous with enumeration area's (EAs) in Rwanda and there are a total of 14,953 EAs in Rwanda. A total of 180 EA's (or 1.2%) were randomly selected from this total using a probability proportional to size method that gives those EA's with more people living in them a higher chance of being selected. In this way, the representativeness of the selected EAs is maximized.

Age range of participants included: 15-64 years

Source: Republic of Rwanda Non-communicable Diseases Risk Factors Report 2012. Available at: https://extranet.who.int/ncdsmicrodata/index.php/catalog/709

Samoa: STEPS 2013

The STEPS survey of chronic disease risk factors in Samoa was carried out from April 2013 to May 2013. Samoa carried out Step 1, Step 2, and Step 3. Socio demographic and behavioural information was collected in Step 1. Physical measurements such as height, weight and blood pressure were collected in Step 2. Biochemical measurements were collected to assess blood glucose and cholesterol levels in Step 3. The STEPS survey was a population-based survey of adults aged 18-64. A multi-stage, cluster sample design was used to produce representative data for that age range in Samoa. A total of 1766 adults participated in the survey. The overall response rate was 64%. Age range of participants included: 18 to 64 years

Source: Samoa STEPS Survey 2013 Fact Sheet. Available at: https://extranet.who.int/ncdsmicrodata/index.php/catalog/707

Sao Tome and Principe: STEPS 2009

The STEPS survey on risk factors for chronic diseases in São Tomé and Príncipe took place from January to February 2008. São Tomé and Principe has undertaken Step 1, Step 2 and Step 3. Sociodemographic and behavioral data were collected in Step 1. Physical measurements such as height, weight and blood pressure were collected in Step 2. Biochemical measurements were

collected to assess blood sugar and blood sugar levels cholesterol in Step 3. The São Tomé and Principe STEPS survey is a survey of the general population, targeting adults aged 25 to 64. A cluster draw was used to produce representative data for this age group in São Tomé and Principe. A total of 2,457 adults participated in the São Tomé STEPS survey and Principle.
Age range of participants included: 25 to 64 years
Source: Translated from Sao Tome and Principe STEPS 2008 Fact Sheet. Available at: https://extranet.who.int/ncdsmicrodata/index.php/catalog/735

Seychelles: Seychelles Heart Study IV (Seychelles NCD Survey 2013)
"The survey was performed in a sex and age stratified random sample of all adults aged 25-64 years of Seychelles between October and December 2013 on Mahé and during 2 weeks in February 2014 in the islands of Praslin and La Digue. These three islands account for >98% of the total population of Seychelles. The eligible sample was extracted from the population registry. The survey was attended by 1240 adults, with a participation rate of 73%. Participants were invited to attend the survey on selected days in study centers located in Mahé, Praslin, and La Digue. All the eligible participants who did not attend were actively traced using (telephone, local administration, announcements on radio, etc) and invited to attend the survey. Since participants were randomly selected from the general adult population, findings of the survey can be inferred to the general adult population of Seychelles. Blood tests (including glycaemia and A1c) were based on fasting venous plasma blood and analyzed along standard methods in a central laboratory as well as capillary glycaemia and capillary A1c." The survey partly included methods and questions compliant with those used in STEPS surveys."
Age range of participants included: 25 to 64 years

Solomon Islands: STEPS 2015
A multi-stage cluster sample design was used to produce representative data. Analysis weights were calculated by taking the inverse of the probability of selection of each participant. These weights were adjusted for differences in the age-sex composition of the sample population as compared to the target population.
Different weight variables are available per Step:
wStep1 - for interview data
wStep2 - for physical measures
wStep3 - for biochemical measures

This allows for differences in the weight calculation for each Step of the survey as the age-sex composition of the respondents to each Step can differ slightly due to refusal or drop out."
Age range of participants included: 18 to 69 years
Source: no report or fact sheet available. Sampling information obtained from: https://extranet.who.int/ncdsmicrodata/index.php/catalog/710/study-description#page=overview&tab=study-desc

South Africa: SANHANES 2012
"The survey applied a multi-stage disproportionate, stratified cluster sampling approach. A total of 1000 census enumeration areas (EAs) from the 2001 population census were selected from a database of 86,000 EAs and mapped in 2007 using aerial photography to create the 2007 HSRC master sample to use as a basis for sampling of households. The selection of EAs was stratified by province and locality type. In the formal urban areas, race was also used as a third stratification variable (based on the predominant race group in the selected EA at the time of the 2001 census). The allocation of EAs to different stratification categories was disproportionate, in other words, over-sampling or over-allocation of EAs occurred in areas that were dominated by Indian, coloured or white race groups to ensure that the minimum required sample size in those smaller race groups were obtained. Based on the HSRC 2007 Master Sample, 500 Enumerator Areas (EAs) representative of the sociodemographic profile of South Africa were identified and a random sample of 20 visiting points (VPs) were randomly selected from each EA, yielding an overall sample of 10 000 VPs. EAs were sampled with probability proportional to the size of the EA using the 2001 census estimate of the number of VPs in the EA database as a measure of size (MOS). One of the tasks of SANHANES-1 was to recruit and establish a cohort of 5 000 households to be followed up over the coming years. The sampling consisted of: Multi-stage disproportionate, stratified cluster sampling approach; 500 EAs within which 20 VPs/households per EA were sampled; Main reporting domains: sex (male, female), age-group (< 2 years, 2–5 years, 6–14 years, 15–24 years, 25–49 years, 50 years and older), race group (black African, white, coloured, Indian), locality type (urban formal, urban informal, rural formal [including commercial farms] and rural informal], and province (Western Cape, Eastern Cape, Northern Cape, Free State, KwaZulu-Natal, North West, Gauteng, Mpumalanga, Limpopo)."
Age range of participants included: all ages; biomarker information collected on participants 6 years or older

Source: Human Sciences Research Council. SANHANES: Health and Nutrition. 2015. Available at: http://www.hsrc.ac.za/en/research-areas/Research_Areas_PHHSI/sanhanes-health-and-nutrition

Additional reference: Stokes A, Berry KM, McHiza Z, Parker WA, Labadarios D, Chola L, et al. Prevalence and unmet need for diabetes care across the care continuum in a national sample of South African adults: evidence from the SANHANES-1, 2011–2012. PLoS ONE. 2017; 12(10):e0184264. https://doi.org/10.1371/journal.pone.0184264 PMID: 28968435.

Sri Lanka STEPS 2014

A multi stage cluster sampling method was used to select a nationally representative sample from the total population. Department of Census and Statistics of Sri Lanka performed the selection of the study sample. Population of each divisional secretariat (DS) divisions as per the preliminary results of the Census done in 2012 was used for sampling.Sri Lanka is administratively divided in to 9 provinces and 25 districts. Each district is divided to Divisional Secretariat (DS) areas. Each DS area is divided to many Census Blocks, and each Census Block consists of many households.

The primary sampling unit (PSU) was a Divisional Secretariat (DS) area. Out of 331 DS areas available, 80 DS divisions were selected using proportionate to the size (PPS) sampling.
A census block was considered as a SSU. From each DS division (PSU), six secondary sampling units (SSU) were selected using the proportionate to the size (PPS) sampling technique. Therefore, a total of 480 SSUs or census blocks were selected from 80 PSUs.

Number of houses in each census block depends on the area density and the population density in each DS division. Tertiary sampling unit (TSU) was the household and 15 households from each CB by random systematic sampling by the Department Census and Statistics. Therefore, a sample of 7200 (80x6x15) households were selected. In some instances, there were more than one household living in one house. People who are cooking and eating together were considered as one household. Whenever there were more than one household in a house, one household was selected randomly to be included in the study.
Only one participant from each household was included in the survey. All the eligible members in the selected family were listed in descending order according to the age. Once this was done, these data was fed to the personal digital assistants (PDAs). The PDAs then automatically selected the eligible participant using the Kish method.

Age range of participants included: 18 to 69 years

Source: Sri Lanka STEPS 2014 Report. Available at:
https://extranet.who.int/ncdsmicrodata/index.php/catalog/614/study-description#page=overview&tab=study-desc

St. Vincent & the Grenadines: STEPS 2013

"The survey covered the entire island St. Vincent and the Grenadines, and was conducted using the following zoning categories:

1) Mainland (St. Vincent)
2) Northern Grenadines (Bequia and Mustique)
3) Southern Grenadines (Canouan and Union Island)

The sample size was proportionately divided between the three main reporting strata (St.Vincent/Northern Grenadines/Southern Grenadines). The country's most recent age breakdown based on the 2001 national census by St. Vincent was used to approximate the adult population 18-69 years by Island grouping. The survey was stratified by sex, age groups 18-29, 30-44 and 45-69 years and by geographical location – St. Vincent, Northern Grenadines and Southern Grenadines.

A three-stage cluster sampling approach was used. Enumeration districts were randomly selected using Probability Proportional to Size (PPS) from the sampling frame. A total of 199 enumeration districts were selected. The sampling frame was developed using the number of households per enumeration district taken from the 2012 preliminary census report; enumeration districts had been subsequently revised (2010-2011) so that no enumeration district containing more than 150 Households would be randomly selected from the selected enumeration districts. The number of households per enumeration district to be selected was 26. Where an enumeration district had been split into 2 or more new enumeration districts the number of households in the previously defined enumeration district was divided equally between the newly revised enumeration districts. The household list for each selected enumeration district was updated prior to selection of households during a re-listing exercise. This was necessary as the existing household listing for each enumeration district was outdated.

Eligible persons at the household level were randomly selected using the Kish method. If no one was present in the selected household, a notification of visit card was left and the interviewer revisited. There was a total of three visits to the household before it was listed as non-response (one initial recruitment visit and two call backs). The interviewer then moved on to the next house on the list in the original order. Although the person selected for interview were to be at least 18 years and not older than 69 years on the last birthday, there were a few instances where some participants were turning 18 or 70 years; those cases were addressed during data cleaning.

Biological samples, testing and Nutrition intake (24 hour recall):
Fifty percent (50%) of the survey participants were asked to provide a biological specimen (finger prick) for Glucose and cholesterol testing using Glucose and Lipid Sampling Kits and respond to the nutrition intake (24 hour recall). The biological sample was only collected with participants' explicit consent; the samples were not stored or used for additional undetermined or undisclosed future testing to which respondents did not agree at the time of participation."
Age range of participants included: 18 to 69 years
Source: WHO STEPS: Noncommunicable Disease Risk Factor Surveillance. Report for St. Vincent & the Grenadines 2015. Available at: http://www.who.int/ncds/surveillance/steps/stvincent/en/

Sudan: STEPS 2016
A four-stage cluster sampling design was implemented. The four sampling stages were; 1) selection of states from the six regions 2) selection of clusters (a cluster was a Popular Administrative unit), 3) selection of households and 4) selection of eligible individuals. First Stage (State): Administratively Sudan is divided into 18 states which are grouped in six regions, (North, East, Khartoum, Central, Kordofan and Darfur region (Table 1). States were randomly selected from each region. No geographical areas or populations were excluded from the sampling frame. Thus 11 states were selected, probability proportional to the size, to represent the six regions. A list of the selected states is shown in Table 2.1. Second Stage (Cluster PAU): The Popular Administrative Units (PAU) is the smallest geographically border unit. These were defined as the 'cluster' in the region. Clusters were randomly sampled from all PAUs, from both urban and rural strata, according to probability proportional to size in each state, and urban/rural distribution. The PAUs inaccessible due to security conditions were not excluded from the sampling frame, because within certain areas the security status was continuously changing. However, it was planned that if a PAU was found to be inaccessible at survey time, it should be replaced. However, no replacement was required during

this survey. Third Stage (Household): Within the selected PAUs, all households (HH) were included in the sampling frame. Accordingly (HH) were selected using systematic random methods.
Fourth Stage (Individual): The members of the household were first listed in the mobile application (customized software). The inclusion criteria for the listed members were: all individuals aged between 18 to 69 years, from both sexes, irrespective of his health status and living in the selected household for a minimum of 6 weeks. The application was then run and it randomly selected the individual who will be selected to participate in the study.
Age of participants included: 18-69 years.
Source: Sudan STEPS 2016 report. Available at:
https://www.who.int/ncds/surveillance/steps/Sudan_STEPwise_SURVEY_final_2016.pdf?ua=1

Tajikistan: STEPS 2016
A multi-stage cluster sample of households. One individual within the age range of the survey was selected per household.
Analysis weights were calculated by taking the inverse of the probability of selection of each participant. These weights were adjusted for differences in the age-sex composition of the sample population as compared to the target population.

Different weight variables are available per Step:
wStep1 - for interview data
wStep2 - for physical measures
wStep3 - for biochemical measures
This allows for differences in the weight calculation for each Step of the survey as the age-sex composition of the respondents to each Step can differ slightly due to refusal or drop out.
Age range of participants included: 18-69 years
Source: report not available. Sampling information obtained from:
https://extranet.who.int/ncdsmicrodata/index.php/catalog/270/study-description#page=sampling&tab=study-desc

Tanzania: STEPS 2012
"The STEPS survey in the United Republic of Tanzania was a population-based survey of adults aged 25-64. The study used both multistage cluster and random probability sampling procedures. Fifty of 119 total districts were randomly selected as primary sampling units (PSUs). Within these

PSUs, enumeration areas (EAs) of > 50 households were randomly selected. Any EA with < 50 households was merged with a neighboring EA. Within the EAs, households were randomly selected from a list of all eligible households in the EA. A total of 5762 adults participated in the Tanzania STEPS survey. Within each selected household, the Kish method was used to select the STEPS participant. This procedure was followed until the predetermined sample was obtained for the enumeration area. The response rate for this survey was 94.7%."

Age range of participants included: 25 to 64 years

Source: Tanzania STEPS Survey Report. Available at: http://www.who.int/chp/steps/UR_Tanzania_2012_STEPS_Report.pdf?ua=1

Additional reference: Mayige M, Kagaruki G. Tanzania STEPS survey report. Dar es Salaam: National Institute of Medical Research; 2013.

Timor-Leste: STEPS 2014

"Note: Data from Census 2010 were used for all sampling considerations. Even though planning and mapping for 2015 Census is ongoing, data from the Census will only be available after July 2015.

STEP 1: Selection of Enumeration Area

(1) List of EA with number of HH by district for Census 2010 was obtained from the Directorate of Statistics. There are 1826 EAs in Timor-Leste. Out of these, 150 EAs were selected.

(2) The number of EAs to be selected from each district was based on their proportion in the country's population as per Census 2010.

(3) The numbers of Households (HH) per EAs varied from 0 to more than 300. Therefore, probability proportion to size (PPS) was used.

(4) For each district, the EAs were arranged in ascending order of HH size.

(5) Sampling interval was obtained by dividing the total number of HH in the district by the number of EA to be selected from that district.

(6) A random number was generated between one and the sampling interval for that district, using tools available at random.org.

(7) The EA where that random number fell was the first EA to be selected.

(8) Subsequently, the sampling interval was added to the random number and the EA where this new number fell was selected. For the next number, the sampling interval was added to the number and so on, till the population of HH was exhausted or target number of EA achieved.

(9) This was done separately for each district.

(10) The final list was compiled and had 150 EAs. These are spread over about 125 sucos.
STEP 2. Selection of Households in an Enumeration Area
Listing the house numbers to be visited
(1) It was decided to use the 2010 HH size of each EA. Based on past experience, it was expected that the increase would be on an average about 4–5%.
(2) The list of households to be selected by enumerators was decided centrally.
(3) Sampling interval was calculated by dividing the total number of households in the EA by 18.
(4) The first HH number was selected randomly by reading the last two digits of a currency note. If the number represented by the two digits was more than 18, the last digit was taken into consideration. For each EA, a different currency note was used. This could also be done it by using the tool at random.org. or by draw of lots.
(5) The subsequent HH are identified by adding the sampling interval as was done for selection of EA."
Age range of participants included: 18 to 69 years
Source: Timor-Leste STEPS Survey Report, [online] at http://www.who.int/entity/chp/steps/Timor-Leste_2014_STEPS_Report.pdf?ua=1

Togo: STEPS 2010
"Those included in this survey are male or female subjects, living in urban or rural areas, aged 15 to 64 on the day of the survey, residing in the enumeration area for at least 6 months and having given their informed consent to participate in this study. [...] Three hundred clusters were randomly selected in a systematic draw with probability proportional to the size of the cluster (number of households) in the 4620 areas of enumeration of the DGSCN (General Directorate of Statistics and National Accounts) sampling frame. In order to obtain the 4,800 households at the rate of 1 individual / household, 16 households per cluster were randomly selected at the second stage of survey. In each of the selected households, one individual was selected as a survey participant via the Kish Method. A household was defined as the group of persons, who regularly share the main meal (regardless of their relationship). Households were not replaced in the event of a refusal or two unsuccessful visits to the eligible person selected by Kish's method. If the selected person was unwell or not present at the time of the interview, the investigators either tried to find a new appointment or searched for the respondent."
Age range of participants included: 15 to 64 years

Source: Translated from WHO: The Final Report on the Togo STEPS Survey 2010. Available at: http://www.who.int/chp/steps/2010STEPS_Report_Togo_FR.pdf?ua=1.

Tokelau STEPS 2014

A whole population-based (census) survey was used to produce representative data for that age range in Tokelau. Analysis weights contain adjustments for differences in the age-sex composition of the sample population as compared to the target population.

Different weight variables are available per Step:

wStep1 - for interview data

wStep2 - for physical measures

wStep3 - for biochemical measures

This allows for differences in the weight calculation for each Step of the survey as the age-sex composition of the respondents to each Step can differ slightly due to refusal or drop out.

Source: Report unavailable. Sampling information obtained from: https://extranet.who.int/ncdsmicrodata/index.php/catalog/638/overview#page=sampling&tab=study-desc

Turkmenistan: STEPS 2018

Sample

The main purpose of the sample design for STEPS research in Turkmenistan - nationwide coverage and reflection of the situation in the country as a whole for measurable indicators.

The survey was conducted among adults in Turkmenistan aged 18-69 years. (target population), who gave written informed consent, for exceptions: persons in the ranks of the National Armed Forces; population WHO STEPS Non-communicable disease risk assessment 26 www.who.int/chp/steps permanently residing (staying) in specialized institutions social and rehabilitation assistance, hospitals and other institutions health care, correctional facilities.

Method of sampling and stratification

The STEPS study was used to generate a sample set two-stage probability sampling method using stratification procedures and selection at each of the sampling stages. Geographical coverage - all regions of Turkmenistan: Akhal, Balkan, Dashoguz, Lebap and Mary provinces and the city of Ashgabat (the capital), which corresponds national administrative-territorial division. To ensure the uniformity of the distribution of the sample set across the country was

stratification. Taking into account the division of each province into urban and rural
The total population was determined by 11 streets (the city of Ashgabat - only the city street, in velayatakh - 10 strat). The total sample size was distributed in proportion to the number households on the streets.

Age range of participants included: 18 to 69 years
Source: Translated from 2018 STEPS Survey Report. Available at: https://www.who.int/ncds/surveillance/steps/turkmenistan/en/

Tuvalu: STEPS 2015
"The Tuvalu STEPS Survey was a population based cross-sectional survey of 18-69 year olds. Analysis weights were calculated by taking the inverse of the probability of selection of each participant. These weights were adjusted for differences in the age-sex composition of the sample population as compared to the target population.

Different weight variables are available per Step:
wStep1 - for interview data
wStep2 - for physical measures
wStep3 - for biochemical measures
This allows for differences in the weight calculation for each Step of the survey as the age-sex composition of the respondents to each Step can differ slightly due to refusal or drop out. Additionally, some countries perform subsampling for Step 2 and/or Step 3. When no subsampling is done and response rates do not differ across Steps of the survey, the 3 weight variables will be the same."
Age range of participants included: 18 to 69 years
Source: no report or fact sheet available. Sampling information obtained from: https://extranet.who.int/ncdsmicrodata/index.php/catalog/639/study-description#page=overview&tab=study-desc

Vanuatu: STEPS 2011
"The survey used a cluster sampling design where the primary sampling unit was enumeration area (EA) and the secondary sampling unit was households. All 6 provinces in Vanuatu were included in

the survey. One hundred and thirteen (113) EAs were randomly selected proportion to the size of the EA from a total of 411 EAs. Forty four (44) households were then randomly selected in each EA proportional to the number of households in each EA. The selection of participants within each household was done using the Kish method. The total number of households selected by combined Enrolment Areas was 4,972.

The required sample size was calculated as 4972 households on a margin of error of 0.05, an anticipated response rate of 89% and with 80% power to detect statistically significant differences between six age/sex groups. Accordingly, from the 4,972 selected households 4,649 individuals aged 25-64 years participated in STEP 1 and STEP 2 giving an overall response rate of 94%. The response rate dropped to 85% for STEP 3 with 4,224 people participating."
Age range of participants included: 25 to 64 years
Source: Vanuatu STEPS report [online]:
https://extranet.who.int/ncdsmicrodata/index.php/catalog/714

Vietnam: STEPS 2015
At the same time of STEP survey, MOH also conduct the Global Adult Tobacco Survey (GATS) at the same scale, location, and study subjects (>15 years for GATS and 18-69 for STEPS). The sampling of STEPS was done in as part of the sampling for the (GATS) conducted in combination manner to save time and resources for these two surveys. Applied the multi-stages complex sampling process, the sampling process done by GSO was as follow: · Sampling of clusters (EA) In the first stage of sampling, the primary sampling unit (PSU) was an enumeration area (EA). There are about 170,000 EAs in the whole Viet Nam and the average number of households in each EA is different between urban and rural areas. An average number of households in an urban EA and a rural EA is 133 households and 120 households, respectively. Sample of EAs were selected from the master sample frame. The master sample frame was a cluster frame made by the GSO based on the frame of Population and Housing Census 2009 and updated with data of 2014. Based on the Population and Housing Census data 2009, GSO prepared a 15% of master sample to serve as a national survey sampling frame. The master sample frame contains 25,500 enumeration areas (EAs) from 706/708 districts of Viet Nam (2 island districts were excluded from the GSO master sample frame). The master sample frame of GSO was divided by two stratification variables: urbanization (1 = urban; 2 = rural) and district group (1 = district/town/city of province; 2 = plain and coastal district; 3 = mountainous, island district). It means that the master sample frame was divided into 6 sample

frames or 6 strata. The probability proportional to size (PPS) sampling method was used to select sample of EAs from 6 strata of master sample frame. The final sample of GATS included 315 EAs in the urban and 342 EAs for the rural. From these 657 EAs, 315 EAs were systematically selected for STEPS.

Sampling of households At the second stage of sampling, 10% households in each EA were selected. Thus, 15 households from the selected urban EA and 14 households from the selected rural EA were chosen using simple systematic random sampling. The total households for STEPS 2015 were 4,651 households.

Sampling of individuals: One eligible person is then randomly selected from each selected household for the STEPS 1 interview. The selection of individual is automatically done by the PDA program after eligible household members are entered into the PDA. The selection probability of an eligible individual was calculated as a product of selection probability for each stage. The sampling base weight for an eligible individual was the inverse of the selection probability shown above.

Age range of participants included: 18 to 69 years

Source: National Survey on the Risk Factors of Non-communicable diseases (STEPS) Viet Nam Report 2015. Available at: https://www.who.int/ncds/surveillance/steps/viet_nam/en/

Zambia: STEPS 2017

To ensure that the sample reflected the entire country of Zambia, a multi-stage cluster sampling technique was used to select a nationally representative sample of adults in Zambia aged 18 to 69 years. It was decided to utilize the household listing from the Zambia PopulationBased HIV Impact Assessment (ZAMPHIA) - a household-based national survey that was conducted between March and August 2016 in order to measure the status of Zambia's national HIV response. ZAMPHIA offered the most pragmatic up to date and accessible national household listing to be used as the sampling frame for this survey. The ZAMPHIA survey included 60,581 households drawn from 1,103 clusters referred to in this report as standard enumeration area (SEA) (Table 2.4.1). Thus the sample drawn for the STEPS survey was a subsample of the households selected for the ZAMPHIA survey. In the first stage of sampling, SEAs were selected from each province using probability proportional to size (PPS). In the second stage, 15 households in rural SEAs and 20 households in urban SEAs were selected systematically using appropriate sampling interval based on the number of households in that SEA. These households constituted the final list of households for the STEPS survey prepared for the field investigators (FI). In the third stage, while the FI approached the household and sought consent, all eligible members in the household were entered into the

Android-based devise used for the survey. The device then selected one member from the eligible members using a simple random sampling technique. The selected member was then interviewed having gone through the ethical process of consent after being provided with information on the survey. If the selected member was not available, a scheduled visit was made. If the selected member could not be reached after two scheduled visits he or she was considered as non-response. There was no replacement strategy so as to maintain the integrity and representativeness of the sample.

Age range of participants included: 18 to 69 years

Source: STEPS 2017 Report. Available at:
https://extranet.who.int/ncdsmicrodata/index.php/catalog/620

Zanzibar: STEPS 2011

"The survey took place in June and July 2011, followed by data cleaning and analysis. One Principal Investigator and five assistant researchers coordinated the survey on site, checked completed questionnaires daily, and organized logistics. The six data collection teams consisted each of six interviewers, one supervisor, one laboratory technician and one driver. Interviewers were either health care workers or professional interviewers familiar with household surveys such as DHS. The sample size was calculated to be 2800 participants. Each interviewer did on average 3 – 4 interviews a day and was assisted on site by local village guides.

The study was a cross-sectional population based survey with a sample of a sufficient size with a power to determine the proportion of adults that are exposed to selected risk factors associated with NCDs; including those having raised BP, FBG or blood lipids, had experienced injuries or traumas in recent times, and/or were mentally unwell (anxiety, depression), as well as linking these conditions with one another and with the sociodemographic and economic information obtained. People reported to be permanent residents (spending on average maximum 3 nights per week outside the house, and not holding an address in another place) in the selected households and fulfilled the inclusion criteria were enrolled into the survey. A person could only appear once in the study. Therefore we classified a husband practicing polygamy to be listed in the household of his first wife but not to be a member in the household of the following wives. Inclusion criteria was age between 25 - 64 years, able to understand the information given by the interviewer about the study prior to the beginning of the interview, signing of the informed consent for accepting participation. Exclusion criteria was inability to understand or comprehend the information given

by data collector, inability to communicate through verbal expression for consent and for responding to the questionnaires, severe/terminal illness that hinders participation in the survey.

The target population is the entire population in Zanzibar whereby the whole of Zanzibar was selected as the survey site, and hence all districts included. The total population is estimated to be 1.2 million distributed unevenly between 10 districts. The sampling frame represented the entire population in Zanzibar. The sampling strategy used is a multi-stage cluster sampling with stratification. The ten districts are considered as different strata, and the total number of primary sampling units, PSU, is allocated proportionately across all strata. Each district is divided into smaller clusters. These clusters are the geographical and administrative units called Shehia11. The Shehia are divided into smaller clusters called zones (also called mitaa, vitongoji, or vijiji) which typically consist of 100-300 households. Zones smaller than that were merged to make up one larger cluster, and zones much larger were split in smaller clusters.

At the first stage clusters were selected using Simple Random Selection, SRS, from the list of clusters (Shehia) within each district. At the second stage clusters (zones) were randomly selected using probability proportionate to size (PPS). At the third stage households were randomly selected from the household lists provided by the administrative leader of the Shehia. The two last stages of sampling were done using the software STEPSsampling.xls from WHO. Finally participants were selected from the household using Kish method. The household lists were complete and included households with no eligible participants for the survey. Therefore an extra 7 households were sampled at third stage in each cluster for replacement in case a selected household had no eligible participants and had to be changed. This was done before data collectors went to the cluster.

Resources allowed for 100 PSU which was why 2800/100 = 28 households were selected from each PSU (and disproportionate from each SSU). A structured questionnaire was used, based on WHO STEPwise approach to chronic diseases risk factor surveillance.. After getting behavioural and socio-demographic information, anthropometric measurements (BP, height, weight, waist and hip circumference) was done the same day. Answers were recorded electronically during interview using a Personal Digital Assistant (PDA). Biochemical measurements (fasting blood glucose, triglyceride, and cholesterol levels) were done the next day at a central place in each study site according to appointment and were done by Laboratory technicians using dry chemistry for rapid

and convenient results and to avoid suspicion surrounding sending away blood samples. Results were recorded electronically on site using a PDA, and participants received a paper copy of the results.

Every study site was visited one day for interviews. Sampled households/ participants were visited at least three times before recorded as non-respondent. The following day the site was visited for biochemical measurements. Laboratory technicians called participants who did not show up to ask them to set up appointment for the following day (at a new study site). After all study sites had been visited call-backs were made to all eligible participants (non-respondents) who's number we had obtained. A time and place near the participants was identified for data collection. Participants met fasting and started with having blood sample drawn, afterwards the interviews and anthropometric measurements were conducted. Laboratory technicians continued biochemistry measurements for another few days.
Age range of participants included: 25 to 69 years
Source: Zanzibar STEPS Survey Report, [online]
https://www.who.int/ncds/surveillance/steps/2011_Zanzibar_STEPS_Report.pdf

Data included in this study are publicly available for 61 of the 69 included country surveys. Microdata can be downloaded at the following links:

Chile National Health Survey: https://www.minsal.cl/estudios_encuestas_salud/

China Health and Nutrition Survey: https://www.cpc.unc.edu/projects/china

Indonesia Family Life Survey (IFLS): https://www.rand.org/well-being/social-and-behavioral-policy/data/FLS/IFLS.html

Mexico National Survey on Health and Nutrition (ENSANUT):
https://ensanut.insp.mx/encuestas/ensanut2018/descargas.php

STEPS Microdata repository: https://extranet.who.int/ncdsmicrodata/index.php/catalog/STEPS)

For countries without publicly available microdata (Belize, Burkina Faso, Costa Rica, Iran, Romania, Seychelles, South Africa, and St. Vincent & the Grenadines*), please contact Paul Martin at pmartin@hsph.harvard.edu for further information on requesting data.

The generic versions of the World Health Organization STEPwise approach to noncommunicable disease surveillance (WHO STEPS) instrument are available online (accessed March 10, 2020) at the following links:

Version 2.1:
https://www.who.int/ncds/surveillance/steps/STEPS_Instrument_v2.1.pdf

Version 3.2:
https://www.who.int/ncds/surveillance/steps/instrument/STEPS_Instrument_V3.2.pdf

Appendix Table 1: Consolidated Health Economic Evaluation Reporting Standards (CHEERS) checklist.[12]

yes

Section/item	Item No	Recommendation	Section
Title and abstract			
Title	1	Identify the study as an economic evaluation or use more specific terms such as "cost-effectiveness analysis", and describe the interventions compared.	Title
Abstract	2	Provide a structured summary of objectives, perspective, setting, methods (including study design and inputs), results (including base case and uncertainty analyses), and conclusions.	Abstract
Introduction			
Background and objectives	3	Provide an explicit statement of the broader context for the study.	Background paragraph 1
		Present the study question and its relevance for health policy or practice decisions.	Background paragraph 2
Methods			
Target population and subgroups	4	Describe characteristics of the base case population and subgroups analysed, including why they were chosen.	Methods section 2, Table 1
Setting and location	5	State relevant aspects of the system(s) in which the decision(s) need(s) to be made.	Methods, section 3
Study perspective	6	Describe the perspective of the study and relate this to the costs being evaluated.	Methods, section 4
Comparators	7	Describe the interventions or strategies being compared and state why they were chosen.	Methods, section 3
Time horizon	8	State the time horizon(s) over which costs and consequences are being evaluated and say why appropriate.	Methods, section 4
Discount rate	9	Report the choice of discount rate(s) used for costs and outcomes and say why appropriate.	Methods, section 4
Choice of health outcomes	10	Describe what outcomes were used as the measure(s) of benefit in the evaluation and their relevance for the type of analysis performed.	Methods, sections 3-4
Measurement of effectiveness	11a	Single study-based estimates: Describe fully the design features of the single effectiveness study and why the single study was a sufficient source of clinical effectiveness data.	NA
	11b	Synthesis-based estimates: Describe fully the methods used for identification of included studies and synthesis of clinical effectiveness data.	Appendix
Measurement and valuation of preference based outcomes	12	If applicable, describe the population and methods used to elicit preferences for outcomes.	Appendix
Estimating resources and costs	13a	Single study-based economic evaluation: Describe approaches used to estimate resource use associated with the alternative interventions. Describe primary or secondary research methods for valuing each resource item in terms of its unit cost. Describe any adjustments made to approximate to opportunity costs.	NA
	13b	Model-based economic evaluation: Describe approaches and data sources used to estimate resource use associated with model health states. Describe primary or secondary research methods for valuing each resource item in terms of its unit cost. Describe any adjustments made to approximate to opportunity costs.	Appendix

Currency, price date, and conversion	14	Report the dates of the estimated resource quantities and unit costs. Describe methods for adjusting estimated unit costs to the year of reported costs if necessary. Describe methods for converting costs into a common currency base and the exchange rate.	Methods section 4, Appendix
Choice of model	15	Describe and give reasons for the specific type of decision-analytical model used. Providing a figure to show model structure is strongly recommended.	Methods, section 1
Assumptions	16	Describe all structural or other assumptions underpinning the decision-analytical model.	Methods section 1, Appendix
Analytical methods	17	Describe all analytical methods supporting the evaluation. This could include methods for dealing with skewed, missing, or censored data; extrapolation methods; methods for pooling data; approaches to validate or make adjustments (such as half cycle corrections) to a model; and methods for handling population heterogeneity and uncertainty.	Appendix
Results			
Study parameters	18	Report the values, ranges, references, and, if used, probability distributions for all parameters. Report reasons or sources for distributions used to represent uncertainty where appropriate. Providing a table to show the input values is strongly recommended.	Table 1, Appendix
Incremental costs and outcomes	19	For each intervention, report mean values for the main categories of estimated costs and outcomes of interest, as well as mean differences between the comparator groups. If applicable, report incremental cost-effectiveness ratios.	Table 3
Characterising uncertainty	20a	Single study-based economic evaluation: Describe the effects of sampling uncertainty for the estimated incremental cost and incremental effectiveness parameters, together with the impact of methodological assumptions (such as discount rate, study perspective).	NA
	20b	Model-based economic evaluation: Describe the effects on the results of uncertainty for all input parameters, and uncertainty related to the structure of the model and assumptions.	Table 3
Characterising heterogeneity	21	If applicable, report differences in costs, outcomes, or cost-effectiveness that can be explained by variations between subgroups of patients with different baseline characteristics or other observed variability in effects that are not reducible by more information.	Table 3
Discussion			
Study findings, limitations, generalisability, and current knowledge	22	Summarise key study findings and describe how they support the conclusions reached. Discuss limitations and the generalisability of the findings and how the findings fit with current knowledge.	Discussion section 1
Other			
Source of funding	23	Describe how the study was funded and the role of the funder in the identification, design, conduct, and reporting of the analysis. Describe other non-monetary sources of support.	Funding statement
Conflicts of interest	24	Describe any potential for conflict of interest of study contributors in accordance with journal policy. In the absence of a journal policy, we recommend authors comply with International Committee of Medical Journal Editors recommendations.	Competing interest statement

Appendix Table 2: Descriptive statistics on the study sample, by country cohort. A spreadsheet form is available for download at: https://docs.google.com/spreadsheets/d/1j2ETzKfB3V5a_jfF-_6nBJosHqJyzx4C5uAWt8f9tv8/edit?usp=sharing

Covariate	Total (n [%])	Females (n [%])	Age (median, IQR)	BMI kg/m2 (median [IQR])	Currently smoke tobacco (n [%])	History of heart attack (n [%])	Systolic blood pressure, mmHg (median [IQR])	Diastolic blood pressure, mmHg (median [IQR])	Fasting blood glucose mmol/L (median [IQR])	HbA1c, % (median [IQR])	Total cholesterol, mg/dL (median [IQR])	HDL cholesterol, mg/dL (median [IQR])	LDL cholesterol, mg/dL (median [IQR])	Clinical diabetes (n [%])	Diabetes diagnosis rate(n [%])	On treatment for diabetes in the past 2 weeks (n [%])	Diabetes treatment rate(%)	Current insulin use (n [%])	HbA1c <=7% or FBG<7 mmol/L (n [%])	Diabetes control rate,(% of those diagnosed and treated)	Hypertension(n [%])	Previous diagnosis of hypertension (n [% of those with hypertension])	Medication for raised blood pressure, past 2 weeks (n [%])	Hypertension treatment rate(%)	SBP <130 & DBP < 80 (n [%])	Hypertension control rate, (% of those diagnosed and treated)	Statin treatment indicated (n [% total])	Current statin use
Overall	23 678	14 164 (59·8 %)	53·0 (42·0 – 61·0)	27·8 (24·0 – 32·1)	3513 (14·8 %)	2522 (10·7 %)	134·0 (121·0–150·7)	82·3 (74·5 – 91·0)	8·3 (7·2–11·0)	7·4 (6·6–9·2)	177·9 (149·7–210·0)	41·0 (33·0 – 50·3)	101·6 (76·2 – 128·3)	23 678 (100 %)	11 967 (50·5 %)	9420 (39·8 %)	78·7 %	3599 (15·2 %)	11 357 (48·0 %)	52·6 %	13 217 (55·8 %)	9288 (70·3 %)	6322 (26·7 %)	68·1 %	6646 (28·1 %)	16·3 %	18 515 (78·2 %)	2285 (9·7 %)
Afghanistan	407	217 (53.3)	45.00 [34.00, 57.00]	26.66 [23.21, 30.45]	40 (9.8)	62 (15.2)	129.00 [118.00, 144.75]	84.50 [76.00, 93.50]	8.44 [7.22, 10.78]	7.50 [6.60, 9.40]	149.00 [116.00, 193.00]	37.51 [31.00, 47.00]	83.60 [57.85, 114.83]	407 (100.0)	164 (40.3)	129 (31.7)	129 (31.7)	65 (16.0)	190 (46.7)	69 (17.0)	204 (50.1)	165 (40.5)	107 (26.3)	107 (26.3)	128 (31.4)	18 (4.4)	239 (58.7)	29 (7.1)
Algeria	714	410 (57.4)	53.00 [43.00, 61.00]	27.95 [24.71, 31.63]	79 (11.1)	87 (12.2)	137.00 [124.33, 152.00]	78.67 [71.00, 86.00]	8.32 [7.22, 10.82]	7.10 [6.60, 9.00]	170.00 [143.00, 199.00]	43.00 [35.00, 51.00]	100.90 [79.85, 122.60]	714 (100.0)	463 (64.8)	417 (58.4)	417 (58.4)	146 (20.4)	387 (54.2)	241 (33.8)	415 (58.1)	308 (43.1)	220 (30.8)	220 (30.8)	206 (28.9)	31 (4.3)	583 (81.7)	121 (16.9)
Armenia	134	89 (66.4)	57.50 [49.25, 63.00]	30.05 [26.15, 33.88]	22 (16.4)	30 (22.4)	141.00 [129.50	90.00 [82.12, 98.00]	8.95 [7.12, 12.08]	7.75 [6.70, 9.70]	189.48 [168.99	41.57 [32.48, 51.76]	113.13 [96.44, 140.38]	134 (100.0)	75 (56.0)	64 (47.8)	64 (47.8)	23 (17.2)	61 (45.5)	33 (24.6)	98 (73.1)	60 (44.8)	45 (33.6)	45 (33.6)	16 (11.9)	3 (2.2)	113 (84.3)	9 (6.7)

							, 157.88]				, 226.61]																	
Azerbaijan	269	177 (65.8)	57.00 [50.00, 62.00]	29.48 [26.64, 33.46]	32 (11.9)	50 (18.6)	141.33 [128.33 , 163.00]	87.00 [79.67, 96.00]	8.70 [7.40, 11.80]	7.80 [6.70, 9.20]	191.80 [168.60 , 222.35]	40.22 [33.64, 47.56]	115.40 [90.73, 140.12]	269 (100.0)	166 (61.7)	151 (56.1)	151 (56.1)	44 (16.4)	112 (41.6)	60 (22.3)	190 (70.6)	147 (54.6)	105 (39.0)	105 (39.0)	40 (14.9)	9 (3.3)	230 (85.5)	15 (5.6)
Bangladesh	677	345 (51.0)	46.00 [38.00, 55.00]	24.92 [22.29, 27.82]	128 (18.9)	104 (15.4)	127.00 [116.33 , 142.33]	84.67 [77.00, 92.00]	8.61 [7.33, 11.61]	7.50 [6.60, 9.30]	185.00 [161.00 , 215.00]	36.00 [32.00, 41.00]	111.20 [88.00, 134.20]	677 (100.0)	347 (51.3)	253 (37.4)	253 (37.4)	86 (12.7)	307 (45.3)	132 (19.5)	336 (49.6)	256 (37.8)	154 (22.7)	154 (22.7)	221 (32.6)	34 (5.0)	433 (64.0)	50 (7.4)
Belarus	264	163 (61.7)	58.00 [51.00, 64.00]	31.78 [27.95, 35.72]	54 (20.5)	55 (20.8)	151.00 [138.17 , 168.67]	92.67 [84.33, 100.00]	7.30 [6.20, 9.20]	7.30 [6.50, 9.00]	193.35 [162.80 , 227.28]	46.40 [38.57, 60.71]	113.05 [83.28, 135.11]	264 (100.0)	186 (70.5)	161 (61.0)	161 (61.0)	52 (19.7)	152 (57.6)	103 (39.0)	218 (82.6)	196 (74.2)	158 (59.8)	158 (59.8)	18 (6.8)	3 (1.1)	240 (90.9)	36 (13.6)
Belize	248	182 (73.4)	58.00 [47.00, 69.25]	29.35 [25.97, 33.71]	14 (5.6)	23 (9.3)	132.00 [115.75 , 150.00]	77.00 [70.00, 85.00]	8.97 [7.33, 12.35]	7.40 [6.57, 9.10]	180.00 [152.00 , 217.00]	45.00 [35.97, 58.77]	102.03 [77.89, 133.62]	248 (100.0)	170 (68.5)	139 (56.0)	139 (56.0)	10 (4.0)	123 (49.6)	72 (29.0)	138 (55.6)	115 (46.4)	77 (31.0)	77 (31.0)	85 (34.3)	15 (6.0)	216 (87.1)	16 (6.5)
Benin	312	181 (58.0)	42.00 [31.75, 51.00]	25.34 [21.52, 31.31]	12 (3.8)	24 (7.7)	131.17 [115.67 , 150.75]	86.50 [77.67, 95.75]	7.94 [7.38, 8.95]	7.30 [6.60, 8.82]	189.00 [143.75 , 222.50]	42.00 [33.00, 55.57]	103.48 [75.00, 143.99]	312 (100.0)	31 (9.9)	25 (8.0)	25 (8.0)	16 (5.1)	140 (44.9)	14 (4.5)	157 (50.3)	50 (16.0)	32 (10.3)	32 (10.3)	85 (27.2)	3 (1.0)	169 (54.2)	2 (0.6)
Botswana	125	94 (75.2)	51.00 [43.00, 60.00]	28.72 [22.77, 32.64]	15 (12.0)	8 (6.4)	135.00 [122.00 , 157.00]	83.33 [74.67, 93.00]	7.70 [7.00, 10.80]	7.40 [6.62, 9.00]	162.80 [132.25 , 197.22]	49.88 [39.44, 64.97]	82.18 [54.60, 112.20]	125 (100.0)	56 (44.8)	52 (41.6)	52 (41.6)	25 (20.0)	57 (45.6)	29 (23.2)	83 (66.4)	76 (60.8)	58 (46.4)	58 (46.4)	45 (36.0)	14 (11.2)	101 (80.8)	4 (3.2)
Burkina Faso	100	47 (47.0)	42.00 [35.00, 51.00]	22.29 [19.82, 26.13]	11 (11.0)	4 (4.0)	124.75 [114.00 , 138.62]	81.00 [72.38, 88.25]	7.60 [7.20, 8.75]	6.90 [6.50, 8.30]	133.60 [104.41 , 162.80]	37.90 [24.36, 51.62]	70.90 [55.63, 92.05]	100 (100.0)	8 (8.0)	5 (5.0)	5 (5.0)	2 (2.0)	55 (55.0)	4 (4.0)	32 (32.0)	12 (12.0)	6 (6.0)	6 (6.0)	40 (40.0)	0 (0.0)	55 (55.0)	2 (2.0)
Cabo Verde	78	49 (62.8)	50.00 [44.00, 56.00]	27.31 [23.29, 29.76]	11 (14.1)	15 (19.2)	145.50 [127.50 , 159.88]	87.75 [78.50, 97.25]	7.07 [7.02, 9.03]	7.08 [6.50, 8.28]	154.68 [150.04 , 158.74]	37.16 [28.42, 49.95]	88.08 [72.70, 98.50]	78 (100.0)	19 (24.4)	17 (21.8)	17 (21.8)	7 (9.0)	43 (55.1)	11 (14.1)	52 (66.7)	21 (26.9)	14 (17.9)	12 (15.4)	17 (21.8)	1 (1.3)	64 (82.1)	3 (3.8)
Cambodia	152	111 (73.0)	51.00 [46.00, 59.00]	23.78 [21.48, 25.79]	21 (13.8)	13 (8.6)	126.00 [116.58 , 141.17]	78.33 [70.67, 86.08]	8.46 [7.25, 11.81]	8.05 [6.70, 9.53]	184.00 [167.00 , 217.25]	44.54 [36.00, 54.00]	111.80 [88.50, 139.06]	152 (100.0)	82 (53.9)	62 (40.8)	62 (40.8)	16 (10.5)	66 (43.4)	30 (19.7)	63 (41.4)	56 (36.8)	36 (23.7)	36 (23.7)	74 (48.7)	8 (5.3)	132 (86.8)	16 (10.5)

Chile	538	317 (58.9)	61.00 [51.25, 70.00]	29.80 [26.25, 34.10]	117 (21.7)	68 (12.6)	137.50 [125.50 , 154.50]	78.00 [70.50, 87.50]	7.94 [7.21, 10.88]	7.40 [6.60, 9.09]	193.10 [164.00 , 222.75]	41.00 [35.00, 49.97]	113.80 [90.65, 140.10]	538 (100.0)	331 (61.5)	244 (45.4)	240 (44.6)	46 (8.6)	272 (50.6)	135 (25.1)	335 (62.3)	316 (58.7)	166 (30.9)	160 (29.7)	152 (28.3)	35 (6.5)	496 (92.2)	64 (11.9)
China	648	300 (46.3)	59.80 [50.18, 69.02]	25.06 [22.66, 27.79]	173 (26.7)	44 (6.8)	131.00 [121.00 , 148.00]	82.00 [79.00, 90.00]	8.00 [7.27, 10.18]	6.90 [5.90, 8.43]	198.38 [170.92 , 226.22]	47.76 [40.22, 57.71]	113.74 [91.54, 142.72]	648 (100.0)	232 (35.8)	213 (32.9)	213 (32.9)	54 (8.3)	382 (59.0)	126 (19.4)	358 (55.2)	211 (32.6)	181 (27.9)	181 (27.9)	111 (17.1)	6 (0.9)	602 (92.9)	38 (5.9)
Comoros	101	76 (75.2)	50.00 [40.00, 56.00]	26.40 [22.23, 31.49]	10 (9.9)	2 (2.0)	133.50 [121.00 , 155.00]	85.00 [76.50, 93.50]	8.78 [7.17, 10.89]	6.90 [6.00, 9.04]	169.76 [149.27 , 196.00]	42.54 [34.80, 56.84]	97.20 [72.86, 122.05]	101 (100.0)	57 (56.4)	43 (42.6)	43 (42.6)	14 (13.9)	56 (55.4)	28 (27.7)	55 (54.5)	28 (27.7)	18 (17.8)	18 (17.8)	25 (24.8)	2 (2.0)	70 (69.3)	2 (2.0)
Costa Rica	385	275 (71.4)	60.00 [52.00, 70.00]	29.62 [25.83, 34.11]	28 (7.3)	57 (14.8)	125.00 [115.00 , 135.00]	80.00 [70.00, 80.50]	7.00 [5.50, 8.89]	8.40 [6.70, 9.90]	192.00 [165.00 , 214.00]	36.00 [32.00, 41.00]	120.00 [96.20, 143.20]	385 (100.0)	286 (74.3)	310 (80.5)	255 (66.2)	110 (28.6)	238 (61.8)	210 (54.5)	299 (77.7)	273 (70.9)	264 (68.6)	248 (64.4)	123 (31.9)	73 (19.0)	351 (91.2)	64 (16.6)
Ecuador	341	194 (56.9)	52.00 [43.00, 60.00]	28.87 [25.34, 32.46]	44 (12.9)	38 (11.1)	125.33 [116.00 , 137.00]	77.67 [71.33, 84.33]	8.11 [7.11, 11.50]	7.02 [6.60, 8.80]	190.00 [157.00 , 226.00]	39.06 [32.00, 49.88]	111.63 [81.60, 141.60]	341 (100.0)	161 (47.2)	144 (42.2)	144 (42.2)	37 (10.9)	198 (58.1)	84 (24.6)	126 (37.0)	121 (35.5)	71 (20.8)	71 (20.8)	160 (46.9)	23 (6.7)	278 (81.5)	24 (7.0)
El Salvador	457	328 (71.8)	57.00 [47.00, 65.00]	28.29 [25.10, 32.45]	19 (4.2)	58 (12.7)	126.50 [117.00 , 139.50]	78.50 [70.50, 86.50]	8.94 [7.17, 13.56]	7.90 [6.75, 9.80]	185.00 [160.00 , 210.00]	40.99 [32.48, 49.00]	104.90 [85.40, 127.20]	457 (100.0)	337 (73.7)	81 (17.7)	81 (17.7)	253 (55.4)	203 (44.4)	44 (9.6)	257 (56.2)	253 (55.4)	192 (42.0)	191 (41.8)	175 (38.3)	67 (14.7)	404 (88.4)	75 (16.4)
Eritrea	205	113 (55.1)	53.00 [41.00, 64.00]	21.62 [18.51, 25.54]	12 (5.9)	28 (13.7)	127.00 [113.00 , 145.50]	77.00 [70.00, 85.50]	8.16 [6.66, 9.77]	8.10 [6.90, 9.70]	185.00 [166.00 , 214.00]	46.50 [36.00, 58.00]	110.20 [91.43, 131.00]	205 (100.0)	110 (53.7)	81 (39.5)	81 (39.5)	51 (24.9)	108 (52.7)	58 (28.3)	77 (37.6)	39 (19.0)	30 (14.6)	30 (14.6)	88 (42.9)	3 (1.5)	154 (75.1)	10 (4.9)
Eswatini	169	124 (73.4)	54.00 [39.00, 62.00]	29.41 [25.04, 34.21]	5 (3.0)	11 (6.5)	138.00 [125.50 , 155.50]	85.00 [78.00, 96.00]	8.00 [7.20, 10.40]	7.60 [6.70, 9.10]	169.37 [140.37 , 200.31]	44.00 [34.42, 57.23]	93.40 [66.40, 118.68]	169 (100.0)	63 (37.3)	55 (32.5)	55 (32.5)	20 (11.8)	69 (40.8)	22 (13.0)	103 (60.9)	81 (47.9)	54 (32.0)	54 (32.0)	35 (20.7)	4 (2.4)	123 (72.8)	3 (1.8)
Ethiopia	227	118 (52.0)	45.00 [31.50, 55.00]	21.86 [18.99, 25.53]	20 (8.8)	7 (3.1)	126.00 [112.00 , 143.25]	81.50 [74.50, 89.50]	7.56 [7.11, 9.14]	7.20 [6.50, 9.00]	151.00 [123.00 , 180.00]	39.00 [31.00, 49.00]	82.00 [57.93, 106.63]	227 (100.0)	66 (29.1)	55 (24.2)	55 (24.2)	26 (11.5)	111 (48.9)	31 (13.7)	85 (37.4)	47 (20.7)	21 (9.3)	21 (9.3)	85 (37.4)	4 (1.8)	121 (53.3)	1 (0.4)
Fiji	430	260 (60.5)	51.00 [44.00, 57.00]	28.46 [25.41, 32.99]	80 (18.6)	60 (14.0)	141.00 [127.00	85.00 [78.62, 92.00]	9.00 [7.52, 12.40]	7.70 [6.89, 9.70]	181.75 [157.54	43.31 [34.00, 54.91]	102.34 [84.20, 132.00]	430 (100.0)	229 (53.3)	26 (6.0)	26 (6.0)	158 (36.7)	140 (32.6)	8 (1.9)	275 (64.0)	180 (41.9)	112 (26.0)	111 (25.8)	76 (17.7)	7 (1.6)	370 (86.0)	16 (3.7)

							, 159.50]				, 213.77]																	
Georgia	262	179 (68.3)	59.00 [53.00, 64.00]	32.72 [29.05, 37.60]	46 (17.6)	94 (35.9)	145.00 [127.50 , 161.88]	88.50 [79.62, 97.00]	8.60 [7.10, 10.97]	8.20 [6.90, 9.67]	186.50 [154.78 , 218.87]	43.00 [34.00, 52.99]	105.40 [82.94, 135.20]	262 (100.0)	174 (66.4)	152 (58.0)	152 (58.0)	46 (17.6)	113 (43.1)	77 (29.4)	201 (76.7)	152 (58.0)	129 (49.2)	129 (49.2)	45 (17.2)	12 (4.6)	253 (96.6)	20 (7.6)
Guyana	129	89 (69.0)	53.00 [45.00, 61.00]	29.18 [24.87, 32.17]	4 (3.1)	20 (15.5)	135.50 [123.00 , 147.00]	82.50 [75.50, 92.50]	9.33 [7.28, 12.56]	8.50 [6.70, 9.80]	206.00 [183.00 , 233.00]	47.18 [36.74, 60.00]	120.00 [99.60, 147.80]	129 (100.0)	84 (65.1)	73 (56.6)	73 (56.6)	15 (11.6)	52 (40.3)	32 (24.8)	81 (62.8)	80 (62.0)	54 (41.9)	54 (41.9)	41 (31.8)	16 (12.4)	105 (81.4)	9 (7.0)
Indonesia	530	322 (60.8)	53.00 [36.00, 62.00]	24.25 [20.90, 27.42]	124 (23.4)	23 (4.3)	133.50 [121.00 , 153.00]	80.50 [73.00, 89.50]	9.16 [7.55, 11.77]	7.69 [6.89, 9.31]	172.66 [146.27 , 203.01]	42.54 [32.10, 56.46]	97.90 [74.61, 123.25]	530 (100.0)	128 (24.2)	87 (16.4)	87 (16.4)	15 (2.8)	165 (31.1)	41 (7.7)	255 (48.1)	161 (30.4)	63 (11.9)	63 (11.9)	175 (33.0)	9 (1.7)	369 (69.6)	13 (2.5)
Iran	1755	1036 (59.0)	59.00 [51.00, 66.00]	28.58 [25.58, 31.74]	189 (10.8)	112 (6.4)	136.50 [122.75 , 151.50]	80.00 [72.50, 88.00]	8.31 [7.06, 11.32]	7.70 [6.60, 9.30]	162.87 [136.89 , 193.85]	37.80 [31.60, 45.00]	93.28 [69.59, 118.81]	1755 (100.0)	1358 (77.4)	1064 (60.6)	1064 (60.6)	243 (13.8)	736 (41.9)	513 (29.2)	1049 (59.8)	920 (52.4)	623 (35.5)	623 (35.5)	508 (28.9)	112 (6.4)	1622 (92.4)	512 (29.2)
Iraq	693	403 (58.2)	52.00 [42.00, 62.00]	29.96 [26.26, 34.25]	116 (16.7)	98 (14.1)	140.00 [126.00 , 150.00]	87.67 [80.00, 92.00]	8.66 [7.22, 12.15]	7.70 [6.70, 9.50]	194.00 [157.00 , 223.00]	42.00 [36.00, 50.00]	116.20 [82.86, 144.40]	693 (100.0)	457 (65.9)	385 (55.6)	385 (55.6)	90 (13.0)	304 (43.9)	196 (28.3)	450 (64.9)	359 (51.8)	249 (35.9)	249 (35.9)	78 (11.3)	6 (0.9)	541 (78.1)	40 (5.8)
Jordan	484	304 (62.8)	55.50 [48.00, 63.00]	32.25 [28.11, 36.34]	117 (24.2)	99 (20.5)	127.50 [116.00 , 141.00]	83.50 [76.50, 90.00]	7.55 [5.57, 9.80]	7.80 [6.79, 9.50]	151.01 [119.88 , 181.75]	37.12 [31.32, 43.70]	80.57 [55.39, 108.93]	484 (100.0)	404 (83.5)	385 (79.5)	385 (79.5)	118 (24.4)	262 (54.1)	224 (46.3)	319 (65.9)	298 (61.6)	252 (52.1)	252 (52.1)	126 (26.0)	61 (12.6)	436 (90.1)	135 (27.9)
Kenya	109	78 (71.6)	48.00 [39.00, 58.00]	27.14 [22.26, 30.09]	5 (4.6)	6 (5.5)	132.50 [118.50 , 150.50]	87.00 [75.00, 96.00]	8.00 [7.10, 10.40]	7.10 [6.60, 8.50]	178.00 [147.33 , 208.00]	40.22 [32.00, 47.00]	98.10 [76.00, 122.60]	109 (100.0)	38 (34.9)	33 (30.3)	33 (30.3)	17 (15.6)	56 (51.4)	18 (16.5)	64 (58.7)	40 (36.7)	18 (16.5)	18 (16.5)	29 (26.6)	2 (1.8)	75 (68.8)	0 (0.0)
Kiribati	219	127 (58.0)	46.00 [38.00, 55.00]	30.31 [26.41, 34.22]	92 (42.0)	22 (10.0)	132.00 [121.50 , 145.33]	86.33 [78.83, 94.67]	8.40 [7.40, 10.70]	7.80 [6.73, 8.85]	157.00 [127.22 , 190.26]	27.46 [19.53, 35.19]	90.60 [71.90, 118.03]	219 (100.0)	66 (30.1)	43 (19.6)	43 (19.6)	18 (8.2)	83 (37.9)	18 (8.2)	119 (54.3)	53 (24.2)	26 (11.9)	26 (11.9)	51 (23.3)	5 (2.3)	156 (71.2)	2 (0.9)
Kyrgyzstan	153	97 (63.4)	53.00 [45.00, 58.00]	29.30 [25.57, 33.79]	19 (12.4)	39 (25.5)	149.00 [133.00 , 169.33]	94.33 [87.50, 104.00]	8.80 [7.40, 12.00]	7.20 [6.60, 8.70]	182.14 [157.39 , 208.82]	37.90 [33.00, 46.00]	107.40 [87.01, 129.60]	153 (100.0)	73 (47.7)	59 (38.6)	59 (38.6)	20 (13.1)	73 (47.7)	27 (17.6)	121 (79.1)	78 (51.0)	48 (31.4)	48 (31.4)	10 (6.5)	1 (0.7)	132 (86.3)	9 (5.9)

Laos	131	92 (70.2)	50.00 [42.50, 56.50]	24.39 [21.40, 26.86]	35 (26.7)	20 (15.3)	124.67 [113.83 , 138.83]	81.33 [73.67, 89.17]	8.90 [7.35, 12.05]	8.20 [6.80, 9.70]	191.42 [157.19 , 216.55]	35.58 [28.42, 43.31]	114.00 [87.49, 137.09]	131 (100.0)	67 (51.1)	52 (39.7)	52 (39.7)	8 (6.1)	51 (38.9)	19 (14.5)	48 (36.6)	32 (24.4)	18 (13.7)	18 (13.7)	51 (38.9)	3 (2.3)	103 (78.6)	11 (8.4)
Lebanon	177	91 (51.4)	57.00 [50.00, 63.00]	28.52 [25.18, 31.64]	80 (45.2)	24 (13.6)	134.33 [123.00 , 150.00]	80.00 [71.00, 85.67]	8.44 [7.10, 11.27]	7.70 [6.70, 9.00]	201.00 [168.00 , 228.00]	42.00 [36.00, 51.00]	123.40 [96.80, 148.20]	177 (100.0)	108 (61.0)	107 (60.5)	107 (60.5)	26 (14.7)	92 (52.0)	58 (32.8)	117 (66.1)	89 (50.3)	76 (42.9)	76 (42.9)	49 (27.7)	18 (10.2)	161 (91.0)	61 (34.5)
Lesotho	82	60 (73.2)	54.00 [48.00, 60.00]	29.05 [24.14, 34.05]	6 (7.3)	12 (14.6)	139.67 [123.00 , 157.92]	87.83 [80.08, 100.08]	7.60 [7.00, 9.67]	7.20 [6.50, 8.60]	152.94 [133.51 , 175.47]	41.18 [34.42, 51.72]	83.68 [57.00, 103.87]	82 (100.0)	42 (51.2)	37 (45.1)	37 (45.1)	11 (13.4)	45 (54.9)	19 (23.2)	57 (69.5)	45 (54.9)	33 (40.2)	33 (40.2)	15 (18.3)	4 (4.9)	69 (84.1)	10 (12.2)
Liberia	304	172 (56.6)	42.00 [32.00, 53.25]	26.67 [23.04, 32.00]	25 (8.2)	13 (4.3)	128.00 [115.50 , 146.12]	80.00 [71.38, 89.00]	7.52 [7.21, 8.25]	6.40 [5.80, 7.30]	174.01 [144.91 , 198.47]	41.57 [33.19, 51.24]	97.19 [75.00, 121.44]	304 (100.0)	20 (6.6)	10 (3.3)	10 (3.3)	8 (2.6)	215 (70.7)	6 (2.0)	119 (39.1)	46 (15.1)	26 (8.6)	26 (8.6)	122 (40.1)	2 (0.7)	159 (52.3)	10 (3.3)
Libya	298	129 (43.3)	50.00 [41.25, 58.00]	28.57 [25.58, 31.85]	77 (25.8)	45 (15.1)	146.50 [132.00 , 165.50]	83.50 [77.50, 91.50]	7.80 [7.00, 10.10]	7.60 [6.62, 9.30]	181.75 [163.28 , 207.66]	41.53 [35.00, 50.27]	105.50 [87.05, 128.92]	298 (100.0)	175 (58.7)	91 (30.5)	91 (30.5)	90 (30.2)	156 (52.3)	51 (17.1)	189 (63.4)	89 (29.9)	65 (21.8)	65 (21.8)	47 (15.8)	2 (0.7)	229 (76.8)	18 (6.0)
Malawi	26	17 (65.4)	41.50 [31.75, 56.50]	25.35 [22.08, 27.58]	0 (0.0)	1 (3.8)	123.33 [117.17 , 140.42]	79.83 [76.50, 84.42]	7.97 [7.18, 9.53]	7.10 [6.50, 8.23]	162.74 [156.78 , 201.14]	39.00 [32.00, 49.28]	96.54 [89.40, 122.35]	26 (100.0)	6 (23.1)	4 (15.4)	4 (15.4)	1 (3.8)	14 (53.8)	3 (11.5)	7 (26.9)	8 (30.8)	2 (7.7)	2 (7.7)	11 (42.3)	0 (0.0)	13 (50.0)	1 (3.8)
Marshall Islands	774	424 (54.8)	49.00 [38.00, 57.00]	29.96 [26.15, 34.07]	149 (19.3)	24 (3.1)	125.00 [112.00 , 137.00]	77.00 [68.33, 85.67]	10.10 [7.94, 13.88]	7.60 [6.40, 9.50]	168.26 [138.21 , 198.31]	39.64 [29.83, 50.27]	92.60 [70.61, 119.79]	774 (100.0)	287 (37.1)	147 (19.0)	147 (19.0)	99 (12.8)	313 (40.4)	48 (6.2)	247 (31.9)	157 (20.3)	75 (9.7)	75 (9.7)	377 (48.7)	28 (3.6)	546 (70.5)	36 (4.7)
Mexico	2233	1383 (61.9)	57.00 [47.00, 67.00]	29.52 [26.45, 33.33]	267 (12.0)	103 (4.6)	135.00 [120.00 , 158.00]	79.00 [71.00, 89.00]	8.28 [6.67, 12.33]	7.70 [6.60, 9.60]	189.00 [164.00 , 216.00]	41.00 [35.00, 48.00]	105.80 [83.80, 128.60]	2233 (100.0)	1485 (66.5)	1350 (60.5)	1350 (60.5)	708 (31.7)	1171 (52.4)	718 (32.2)	1314 (58.8)	914 (40.9)	756 (33.9)	756 (33.9)	724 (32.4)	177 (7.9)	1932 (86.5)	220 (9.9)
Moldova	322	217 (67.4)	58.00 [51.00, 64.00]	30.48 [26.35, 35.19]	31 (9.6)	92 (28.6)	150.00 [133.08 , 170.33]	90.00 [80.67, 98.00]	8.10 [7.10, 10.35]	7.69 [6.60, 9.30]	192.96 [167.54 , 223.03]	49.69 [39.93, 62.45]	107.16 [86.60, 134.76]	322 (100.0)	170 (52.8)	135 (41.9)	134 (41.6)	42 (13.0)	141 (43.8)	71 (22.0)	253 (78.6)	193 (59.9)	140 (43.5)	140 (43.5)	36 (11.2)	5 (1.6)	280 (87.0)	27 (8.4)
Mongolia	589	284 (48.2)	47.00 [36.00, 57.00]	28.51 [24.80, 32.32]	165 (28.0)	110 (18.7)	127.00 [115.00	81.00 [74.00, 90.00]	7.70 [7.10, 9.00]	7.30 [6.70, 8.90]	177.88 [146.95	46.40 [40.00, 58.01]	94.75 [69.78, 121.82]	589 (100.0)	165 (28.0)	100 (17.0)	100 (17.0)	58 (9.8)	262 (44.5)	47 (8.0)	281 (47.7)	256 (43.5)	179 (30.4)	179 (30.4)	231 (39.2)	44 (7.5)	402 (68.3)	39 (6.6)

							, 141.00]				, 208.82]																	
Morocco	680	469 (69.0)	56.00 [46.00, 65.00]	28.40 [24.99, 32.05]	31 (4.6)	32 (4.7)	140.17 [127.67, 156.67]	81.67 [74.58, 88.75]	8.21 [7.27, 10.84]	7.56 [6.75, 9.00]	148.11 [118.00, 175.00]	43.00 [34.00, 52.25]	77.90 [62.00, 100.95]	680 (100.0)	363 (53.4)	314 (46.2)	314 (46.2)	91 (13.4)	263 (38.7)	160 (23.5)	401 (59.0)	229 (33.7)	155 (22.8)	155 (22.8)	154 (22.6)	16 (2.4)	579 (85.1)	40 (5.9)
Myanmar	616	476 (77.3)	51.50 [45.00, 58.00]	25.15 [22.65, 28.00]	64 (10.4)	118 (19.2)	135.00 [123.00, 150.33]	86.67 [79.58, 95.00]	8.71 [7.33, 11.88]	7.70 [6.62, 9.50]	200.00 [171.00, 226.00]	42.00 [37.00, 50.00]	119.90 [93.80, 146.30]	616 (100.0)	389 (63.1)	333 (54.1)	333 (54.1)	32 (5.2)	268 (43.5)	161 (26.1)	399 (64.8)	338 (54.9)	214 (34.7)	214 (34.7)	129 (20.9)	34 (5.5)	541 (87.8)	52 (8.4)
Nauru	175	103 (58.9)	46.00 [35.50, 55.00]	33.62 [29.08, 37.73]	60 (34.3)	35 (20.0)	124.00 [112.75, 136.25]	81.00 [72.00, 89.25]	9.90 [7.85, 12.90]	8.23 [7.29, 9.54]	154.68 [118.91, 190.06]	29.00 [22.04, 36.74]	84.00 [63.90, 114.03]	175 (100.0)	112 (64.0)	65 (37.1)	65 (37.1)	13 (7.4)	39 (22.3)	15 (8.6)	71 (40.6)	53 (30.3)	23 (13.1)	23 (13.1)	75 (42.9)	7 (4.0)	108 (61.7)	12 (6.9)
Nepal	341	198 (58.1)	48.00 [37.00, 57.00]	24.12 [21.63, 26.71]	67 (19.6)	9 (2.6)	133.00 [121.00, 144.33]	85.00 [78.67, 93.00]	8.17 [7.33, 9.67]	7.10 [6.50, 8.70]	180.00 [145.00, 210.75]	39.83 [34.00, 49.00]	104.20 [75.00, 129.40]	341 (100.0)	90 (26.4)	76 (22.3)	76 (22.3)	19 (5.6)	170 (49.9)	34 (10.0)	164 (48.1)	81 (23.8)	50 (14.7)	50 (14.7)	75 (22.0)	4 (1.2)	228 (66.9)	5 (1.5)
Niger	584	290 (49.7)	35.00 [25.00, 47.00]	20.73 [19.04, 22.69]	28 (4.8)	11 (1.9)	130.17 [119.33, 144.00]	80.33 [73.67, 88.00]	10.39 [10.39, 10.39]	6.90 [5.80, 8.30]	167.83 [141.00, 203.79]	44.14 [35.00, 56.94]	92.44 [71.16, 120.47]	584 (100.0)	1 (0.2)	1 (0.2)	1 (0.2)	0 (0.0)	304 (52.1)	0 (0.0)	214 (36.6)	15 (2.6)	5 (0.9)	5 (0.9)	205 (35.1)	1 (0.2)	200 (34.2)	9 (1.5)
Romania	256	107 (41.8)	63.00 [53.00, 70.00]	30.78 [27.96, 34.58]	47 (18.4)	13 (5.1)	138.00 [125.38, 151.00]	80.50 [74.50, 87.50]	7.78 [7.06, 9.31]	6.55 [5.70, 7.60]	193.50 [161.75, 225.09]	47.00 [38.00, 55.25]	116.10 [88.00, 142.51]	256 (100.0)	198 (77.3)	37 (14.5)	37 (14.5)	12 (4.7)	162 (63.3)	36 (14.1)	120 (46.9)	162 (63.3)	0 (0.0)	0 (0.0)	67 (26.2)	0 (0.0)	228 (89.1)	13 (5.1)
Rwanda	104	67 (64.4)	41.00 [28.00, 51.00]	22.29 [20.64, 26.37]	15 (14.4)	6 (5.8)	123.00 [113.67, 137.83]	80.67 [72.92, 89.42]	8.50 [7.40, 9.92]	8.00 [6.90, 9.40]	141.77 [121.33, 183.78]	46.60 [34.42, 63.10]	71.50 [52.54, 104.11]	104 (100.0)	13 (12.5)	6 (5.8)	6 (5.8)	10 (9.6)	31 (29.8)	2 (1.9)	31 (29.8)	5 (4.8)	2 (1.9)	2 (1.9)	46 (44.2)	0 (0.0)	53 (51.0)	1 (1.0)
Samoa	372	208 (55.9)	46.50 [36.00, 55.00]	33.16 [28.31, 37.88]	91 (24.5)	38 (10.2)	129.50 [118.25, 143.54]	79.33 [70.33, 88.00]	8.05 [7.40, 10.50]	7.10 [6.60, 8.32]	162.41 [150.04, 193.35]	40.41 [31.23, 51.78]	94.64 [80.67, 109.89]	372 (100.0)	56 (15.1)	37 (9.9)	37 (9.9)	15 (4.0)	182 (48.9)	9 (2.4)	139 (37.4)	37 (9.9)	22 (5.9)	22 (5.9)	151 (40.6)	2 (0.5)	252 (67.7)	13 (3.5)
Sao Tome and Principe	59	36 (61.0)	41.00 [34.00, 54.00]	25.40 [22.01, 30.82]	4 (6.8)	3 (5.1)	142.33 [124.67, 165.50]	87.00 [77.17, 96.17]	10.07 [7.51, 15.26]	8.10 [7.13, 9.36]	140.00 [140.00, 167.00]	37.00 [30.00, 48.28]	71.24 [61.40, 88.62]	59 (100.0)	27 (45.8)	22 (37.3)	20 (33.9)	17 (28.8)	20 (33.9)	8 (13.6)	40 (67.8)	26 (44.1)	18 (30.5)	18 (30.5)	14 (23.7)	2 (3.4)	30 (50.8)	3 (5.1)

Seychelles	179	97 (54.2)	55.00 [48.50, 59.50]	29.55 [25.82, 34.20]	25 (14.0)	28 (15.6)	137.50 [127.00, 155.00]	82.50 [75.50, 88.00]	8.31 [7.19, 11.33]	7.40 [6.60, 9.20]	192.96 [163.57, 221.39]	46.40 [36.74, 56.84]	117.35 [90.28, 149.64]	179 (100.0)	114 (63.7)	102 (57.0)	102 (57.0)	24 (13.4)	79 (44.1)	45 (25.1)	86 (48.0)	111 (62.0)	0 (0.0)	0 (0.0)	41 (22.9)	0 (0.0)	167 (93.3)	11 (6.1)
Solomon Islands	101	59 (58.4)	48.00 [38.00, 54.00]	27.80 [24.43, 30.08]	26 (25.7)	10 (9.9)	129.33 [118.33, 142.00]	81.67 [74.67, 90.67]	8.30 [7.40, 12.20]	7.80 [6.70, 9.50]	189.48 [155.00, 223.90]	41.76 [31.00, 53.75]	107.57 [81.55, 143.76]	101 (100.0)	17 (16.8)	8 (7.9)	8 (7.9)	4 (4.0)	38 (37.6)	2 (2.0)	39 (38.6)	25 (24.8)	8 (7.9)	8 (7.9)	38 (37.6)	2 (2.0)	70 (69.3)	1 (1.0)
South Africa	588	425 (72.3)	58.50 [49.00, 67.00]	30.57 [26.61, 34.78]	61 (10.4)	73 (12.4)	148.00 [133.00, 167.00]	82.75 [76.25, 91.12]	8.18 [7.20, 10.64]	7.10 [6.60, 8.90]	180.49 [150.04, 214.62]	41.65 [34.30, 50.65]	104.20 [76.55, 134.10]	588 (100.0)	275 (46.8)	251 (42.7)	238 (40.5)	104 (17.7)	346 (58.8)	137 (23.3)	450 (76.5)	289 (49.1)	250 (42.5)	243 (41.3)	86 (14.6)	24 (4.1)	524 (89.1)	77 (13.1)
Sri Lanka	534	337 (63.1)	54.00 [45.00, 61.00]	24.58 [22.03, 27.60]	51 (9.6)	62 (11.6)	134.17 [122.33, 150.58]	86.00 [78.75, 93.33]	7.38 [5.44, 9.27]	7.55 [6.50, 9.70]	149.00 [117.00, 189.00]	38.00 [29.78, 46.31]	80.59 [58.02, 111.85]	534 (100.0)	405 (75.8)	374 (70.0)	374 (70.0)	64 (12.0)	333 (62.4)	258 (48.3)	326 (61.0)	211 (39.5)	180 (33.7)	180 (33.7)	122 (22.8)	23 (4.3)	455 (85.2)	144 (27.0)
St. Vincent & the Grenadines	116	87 (75.0)	55.00 [48.75, 63.00]	29.30 [26.17, 34.03]	5 (4.3)	15 (12.9)	137.25 [118.75, 152.75]	79.50 [71.00, 84.75]	7.50 [5.40, 9.43]	7.24 [6.67, 8.60]	173.24 [135.73, 210.07]	48.34 [36.93, 56.94]	103.62 [69.94, 133.92]	116 (100.0)	95 (81.9)	87 (75.0)	87 (75.0)	24 (20.7)	66 (56.9)	55 (47.4)	79 (68.1)	67 (57.8)	55 (47.4)	55 (47.4)	38 (32.8)	13 (11.2)	102 (87.9)	9 (7.8)
Sudan	569	370 (65.0)	48.00 [39.00, 55.00]	26.32 [22.86, 30.63]	31 (5.4)	17 (3.0)	137.67 [124.67, 153.00]	88.67 [82.00, 95.67]	9.00 [7.40, 13.10]	7.90 [6.60, 9.70]	163.19 [131.86, 199.15]	31.32 [22.82, 42.92]	92.40 [73.10, 122.90]	569 (100.0)	298 (52.4)	252 (44.3)	252 (44.3)	93 (16.3)	253 (44.5)	102 (17.9)	366 (64.3)	161 (28.3)	115 (20.2)	115 (20.2)	75 (13.2)	10 (1.8)	396 (69.6)	25 (4.4)
Tajikistan	70	44 (62.9)	56.00 [46.25, 60.75]	30.52 [26.10, 33.50]	1 (1.4)	9 (12.9)	148.33 [134.83, 164.75]	93.33 [88.08, 103.58]	12.00 [9.30, 15.20]	8.60 [7.00, 10.35]	195.09 [155.94, 225.91]	40.41 [33.45, 45.92]	117.04 [83.08, 143.60]	70 (100.0)	48 (68.6)	38 (54.3)	38 (54.3)	8 (11.4)	23 (32.9)	15 (21.4)	57 (81.4)	36 (51.4)	27 (38.6)	27 (38.6)	6 (8.6)	0 (0.0)	59 (84.3)	7 (10.0)
Tanzania	143	75 (52.4)	49.00 [40.50, 58.00]	24.13 [20.30, 28.06]	14 (9.8)	18 (12.6)	135.00 [122.00, 153.00]	84.50 [76.50, 93.00]	8.20 [7.20, 12.50]	7.20 [6.50, 9.50]	173.87 [150.81, 216.55]	41.90 [35.09, 50.50]	101.40 [78.12, 130.95]	143 (100.0)	64 (44.8)	43 (30.1)	43 (30.1)	16 (11.2)	69 (48.3)	26 (18.2)	77 (53.8)	35 (24.5)	17 (11.9)	17 (11.9)	40 (28.0)	3 (2.1)	107 (74.8)	7 (4.9)
Timor Leste	64	35 (54.7)	45.50 [37.00, 56.50]	22.16 [19.55, 25.62]	21 (32.8)	0 (0.0)	125.75 [113.88, 145.00]	81.50 [74.88, 90.88]	8.10 [7.40, 11.65]	6.60 [5.90, 7.60]	154.68 [116.01, 193.35]	36.54 [26.82, 50.45]	68.80 [49.95, 107.04]	64 (100.0)	7 (10.9)	5 (7.8)	5 (7.8)	0 (0.0)	44 (68.8)	4 (6.2)	25 (39.1)	14 (21.9)	11 (17.2)	11 (17.2)	24 (37.5)	1 (1.6)	44 (68.8)	0 (0.0)

Togo	89	35 (39.3)	45.00 [33.00, 54.00]	23.53 [21.21, 26.70]	10 (11.2)	15 (16.9)	124.50 [115.00 , 136.00]	80.00 [74.00, 87.50]	7.58 [7.21, 10.08]	8.00 [6.70, 9.60]	164.00 [153.00 , 191.03]	41.00 [35.00, 52.00]	94.40 [75.00, 117.44]	89 (100.0)	14 (15.7)	9 (10.1)	9 (10.1)	5 (5.6)	40 (44.9)	8 (9.0)	25 (28.1)	11 (12.4)	6 (6.7)	6 (6.7)	33 (37.1)	2 (2.2)	51 (57.3)	9 (10.1)
Tokelau	155	72 (46.5)	50.00 [42.00, 57.00]	33.99 [29.76, 37.12]	83 (53.5)	11 (7.1)	133.33 [123.50 , 144.67]	85.00 [78.00, 92.83]	9.30 [7.50, 12.85]	7.90 [7.16, 9.50]	201.08 [174.21 , 227.57]	37.90 [30.36, 47.18]	126.69 [99.43, 152.54]	155 (100.0)	89 (57.4)	67 (43.2)	67 (43.2)	8 (5.2)	30 (19.4)	17 (11.0)	74 (47.7)	53 (34.2)	32 (20.6)	32 (20.6)	37 (23.9)	4 (2.6)	120 (77.4)	14 (9.0)
Turkmenistan	263	155 (58.9)	50.00 [40.00, 58.50]	27.59 [24.75, 31.89]	12 (4.6)	70 (26.6)	134.50 [125.50 , 151.25]	89.00 [82.00, 95.00]	8.30 [7.30, 11.50]	7.40 [6.70, 9.60]	182.52 [147.72 , 212.68]	48.72 [39.06, 58.39]	97.70 [68.46, 125.65]	263 (100.0)	64 (24.3)	53 (20.2)	53 (20.2)	10 (3.8)	115 (43.7)	18 (6.8)	155 (58.9)	105 (39.9)	81 (30.8)	81 (30.8)	37 (14.1)	1 (0.4)	191 (72.6)	34 (12.9)
Tuvalu	121	78 (64.5)	52.00 [44.00, 57.00]	33.13 [29.41, 36.82]	34 (28.1)	8 (6.6)	144.00 [130.33 , 157.33]	88.33 [79.67, 98.00]	9.20 [7.30, 12.00]	7.80 [7.02, 9.85]	174.40 [146.17 , 197.99]	23.98 [18.95, 30.55]	102.60 [75.57, 127.40]	121 (100.0)	65 (53.7)	45 (37.2)	45 (37.2)	37 (30.6)	40 (33.1)	17 (14.0)	77 (63.6)	30 (24.8)	20 (16.5)	20 (16.5)	23 (19.0)	1 (0.8)	100 (82.6)	1 (0.8)
Vanuatu	780	375 (48.1)	44.00 [36.00, 54.00]	26.03 [23.01, 29.58]	170 (21.8)	74 (9.5)	133.50 [122.50 , 147.50]	81.25 [73.50, 88.62]	7.89 [7.26, 9.13]	6.90 [6.50, 7.93]	186.78 [170.92 , 216.94]	45.12 [35.96, 57.23]	111.03 [93.66, 134.20]	780 (100.0)	91 (11.7)	63 (8.1)	63 (8.1)	50 (6.4)	419 (53.7)	24 (3.1)	323 (41.4)	87 (11.2)	14 (1.8)	14 (1.8)	237 (30.4)	1 (0.1)	460 (59.0)	6 (0.8)
Vietnam	109	68 (62.4)	54.00 [45.00, 61.00]	23.24 [20.89, 25.75]	22 (20.2)	17 (15.6)	127.33 [117.33 , 146.67]	82.33 [76.33, 93.00]	7.10 [5.30, 9.70]	7.40 [6.60, 9.50]	178.27 [146.95 , 207.27]	40.99 [32.00, 48.00]	100.60 [75.20, 128.84]	109 (100.0)	63 (57.8)	59 (54.1)	59 (54.1)	32 (29.4)	74 (67.9)	53 (48.6)	61 (56.0)	40 (36.7)	29 (26.6)	29 (26.6)	43 (39.4)	8 (7.3)	94 (86.2)	14 (12.8)
Zambia	265	163 (61.5)	44.00 [33.00, 59.00]	23.07 [20.57, 27.20]	40 (15.1)	11 (4.2)	130.67 [119.67 , 145.33]	81.00 [74.00, 92.00]	7.60 [7.20, 8.40]	7.10 [6.60, 8.50]	140.76 [109.05 , 183.68]	35.00 [27.00, 46.40]	76.00 [58.40, 103.45]	265 (100.0)	31 (11.7)	20 (7.5)	20 (7.5)	10 (3.8)	134 (50.6)	13 (4.9)	109 (41.1)	50 (18.9)	23 (8.7)	23 (8.7)	100 (37.7)	3 (1.1)	160 (60.4)	4 (1.5)
Zanzibar	94	61 (64.9)	49.50 [43.00, 55.00]	24.81 [22.61, 29.68]	6 (6.4)	14 (14.9)	150.25 [131.38 , 176.50]	85.00 [79.12, 93.88]	8.80 [7.48, 11.96]	7.70 [6.60, 9.07]	190.45 [169.86 , 227.57]	50.27 [38.28, 61.23]	106.49 [90.83, 140.92]	94 (100.0)	32 (34.0)	12 (12.8)	12 (12.8)	7 (7.4)	41 (43.6)	8 (8.5)	62 (66.0)	36 (38.3)	12 (12.8)	12 (12.8)	19 (20.2)	0 (0.0)	77 (81.9)	1 (1.1)

Appendix Table 3: **Risk Equations for Complications of type 2 Diabetes (RECODe).**[2,3] The 10-year risk of an outcome can be computed as $1 - \lambda^{\exp(\Sigma(\beta \times x) - \text{mean}(\Sigma(\beta \times x)))}$, where β are the equation coefficients and x are the values for each covariate for an individual patient within the cohort under study. λ values are: 0.973 for renal failure, 0.921 for vision loss, 0.870 for pressure sensation loss, and 0.960 for heart failure, and corresponding values of mean(Σ(β×x)) are 0.23 for renal failure, 4.56 for vision loss, 4.75 for pressure sensation loss, and 5.15 for heart failure.

	**Renal failure/end-stage renal disease**	**Severe vision loss**	**Pressure sensation loss**	**Heart failure**
**Demographics**				
Age, years	-0.019380	0.022850	0.03022	0.052680
Women	-0.011290	0.226400	-0.18680	0.252900
Black	-0.088120	-0.167700	-0.09448	-0.049690
**Clinical features**				
Tobacco smoking, current	0.148300			0.290500
Systolic blood pressure, mm Hg	0.003027	0.008243	0.00456	0.001217
Cardiovascular disease history	-0.021640	0.112700	0.26672	1.007000
**Drug use**				
Blood pressure- lowering drugs	-0.079520	0.063930	0.18192	0.638900
Oral diabetes drugs	-0.125600	-0.234900	-0.25747	-0.117500
Anticoagulants	0.031990			0.736500
**Biomarkers**				

HbA1c, %	0.136900	0.144900	0.18866	0.209200
Total cholesterol, mg/dL	-0.001112	-0.000168	0.00219	-0.001358
HDL cholesterol, mg/dL	0.006289	0.005447	-0.00539	-0.017580
Serum creatinine, mg/dL	0.860900	0.694700	0.60442	0.821400
Urine albumin:creatinine ratio, mg/g	0.000362	0.000199		0.000414

Appendix Table 4: Disutilities used to computed disability-adjusted life-years, which were elicited through international standardized preference elicitation surveys.[13] The method of calculating DALYs from disutilities is specified by Rushby and Hanson.[14]

**Disease event**	**Estimated disutility on scale of 0 to 1 (95% CI)**
Atherosclerotic cardiovascular disease	0.28 (0.06, 0.57)
Congestive heart failure	0.10 (0.04, 0.19)
Renal failure/end-stage renal disease	0.34 (0.11, 0.57)
Retinopathy	0.20 (0.10, 0.40)
Neuropathy	0.10 (0.05, 0.20)

Appendix Table 5: Care components used in the microsimulation model. OP is outpatient; HbA1C is glycosylated haemoglobin; IU is international units; IP is inpatient; GTN is glyceryl tri-nitrate. These are based on recommendations in WHO PEN, other national or international guidelines, or the authors' clinical knowledge.[16–19] The authors' clinical knowledge was based on a conservative approach in acknowledgement of the availability of resources in many low or middle income countries. Medication costs for individual countries were extracted from the United Nations, Management Sciences for Health, and International Dispensary Association database. Other costs were extracted from the WHO One Health Tool (OHT), where available. Costs for blood tests are based on the only blood test cost in the OHT, which was full blood count. Additionally, it was not possible to extract costs that included capital expenditure for equipment, thus costs of (for example) X rays and echocardiography do not include costs of purchase, or of wear and tear-on the equipment. When major treatment costs were not available in WHO OHT, a literature search was done to identify systematic reviews from which costs in LMICs could be extracted. Major treatment costs not included in the WHO OHT were: (1) costs of practitioners; (2) costs of photocoagulation; (3) costs of surgery for peripheral vascular or diabetic foot diseases; (4) costs of CT scan to investigate stroke; and (5) costs of haemo or peripheral dialysis. We found one systematic review of these costs of dialysis in low- and middle-income countries in the literature.[20] From this review, we extracted costs of dialysis for low, lower middle, or upper middle income countries where the authors were certain of the provider perspective being taken.

**Reason**		**Component**	**Frequency/dose**
**Screening**	**Diabetes**	Random capillary glucose screening using point-of-care test	Every 3-5 years starting age 40
		Confirmatory testing	If random glucose $\geq$6.1 mmol/L, fasting plasma glucose, then fasting plasma glucose or hemoglobin A1c
	**Hypertension**	Upper arm sphygmomanometry	Annual

	**Hyperlipidemia**	Lipid panel with direct LDL	Every 5 years starting age 35 in men and 45 in women
**Diabetes management**		OPA visit at secondary hospital for specialist care to check for complications	annual
		OP visit at primary hospital for regular follow up	4x per year
		Urine glucose testing	3x per week
		Urine protein testing	4x per year
		HbA1c	2x per year
		Urea and Electrolytes	annual
		Treatment and order:	
		1st Metformin	start 500mg per day and increase to 850mg three times per day until controlled
		2nd Glibenclamide	start at 2.5mg and increase to 7.5mg twice per day
		3rd basal long acting Insulin	start at 8IU and increase to 20IU
		Simvastatin if ≥ 40 years	10mg once per day
**Hypertension management if BP≥130/80mmHg**		1st Enalapril	start at 10mg per day and increase to 20mg per day
		2nd Amlodipine	5mg per day and increase to 10mg per day
		3rd Atenolol	50mg per day
	**Hypoglycaemia**	IP days at secondary hospital	two days

**Complications management**		Glucose infusion	X2
		Blood glucose tests (total)	x8
		Urea and Electrolytes	x1
	**Renal disease**	1. Microalbuminuria	
		Enalapril	start at 10mg per day and increase to 20mg per day
		2. End stage renal failure	
		OP visit at tertiary hospital	4x per year
		Dialysis	As per systematic review for healthcare
		Urea and Electrolytes	12x per year
		Full blood count	4x per year
		Bone function tests	4x per year
		Liver function tests	4x per year
	**Retinopathy and blindness**	OP visit at secondary hospital	2x per year
		OP visit at tertiary hospital	3x after diagnosis made for photocoagulation
	**Foot disease/PVD**	IP days at tertiary hospital	7 days
		OP visit at secondary hospital	2 per year
	**MI**	IP days at tertiary hospital	5 days

		Investigations whilst inpatient:	
		Urea and Electrolytes	x5
		Full blood count	x5
		ECG	x5
		Echocardiography	x2
		Chest X ray	x2
		Acute treatment/medication:	
		Streptokinase	1.5MUnits x1
		Oxygen	3 days
		Long term management:	
		Aspirin	150mg per day
		Clopidogrel	600mg loading dose
		Clopidogrel	75mg per day for one year
		Enalapril	20mg per day
		Specialist OP visit at secondary hospital	2x per year
	**Congestive cardiac failure**	OP visit at secondary hospital	2x per year
		Echocardiography	annual

		Enalapril	20mg per day
		Furosemide	start at 20mg per day and increase to 160mg per day
	**Angina**	OP visit at secondary hospital	2x per year
		Echocardiography	annual
		Treatment/medication:	
		GTN spray	x3 per year
		Aspirin	150mg per day
		Enalapril	20mg per day
		Atenolol then:	start at 50mg per day and increase to 100mg per day
		Amplodipine then:	10mg per day
		Isosorbide mononitrate	10mg per day
	**Stroke**	IP days at tertiary hospital	14 days
		Investigations whilst inpatient:	
		Urea and Electrolytes	x5
		Full blood count	x5
		Echocardiography	x2
		Long term treatment:	

		OP visit at secondary hospital	2x per year
		Aspirin	150mg per day

Appendix Table 6: Modeled **combined impact on DALYs and costs of increased hypertension and diabetes diagnosis to 60% or 80%; increased blood pressure, glycaemia, and statin treatment to 60% or 80%; and increased blood pressure and glucose control to 60% or 80%, on risk of diabetes complications.** The 'overall' column is population weighted. A spreadsheet version can be downloaded at: https://docs.google.com/spreadsheets/d/1j2ETzKfB3V5a_jfF-_6nBJosHqJyzx4C5uAWt8f9tv8/edit?usp=sharing

**DALYs**					Region															
Diagnosis	Treatment	Control	Condition	Overall	Andean Latin America	Caribbean	Central Asia	Central Europe	Central Latin America	East Asia	Eastern Europe	East Sub-Saharan Africa	North Africa/ Middle East	Oceania	South Asia	Southeast Asia	South Latin America	South Sub-Saharan Africa	West Sub-Saharan Africa
Baseline	Baseline	Baseline	Cardiovascular events	143 [133, 155]	95 [91, 98]	130 [122, 138]	246 [210, 270]	243 [238, 248]	107 [102, 126]	220 [217, 223]	296 [273, 321]	114 [96, 132]	242 [233, 274]	147 [130, 176]	129 [126, 132]	135 [129, 145]	170 [168, 173]	133 [115, 158]	90 [80, 104]
Baseline	60%	60%	Cardiovascular events	124 [116, 134]	82 [79, 85]	98 [90, 104]	185 [177, 204]	178 [173, 182]	87 [83, 95]	194 [192, 197]	209 [198, 224]	96 [86, 107]	186 [180, 211]	134 [121, 148]	110 [106, 114]	115 [105, 121]	135 [133, 138]	106 [93, 127]	85 [76, 98]
Baseline	60%	80%	Cardiovascular events	124 [116, 134]	82 [79, 85]	98 [90, 104]	185 [177, 204]	178 [173, 182]	87 [83, 95]	194 [192, 197]	209 [198, 224]	96 [86, 107]	186 [180, 211]	134 [121, 148]	110 [106, 114]	115 [105, 121]	135 [133, 138]	106 [93, 127]	85 [76, 98]
Baseline	80%	60%	Cardiovascular events	118 [111, 129]	78 [75, 81]	90 [82, 96]	177 [166, 190]	161 [157, 166]	81 [78, 88]	188 [185, 191]	192 [183, 204]	93 [83, 101]	173 [166, 194]	130 [118, 142]	105 [101, 110]	109 [100, 117]	127 [124, 129]	101 [88, 120]	84 [75, 97]
Baseline	80%	80%	Cardiovascular events	117 [109, 127]	78 [74, 80]	87 [79, 94]	173 [160, 183]	156 [152, 159]	80 [77, 85]	186 [183, 189]	181 [173, 192]	91 [82, 99]	168 [161, 188]	129 [117, 140]	104 [99, 109]	107 [98, 115]	123 [121, 126]	98 [86, 116]	83 [75, 96]
Baseline	Baseline	Baseline	Heart failure	86 [80, 94]	79 [75, 83]	86 [79, 94]	100 [90, 117]	45 [42, 47]	108 [96, 131]	65 [63, 67]	99 [95, 102]	71 [62, 79]	90 [84, 101]	86 [64, 106]	89 [68, 105]	81 [69, 90]	99 [97, 102]	81 [71, 93]	60 [52, 72]
Baseline	60%	60%	Heart failure	88 [82, 95]	79 [75, 83]	86 [79, 94]	98 [88, 114]	45 [42, 47]	107 [95, 125]	65 [63, 67]	94 [90, 97]	71 [62, 79]	90 [84, 100]	86 [64, 106]	89 [68, 105]	81 [69, 90]	97 [95, 100]	81 [71, 93]	60 [52, 72]
Baseline	60%	80%	Heart failure	88 [82, 95]	79 [75, 83]	86 [79, 94]	98 [88, 114]	45 [42, 47]	107 [95, 125]	65 [63, 67]	94 [90, 97]	71 [62, 79]	90 [84, 100]	86 [64, 106]	89 [68, 105]	81 [69, 90]	97 [95, 100]	81 [71, 93]	60 [52, 72]
Baseline	80%	60%	Heart failure	87 [81, 95]	78 [74, 82]	84 [78, 92]	96 [87, 111]	45 [42, 47]	105 [94, 122]	64 [62, 66]	94 [90, 97]	71 [62, 78]	89 [83, 99]	85 [64, 105]	89 [68, 104]	80 [69, 90]	96 [94, 99]	80 [70, 91]	60 [52, 72]

Baseline	80%	80%	Heart failure	87 [82, 95]	78 [74, 82]	84 [78, 92]	96 [87, 111]	45 [42, 47]	105 [94, 122]	64 [62, 66]	94 [90, 97]	71 [62, 78]	89 [83, 99]	85 [64, 105]	89 [68, 104]	80 [69, 90]	96 [94, 99]	80 [70, 91]	60 [52, 72]
Baseline	Baseline	Baseline	ESRD	787 [734, 857]	811 [787, 833]	509 [452, 660]	612 [540, 907]	391 [373, 410]	678 [422, 722]	412 [401, 423]	484 [442, 530]	891 [775, 1200]	748 [491, 929]	1000 [849, 1096]	1057 [1031, 1081]	618 [569, 875]	528 [516, 541]	687 [496, 849]	1226 [1076, 1356]
Baseline	60%	60%	ESRD	801 [749, 870]	811 [787, 833]	509 [452, 660]	612 [540, 907]	391 [373, 410]	678 [422, 722]	412 [401, 423]	484 [442, 530]	891 [775, 1200]	748 [491, 929]	1000 [849, 1096]	1057 [1031, 1081]	618 [569, 875]	528 [516, 541]	687 [496, 849]	1226 [1076, 1356]
Baseline	60%	80%	ESRD	802 [750, 871]	811 [787, 833]	509 [452, 660]	612 [540, 907]	391 [373, 410]	678 [422, 722]	412 [401, 423]	484 [442, 530]	891 [775, 1200]	748 [491, 929]	1000 [849, 1096]	1057 [1031, 1081]	618 [569, 875]	528 [516, 541]	687 [496, 849]	1226 [1076, 1356]
Baseline	80%	60%	ESRD	806 [754, 876]	811 [787, 833]	508 [452, 658]	611 [539, 907]	391 [373, 410]	671 [422, 714]	412 [401, 423]	484 [442, 530]	891 [775, 1199]	746 [491, 927]	999 [845, 1096]	1056 [1031, 1081]	618 [569, 875]	528 [515, 541]	687 [495, 848]	1223 [1076, 1354]
Baseline	80%	80%	ESRD	809 [756, 877]	811 [787, 833]	508 [452, 658]	611 [539, 907]	391 [373, 410]	666 [422, 711]	412 [401, 423]	484 [442, 530]	891 [775, 1199]	745 [491, 924]	999 [845, 1096]	1056 [1031, 1081]	618 [569, 875]	528 [515, 541]	687 [495, 848]	1223 [1076, 1354]
Baseline	Baseline	Baseline	Neuropathy	81 [76, 88]	75 [72, 77]	64 [57, 83]	81 [70, 90]	55 [53, 57]	81 [61, 83]	63 [61, 64]	70 [67, 73]	81 [74, 88]	77 [65, 93]	92 [85, 97]	99 [86, 109]	76 [68, 83]	71 [70, 73]	67 [62, 70]	90 [77, 98]
Baseline	60%	60%	Neuropathy	83 [77, 90]	75 [72, 77]	64 [57, 82]	80 [70, 90]	55 [53, 57]	81 [61, 83]	63 [61, 64]	70 [67, 73]	81 [74, 88]	77 [65, 93]	92 [85, 97]	99 [86, 109]	76 [68, 83]	70 [69, 72]	67 [62, 70]	90 [77, 98]
Baseline	60%	80%	Neuropathy	83 [77, 90]	75 [72, 77]	64 [57, 82]	80 [70, 90]	55 [53, 57]	81 [61, 83]	63 [61, 64]	70 [67, 73]	81 [74, 88]	77 [65, 93]	92 [85, 97]	99 [86, 109]	76 [68, 83]	70 [69, 72]	67 [62, 70]	90 [77, 98]
Baseline	80%	60%	Neuropathy	83 [77, 90]	74 [72, 77]	63 [56, 82]	79 [69, 89]	55 [53, 56]	80 [59, 82]	62 [61, 64]	69 [66, 72]	81 [74, 88]	76 [64, 93]	91 [85, 97]	98 [86, 108]	75 [68, 83]	69 [68, 70]	66 [62, 70]	90 [76, 98]
Baseline	80%	80%	Neuropathy	82 [77, 89]	74 [72, 77]	63 [56, 81]	78 [69, 88]	54 [52, 56]	79 [58, 80]	62 [61, 63]	68 [66, 71]	80 [74, 88]	76 [64, 92]	91 [85, 97]	98 [86, 108]	75 [67, 82]	68 [67, 69]	66 [62, 70]	90 [76, 98]
Baseline	Baseline	Baseline	Vision loss	64 [60, 70]	54 [52, 56]	49 [45, 57]	63 [54, 70]	42 [40, 44]	60 [40, 63]	44 [43, 46]	57 [54, 60]	64 [57, 72]	62 [48, 72]	70 [65, 74]	75 [72, 78]	59 [53, 67]	50 [49, 52]	53 [50, 60]	70 [61, 81]
Baseline	60%	60%	Vision loss	65 [61, 71]	54 [52, 56]	49 [45, 57]	63 [54, 70]	42 [40, 44]	60 [40, 63]	44 [43, 46]	57 [54, 60]	64 [57, 72]	62 [48, 72]	70 [65, 74]	75 [72, 78]	59 [53, 67]	50 [49, 52]	53 [50, 60]	70 [61, 81]
Baseline	60%	80%	Vision loss	65 [61, 71]	54 [52, 56]	49 [45, 57]	63 [54, 70]	42 [40, 44]	60 [40, 63]	44 [43, 46]	57 [54, 60]	64 [57, 72]	62 [48, 72]	70 [65, 74]	75 [72, 78]	59 [53, 67]	50 [49, 52]	53 [50, 60]	70 [61, 81]

Baseline	80%	60%	Vision loss	66 [62, 72]	54 [52, 56]	49 [45, 57]	62 [54, 70]	42 [40, 44]	60 [40, 62]	44 [43, 46]	57 [54, 60]	64 [57, 71]	62 [48, 72]	70 [65, 74]	75 [72, 78]	59 [53, 67]	50 [49, 51]	53 [50, 59]	70 [61, 81]
Baseline	80%	80%	Vision loss	66 [62, 72]	54 [52, 56]	49 [45, 57]	62 [54, 70]	42 [40, 44]	60 [40, 62]	44 [43, 46]	57 [54, 60]	64 [57, 71]	62 [48, 72]	70 [65, 74]	75 [72, 78]	59 [53, 67]	50 [49, 51]	53 [50, 59]	70 [61, 81]
60%	Baseline	Baseline	Cardiovascular events	143 [133, 156]	95 [91, 98]	130 [122, 138]	246 [210, 270]	243 [238, 248]	107 [102, 126]	220 [217, 223]	296 [273, 321]	114 [96, 132]	242 [233, 274]	147 [130, 176]	129 [126, 132]	135 [129, 145]	170 [168, 173]	133 [115, 158]	90 [80, 104]
60%	60%	60%	Cardiovascular events	117 [110, 127]	80 [77, 83]	98 [90, 104]	180 [166, 201]	178 [173, 182]	87 [83, 95]	183 [181, 186]	208 [194, 224]	90 [77, 103]	185 [179, 210]	120 [105, 141]	105 [101, 108]	109 [101, 117]	135 [133, 138]	103 [89, 122]	71 [63, 81]
60%	60%	80%	Cardiovascular events	117 [110, 127]	80 [77, 83]	98 [90, 104]	180 [166, 201]	178 [173, 182]	87 [83, 95]	183 [181, 186]	208 [194, 224]	90 [77, 103]	185 [179, 210]	120 [105, 141]	105 [101, 108]	109 [101, 117]	135 [133, 138]	103 [89, 122]	71 [63, 81]
60%	80%	60%	Cardiovascular events	111 [104, 120]	76 [73, 79]	90 [82, 96]	166 [155, 186]	161 [157, 166]	81 [78, 88]	174 [171, 177]	189 [178, 204]	84 [72, 97]	171 [165, 194]	112 [99, 133]	98 [95, 101]	103 [94, 111]	127 [124, 129]	96 [83, 114]	67 [58, 76]
60%	80%	80%	Cardiovascular events	108 [101, 117]	76 [72, 78]	87 [79, 94]	158 [149, 178]	156 [152, 159]	80 [77, 85]	171 [168, 174]	178 [168, 192]	81 [70, 94]	166 [160, 188]	110 [97, 130]	96 [93, 99]	100 [91, 108]	123 [121, 126]	92 [81, 109]	65 [56, 74]
60%	Baseline	Baseline	Heart failure	86 [80, 93]	79 [75, 83]	86 [79, 94]	100 [90, 117]	45 [42, 47]	108 [96, 131]	65 [63, 67]	99 [95, 102]	71 [62, 79]	90 [84, 101]	86 [64, 106]	89 [68, 105]	81 [69, 90]	99 [97, 102]	81 [71, 93]	60 [52, 72]
60%	60%	60%	Heart failure	88 [82, 95]	79 [75, 83]	86 [79, 94]	98 [88, 114]	45 [42, 47]	107 [95, 125]	65 [63, 67]	94 [90, 97]	71 [62, 79]	90 [84, 100]	86 [64, 106]	89 [68, 105]	81 [69, 90]	97 [95, 100]	81 [71, 93]	60 [52, 72]
60%	60%	80%	Heart failure	88 [82, 95]	79 [75, 83]	86 [79, 94]	98 [88, 114]	45 [42, 47]	107 [95, 125]	65 [63, 67]	94 [90, 97]	71 [62, 79]	90 [84, 100]	86 [64, 106]	89 [68, 105]	81 [69, 90]	97 [95, 100]	81 [71, 93]	60 [52, 72]
60%	80%	60%	Heart failure	87 [82, 95]	78 [74, 82]	84 [78, 92]	96 [87, 111]	45 [42, 47]	105 [94, 122]	64 [62, 66]	94 [90, 97]	70 [62, 78]	89 [83, 99]	85 [64, 105]	89 [68, 104]	80 [69, 90]	96 [94, 99]	80 [70, 91]	60 [52, 72]
60%	80%	80%	Heart failure	88 [82, 95]	78 [74, 82]	84 [78, 92]	96 [87, 111]	45 [42, 47]	105 [94, 122]	64 [62, 66]	94 [90, 97]	70 [62, 78]	89 [83, 99]	85 [64, 105]	89 [68, 104]	80 [69, 90]	96 [94, 99]	80 [70, 91]	60 [52, 72]
60%	Baseline	Baseline	ESRD	787 [734, 857]	811 [787, 833]	509 [452, 660]	612 [540, 907]	391 [373, 410]	678 [422, 722]	412 [401, 423]	484 [442, 530]	891 [775, 1200]	748 [491, 929]	1000 [849, 1096]	1057 [1031, 1081]	618 [569, 875]	528 [516, 541]	687 [496, 849]	1226 [1076, 1356]
60%	60%	60%	ESRD	807 [755, 876]	811 [787, 833]	509 [452, 660]	612 [540, 907]	391 [373, 410]	678 [422, 722]	412 [401, 423]	484 [442, 530]	891 [775, 1200]	748 [491, 929]	1000 [849, 1096]	1057 [1031, 1081]	618 [569, 875]	528 [516, 541]	687 [496, 849]	1226 [1076, 1356]
60%	60%	80%	ESRD	807 [755, 877]	811 [787, 833]	509 [452, 660]	612 [540, 907]	391 [373, 410]	678 [422, 722]	412 [401, 423]	484 [442, 530]	891 [775, 1200]	748 [491, 929]	1000 [849, 1096]	1057 [1031, 1081]	618 [569, 875]	528 [516, 541]	687 [496, 849]	1226 [1076, 1356]

60%	80%	60%	ESRD	813 [760, 883]	811 [787, 832]	508 [452, 658]	611 [539, 906]	391 [373, 410]	671 [422, 714]	412 [401, 423]	484 [442, 530]	889 [774, 1198]	746 [491, 927]	997 [845, 1095]	1053 [1027, 1078]	618 [569, 867]	528 [515, 541]	687 [495, 848]	1221 [1074, 1349]
60%	80%	80%	ESRD	815 [762, 884]	810 [786, 832]	508 [452, 658]	611 [539, 906]	391 [373, 410]	666 [422, 711]	412 [401, 423]	483 [442, 529]	886 [773, 1196]	745 [491, 924]	997 [845, 1094]	1050 [1025, 1077]	618 [569, 867]	528 [515, 541]	687 [495, 848]	1220 [1074, 1346]
60%	Baseline	Baseline	Neuropathy	82 [76, 89]	75 [72, 77]	64 [57, 83]	81 [70, 90]	55 [53, 57]	81 [61, 83]	63 [61, 64]	70 [67, 73]	81 [74, 88]	77 [65, 93]	92 [85, 97]	99 [86, 109]	76 [68, 83]	71 [70, 73]	67 [62, 70]	90 [77, 98]
60%	60%	60%	Neuropathy	84 [78, 91]	75 [72, 77]	64 [57, 82]	80 [70, 90]	55 [53, 57]	81 [61, 83]	62 [61, 64]	70 [67, 73]	81 [74, 88]	77 [65, 93]	92 [85, 97]	99 [86, 109]	76 [68, 83]	70 [69, 72]	67 [62, 70]	90 [77, 98]
60%	60%	80%	Neuropathy	84 [78, 91]	75 [72, 77]	64 [57, 82]	80 [70, 90]	55 [53, 57]	81 [61, 83]	62 [61, 64]	70 [67, 73]	81 [74, 88]	77 [65, 93]	92 [85, 97]	99 [86, 109]	76 [68, 83]	70 [69, 72]	67 [62, 70]	90 [77, 98]
60%	80%	60%	Neuropathy	84 [79, 92]	74 [72, 77]	63 [56, 82]	79 [69, 89]	55 [53, 56]	80 [59, 82]	62 [61, 63]	69 [66, 71]	80 [74, 88]	76 [64, 92]	91 [85, 97]	98 [86, 108]	75 [68, 82]	69 [68, 70]	66 [62, 70]	90 [76, 98]
60%	80%	80%	Neuropathy	84 [79, 91]	74 [72, 77]	63 [56, 81]	78 [69, 88]	54 [52, 56]	79 [58, 80]	62 [60, 63]	68 [66, 71]	80 [74, 87]	76 [64, 92]	91 [85, 97]	98 [85, 108]	75 [67, 82]	68 [67, 69]	66 [62, 70]	90 [76, 98]
60%	Baseline	Baseline	Vision loss	64 [59, 69]	54 [52, 56]	49 [45, 57]	63 [54, 70]	42 [40, 44]	60 [40, 63]	44 [43, 46]	57 [54, 60]	64 [57, 72]	62 [48, 72]	70 [65, 74]	75 [72, 78]	59 [53, 67]	50 [49, 52]	53 [50, 60]	70 [61, 81]
60%	60%	60%	Vision loss	65 [61, 71]	54 [52, 56]	49 [45, 57]	63 [54, 70]	42 [40, 44]	60 [40, 63]	44 [43, 46]	57 [54, 60]	64 [57, 72]	62 [48, 72]	70 [65, 74]	75 [72, 78]	59 [53, 67]	50 [49, 52]	53 [50, 60]	70 [61, 81]
60%	60%	80%	Vision loss	65 [61, 71]	54 [52, 56]	49 [45, 57]	63 [54, 70]	42 [40, 44]	60 [40, 63]	44 [43, 46]	57 [54, 60]	64 [57, 72]	62 [48, 72]	70 [65, 74]	75 [72, 78]	59 [53, 67]	50 [49, 52]	53 [50, 60]	70 [61, 81]
60%	80%	60%	Vision loss	66 [61, 71]	54 [52, 56]	49 [45, 57]	62 [54, 70]	42 [40, 44]	60 [40, 62]	44 [43, 45]	57 [54, 60]	64 [57, 71]	62 [48, 72]	70 [65, 74]	75 [72, 78]	59 [53, 67]	50 [49, 51]	53 [50, 59]	70 [61, 81]
60%	80%	80%	Vision loss	66 [62, 71]	54 [52, 56]	49 [45, 57]	62 [54, 70]	42 [40, 44]	60 [40, 62]	44 [43, 45]	57 [54, 60]	64 [57, 71]	62 [48, 72]	70 [65, 74]	75 [72, 78]	59 [53, 67]	50 [49, 51]	53 [50, 59]	70 [61, 81]
80%	Baseline	Baseline	Cardiovascular events	143 [134, 156]	95 [91, 98]	130 [122, 138]	246 [210, 270]	243 [238, 248]	107 [102, 126]	220 [217, 223]	296 [273, 321]	114 [96, 132]	242 [233, 274]	147 [130, 176]	129 [126, 132]	135 [129, 145]	170 [168, 173]	133 [115, 158]	90 [80, 104]
80%	60%	60%	Cardiovascular events	111 [103, 120]	78 [74, 81]	96 [89, 102]	171 [158, 192]	177 [173, 181]	86 [83, 93]	175 [172, 178]	201 [184, 219]	85 [72, 99]	181 [173, 204]	112 [100, 134]	100 [97, 103]	104 [96, 113]	130 [127, 132]	98 [84, 115]	66 [58, 76]
80%	60%	80%	Cardiovascular events	111 [103, 120]	78 [74, 81]	96 [89, 102]	171 [158, 192]	177 [173, 181]	86 [83, 93]	175 [172, 178]	201 [184, 219]	85 [72, 99]	181 [173, 204]	112 [100, 134]	100 [97, 103]	104 [96, 113]	130 [127, 132]	98 [84, 115]	66 [58, 76]

80%	80%	60%	Cardiovascular events	103 [96, 112]	73 [70, 76]	88 [81, 94]	156 [145, 174]	161 [157, 165]	80 [77, 85]	164 [161, 166]	181 [167, 198]	78 [66, 91]	166 [159, 187]	103 [92, 124]	93 [89, 96]	97 [88, 105]	120 [118, 123]	90 [77, 105]	61 [52, 69]
80%	80%	80%	Cardiovascular events	100 [93, 108]	72 [69, 75]	85 [77, 91]	147 [139, 166]	155 [151, 159]	79 [76, 82]	160 [157, 163]	169 [155, 186]	75 [64, 87]	161 [153, 179]	101 [90, 120]	90 [87, 94]	94 [85, 102]	116 [114, 119]	86 [74, 100]	58 [50, 67]
80%	Baseline	Baseline	Heart failure	85 [79, 93]	79 [75, 83]	86 [79, 94]	100 [90, 117]	45 [42, 47]	108 [96, 131]	65 [63, 67]	99 [95, 102]	71 [62, 79]	90 [84, 101]	86 [64, 106]	89 [68, 105]	81 [69, 90]	99 [97, 102]	81 [71, 93]	60 [52, 72]
80%	60%	60%	Heart failure	88 [82, 95]	79 [75, 83]	86 [79, 94]	98 [88, 114]	45 [42, 47]	106 [95, 125]	65 [63, 67]	94 [90, 97]	71 [62, 79]	90 [84, 100]	86 [64, 106]	89 [68, 105]	81 [69, 90]	97 [95, 100]	81 [71, 93]	60 [52, 72]
80%	60%	80%	Heart failure	88 [82, 95]	79 [75, 83]	86 [79, 94]	98 [88, 114]	45 [42, 47]	106 [95, 125]	65 [63, 67]	94 [90, 97]	71 [62, 79]	90 [84, 100]	86 [64, 106]	89 [68, 105]	81 [69, 90]	97 [95, 100]	81 [71, 93]	60 [52, 72]
80%	80%	60%	Heart failure	88 [82, 95]	78 [74, 82]	84 [78, 92]	96 [87, 111]	45 [42, 47]	105 [94, 122]	64 [62, 66]	93 [90, 97]	70 [62, 78]	89 [83, 99]	85 [64, 105]	89 [68, 104]	80 [69, 90]	96 [93, 98]	80 [70, 91]	60 [52, 72]
80%	80%	80%	Heart failure	88 [82, 95]	78 [74, 82]	84 [78, 92]	96 [87, 111]	45 [42, 47]	105 [94, 122]	64 [62, 66]	93 [90, 97]	70 [62, 78]	89 [83, 99]	85 [64, 105]	89 [68, 104]	80 [69, 90]	96 [93, 98]	80 [70, 91]	60 [52, 72]
80%	Baseline	Baseline	ESRD	787 [734, 857]	811 [787, 833]	509 [452, 660]	612 [540, 907]	391 [373, 410]	678 [422, 722]	412 [401, 423]	484 [442, 530]	891 [775, 1200]	748 [491, 929]	1000 [849, 1096]	1057 [1031, 1081]	618 [569, 875]	528 [516, 541]	687 [496, 849]	1226 [1076, 1356]
80%	60%	60%	ESRD	812 [760, 882]	811 [787, 833]	509 [452, 660]	612 [540, 907]	391 [373, 410]	678 [422, 722]	412 [401, 423]	484 [442, 530]	891 [775, 1200]	748 [491, 929]	1000 [849, 1096]	1057 [1031, 1081]	618 [569, 875]	528 [516, 541]	687 [496, 849]	1226 [1076, 1356]
80%	60%	80%	ESRD	812 [760, 882]	811 [787, 833]	509 [452, 660]	612 [540, 907]	391 [373, 410]	678 [422, 722]	412 [401, 423]	484 [442, 530]	891 [775, 1200]	748 [491, 929]	1000 [849, 1096]	1057 [1031, 1081]	618 [569, 875]	528 [516, 541]	687 [496, 849]	1226 [1076, 1356]
80%	80%	60%	ESRD	819 [766, 890]	810 [786, 832]	508 [452, 658]	611 [539, 906]	391 [373, 410]	670 [422, 713]	412 [401, 423]	483 [442, 529]	886 [773, 1198]	745 [490, 925]	997 [845, 1094]	1051 [1025, 1077]	617 [569, 867]	527 [514, 540]	687 [495, 848]	1221 [1074, 1348]
80%	80%	80%	ESRD	822 [769, 892]	809 [784, 830]	508 [452, 658]	611 [539, 906]	391 [373, 410]	664 [422, 707]	412 [401, 423]	483 [442, 529]	886 [772, 1196]	745 [490, 920]	997 [845, 1094]	1050 [1024, 1075]	617 [569, 867]	523 [511, 537]	687 [495, 848]	1220 [1074, 1345]
80%	Baseline	Baseline	Neuropathy	83 [77, 90]	75 [72, 77]	64 [57, 83]	81 [70, 90]	55 [53, 57]	81 [61, 83]	63 [61, 64]	70 [67, 73]	81 [74, 88]	77 [65, 93]	92 [85, 97]	99 [86, 109]	76 [68, 83]	71 [70, 73]	67 [62, 70]	90 [77, 98]
80%	60%	60%	Neuropathy	85 [80, 92]	75 [72, 77]	64 [57, 82]	80 [70, 90]	55 [53, 57]	81 [60, 83]	62 [61, 64]	70 [67, 73]	81 [74, 88]	77 [65, 93]	92 [85, 97]	99 [86, 109]	76 [68, 83]	70 [69, 72]	67 [62, 70]	90 [77, 98]

80%	60%	80%	Neuropathy	85 [80, 92]	75 [72, 77]	64 [57, 82]	80 [70, 90]	55 [53, 57]	81 [60, 83]	62 [61, 64]	70 [67, 73]	81 [74, 88]	77 [65, 93]	92 [85, 97]	99 [86, 109]	76 [68, 83]	70 [69, 72]	67 [62, 70]	90 [77, 98]
80%	80%	60%	Neuropathy	86 [80, 93]	74 [72, 77]	63 [56, 82]	79 [69, 89]	55 [53, 56]	80 [59, 82]	62 [60, 63]	69 [66, 71]	80 [74, 88]	76 [64, 92]	91 [85, 97]	98 [86, 108]	75 [68, 82]	69 [67, 70]	66 [62, 70]	90 [76, 98]
80%	80%	80%	Neuropathy	86 [80, 93]	74 [72, 77]	63 [56, 80]	78 [68, 88]	54 [52, 56]	78 [58, 80]	61 [60, 63]	68 [66, 71]	80 [74, 87]	76 [64, 92]	91 [85, 97]	98 [85, 108]	75 [67, 82]	68 [66, 69]	66 [62, 70]	90 [76, 98]
80%	Baseline	Baseline	Vision loss	63 [59, 69]	54 [52, 56]	49 [45, 57]	63 [54, 70]	42 [40, 44]	60 [40, 63]	44 [43, 46]	57 [54, 60]	64 [57, 72]	62 [48, 72]	70 [65, 74]	75 [72, 78]	59 [53, 67]	50 [49, 52]	53 [50, 60]	70 [61, 81]
80%	60%	60%	Vision loss	65 [61, 71]	54 [52, 56]	49 [45, 57]	63 [54, 70]	42 [40, 44]	60 [40, 63]	44 [43, 46]	57 [54, 60]	64 [57, 72]	62 [48, 72]	70 [65, 74]	75 [72, 78]	59 [53, 67]	50 [49, 52]	53 [50, 60]	70 [61, 81]
80%	60%	80%	Vision loss	65 [61, 71]	54 [52, 56]	49 [45, 57]	63 [54, 70]	42 [40, 44]	60 [40, 63]	44 [43, 46]	57 [54, 60]	64 [57, 72]	62 [48, 72]	70 [65, 74]	75 [72, 78]	59 [53, 67]	50 [49, 52]	53 [50, 60]	70 [61, 81]
80%	80%	60%	Vision loss	65 [61, 71]	54 [52, 56]	49 [45, 57]	62 [53, 70]	42 [40, 44]	60 [40, 62]	44 [43, 45]	56 [54, 60]	64 [57, 71]	62 [48, 72]	70 [65, 74]	75 [72, 78]	59 [53, 67]	50 [48, 51]	53 [50, 59]	70 [61, 81]
80%	80%	80%	Vision loss	66 [61, 71]	54 [52, 56]	49 [45, 57]	62 [53, 70]	42 [40, 44]	60 [40, 62]	44 [43, 45]	56 [54, 60]	64 [57, 71]	62 [48, 72]	70 [65, 74]	75 [72, 78]	59 [53, 67]	50 [48, 51]	53 [50, 59]	70 [61, 81]
**Costs**																			
Baseline	Baseline	Baseline	Screening	0 [0, 0]	0 [0, 0]	0 [0, 0]	0 [0, 0]	0 [0, 0]	0 [0, 0]	0 [0, 0]	0 [0, 0]	0 [0, 0]	0 [0, 0]	0 [0, 0]	0 [0, 0]	0 [0, 0]	0 [0, 0]	0 [0, 0]	0 [0, 0]
Baseline	60%	60%	Screening	0 [0, 0]	0 [0, 0]	0 [0, 0]	0 [0, 0]	0 [0, 0]	0 [0, 0]	0 [0, 0]	0 [0, 0]	0 [0, 0]	0 [0, 0]	0 [0, 0]	0 [0, 0]	0 [0, 0]	0 [0, 0]	0 [0, 0]	0 [0, 0]
Baseline	60%	80%	Screening	0 [0, 0]	0 [0, 0]	0 [0, 0]	0 [0, 0]	0 [0, 0]	0 [0, 0]	0 [0, 0]	0 [0, 0]	0 [0, 0]	0 [0, 0]	0 [0, 0]	0 [0, 0]	0 [0, 0]	0 [0, 0]	0 [0, 0]	0 [0, 0]
Baseline	80%	60%	Screening	0 [0, 0]	0 [0, 0]	0 [0, 0]	0 [0, 0]	0 [0, 0]	0 [0, 0]	0 [0, 0]	0 [0, 0]	0 [0, 0]	0 [0, 0]	0 [0, 0]	0 [0, 0]	0 [0, 0]	0 [0, 0]	0 [0, 0]	0 [0, 0]
Baseline	80%	80%	Screening	0 [0, 0]	0 [0, 0]	0 [0, 0]	0 [0, 0]	0 [0, 0]	0 [0, 0]	0 [0, 0]	0 [0, 0]	0 [0, 0]	0 [0, 0]	0 [0, 0]	0 [0, 0]	0 [0, 0]	0 [0, 0]	0 [0, 0]	0 [0, 0]
Baseline	Baseline	Baseline	Blood pressure medicines	10383 [7712, 31445]	27251 [27251, 27251]	20202 [7416, 113126]	8971 [6911, 14177]	0 [0, 0]	26924 [20985, 151042]	30277 [30277, 30277]	15416 [12765, 18066]	2553 [1538, 4196]	7302 [4344, 8858]	1483 [737, 8647]	2463 [1708, 3219]	9522 [5612, 19412]	20282 [20282, 20282]	11410 [10122, 13140]	1973 [810, 3156]
Baseline	60%	60%	Blood pressure medicines	43913 [35431, 88687]	74066 [73205, 74926]	63067 [16869, 247812]	27578 [26336, 53536]	47809 [47360, 48258]	59776 [58814, 232818]	103157 [102337, 103905]	40584 [35318, 46250]	14117 [8893, 20961]	22830 [18816, 30273]	6241 [2922, 45883]	9825 [7636, 11885]	39829 [21394, 75767]	61097 [60640, 61538]	26421 [23545, 29265]	6155 [3515, 15656]

Baseline	60%	80%	Blood pressure medicines	43913 [35431, 88687]	74066 [73205, 74926]	63067 [16869, 247812]	27578 [26336, 53536]	47809 [47360, 48258]	59776 [58814, 232818]	103157 [102337, 103905]	40584 [35318, 46250]	14117 [8893, 20961]	22830 [18816, 30273]	6241 [2922, 45883]	9825 [7636, 11885]	39829 [21394, 75767]	61097 [60640, 61538]	26421 [23545, 29265]	6155 [3515, 15656]
Baseline	80%	60%	Blood pressure medicines	48777 [39412, 98598]	82270 [81562, 82997]	70126 [18675, 276293]	30569 [29209, 59668]	53114 [52743, 53487]	66396 [65205, 259203]	114650 [113878, 115268]	45125 [39234, 51402]	15686 [9932, 23163]	25367 [20940, 33601]	6935 [3259, 50878]	10885 [8484, 13208]	44292 [24307, 83410]	67881 [67504, 68264]	29388 [26158, 32506]	6844 [3877, 17529]
Baseline	80%	80%	Blood pressure medicines	58550 [47352, 118332]	98678 [97990, 99424]	84172 [22347, 332456]	36616 [34981, 71718]	63745 [63382, 64088]	79668 [78161, 311413]	137530 [136854, 138145]	54321 [47110, 61690]	18836 [11961, 27655]	30421 [25155, 40268]	8324 [3916, 60949]	13031 [10181, 15851]	53394 [29811, 99011]	81475 [81095, 81820]	35646 [31372, 38985]	8207 [4616, 21148]
Baseline	Baseline	Baseline	Diabetes medicines	28462 [15229, 39315]	53571 [53571, 53571]	82390 [20605, 131214]	19477 [11090, 20815]	18460 [18460, 18460]	88212 [26054, 115877]	21471 [21471, 21471]	23901 [19389, 28414]	8718 [4136, 9808]	19984 [15935, 21689]	14076 [4717, 26878]	8687 [6491, 10884]	22081 [17781, 56564]	53533 [53533, 53533]	13185 [12401, 13762]	3419 [1404, 9300]
Baseline	60%	60%	Diabetes medicines	65420 [51561, 83194]	70275 [69642, 70945]	130757 [29609, 167298]	27822 [23776, 33264]	116699 [115701, 117796]	145293 [121712, 158411]	24021 [23829, 24253]	33154 [30805, 35702]	13600 [6978, 17610]	28894 [25236, 32557]	44543 [23196, 71254]	14277 [9608, 18902]	36142 [22511, 81344]	95021 [94374, 95607]	17883 [16716, 19482]	5539 [2987, 11105]
Baseline	60%	80%	Diabetes medicines	65420 [51561, 83194]	70275 [69642, 70945]	130757 [29609, 167298]	27822 [23776, 33264]	116699 [115701, 117796]	145293 [121712, 158411]	24021 [23829, 24253]	33154 [30805, 35702]	13600 [6978, 17610]	28894 [25236, 32557]	44543 [23196, 71254]	14277 [9608, 18902]	36142 [22511, 81344]	95021 [94374, 95607]	17883 [16716, 19482]	5539 [2987, 11105]
Baseline	80%	60%	Diabetes medicines	74168 [58458, 94338]	79672 [79129, 80245]	148159 [33442, 190215]	31529 [26932, 37859]	132285 [131445, 133203]	164705 [137837, 179823]	27226 [27065, 27426]	37550 [34915, 40467]	15372 [8074, 19913]	32745 [28697, 36911]	50588 [26420, 80605]	16133 [10891, 21425]	41207 [25636, 91467]	107668 [107132, 108182]	20279 [18951, 22080]	6277 [3387, 12727]
Baseline	80%	80%	Diabetes medicines	87288 [68773, 110908]	93787 [93191, 94382]	174382 [39262, 224270]	37092 [31661, 44642]	155665 [154817, 156463]	193734 [162105, 211760]	32055 [31863, 32226]	44175 [41078, 47650]	18085 [9594, 23351]	38518 [33790, 43432]	59590 [31135, 94750]	19006 [12811, 25213]	48415 [30165, 107605]	126695 [126182, 127163]	23805 [22275, 25957]	7385 [3972, 15044]

Baseline	Baseline	Baseline	Statins	2979 [1784, 9132]	10299 [10299, 10299]	2752 [2740, 18628]	1142 [854, 1515]	931 [931, 931]	8212 [5042, 42094]	1749 [1749, 1749]	2854 [2297, 3410]	301 [107, 1087]	3078 [1749, 4674]	397 [62, 3579]	861 [318, 1403]	4788 [1803, 9098]	4039 [4039, 4039]	1201 [593, 1858]	444 [0, 1350]
Baseline	60%	60%	Statins	14685 [11083, 36593]	54135 [53669, 54620]	21315 [18069, 118497]	6666 [5693, 7063]	12561 [12458, 12663]	23522 [20992, 115899]	11290 [11217, 11370]	11302 [9822, 12862]	5582 [2436, 7578]	8235 [6015, 14461]	1990 [977, 25174]	5297 [3820, 6729]	13878 [9846, 36587]	12305 [12224, 12386]	7314 [6332, 7747]	3635 [2133, 7547]
Baseline	60%	80%	Statins	14685 [11083, 36593]	54135 [53669, 54620]	21315 [18069, 118497]	6666 [5693, 7063]	12561 [12458, 12663]	23522 [20992, 115899]	11290 [11217, 11370]	11302 [9822, 12862]	5582 [2436, 7578]	8235 [6015, 14461]	1990 [977, 25174]	5297 [3820, 6729]	13878 [9846, 36587]	12305 [12224, 12386]	7314 [6332, 7747]	3635 [2133, 7547]
Baseline	80%	60%	Statins	19579 [14778, 48827]	72237 [71801, 72625]	28446 [23971, 158896]	8888 [7555, 9463]	16749 [16670, 16833]	31364 [27962, 154850]	15058 [14995, 15125]	15092 [13098, 17150]	7459 [3349, 10058]	10983 [8016, 19228]	2645 [1310, 33467]	7031 [5096, 8978]	18653 [13133, 48716]	16409 [16348, 16473]	9732 [8693, 10406]	4847 [2781, 10213]
Baseline	80%	80%	Statins	19579 [14778, 48827]	72237 [71801, 72625]	28446 [23971, 158896]	8888 [7555, 9463]	16749 [16670, 16833]	31364 [27962, 154850]	15058 [14995, 15125]	15092 [13098, 17150]	7459 [3349, 10058]	10983 [8016, 19228]	2645 [1310, 33467]	7031 [5096, 8978]	18653 [13133, 48716]	16409 [16348, 16473]	9732 [8693, 10406]	4847 [2781, 10213]
Baseline	Baseline	Baseline	Cardiovascular events	144469 [113718, 174699]	98405 [95714, 101288]	156038 [46437, 178629]	191734 [168474, 214715]	625009 [614462, 634638]	261087 [201619, 312560]	246503 [243494, 249178]	279274 [265322, 295326]	36681 [22172, 92644]	154517 [97487, 187204]	60877 [56258, 80349]	21588 [21088, 22054]	58906 [54244, 76702]	554756 [546712, 561389]	88743 [30433, 211586]	12004 [10177, 16660]
Baseline	60%	60%	Cardiovascular events	107506 [86504, 126246]	84182 [81492, 86489]	114121 [35669, 124086]	143253 [127363, 156501]	457178 [447548, 465890]	204546 [166584, 229900]	215826 [213067, 218521]	195635 [191261, 200165]	29712 [18075, 77640]	114555 [81827, 136551]	55594 [51365, 64172]	18145 [17584, 18783]	47125 [40694, 65982]	433672 [427040, 440023]	62190 [22430, 169696]	11123 [9806, 13766]
Baseline	60%	80%	Cardiovascular events	107506 [86504, 126246]	84182 [81492, 86489]	114121 [35669, 124086]	143253 [127363, 156501]	457178 [447548, 465890]	204546 [166584, 229900]	215826 [213067, 218521]	195635 [191261, 200165]	29712 [18075, 77640]	114555 [81827, 136551]	55594 [51365, 64172]	18145 [17584, 18783]	47125 [40694, 65982]	433672 [427040, 440023]	62190 [22430, 169696]	11123 [9806, 13766]

Baseline	80%	60%	Cardiovascular events	98520 [79707, 114820]	80146 [77456, 82453]	104464 [32809, 112146]	135540 [115784, 144128]	416367 [407654, 425079]	189441 [155961, 211351]	208303 [205461, 210978]	178456 [175121, 181909]	28245 [17232, 72815]	105041 [77935, 124624]	54361 [50010, 60636]	17312 [16617, 18080]	44298 [37323, 63306]	405307 [399097, 411516]	56487 [21002, 160789]	10903 [9691, 13378]
Baseline	80%	80%	Cardiovascular events	95347 [77372, 109196]	79378 [76687, 81492]	100149 [32136, 107317]	131051 [109469, 138644]	401234 [392407, 409488]	185699 [155057, 202332]	205712 [202953, 208470]	167601 [164458, 170913]	27500 [16750, 71718]	101844 [76577, 118438]	53777 [49620, 59324]	17042 [16288, 17813]	43355 [35933, 62300]	393452 [386678, 399662]	54109 [20227, 153583]	10792 [9655, 13131]
Baseline	Baseline	Baseline	Heart failure	2187 [1701, 2575]	2002 [1921, 2077]	2446 [1345, 2626]	2196 [1782, 3033]	2324 [2216, 2431]	5323 [4648, 5502]	2148 [2098, 2195]	2791 [2713, 2871]	745 [552, 1664]	2117 [1200, 2560]	1175 [797, 1391]	667 [520, 777]	1307 [1169, 1491]	6587 [6460, 6701]	1472 [778, 3379]	374 [295, 447]
Baseline	60%	60%	Heart failure	2135 [1684, 2521]	2002 [1921, 2077]	2442 [1345, 2611]	2152 [1770, 2962]	2324 [2216, 2431]	5131 [4600, 5301]	2148 [2095, 2195]	2638 [2551, 2733]	745 [552, 1664]	2062 [1200, 2518]	1175 [797, 1391]	667 [520, 777]	1306 [1169, 1468]	6400 [6286, 6520]	1472 [778, 3377]	374 [295, 447]
Baseline	60%	80%	Heart failure	2135 [1684, 2521]	2002 [1921, 2077]	2442 [1345, 2611]	2152 [1770, 2962]	2324 [2216, 2431]	5131 [4600, 5301]	2148 [2095, 2195]	2638 [2551, 2733]	745 [552, 1664]	2062 [1200, 2518]	1175 [797, 1391]	667 [520, 777]	1306 [1169, 1468]	6400 [6286, 6520]	1472 [778, 3377]	374 [295, 447]
Baseline	80%	60%	Heart failure	2096 [1662, 2477]	1971 [1890, 2046]	2380 [1310, 2549]	2125 [1730, 2852]	2324 [2216, 2431]	5002 [4521, 5187]	2122 [2069, 2169]	2632 [2551, 2713]	738 [544, 1664]	2016 [1200, 2481]	1168 [797, 1381]	662 [518, 767]	1293 [1152, 1464]	6266 [6152, 6386]	1453 [768, 3279]	374 [295, 447]
Baseline	80%	80%	Heart failure	2096 [1662, 2477]	1971 [1890, 2046]	2380 [1310, 2549]	2125 [1730, 2852]	2324 [2216, 2431]	5002 [4521, 5187]	2122 [2069, 2169]	2632 [2551, 2713]	738 [544, 1664]	2016 [1200, 2481]	1168 [797, 1381]	662 [518, 767]	1293 [1152, 1464]	6266 [6152, 6386]	1453 [768, 3279]	374 [295, 447]
Baseline	Baseline	Baseline	ESRD	2044611 [1840457, 2157050]	2285188 [2234207, 2339169]	1667031 [1570008, 1819792]	1987311 [1799836, 2141211]	1532724 [1480700, 1588751]	2326995 [1698761, 2372481]	1618453 [1588088, 1653157]	1830986 [1765179, 1891895]	2021585 [1854175, 2164250]	1998889 [1762171, 2138219]	2154828 [2094987, 2223453]	2157835 [2114136, 2201534]	1944053 [1858166, 2103975]	2148376 [2110251, 2188408]	1780414 [1663407, 1925261]	2131810 [1938757, 2335811]
Baseline	60%	60%	ESRD	2044611 [1840457, 2157050]	2285188 [2234207, 2339169]	1667031 [1570008, 1819792]	1987311 [1799836, 2141211]	1532724 [1480700, 1588751]	2326994 [1698761, 2372481]	1618453 [1588087, 1653157]	1830986 [1765179, 1891894]	2021585 [1854175, 2164250]	1998889 [1762171, 2138219]	2154828 [2094987, 2223453]	2157835 [2114136, 2201534]	1944053 [1858166, 2103975]	2148376 [2110251, 2188408]	1780414 [1663407, 1925261]	2131810 [1938757, 2335811]
Baseline	60%	80%	ESRD	2044611 [1840457,	2285188 [2234207,	1667031 [1570008,	1987311 [1799836,	1532724 [1480700,	2326994 [1698761,	1618453 [1588087,	1830986 [1765179,	2021585 [1854175,	1998889 [1762171,	2154828 [2094987,	2157835 [2114136,	1944053 [1858166,	2148376 [2110251,	1780414 [1663407,	2131810 [1938757,

				2157050]	2339169]	1819792]	2141211]	1588751]	2372481]	1653157]	1891894]	2164250]	2138219]	2223453]	2201534]	2103975]	2188408]	1925261]	2335811]
Baseline	80%	60%	ESRD	2040606 [1838621, 2149724]	2285188 [2234207, 2339169]	1665970 [1570008, 1819792]	1987311 [1799836, 2141211]	1532724 [1480700, 1588751]	2312997 [1698761, 2358890]	1618453 [1588087, 1653157]	1830986 [1765179, 1891894]	2021074 [1853119, 2164250]	1997161 [1762171, 2134235]	2154279 [2094438, 2220968]	2157835 [2113759, 2201534]	1943913 [1858166, 2103975]	2147423 [2109774, 2186979]	1780414 [1662972, 1919777]	2131549 [1938009, 2334269]
Baseline	80%	80%	ESRD	2038720 [1838439, 2147827]	2285188 [2234207, 2339169]	1665970 [1570008, 1819792]	1987311 [1799836, 2141211]	1532724 [1480700, 1588751]	2307450 [1698761, 2352924]	1618453 [1588087, 1653157]	1830986 [1765179, 1891894]	2021074 [1853119, 2164250]	1996031 [1762171, 2133368]	2154279 [2094438, 2220968]	2157835 [2113759, 2201534]	1943913 [1858166, 2103975]	2147423 [2109774, 2186979]	1780414 [1662972, 1919777]	2131549 [1938009, 2334269]
Baseline	Baseline	Baseline	Neuropathy	85063 [66384, 96398]	98413 [96029, 100533]	83958 [31178, 104723]	69397 [62269, 74224]	208712 [203813, 213611]	262793 [185426, 277257]	95386 [94026, 96803]	90331 [88308, 92287]	20778 [10910, 67367]	58641 [44908, 63072]	39211 [36370, 45398]	11305 [9252, 13310]	40633 [33082, 45843]	351188 [346116, 356666]	51847 [12872, 133124]	6429 [5665, 7441]
Baseline	60%	60%	Neuropathy	84661 [65890, 95481]	98413 [96029, 100533]	83958 [31080, 104723]	69306 [62269, 74200]	208712 [203813, 213611]	261658 [182826, 271663]	95386 [94026, 96803]	90230 [88207, 92163]	20194 [10910, 67367]	58641 [44908, 63058]	39211 [36370, 45398]	11305 [9252, 13310]	40633 [33082, 45793]	344899 [340030, 350377]	51847 [12872, 133124]	6429 [5665, 7441]
Baseline	60%	80%	Neuropathy	84661 [65890, 95481]	98413 [96029, 100533]	83958 [31080, 104723]	69306 [62269, 74200]	208712 [203813, 213611]	261658 [182826, 271663]	95386 [94026, 96803]	90230 [88207, 92163]	20194 [10910, 67367]	58641 [44908, 63058]	39211 [36370, 45398]	11305 [9252, 13310]	40633 [33082, 45793]	344899 [340030, 350377]	51847 [12872, 133124]	6429 [5665, 7441]
Baseline	80%	60%	Neuropathy	82905 [64446, 93568]	97884 [95466, 100036]	82510 [30689, 103771]	67778 [61293, 72454]	204466 [199893, 209692]	254810 [176813, 264535]	94592 [93218, 96066]	88510 [86488, 90436]	20259 [10910, 66784]	57967 [44673, 62188]	38894 [36181, 45109]	11219 [9165, 13191]	40250 [32742, 45437]	335769 [330900, 341044]	51517 [12853, 131533]	6429 [5665, 7441]
Baseline	80%	80%	Neuropathy	81522 [63347, 91809]	97751 [95367, 100003]	82221 [30200, 102343]	66470 [60365, 71332]	203159 [198505, 208140]	248934 [172710, 256417]	94196 [92779, 95612]	87195 [85172, 89218]	19953 [10910, 66695]	57563 [44556, 61739]	38744 [36034, 44859]	11138 [9075, 13136]	39994 [32566, 45147]	327857 [322582, 332980]	51517 [12853, 130544]	6429 [5665, 7441]

Baseline	Baseline	Baseline	Vision loss	5480 [4329, 6210]	6113 [5923, 6268]	2518 [1674, 6753]	5249 [4927, 5755]	11770 [11447, 12093]	14355 [9184, 14639]	6521 [6403, 6639]	7091 [6902, 7284]	2276 [1235, 4535]	4353 [3549, 4801]	3242 [3075, 3642]	1275 [1179, 1357]	3418 [3057, 4065]	17344 [17043, 17674]	3909 [1554, 8464]	832 [705, 996]
Baseline	60%	60%	Vision loss	5480 [4329, 6209]	6113 [5923, 6268]	2518 [1674, 6753]	5249 [4927, 5755]	11770 [11447, 12093]	14353 [9184, 14634]	6521 [6403, 6639]	7091 [6902, 7284]	2276 [1235, 4535]	4353 [3549, 4801]	3242 [3075, 3642]	1275 [1179, 1357]	3418 [3057, 4065]	17344 [17043, 17674]	3909 [1554, 8464]	832 [705, 996]
Baseline	60%	80%	Vision loss	5480 [4329, 6209]	6113 [5923, 6268]	2518 [1674, 6753]	5249 [4927, 5755]	11770 [11447, 12093]	14353 [9184, 14634]	6521 [6403, 6639]	7091 [6902, 7284]	2276 [1235, 4535]	4353 [3549, 4801]	3242 [3075, 3642]	1275 [1179, 1357]	3418 [3057, 4065]	17344 [17043, 17674]	3909 [1554, 8464]	832 [705, 996]
Baseline	80%	60%	Vision loss	5428 [4309, 6153]	6113 [5923, 6268]	2505 [1658, 6753]	5214 [4914, 5688]	11770 [11447, 12074]	14132 [9144, 14411]	6510 [6392, 6628]	7040 [6850, 7218]	2249 [1235, 4535]	4338 [3549, 4791]	3236 [3072, 3633]	1274 [1179, 1354]	3411 [3056, 4057]	17028 [16741, 17362]	3909 [1554, 8445]	832 [705, 996]
Baseline	80%	80%	Vision loss	5423 [4308, 6147]	6113 [5923, 6268]	2505 [1658, 6753]	5214 [4914, 5688]	11770 [11447, 12074]	14128 [9144, 14401]	6510 [6392, 6628]	7040 [6850, 7218]	2249 [1235, 4535]	4338 [3549, 4791]	3236 [3072, 3633]	1274 [1179, 1354]	3411 [3056, 4057]	17028 [16738, 17362]	3909 [1554, 8445]	832 [705, 996]
60%	Baseline	Baseline	Screening	4555 [3444, 5694]	4815 [3611, 6019]	5613 [4210, 7017]	4276 [3207, 5345]	9256 [6942, 11570]	8289 [6217, 10362]	4196 [3147, 5245]	4935 [3701, 6169]	2785 [2089, 3481]	4228 [3171, 5285]	3560 [2670, 4450]	2524 [1893, 3155]	3717 [2788, 4646]	8472 [6354, 10590]	4001 [3001, 5001]	2524 [2316, 3154]
60%	60%	60%	Screening	4555 [3444, 5694]	4815 [3611, 6019]	5613 [4210, 7017]	4276 [3207, 5345]	9256 [6942, 11570]	8289 [6217, 10362]	4196 [3147, 5245]	4935 [3701, 6169]	2785 [2089, 3481]	4228 [3171, 5285]	3560 [2670, 4450]	2524 [1893, 3155]	3717 [2788, 4646]	8472 [6354, 10590]	4001 [3001, 5001]	2524 [2316, 3154]
60%	60%	80%	Screening	4555 [3444, 5694]	4815 [3611, 6019]	5613 [4210, 7017]	4276 [3207, 5345]	9256 [6942, 11570]	8289 [6217, 10362]	4196 [3147, 5245]	4935 [3701, 6169]	2785 [2089, 3481]	4228 [3171, 5285]	3560 [2670, 4450]	2524 [1893, 3155]	3717 [2788, 4646]	8472 [6354, 10590]	4001 [3001, 5001]	2524 [2316, 3154]
60%	80%	60%	Screening	4555 [3444	4815 [3611	5613 [4210	4276 [3207	9256 [6942,	8289 [6217,	4196 [3147	4935 [3701	2785 [2089	4228 [3171, 5285]	3560 [2670	2524 [1893	3717 [2788	8472 [6354,	4001 [3001	2524 [2316

				, 5694]	, 6019]	, 7017]	, 5345]	11570]	10362]	, 5245]	, 6169]	, 3481]		, 4450]	, 3155]	, 4646]	10590]	, 5001]	, 3154]
60%	80%	80%	Screening	4555 [3444, 5694]	4815 [3611, 6019]	5613 [4210, 7017]	4276 [3207, 5345]	9256 [6942, 11570]	8289 [6217, 10362]	4196 [3147, 5245]	4935 [3701, 6169]	2785 [2089, 3481]	4228 [3171, 5285]	3560 [2670, 4450]	2524 [1893, 3155]	3717 [2788, 4646]	8472 [6354, 10590]	4001 [3001, 5001]	2524 [2316, 3154]
60%	Baseline	Baseline	Blood pressure medicines	10383 [7712, 31445]	27251 [27251, 27251]	20202 [7416, 113126]	8971 [6911, 14177]	0 [0, 0]	26924 [20985, 151042]	30277 [30277, 30277]	15416 [12765, 18066]	2553 [1538, 4196]	7302 [4344, 8858]	1483 [737, 8647]	2463 [1708, 3219]	9522 [5612, 19412]	20282 [20282, 20282]	11410 [10122, 13140]	1973 [810, 3156]
60%	60%	60%	Blood pressure medicines	38439 [30994, 80202]	84517 [83665, 85351]	63067 [16869, 247812]	27608 [26811, 60845]	47809 [47360, 48258]	59776 [58814, 232818]	147637 [146743, 148458]	41512 [37142, 46250]	25304 [21593, 29397]	22830 [20148, 30273]	6609 [5353, 80005]	13380 [12833, 13932]	78423 [29360, 87320]	61097 [60640, 61538]	30626 [28530, 33104]	28712 [25391, 30435]
60%	60%	80%	Blood pressure medicines	38439 [30994, 80202]	84517 [83665, 85351]	63067 [16869, 247812]	27608 [26811, 60845]	47809 [47360, 48258]	59776 [58814, 232818]	147637 [146743, 148458]	41512 [37142, 46250]	25304 [21593, 29397]	22830 [20148, 30273]	6609 [5353, 80005]	13380 [12833, 13932]	78423 [29360, 87320]	61097 [60640, 61538]	30626 [28530, 33104]	28712 [25391, 30435]
60%	80%	60%	Blood pressure medicines	42649 [34480, 89165]	93890 [93154, 94611]	70126 [18675, 276293]	30577 [29824, 67819]	53114 [52743, 53487]	66396 [65205, 259203]	164038 [163235, 164751]	46152 [41275, 51402]	28113 [24179, 32660]	25367 [22419, 33601]	7345 [5946, 88729]	14844 [14261, 15480]	86595 [32919, 96899]	67881 [67504, 68264]	34043 [31771, 36781]	31908 [28179, 33885]
60%	80%	80%	Blood pressure medicines	51171 [41429, 107040]	112599 [111939, 113337]	84172 [22347, 332456]	36616 [35840, 81510]	63745 [63382, 64088]	79668 [78161, 311413]	196807 [196106, 197511]	55565 [49563, 61690]	33733 [29189, 39226]	30421 [26934, 40268]	8815 [7133, 106382]	17810 [17115, 18581]	103965 [39870, 116154]	81475 [81095, 81820]	40724 [38089, 44125]	38299 [33786, 40854]
60%	Baseline	Baseline	Diabetes medicines	28462 [15229, 39315]	53571 [53571, 53571]	82390 [20605, 131214]	19477 [11090, 20815]	18460 [18460, 18460]	88212 [26054, 115877]	21471 [21471, 21471]	23901 [19389, 28414]	8718 [4136, 9808]	19984 [15935, 21689]	14076 [4717, 26878]	8687 [6491, 10884]	22081 [17781, 56564]	53533 [53533, 53533]	13185 [12401, 13762]	3419 [1404, 9300]
60%	60%	60%	Diabetes medicines	57286 [44880,	83105 [82483,	130757 [29609,	28949 [26989,	116699 [115701,	145293 [121712,	39253 [39018,	34332 [33070,	23804 [21235,	28894 [26148, 32793]	48419 [46963,	20604 [20403,	85033 [24589,	95021 [94374,	22029 [21130,	34201 [33189,

				72465]	83800]	167298]	45910]	117796]	158411]	39477]	35702]	25359]		112467]	20786]	103554]	95607]	22850]	34949]
60%	60%	80%	Diabetes medicines	57286 [44880, 72465]	83105 [82483, 83800]	130757 [29609, 167298]	28949 [26989, 45910]	116699 [115701, 117796]	145293 [121712, 158411]	39253 [39018, 39477]	34332 [33070, 35702]	23804 [21235, 25359]	28894 [26148, 32793]	48419 [46963, 112467]	20604 [20403, 20786]	85033 [24589, 103554]	95021 [94374, 95607]	22029 [21130, 22850]	34201 [33189, 34949]
60%	80%	60%	Diabetes medicines	64906 [50893, 82150]	94232 [93670, 94809]	148159 [33442, 190215]	32812 [30532, 52101]	132285 [131445, 133203]	164705 [137837, 179823]	44484 [44289, 44690]	38823 [37473, 40467]	26978 [23998, 28758]	32745 [29622, 37154]	54761 [53282, 127320]	23354 [23134, 23540]	96327 [28167, 117405]	107668 [107132, 108182]	24950 [23931, 25887]	38752 [37720, 39534]
60%	80%	80%	Diabetes medicines	76396 [59888, 96677]	110878 [110310, 111512]	174382 [39262, 224270]	38617 [35910, 61356]	155665 [154817, 156463]	193734 [162105, 211760]	52350 [52140, 52540]	45701 [44097, 47650]	31758 [28205, 33838]	38518 [34867, 43718]	64356 [62735, 149747]	27471 [27217, 27698]	113804 [33154, 138133]	126695 [126182, 127163]	29317 [28162, 30454]	45597 [44440, 46464]
60%	Baseline	Baseline	Statins	2979 [1784, 9132]	10299 [10299, 10299]	2752 [2740, 18628]	1142 [854, 1515]	931 [931, 931]	8212 [5042, 42094]	1749 [1749, 1749]	2854 [2297, 3410]	301 [107, 1087]	3078 [1749, 4674]	397 [62, 3579]	861 [318, 1403]	4788 [1803, 9098]	4039 [4039, 4039]	1201 [593, 1858]	444 [0, 1350]
60%	60%	60%	Statins	13038 [9920, 33853]	60487 [60032, 61010]	21315 [18069, 118497]	6666 [5839, 7935]	12561 [12458, 12663]	23522 [20992, 115899]	15850 [15767, 15932]	11549 [10320, 12862]	7569 [5975, 8705]	8235 [6015, 15146]	2098 [1593, 31998]	6755 [6305, 7118]	29501 [10887, 38532]	12305 [12224, 12386]	8358 [7604, 8723]	12961 [12104, 13684]
60%	60%	80%	Statins	13038 [9920, 33853]	60487 [60032, 61010]	21315 [18069, 118497]	6666 [5839, 7935]	12561 [12458, 12663]	23522 [20992, 115899]	15850 [15767, 15932]	11549 [10320, 12862]	7569 [5975, 8705]	8235 [6015, 15146]	2098 [1593, 31998]	6755 [6305, 7118]	29501 [10887, 38532]	12305 [12224, 12386]	8358 [7604, 8723]	12961 [12104, 13684]
60%	80%	60%	Statins	17383 [13228, 45166]	80713 [80289, 81120]	28446 [23971, 158896]	8888 [7750, 10640]	16749 [16670, 16833]	31364 [27962, 154850]	21139 [21069, 21204]	15427 [13758, 17150]	10123 [7994, 11541]	10983 [8016, 20139]	2790 [2122, 42579]	8968 [8413, 9497]	39349 [14566, 51362]	16409 [16348, 16473]	11125 [10196, 11630]	17214 [16071, 18230]
60%	80%	80%	Statins	17383 [13228,	80713 [80289,	28446 [23971,	8888 [7750,	16749 [16670,	31364 [27962,	21139 [21069,	15427 [13758,	10123 [7994,	10983 [8016, 20139]	2790 [2122,	8968 [8413, 9497]	39349 [14566,	16409 [16348,	11125 [10196,	17214 [16071,

				45166]	81120]	158896]	10640]	16833]	154850]	21204]	17150]	11541]		42579]		51362]	16473]	11630]	18230]
60%	Baseline	Baseline	Cardiovascular events	144469 [113718, 174699]	98405 [95714, 101288]	156038 [46437, 178629]	191734 [168474, 214715]	625009 [614462, 634638]	261087 [201619, 312560]	246503 [243494, 249178]	279274 [265322, 295326]	36681 [22172, 92644]	154517 [97487, 187204]	60877 [56258, 80349]	21588 [21088, 22054]	58906 [54244, 76702]	554756 [546712, 561389]	88743 [30433, 211586]	12004 [10177, 16660]
60%	60%	60%	Cardiovascular events	109755 [89351, 130527]	82260 [79570, 84567]	114121 [35669, 124086]	137501 [126815, 156080]	457178 [447548, 465890]	204546 [166584, 229900]	202870 [200362, 205628]	193532 [188093, 199613]	27144 [16870, 74605]	114403 [77845, 136430]	49936 [44280, 63128]	17245 [16777, 17679]	46707 [40613, 60161]	433672 [427040, 440023]	61165 [21810, 165746]	9657 [7780, 11932]
60%	60%	80%	Cardiovascular events	109755 [89351, 130527]	82260 [79570, 84567]	114121 [35669, 124086]	137501 [126815, 156080]	457178 [447548, 465890]	204546 [166584, 229900]	202870 [200362, 205628]	193532 [188093, 199613]	27144 [16870, 74605]	114403 [77845, 136430]	49936 [44280, 63128]	17245 [16777, 17679]	46707 [40613, 60161]	433672 [427040, 440023]	61165 [21810, 165746]	9657 [7780, 11932]
60%	80%	60%	Cardiovascular events	101346 [83191, 120366]	77648 [75149, 80146]	104464 [32809, 112146]	126545 [115693, 143513]	416367 [407654, 425079]	189441 [155961, 211351]	192170 [189579, 194845]	176177 [172105, 180295]	25310 [15545, 69306]	104330 [73138, 124322]	47092 [41405, 58933]	16142 [15708, 16563]	43661 [37229, 56353]	405307 [399097, 411516]	55048 [20083, 156344]	9030 [7204, 11031]
60%	80%	80%	Cardiovascular events	98450 [80961, 115339]	76687 [74380, 79378]	100149 [32136, 107317]	121708 [109469, 136675]	401234 [392407, 409488]	185699 [155057, 202332]	188576 [185985, 191167]	165025 [161697, 168705]	24210 [15063, 68209]	101235 [71327, 117721]	46261 [40326, 57161]	15774 [15321, 16209]	42648 [35815, 54654]	393452 [386678, 399662]	52808 [18966, 148974]	8810 [6957, 10554]
60%	Baseline	Baseline	Heart failure	2187 [1701, 2575]	2002 [1921, 2077]	2446 [1345, 2626]	2196 [1782, 3033]	2324 [2216, 2431]	5323 [4648, 5502]	2148 [2098, 2195]	2791 [2713, 2871]	745 [552, 1664]	2117 [1200, 2560]	1175 [797, 1391]	667 [520, 777]	1307 [1169, 1491]	6587 [6460, 6701]	1472 [778, 3379]	374 [295, 447]
60%	60%	60%	Heart failure	2138 [1685, 2523]	2002 [1921, 2077]	2442 [1345, 2611]	2152 [1770, 2962]	2324 [2216, 2431]	5131 [4600, 5301]	2148 [2092, 2192]	2638 [2551, 2733]	745 [552, 1664]	2062 [1200, 2518]	1175 [797, 1391]	667 [520, 777]	1306 [1169, 1468]	6400 [6286, 6520]	1472 [778, 3377]	374 [295, 447]
60%	60%	80%	Heart failure	2138 [1685, 2523]	2002 [1921, 2077]	2442 [1345, 2611]	2152 [1770, 2962]	2324 [2216, 2431]	5131 [4600, 5301]	2148 [2092, 2192]	2638 [2551, 2733]	745 [552, 1664]	2062 [1200, 2518]	1175 [797, 1391]	667 [520, 777]	1306 [1169, 1468]	6400 [6286, 6520]	1472 [778, 3377]	374 [295, 447]
60%	80%	60%	Heart failure	2098 [1664, 2479]	1971 [1890, 2046]	2380 [1310, 2549]	2123 [1724, 2852]	2324 [2216, 2431]	5002 [4521, 5187]	2119 [2066, 2166]	2632 [2551, 2713]	738 [544, 1664]	2016 [1200, 2481]	1168 [797, 1381]	662 [518, 767]	1292 [1148, 1464]	6266 [6152, 6386]	1453 [768, 3277]	374 [295, 447]

60%	80%	80%	Heart failure	2098 [1664, 2479]	1971 [1890, 2046]	2380 [1310, 2549]	2123 [1724, 2852]	2324 [2216, 2431]	5002 [4521, 5187]	2119 [2066, 2166]	2632 [2551, 2713]	738 [544, 1664]	2016 [1200, 2481]	1168 [797, 1381]	662 [518, 767]	1292 [1148, 1464]	6266 [6152, 6386]	1453 [768, 3277]	374 [295, 447]
60%	Baseline	Baseline	ESRD	2044611 [1840457, 2157050]	2285188 [2234207, 2339169]	1667031 [1570008, 1819792]	1987311 [1799836, 2141211]	1532724 [1480700, 1588751]	2326995 [1698761, 2372481]	1618453 [1588088, 1653157]	1830986 [1765179, 1891895]	2021585 [1854175, 2164250]	1998889 [1762171, 2138219]	2154828 [2094987, 2223453]	2157835 [2114136, 2201534]	1944053 [1858166, 2103975]	2148376 [2110251, 2188408]	1780414 [1663407, 1925261]	2131810 [1938757, 2335811]
60%	60%	60%	ESRD	2044611 [1840457, 2157050]	2285188 [2234207, 2339169]	1667031 [1570008, 1819792]	1987311 [1799836, 2141211]	1532724 [1480700, 1588751]	2326994 [1698761, 2372481]	1618453 [1588087, 1653157]	1830986 [1765179, 1891894]	2021585 [1854175, 2164250]	1998889 [1762171, 2138219]	2154828 [2094987, 2223453]	2157835 [2114136, 2201534]	1944053 [1858166, 2103975]	2148376 [2110251, 2188408]	1780414 [1663407, 1925261]	2131810 [1938757, 2335811]
60%	60%	80%	ESRD	2044611 [1840457, 2157050]	2285188 [2234207, 2339169]	1667031 [1570008, 1819792]	1987311 [1799836, 2141211]	1532724 [1480700, 1588751]	2326994 [1698761, 2372481]	1618453 [1588087, 1653157]	1830986 [1765179, 1891894]	2021585 [1854175, 2164250]	1998889 [1762171, 2138219]	2154828 [2094987, 2223453]	2157835 [2114136, 2201534]	1944053 [1858166, 2103975]	2148376 [2110251, 2188408]	1780414 [1663407, 1925261]	2131810 [1938757, 2335811]
60%	80%	60%	ESRD	2041355 [1839604, 2151259]	2285188 [2231208, 2336170]	1665970 [1570008, 1819792]	1987311 [1799836, 2138076]	1532724 [1480700, 1588751]	2312997 [1698761, 2358890]	1618453 [1588087, 1653157]	1830986 [1765179, 1891552]	2019416 [1853119, 2164250]	1997161 [1762171, 2133368]	2153669 [2093678, 2220159]	2154822 [2108866, 2198521]	1941512 [1856255, 2089124]	2147423 [2109774, 2186979]	1780414 [1662972, 1919226]	2126778 [1936612, 2331689]
60%	80%	80%	ESRD	2039864 [1839175, 2149646]	2282190 [2231208, 2336170]	1665970 [1570008, 1819792]	1987311 [1799836, 2136062]	1532724 [1480700, 1588751]	2307450 [1698761, 2352924]	1618453 [1588087, 1653157]	1828077 [1765179, 1891552]	2019416 [1849450, 2164250]	1996031 [1762171, 2131287]	2153604 [2093023, 2220159]	2153736 [2105875, 2197014]	1941512 [1856081, 2089071]	2147423 [2109774, 2186979]	1780414 [1662972, 1919226]	2121741 [1936612, 2329943]
60%	Baseline	Baseline	Neuropathy	85063 [66384, 96398]	98413 [96029, 100533]	83958 [31178, 104723]	69397 [62269, 74224]	208712 [203813, 213611]	262793 [185426, 277257]	95386 [94026, 96803]	90331 [88308, 92287]	20778 [10910, 67367]	58641 [44908, 63072]	39211 [36370, 45398]	11305 [9252, 13310]	40633 [33082, 45843]	351188 [346116, 356666]	51847 [12872, 133124]	6429 [5665, 7441]
60%	60%	60%	Neuropathy	84730 [65897, 95508]	98413 [96029, 100533]	83958 [31080, 104723]	69306 [62269, 74134]	208712 [203813, 213611]	261658 [182826, 271663]	95301 [93912, 96689]	90230 [88207, 92163]	20194 [10910, 67367]	58641 [44908, 63058]	39211 [36344, 45398]	11305 [9252, 13310]	40633 [33082, 45793]	344899 [340030, 350377]	51847 [12872, 133124]	6429 [5665, 7441]

60%	60%	80%	Neuropathy	84730 [65897, 95508]	98413 [96029, 100533]	83958 [31080, 104723]	69306 [62269, 74134]	208712 [203813, 213611]	261658 [182826, 271663]	95301 [93912, 96689]	90230 [88207, 92163]	20194 [10910, 67367]	58641 [44908, 63058]	39211 [36344, 45398]	11305 [9252, 13310]	40633 [33082, 45793]	344899 [340030, 350377]	51847 [12872, 133124]	6429 [5665, 7441]
60%	80%	60%	Neuropathy	83085 [64533, 93675]	97751 [95235, 100003]	82510 [30689, 103771]	67758 [61293, 72330]	204466 [199893, 209692]	254810 [176813, 264535]	94054 [92665, 95499]	88510 [86487, 90436]	20259 [10853, 66717]	57967 [44556, 62183]	38844 [36070, 45109]	11204 [9136, 13191]	40178 [32742, 45233]	335769 [330900, 341044]	51517 [12841, 131372]	6429 [5659, 7441]
60%	80%	80%	Neuropathy	81805 [63528, 92004]	97552 [95102, 99738]	82221 [30200, 102343]	66470 [60318, 71219]	203159 [198505, 208140]	248934 [172710, 256417]	93232 [91872, 94649]	87105 [85131, 89202]	19934 [10795, 66638]	57563 [44263, 61726]	38658 [35848, 44859]	11119 [8991, 13118]	39994 [32566, 44724]	327857 [322582, 332980]	51517 [12841, 130242]	6429 [5637, 7441]
60%	Baseline	Baseline	Vision loss	5480 [4329, 6210]	6113 [5923, 6268]	2518 [1674, 6753]	5249 [4927, 5755]	11770 [11447, 12093]	14355 [9184, 14639]	6521 [6403, 6639]	7091 [6902, 7284]	2276 [1235, 4535]	4353 [3549, 4801]	3242 [3075, 3642]	1275 [1179, 1357]	3418 [3057, 4065]	17344 [17043, 17674]	3909 [1554, 8464]	832 [705, 996]
60%	60%	60%	Vision loss	5480 [4329, 6209]	6113 [5923, 6268]	2518 [1674, 6753]	5249 [4927, 5755]	11770 [11447, 12093]	14353 [9184, 14634]	6521 [6403, 6639]	7091 [6902, 7284]	2276 [1235, 4535]	4353 [3549, 4801]	3242 [3075, 3642]	1275 [1179, 1357]	3418 [3057, 4065]	17344 [17043, 17674]	3909 [1554, 8464]	832 [705, 996]
60%	60%	80%	Vision loss	5480 [4329, 6209]	6113 [5923, 6268]	2518 [1674, 6753]	5249 [4927, 5755]	11770 [11447, 12093]	14353 [9184, 14634]	6521 [6403, 6639]	7091 [6902, 7284]	2276 [1235, 4535]	4353 [3549, 4801]	3242 [3075, 3642]	1275 [1179, 1357]	3418 [3057, 4065]	17344 [17043, 17674]	3909 [1554, 8464]	832 [705, 996]
60%	80%	60%	Vision loss	5432 [4311, 6159]	6113 [5923, 6268]	2505 [1658, 6753]	5213 [4905, 5688]	11770 [11447, 12074]	14132 [9144, 14411]	6483 [6365, 6603]	7040 [6850, 7218]	2249 [1226, 4535]	4338 [3549, 4791]	3236 [3068, 3633]	1274 [1179, 1354]	3411 [3056, 4045]	17028 [16741, 17362]	3909 [1554, 8429]	832 [705, 996]
60%	80%	80%	Vision loss	5431 [4310, 6156]	6113 [5923, 6268]	2505 [1658, 6753]	5213 [4905, 5688]	11770 [11447, 12074]	14128 [9144, 14401]	6478 [6360, 6596]	7031 [6838, 7212]	2249 [1226, 4521]	4338 [3549, 4791]	3236 [3068, 3633]	1274 [1179, 1354]	3411 [3056, 4045]	17028 [16738, 17362]	3909 [1554, 8427]	832 [705, 996]

80%	Baseline	Base line	Screening	5204 [3903, 6505]	5757 [4318, 7197]	5908 [4431, 7385]	4918 [3688, 6147]	9328 [6996, 11660]	8853 [6640, 11066]	5245 [3934, 6556]	5410 [4058, 6763]	3383 [2537, 4229]	4875 [3656, 6094]	4359 [3269, 5448]	3100 [2325, 3875]	4268 [3201, 5335]	9451 [7088, 11813]	4809 [3606, 6011]	3116 [2337, 3896]
80%	60%	60%	Screening	5204 [3903, 6505]	5757 [4318, 7197]	5908 [4431, 7385]	4918 [3688, 6147]	9328 [6996, 11660]	8853 [6640, 11066]	5245 [3934, 6556]	5410 [4058, 6763]	3383 [2537, 4229]	4875 [3656, 6094]	4359 [3269, 5448]	3100 [2325, 3875]	4268 [3201, 5335]	9451 [7088, 11813]	4809 [3606, 6011]	3116 [2337, 3896]
80%	60%	80%	Screening	5204 [3903, 6505]	5757 [4318, 7197]	5908 [4431, 7385]	4918 [3688, 6147]	9328 [6996, 11660]	8853 [6640, 11066]	5245 [3934, 6556]	5410 [4058, 6763]	3383 [2537, 4229]	4875 [3656, 6094]	4359 [3269, 5448]	3100 [2325, 3875]	4268 [3201, 5335]	9451 [7088, 11813]	4809 [3606, 6011]	3116 [2337, 3896]
80%	80%	60%	Screening	5204 [3903, 6505]	5757 [4318, 7197]	5908 [4431, 7385]	4918 [3688, 6147]	9328 [6996, 11660]	8853 [6640, 11066]	5245 [3934, 6556]	5410 [4058, 6763]	3383 [2537, 4229]	4875 [3656, 6094]	4359 [3269, 5448]	3100 [2325, 3875]	4268 [3201, 5335]	9451 [7088, 11813]	4809 [3606, 6011]	3116 [2337, 3896]
80%	80%	80%	Screening	5204 [3903, 6505]	5757 [4318, 7197]	5908 [4431, 7385]	4918 [3688, 6147]	9328 [6996, 11660]	8853 [6640, 11066]	5245 [3934, 6556]	5410 [4058, 6763]	3383 [2537, 4229]	4875 [3656, 6094]	4359 [3269, 5448]	3100 [2325, 3875]	4268 [3201, 5335]	9451 [7088, 11813]	4809 [3606, 6011]	3116 [2337, 3896]
80%	Baseline	Base line	Blood pressure medicines	10383 [7712, 31445]	27251 [27251, 27251]	20202 [7416, 113126]	8971 [6911, 14177]	0 [0, 0]	26924 [20985, 151042]	30277 [30277, 30277]	15416 [12765, 18066]	2553 [1538, 4196]	7302 [4344, 8858]	1483 [737, 8647]	2463 [1708, 3219]	9522 [5612, 19412]	20282 [20282, 20282]	11410 [10122, 13140]	1973 [810, 3156]
80%	60%	60%	Blood pressure medicines	43913 [35431, 88687]	100880 [99973, 101796]	65845 [18317, 247812]	30613 [29588, 72777]	48145 [47699, 48590]	63748 [60283, 237150]	184371 [183413, 185331]	45100 [42475, 48029]	32846 [27578, 35569]	25383 [23658, 31497]	7635 [6987, 94469]	16337 [14999, 17675]	89610 [32000, 108681]	68276 [67801, 68709]	36572 [33864, 39371]	37336 [33341, 38869]
80%	60%	80%	Blood pressure medicines	43913 [35431, 88687]	100880 [99973, 101796]	65845 [18317, 247812]	30613 [29588, 72777]	48145 [47699, 48590]	63748 [60283, 237150]	184371 [183413, 185331]	45100 [42475, 48029]	32846 [27578, 35569]	25383 [23658, 31497]	7635 [6987, 94469]	16337 [14999, 17675]	89610 [32000, 108681]	68276 [67801, 68709]	36572 [33864, 39371]	37336 [33341, 38869]
80%	80%	60%	Blood pressure medicines	48777 [39412,	112038 [111271,	73195 [20290,	33926 [32858,	53495 [53119,	70825 [66848,	204876 [203982,	50127 [47198,	36506 [30843,	28196 [26301, 34954]	8483 [7767, 104697]	18123 [16672,	99484 [35862,	75846 [75474,	40612 [37839,	41525 [37021,

				98598]	112823]	276293]	81071]	53863]	264020]	205633]	53387]	39463]			19642]	120917]	76237]	43750]	43224]
80%	80%	80%	Blood pressure medicines	58550 [47352, 118332]	134375 [133706, 135197]	87817 [24292, 332456]	40641 [39370, 97428]	64192 [63841, 64540]	84979 [80116, 317188]	245817 [245042, 246552]	60367 [56665, 64084]	43808 [37204, 47252]	33810 [31586, 41900]	10180 [9326, 125338]	21705 [20006, 23576]	119688 [43426, 145105]	91033 [90664, 91396]	48629 [45590, 52495]	49874 [44408, 51945]
80%	Baseline	Baseline	Diabetes medicines	28462 [15229, 39315]	53571 [53571, 53571]	82390 [20605, 131214]	19477 [11090, 20815]	18460 [18460, 18460]	88212 [26054, 115877]	21471 [21471, 21471]	23901 [19389, 28414]	8718 [4136, 9808]	19984 [15935, 21689]	14076 [4717, 26878]	8687 [6491, 10884]	22081 [17781, 56564]	53533 [53533, 53533]	13185 [12401, 13762]	3419 [1404, 9300]
80%	60%	60%	Diabetes medicines	65420 [51560, 83195]	103182 [102488, 103930]	138694 [32453, 167298]	34816 [31378, 59969]	117854 [116864, 118966]	148936 [130589, 161115]	51845 [51557, 52081]	38674 [37670, 39600]	30107 [27772, 33198]	32043 [31281, 33216]	62943 [56485, 141069]	25985 [25018, 26838]	103659 [27428, 131941]	107736 [107107, 108362]	27449 [26199, 28142]	45428 [41793, 46712]
80%	60%	80%	Diabetes medicines	65420 [51560, 83195]	103182 [102488, 103930]	138694 [32453, 167298]	34816 [31378, 59969]	117854 [116864, 118966]	148936 [130589, 161115]	51845 [51557, 52081]	38674 [37670, 39600]	30107 [27772, 33198]	32043 [31281, 33216]	62943 [56485, 141069]	25985 [25018, 26838]	103659 [27428, 131941]	107736 [107107, 108362]	27449 [26199, 28142]	45428 [41793, 46712]
80%	80%	60%	Diabetes medicines	74168 [58458, 94338]	117017 [116379, 117584]	157172 [36656, 190215]	39547 [35594, 68053]	133599 [132751, 134507]	168829 [147902, 182888]	58745 [58520, 58979]	43892 [42709, 44895]	34166 [31554, 37636]	36330 [35470, 37605]	71321 [63958, 160012]	29401 [28357, 30423]	117545 [31375, 149524]	122095 [121579, 122619]	31113 [29706, 31898]	51550 [47240, 52895]
80%	80%	80%	Diabetes medicines	87289 [68773, 110908]	137661 [137052, 138300]	184929 [43036, 224270]	46556 [41901, 80128]	157180 [156357, 157994]	198570 [173935, 215359]	69110 [68882, 69345]	51389 [50278, 52837]	40227 [37172, 44264]	42758 [41741, 44223]	83896 [75199, 188378]	34457 [33378, 35790]	138716 [36969, 174652]	143667 [143169, 144161]	36524 [34943, 37519]	60715 [55476, 62229]
80%	Baseline	Baseline	Statins	2979 [1784, 9132]	10299 [10299, 10299]	2752 [2740, 18628]	1142 [854, 1515]	931 [931, 931]	8212 [5042, 42094]	1749 [1749, 1749]	2854 [2297, 3410]	301 [107, 1087]	3078 [1749, 4674]	397 [62, 3579]	861 [318, 1403]	4788 [1803, 9098]	4039 [4039, 4039]	1201 [593, 1858]	444 [0, 1350]
80%	60%	60%	Statins	14685 [11083,	70465 [69991,	22336 [19095,	6952 [6546, 9365]	12660 [12556,	23979 [22211,	19616 [19528,	12495 [11760,	8952 [7716, 9762]	8235 [6692, 16940]	2411 [2023,	7916 [7786, 8012]	37342 [11290,	13585 [13503,	9785 [8956,	16912 [14366,

				36593]	71000]	118497]		12761]	117609]	19712]	13321]			37730]		43929]	13662]	10179]	17521]
80%	60%	80%	Statins	14685 [11083, 36593]	70465 [69991, 71000]	22336 [19095, 118497]	6952 [6546, 9365]	12660 [12556, 12761]	23979 [22211, 117609]	19616 [19528, 19712]	12495 [11760, 13321]	8952 [7716, 9762]	8235 [6692, 16940]	2411 [2023, 37730]	7916 [7786, 8012]	37342 [11290, 43929]	13585 [13503, 13662]	9785 [8956, 10179]	16912 [14366, 17521]
80%	80%	60%	Statins	19579 [14778, 48827]	93973 [93549, 94416]	29808 [25322, 158896]	9255 [8720, 12539]	16877 [16798, 16959]	31966 [29588, 157154]	26165 [26086, 26236]	16706 [15676, 17763]	11938 [10322, 12909]	10983 [8952, 22526]	3211 [2694, 50363]	10552 [10386, 10684]	49756 [15042, 58494]	18117 [18054, 18179]	13072 [12006, 13578]	22551 [19005, 23298]
80%	80%	80%	Statins	19579 [14778, 48827]	93973 [93549, 94416]	29808 [25322, 158896]	9255 [8720, 12539]	16877 [16798, 16959]	31966 [29588, 157154]	26165 [26086, 26236]	16706 [15676, 17763]	11938 [10322, 12909]	10983 [8952, 22526]	3211 [2694, 50363]	10552 [10386, 10684]	49756 [15042, 58494]	18117 [18054, 18179]	13072 [12006, 13578]	22551 [19005, 23298]
80%	Baseline	Baseline	Cardiovascular events	144469 [113718, 174699]	98405 [95714, 101288]	156038 [46437, 178629]	191734 [168474, 214715]	625009 [614462, 634638]	261087 [201619, 312560]	246503 [243494, 249178]	279274 [265322, 295326]	36681 [22172, 92644]	154517 [97487, 187204]	60877 [56258, 80349]	21588 [21088, 22054]	58906 [54244, 76702]	554756 [546712, 561389]	88743 [30433, 211586]	12004 [10177, 16660]
80%	60%	60%	Cardiovascular events	107506 [86504, 126246]	79570 [76879, 81876]	112804 [34996, 123404]	132160 [122083, 151299]	455802 [446173, 464171]	203160 [165906, 224398]	193340 [190728, 196015]	186568 [178591, 195933]	25318 [15906, 71499]	114273 [74224, 131866]	47417 [41626, 60141]	16510 [16008, 16950]	45240 [39882, 56667]	416596 [410669, 423088]	58718 [20750, 158278]	9071 [7158, 11119]
80%	60%	80%	Cardiovascular events	107506 [86504, 126246]	79570 [76879, 81876]	112804 [34996, 123404]	132160 [122083, 151299]	455802 [446173, 464171]	203160 [165906, 224398]	193340 [190728, 196015]	186568 [178591, 195933]	25318 [15906, 71499]	114273 [74224, 131866]	47417 [41626, 60141]	16510 [16008, 16950]	45240 [39882, 56667]	416596 [410669, 423088]	58718 [20750, 158278]	9071 [7158, 11119]
80%	80%	60%	Cardiovascular events	98520 [79707, 114820]	74380 [71690, 76543]	102709 [31967, 111132]	119560 [110521, 137519]	414303 [405820, 423245]	187639 [155057, 204771]	180301 [177709, 182725]	167347 [160943, 175328]	23109 [14460, 65748]	103975 [68521, 117834]	44067 [38169, 55137]	15224 [14705, 15654]	41833 [36389, 52066]	385267 [378493, 391476]	52463 [19023, 145306]	8307 [6445, 10024]
80%	80%	80%	Cardiovascular events	95347 [77372,	73227 [70729,	98539 [31126,	113679 [104732,	399400 [390229,	184036 [154153,	175745 [173363,	155513 [149781,	21822 [13858,	100702 [66530, 110290]	43127 [37040,	14805 [14270,	40763 [35043,	371155 [364381,	49138 [18067,	7996 [6129, 9450]

				109196]	75534]	106359]	130207]	407196]	195412]	178294]	162634]	63384]		52972]	15243]	50028]	377364]	137440]	
80%	Baseline	Baseline	Heart failure	2187 [1701, 2575]	2002 [1921, 2077]	2446 [1345, 2626]	2196 [1782, 3033]	2324 [2216, 2431]	5323 [4648, 5502]	2148 [2098, 2195]	2791 [2713, 2871]	745 [552, 1664]	2117 [1200, 2560]	1175 [797, 1391]	667 [520, 777]	1307 [1169, 1491]	6587 [6460, 6701]	1472 [778, 3379]	374 [295, 447]
80%	60%	60%	Heart failure	2136 [1684, 2521]	2002 [1921, 2077]	2442 [1345, 2611]	2152 [1770, 2962]	2324 [2216, 2431]	5128 [4600, 5297]	2145 [2092, 2192]	2638 [2551, 2733]	745 [552, 1664]	2062 [1200, 2518]	1175 [797, 1391]	667 [520, 777]	1306 [1169, 1468]	6333 [6212, 6446]	1472 [778, 3377]	374 [295, 447]
80%	60%	80%	Heart failure	2136 [1684, 2521]	2002 [1921, 2077]	2442 [1345, 2611]	2152 [1770, 2962]	2324 [2216, 2431]	5128 [4600, 5297]	2145 [2092, 2192]	2638 [2551, 2733]	745 [552, 1664]	2062 [1200, 2518]	1175 [797, 1391]	667 [520, 777]	1306 [1169, 1468]	6333 [6212, 6446]	1472 [778, 3377]	374 [295, 447]
80%	80%	60%	Heart failure	2096 [1662, 2477]	1971 [1890, 2046]	2373 [1310, 2549]	2123 [1718, 2846]	2324 [2216, 2431]	5002 [4519, 5184]	2116 [2063, 2160]	2628 [2545, 2711]	738 [544, 1664]	2013 [1200, 2481]	1168 [797, 1381]	662 [518, 767]	1291 [1148, 1464]	6226 [6105, 6339]	1453 [768, 3273]	374 [295, 447]
80%	80%	80%	Heart failure	2096 [1662, 2477]	1971 [1890, 2046]	2373 [1310, 2549]	2123 [1718, 2846]	2324 [2216, 2431]	5002 [4519, 5184]	2116 [2063, 2160]	2628 [2545, 2711]	738 [544, 1664]	2013 [1200, 2481]	1168 [797, 1381]	662 [518, 767]	1291 [1148, 1464]	6226 [6105, 6339]	1453 [768, 3273]	374 [295, 447]
80%	Baseline	Baseline	ESRD	2044611 [1840457, 2157050]	2285188 [2234207, 2339169]	1667031 [1570008, 1819792]	1987311 [1799836, 2141211]	1532724 [1480700, 1588751]	2326995 [1698761, 2372481]	1618453 [1588088, 1653157]	1830986 [1765179, 1891895]	2021585 [1854175, 2164250]	1998889 [1762171, 2138219]	2154828 [2094987, 2223453]	2157835 [2114136, 2201534]	1944053 [1858166, 2103975]	2148376 [2110251, 2188408]	1780414 [1663407, 1925261]	2131810 [1938757, 2335811]
80%	60%	60%	ESRD	2044611 [1840457, 2157050]	2285188 [2234207, 2339169]	1667031 [1570008, 1819792]	1987311 [1799836, 2141211]	1532724 [1480700, 1588751]	2326994 [1698761, 2372481]	1618453 [1588087, 1653157]	1830986 [1765179, 1891894]	2021585 [1854175, 2164250]	1998889 [1762171, 2138219]	2154828 [2094987, 2223453]	2157835 [2114136, 2201534]	1944053 [1858166, 2103975]	2148376 [2110251, 2188408]	1780414 [1663407, 1925261]	2131810 [1938757, 2335811]
80%	60%	80%	ESRD	2044611 [1840457, 2157050]	2285188 [2234207, 2339169]	1667031 [1570008, 1819792]	1987311 [1799836, 2141211]	1532724 [1480700, 1588751]	2326994 [1698761, 2372481]	1618453 [1588087, 1653157]	1830986 [1765179, 1891894]	2021585 [1854175, 2164250]	1998889 [1762171, 2138219]	2154828 [2094987, 2223453]	2157835 [2114136, 2201534]	1944053 [1858166, 2103975]	2148376 [2110251, 2188408]	1780414 [1663407, 1925261]	2131810 [1938757, 2335811]
80%	80%	60%	ESRD	2040606 [1838621, 2149724]	2282190 [2231208, 2336170]	1665970 [1570008, 1819792]	1987311 [1799836, 2136062]	1532724 [1480700, 1588751]	2311161 [1698761, 2356595]	1618453 [1588087, 1653157]	1828077 [1765179, 1891552]	2019416 [1849450, 2164250]	1996031 [1760084, 2129813]	2153604 [2093023, 2220159]	2153736 [2106602, 2197014]	1941512 [1856081, 2089053]	2144564 [2106438, 2184596]	1780414 [1662972, 1919226]	2126778 [1936612, 2330200]

80%	80%	80%	ESRD	2038720 [1838438, 2147827]	2282190 [2231208, 2333921]	1665970 [1570008, 1819792]	1987311 [1799836, 2134519]	1532724 [1480700, 1588751]	2302966 [1698761, 2347814]	1618453 [1588087, 1653157]	1828077 [1765179, 1891552]	2019416 [1849450, 2164250]	1994602 [1760084, 2127981]	2153604 [2093023, 2220159]	2153315 [2105875, 2195614]	1941512 [1855862, 2089053]	2138845 [2100719, 2180783]	1780414 [1662972, 1919226]	2121741 [1936612, 2329943]
80%	Baseline	Baseline	Neuropathy	85063 [66384, 96398]	98413 [96029, 100533]	83958 [31178, 104723]	69397 [62269, 74224]	208712 [203813, 213611]	262793 [185426, 277257]	95386 [94026, 96803]	90331 [88308, 92287]	20778 [10910, 67367]	58641 [44908, 63072]	39211 [36370, 45398]	11305 [9252, 13310]	40633 [33082, 45843]	351188 [346116, 356666]	51847 [12872, 133124]	6429 [5665, 7441]
80%	60%	60%	Neuropathy	84661 [65890, 95481]	98413 [96029, 100533]	83958 [31080, 104723]	69306 [62180, 74134]	208712 [203813, 213611]	261132 [182826, 271465]	95272 [93912, 96633]	90230 [88207, 92163]	20194 [10910, 67367]	58641 [44908, 63058]	39211 [36344, 45398]	11305 [9252, 13310]	40633 [33082, 45793]	344899 [340030, 350377]	51847 [12872, 133124]	6429 [5665, 7441]
80%	60%	80%	Neuropathy	84661 [65890, 95481]	98413 [96029, 100533]	83958 [31080, 104723]	69306 [62180, 74134]	208712 [203813, 213611]	261132 [182826, 271465]	95272 [93912, 96633]	90230 [88207, 92163]	20194 [10910, 67367]	58641 [44908, 63058]	39211 [36344, 45398]	11305 [9252, 13310]	40633 [33082, 45793]	344899 [340030, 350377]	51847 [12872, 133124]	6429 [5665, 7441]
80%	80%	60%	Neuropathy	82906 [64446, 93568]	97619 [95201, 99870]	82510 [30591, 103771]	67576 [61205, 72265]	204466 [199893, 209692]	254081 [176651, 264337]	93657 [92269, 95102]	88409 [86364, 90317]	20250 [10838, 66638]	57833 [44497, 62146]	38843 [36059, 45109]	11204 [9122, 13173]	40178 [32715, 45081]	334755 [329886, 340030]	51517 [12841, 131165]	6429 [5655, 7441]
80%	80%	80%	Neuropathy	81522 [63347, 91809]	97221 [94837, 99473]	82076 [30005, 102343]	66129 [60052, 70958]	203159 [198505, 208140]	248101 [172588, 256021]	92665 [91305, 94082]	86892 [84767, 88955]	19934 [10681, 66347]	57294 [44028, 61624]	38643 [35800, 44859]	11084 [8977, 13072]	39882 [32511, 44561]	326031 [320959, 331509]	51517 [12834, 130074]	6429 [5634, 7441]
80%	Baseline	Baseline	Vision loss	5480 [4329, 6210]	6113 [5923, 6268]	2518 [1674, 6753]	5249 [4927, 5755]	11770 [11447, 12093]	14355 [9184, 14639]	6521 [6403, 6639]	7091 [6902, 7284]	2276 [1235, 4535]	4353 [3549, 4801]	3242 [3075, 3642]	1275 [1179, 1357]	3418 [3057, 4065]	17344 [17043, 17674]	3909 [1554, 8464]	832 [705, 996]
80%	60%	60%	Vision loss	5480 [4329, 6209]	6113 [5923, 6268]	2518 [1674, 6753]	5249 [4927, 5755]	11770 [11447, 12093]	14353 [9184, 14633]	6521 [6403, 6639]	7091 [6902, 7284]	2276 [1235, 4535]	4353 [3549, 4801]	3242 [3075, 3642]	1275 [1179, 1357]	3418 [3057, 4065]	17344 [17043, 17674]	3909 [1554, 8464]	832 [705, 996]

80%	60%	80%	Vision loss	5480 [4329, 6209]	6113 [5923, 6268]	2518 [1674, 6753]	5249 [4927, 5755]	11770 [11447, 12093]	14353 [9184, 14633]	6521 [6403, 6639]	7091 [6902, 7284]	2276 [1235, 4535]	4353 [3549, 4801]	3242 [3075, 3642]	1275 [1179, 1357]	3418 [3057, 4065]	17344 [17043, 17674]	3909 [1554, 8464]	832 [705, 996]
80%	80%	60%	Vision loss	5428 [4309, 6153]	6113 [5923, 6268]	2505 [1658, 6753]	5213 [4899, 5684]	11770 [11447, 12074]	14109 [9144, 14389]	6478 [6360, 6596]	7020 [6826, 7200]	2249 [1226, 4521]	4338 [3549, 4790]	3236 [3068, 3633]	1274 [1179, 1354]	3411 [3056, 4045]	17000 [16694, 17330]	3909 [1554, 8421]	832 [705, 996]
80%	80%	80%	Vision loss	5423 [4308, 6147]	6113 [5923, 6268]	2505 [1658, 6753]	5210 [4899, 5684]	11770 [11447, 12074]	14073 [9144, 14353]	6475 [6354, 6591]	7019 [6823, 7198]	2249 [1226, 4521]	4338 [3549, 4788]	3236 [3068, 3633]	1274 [1179, 1354]	3411 [3056, 4045]	16985 [16669, 17315]	3909 [1554, 8421]	832 [705, 996]

Appendix Table 7: Survey response rates.

Country	Survey type	Year(s) of data collection	Response rate (%)*
Algeria	STEPS	2016-17	93.8
Azerbaijan	STEPS	2017	97.3
Belarus	STEPS	2016-17	87.1
Belize	CAMDI	2005-06	67.0
Benin	STEPS	2015	98.6
Bhutan	STEPS	2014	93.5
Botswana	STEPS	2014	64.0
Burkina Faso	STEPS	2013	98.7
Chile	National Health Survey	2009-10	85.0
Costa Rica	STEPS	2010	87.8
Eswatini	STEPS	2014	70.0
Guyana	STEPS	2016	50.0
Iran	STEPS	2016	~99
Iraq	STEPS	2015	98.8
Kiribati	STEPS	2015-16	55.0
Kyrgyzstan	STEPS	2013	100
Lebanon	STEPS	2017	~70
Moldova	STEPS	2013	83.5
Mongolia	STEPS	2013	97.4
Morocco	STEPS	2017	89.0
Myanmar	STEPS	2014	90.0
Seychelles	National NCD Survey	2013	73.0
Solomon Islands	STEPS	2015	58.4
Sri Lanka	STEPS	2014-15	72.0
St. Vincent and the Grenadines	STEPS	2013	67.8
Sudan	STEPS	2016	88.0
Tajikistan	STEPS	2016-17	94.0
Timor-Leste	STEPS	2014	96.3
Togo	STEPS	2010-11	91.0
Tuvalu	STEPS	2015	76.0
Uganda	STEPS	2014	92.2
Vietnam	STEPS	2015	79.8
Zambia	STEPS	2017	65.0

* Refers to the response rate for biochemical measurements, when reported. CAMDI=Central America Diabetes Initiative; NCD=Non-communicable diseases; STEPS= The WHO STEPwise approach to noncommunicable disease risk factor surveillance.

Appendix Table 8: Diabetes biomarker devices by country

**Diabetes Biomarker**	**Country**	**Post Hoc Adjustment***
Point-of-care fasting capillary glucose		
Accu-check	Tuvalu	None
Accutrend® Plus (Roche, Basel, Switzerland)	Chile, Guyana, Togo	Multiplied by 1.11
CardioCheck® PA (pts Diagnostics, Indianapolis, Indiana, USA)	Belarus, Benin, Bhutan, Burkina Faso, Eswatini, Kiribati, Moldova, Morocco, Solomon Islands, Sri Lanka, St. Vincent & The Grenadines, Timor-Leste, Sudan, Uganda, Vietnam, Zambia	None
Konelab 30i, Thermo, Vanta, Finland	Seychelles	None
SD LipidoCare Analyzer (automatic plasma equivalent)	Myanmar	None
Prima home test	Mongolia	None
Unknown	Algeria, Azerbaijan, Botswana, Kyrgyzstan, Tajikistan	None
Laboratory-based Assessment of Fasting Plasma Glucose		
Central laboratory was used for processing	Belize, Lebanon	N/A
Cobas 6000 and C311 analyzer (Roche Diagnostics, Indianapolis, Indiana, USA)	Iran	N/A
Enzymatic assay (glucose oxidase)	Iraq	N/A
SYNCHRON® System (Beckman Coulter, Inc., Miami, Florida, USA)	Costa Rica	N/A
Hemoglobin A1c (HbA1c)		
Plasma sample by Cobas C311 auto-analyzer (Roche kits)	Iran	N/A
Unknown	Guyana	N/A
Bayer A1cNow	Seychelles	N/A

*Post hoc adjustment to convert from capillary to plasma equivalents. N/A=Not available.

Appendix Table 9: Blood pressure measurement devices by country

Country	Measurement device	Number of measurements	Interval between measurements
Algeria	No report available	No report available	No report available
Azerbaijan	Riester Ri-Champion Automatic Digital Monitor- 1715	3	10 minutes
Belarus	Boso-Medicus Uno	3	3 minutes
Belize	Omron Digital Blood Pressure Monitor HEM-712C	3	5 minutes
Benin	Boso Medicus Uno	3	3 minutes
Bhutan	Boso Medicus Control	3	3 minutes
Botswana	Not specified	Not specified	Not specified
Burkina Faso	Omron Digital Monitor HEM-705CP	3	10 minutes
Chile	Omron Digital Monitor HEM-742	3	2 minutes
Costa Rica	Digital upper arm meter (Welch Allyn and Omron, models not specified)	2 with a third measurement if the first two differed by >10 mmHg	5 minutes
Eswatini	Boso Medicus PC (model not specified)	3	3-5 minutes
Guyana	Omron digital upper arm meter (model not specified)	3	3 minutes
Iran[2,3]	Beurer BM 20	3	5
Iraq	Not specified	Not specified	Not specified
Kiribati	OMRON M4 Digital Automatic Blood Pressure Monitor	3	2-3 minutes
Kyrgyzstan	No report available	No report available	No report available
Lebanon	Manual mercury sphygmomanometer	2	5 minutes
Moldova	Boso-Medicus Uno	3	3 minutes

Mongolia	Omron M5 Digital Monitor	3	3 minutes
Morocco	Spengler ES 60	3	“a few minutes”
Myanmar	Boso-Medicus automatic digital blood pressure monitor (model not specified)	3	3 minutes
Seychelles	Omron M3 Digital Monitor	5	Once upon arrival, three times during the interview, and once before leaving
Solomon Islands	No report available	No report available	No report available
Sri Lanka	Not specified	Not specified	Not specified
St. Vincent & the Grenadines	Omron Digital Monitor M4 - I	3	3 minutes
Sudan	Boso-Medicus Uno	3	3 minutes
Tajikistan	No report available	No report available	No report available
Timor-Leste	Omron digital upper arm meter (model not specified)	3	2 minutes
Togo	Omron digital upper arm meter (model not specified)	3	5 minutes
Tuvalu	No report available	No report available	No report available
Uganda	Boso Medicus Uno	3	3-5 minutes
Vietnam	BOSO Device	Not specified	Not Specified
Zambia	Not specified	3	3-5 minutes

Appendix Table 10: Cholesterol measurement devices by country

Measurement device	Countries
Accutrend Plus	Tuvalu
CardioCheck PA	Belarus, Benin, Bhutan, Burkina Faso, Eswatini, Kiribati, Moldova, Morocco, Solomon Islands, Sri Lanka, St. Vincent & the Grenadines, Sudan, Timor-Leste, Vietnam, Zambia
Konelab 30i	Seychelles
Laboratory	Belize, Chile, Costa Rica, Guyana, Iran, Iraq, Lebanon
Prima Home Test	Mongolia
SD LipidoCare Analyzer	Myanmar
Unknown	Algeria, Azerbaijan, Botswana, Kyrgyzstan, Tajikistan

Appendix Figure 1: Estimated frequency of cardiovascular disease (CVD) events without new screening but with increased treatment and control. The figure displays increases in treatment (trt) with blood pressure, glycaemic, or statin medicines to 60% or 80% of those diagnosed with hypertension or diabetes or indicated for statins (age 40 or older or >20% estimated 10-year risk of cardiovascular events), respectively; and control (ctrl) of blood pressure (systolic pressure <130 mmHg and diastolic pressure <80 mmHg) or glycaemia (haemoglobin A1c ≤7% or fasting plasma glucose <7 mmol/L [126 mg/dL]) to 60% or 80% of those treated for hypertension or diabetes, respectively. Cardiovascular disease was defined as fatal and non-fatal myocardial infarction and stroke. Region abbreviations: CAR = Caribbean; CASIA = Central Asia; CLA = Central Latin America; EEUR = Eastern Europe; ESSA = Eastern Sub-Saharan Africa; NAME = North Africa/Middle East; OCN = Oceania; SASIA = South Asia; SEASIA = Southeast Asia; SLA = South Latin America; SSSA = South Sub-Saharan Africa; WSSA = Western Sub-Saharan Africa. In this and subsequent figures, the uncertainty is due to sampling, where the sample is all persons in the region (survey sample weighted).

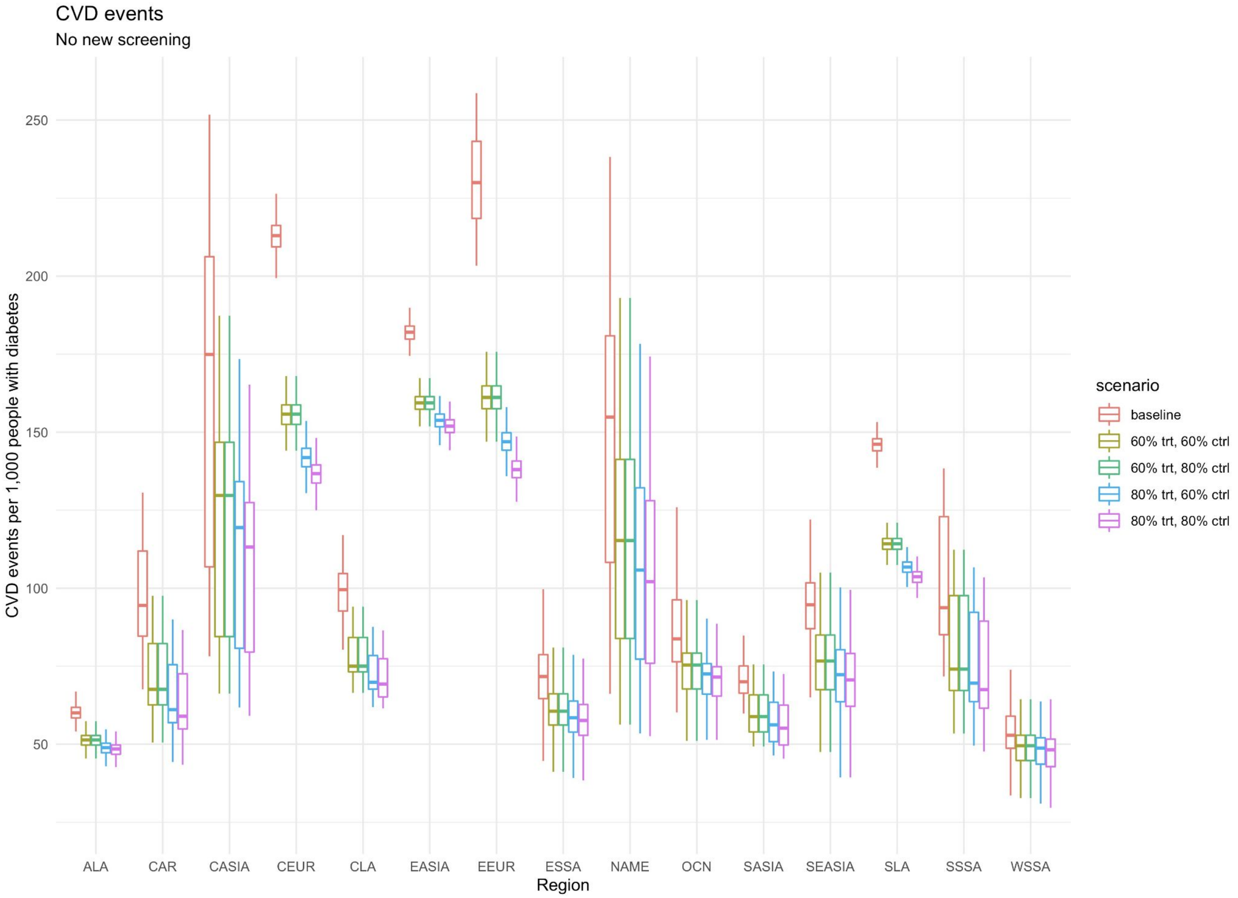
CVD events
No new screening
CVD events per 1,000 people with diabetes
250
200
150
100
50
ALA
CAR
CASIA
CEUR
CLA
EASIA
EEUR
ESSA
NAME
OCN
SASIA
SEASIA
SLA
SSSA
WSSA
Region
scenario
baseline
60% trt, 60% ctrl
60% trt, 80% ctrl
80% trt, 60% ctrl
80% trt, 80% ctrl

Appendix Figure 2: Estimated frequency of cardiovascular disease (CVD) events with screening for 60% diagnosis and with increased treatment and control. We brought the proportion of people with diabetes who were diagnosed up to 60% within each country's population, and the portion of those with diabetes and hypertension who were diagnosed with hypertension up to 60%, leaving unaltered those countries with a baseline level above these proportions The figure displays increases in treatment (trt) with blood pressure, glycaemic, or statin medicines to 60% or 80% of those diagnosed with hypertension or diabetes or indicated for statins (age 40 or older or >20% estimated 10-year risk of cardiovascular events), respectively; and control (ctrl) of blood pressure (systolic pressure <130 mmHg and diastolic pressure <80 mmHg) or glycaemia (haemoglobin A1c ≤7% or fasting plasma glucose <7 mmol/L [126 mg/dL]) to 60% or 80% of those treated for hypertension or diabetes, respectively. Cardiovascular disease was defined as fatal and non-fatal myocardial infarction and stroke. Region abbreviations: CAR = Caribbean; CASIA = Central Asia; CLA = Central Latin America; EEUR = Eastern Europe; ESSA = Eastern Sub-Saharan Africa; NAME = North Africa/Middle East; OCN = Oceania; SASIA = South Asia; SEASIA = Southeast Asia; SLA = South Latin America; SSSA = South Sub-Saharan Africa; WSSA = Western Sub-Saharan Africa.

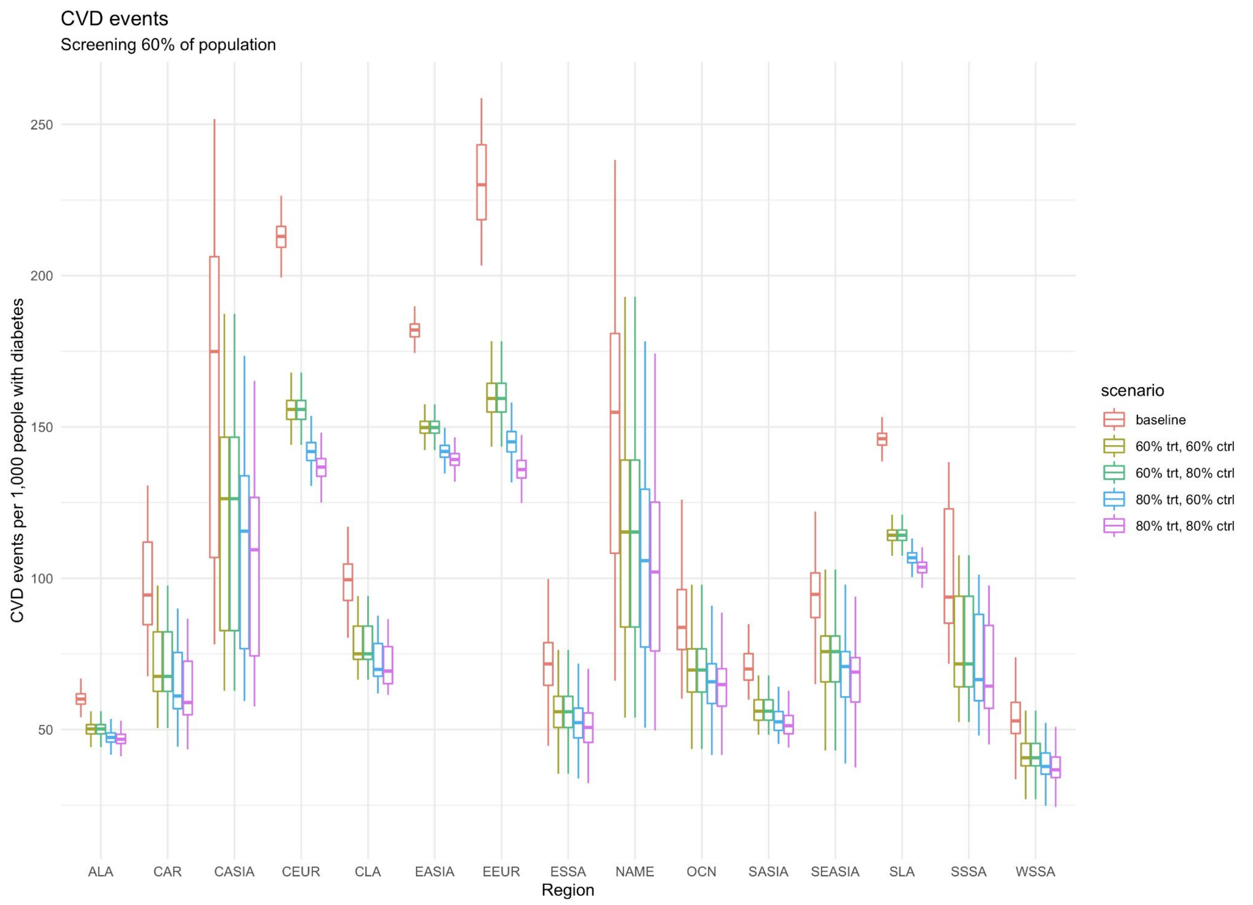

CVD events
Screening 60% of population
CVD events per 1,000 people with diabetes
250
200
150
100
50
ALA
CAR
CASIA
CEUR
CLA
EASIA
EEUR
ESSA
NAME
OCN
SASIA
SEASIA
SLA
SSSA
WSSA
Region
scenario
baseline
60% trt, 60% ctrl
60% trt, 80% ctrl
80% trt, 60% ctrl
80% trt, 80% ctrl

Appendix Figure 3: Estimated frequency of cardiovascular disease (CVD) events with screening for 80% diagnosis and with increased treatment and control. We brought the proportion of people with diabetes who were diagnosed up to 80% within each country's population, and the portion of those with diabetes and hypertension who were diagnosed with hypertension up to 80%, leaving unaltered those countries with a baseline level above these proportions The figure displays increases in treatment (trt) with blood pressure, glycaemic, or statin medicines to 60% or 80% of those diagnosed with hypertension or diabetes or indicated for statins (age 40 or older or >20% estimated 10-year risk of cardiovascular events), respectively; and control (ctrl) of blood pressure (systolic pressure <130 mmHg and diastolic pressure <80 mmHg) or glycaemia (haemoglobin A1c ≤7% or fasting plasma glucose <7 mmol/L [126 mg/dL]) to 60% or 80% of those treated for hypertension or diabetes, respectively. Cardiovascular disease was defined as fatal and non-fatal myocardial infarction and stroke. Region abbreviations: CAR = Caribbean; CASIA = Central Asia; CLA = Central Latin America; EEUR = Eastern Europe; ESSA = Eastern Sub-Saharan Africa; NAME = North Africa/Middle East; OCN = Oceania; SASIA = South Asia; SEASIA = Southeast Asia; SLA = South Latin America; SSSA = South Sub-Saharan Africa; WSSA = Western Sub-Saharan Africa.

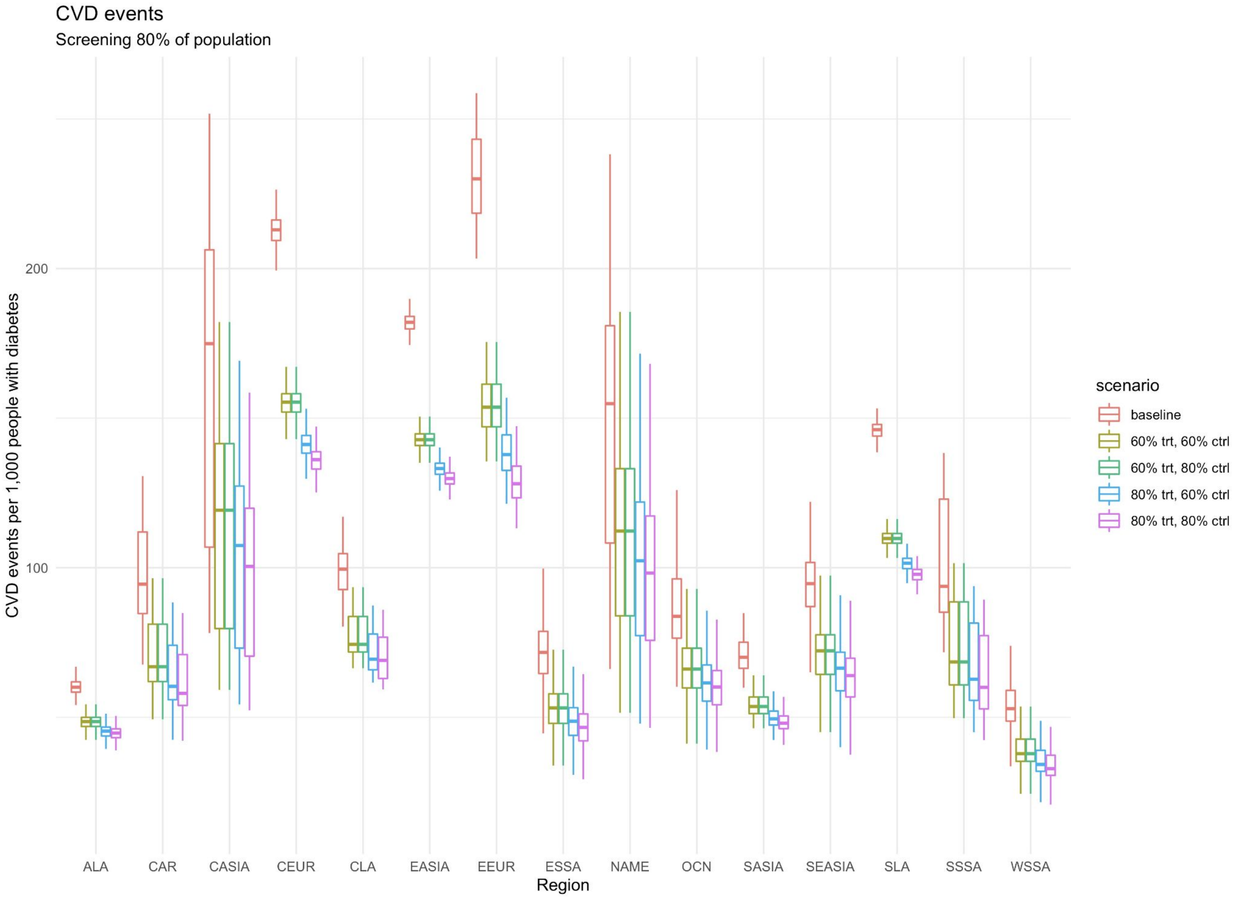
CVD events
Screening 80% of population
CVD events per 1,000 people with diabetes
200
100
ALA
CAR
CASIA
CEUR
CLA
EASIA
EEUR
ESSA
NAME
OCN
SASIA
SEASIA
SLA
SSSA
WSSA
Region
scenario
baseline
60% trt, 60% ctrl
60% trt, 80% ctrl
80% trt, 60% ctrl
80% trt, 80% ctrl

Appendix Figure 4: Estimated frequency of incident congestive heart failure (CHF) events without new screening but with increased treatment and control. The figure displays increases in treatment (trt) with blood pressure, glycaemic, or statin medicines to 60% or 80% of those diagnosed with hypertension or diabetes or indicated for statins (age 40 or older or >20% estimated 10-year risk of cardiovascular events), respectively; and control (ctrl) of blood pressure (systolic pressure <130 mmHg and diastolic pressure <80 mmHg) or glycaemia (haemoglobin A1c ≤7% or fasting plasma glucose <7 mmol/L [126 mg/dL]) to 60% or 80% of those treated for hypertension or diabetes, respectively. Congestive heart failure was defined as an ejection fraction of <40%, with New York Heart Association class III or IV functional limitations. Region abbreviations: CAR = Caribbean; CASIA = Central Asia; CLA = Central Latin America; EEUR = Eastern Europe; ESSA = Eastern Sub-Saharan Africa; NAME = North Africa/Middle East; OCN = Oceania; SASIA = South Asia; SEASIA = Southeast Asia; SLA = South Latin America; SSSA = South Sub-Saharan Africa; WSSA = Western Sub-Saharan Africa.

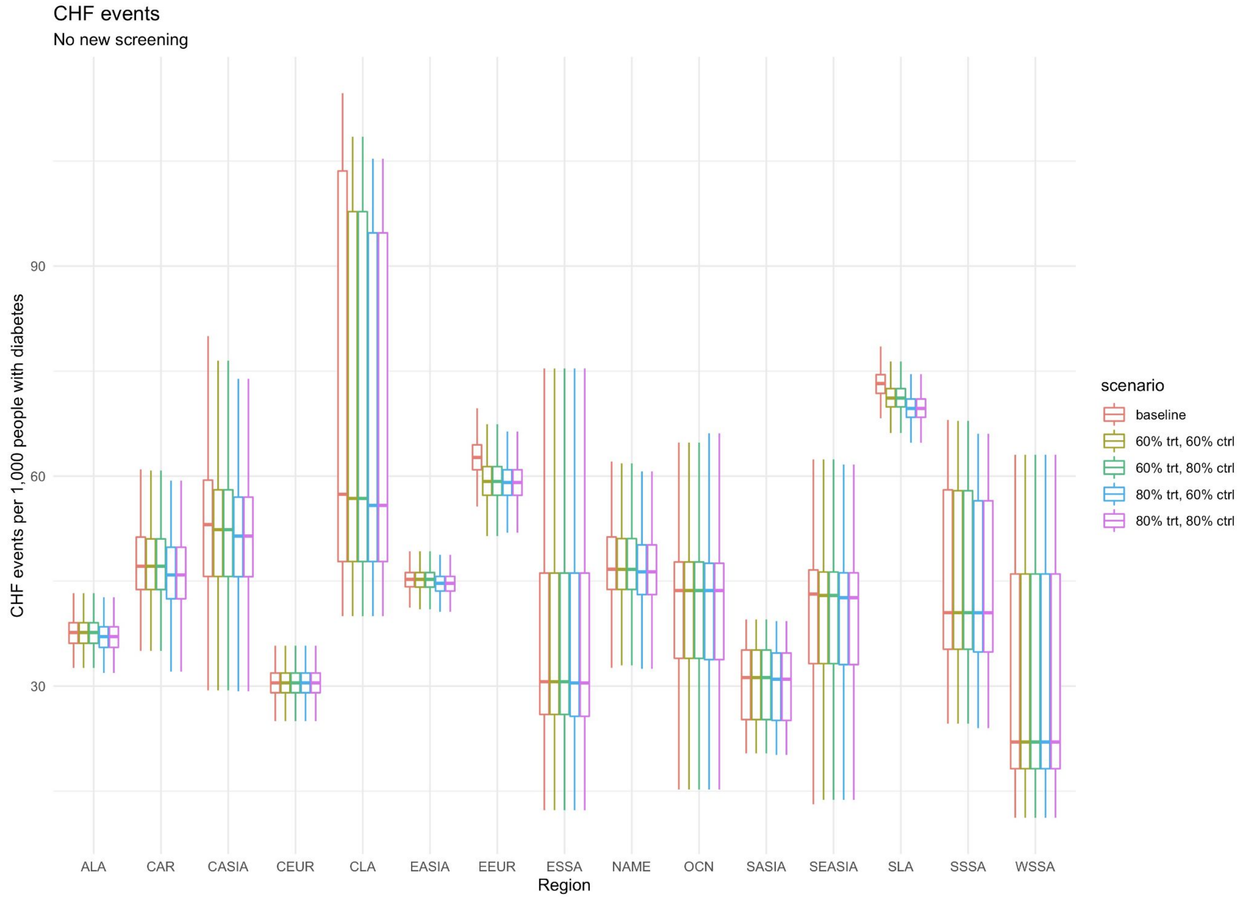
CHF events
No new screening
CHF events per 1,000 people with diabetes
90
60
30
ALA
CAR
CASIA
CEUR
CLA
EASIA
EEUR
ESSA
NAME
OCN
SASIA
SEASIA
SLA
SSSA
WSSA
Region
scenario
baseline
60% trt, 60% ctrl
60% trt, 80% ctrl
80% trt, 60% ctrl
80% trt, 80% ctrl

Appendix Figure 5: Estimated frequency of incident congestive heart failure (CHF) events with screening for 60% diagnosis and with increased treatment and control. We brought the proportion of people with diabetes who were diagnosed up to 60% within each country's population, and the portion of those with diabetes and hypertension who were diagnosed with hypertension up to 60%, leaving unaltered those countries with a baseline level above these proportions The figure displays increases in treatment (trt) with blood pressure, glycaemic, or statin medicines to 60% or 80% of those diagnosed with hypertension or diabetes or indicated for statins (age 40 or older or >20% estimated 10-year risk of cardiovascular events), respectively; and control (ctrl) of blood pressure (systolic pressure <130 mmHg and diastolic pressure <80 mmHg) or glycaemia (haemoglobin A1c ≤7% or fasting plasma glucose <7 mmol/L [126 mg/dL]) to 60% or 80% of those treated for hypertension or diabetes, respectively. Congestive heart failure was defined as an ejection fraction of <40%, with New York Heart Association class III or IV functional limitations. Region abbreviations: CAR = Caribbean; CASIA = Central Asia; CLA = Central Latin America; EEUR = Eastern Europe; ESSA = Eastern Sub-Saharan Africa; NAME = North Africa/Middle East; OCN = Oceania; SASIA = South Asia; SEASIA = Southeast Asia; SLA = South Latin America; SSSA = South Sub-Saharan Africa; WSSA = Western Sub-Saharan Africa.

CHF events

Screening 60% of population

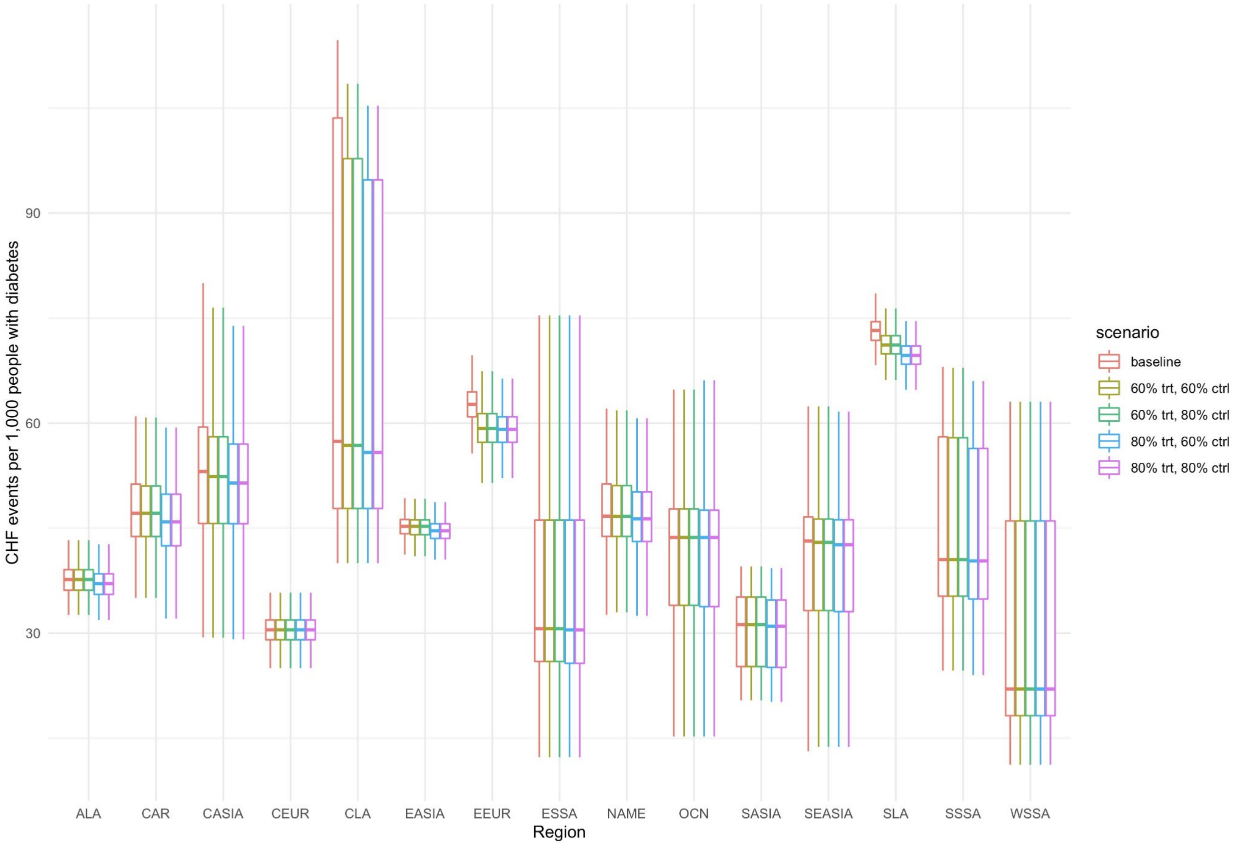

Appendix Figure 6: Estimated frequency of incident congestive heart failure (CHF) events with screening for 80% diagnosis and with increased treatment and control. We brought the proportion of people with diabetes who were diagnosed up to 80% within each country's population, and the portion of those with diabetes and hypertension who were diagnosed with hypertension up to 80%, leaving unaltered those countries with a baseline level above these proportions The figure displays increases in treatment (trt) with blood pressure, glycaemic, or statin medicines to 60% or 80% of those diagnosed with hypertension or diabetes or indicated for statins (age 40 or older or >20% estimated 10-year risk of cardiovascular events), respectively; and control (ctrl) of blood pressure (systolic pressure <130 mmHg and diastolic pressure <80 mmHg) or glycaemia (haemoglobin A1c ≤7% or fasting plasma glucose <7 mmol/L [126 mg/dL]) to 60% or 80% of those treated for hypertension or diabetes, respectively. Congestive heart failure was defined as an ejection fraction of <40%, with New York Heart Association class III or IV functional limitations. Region abbreviations: CAR = Caribbean; CASIA = Central Asia; CLA = Central Latin America; EEUR = Eastern Europe; ESSA = Eastern Sub-Saharan Africa; NAME = North Africa/Middle East; OCN = Oceania; SASIA = South Asia; SEASIA = Southeast Asia; SLA = South Latin America; SSSA = South Sub-Saharan Africa; WSSA = Western Sub-Saharan Africa.

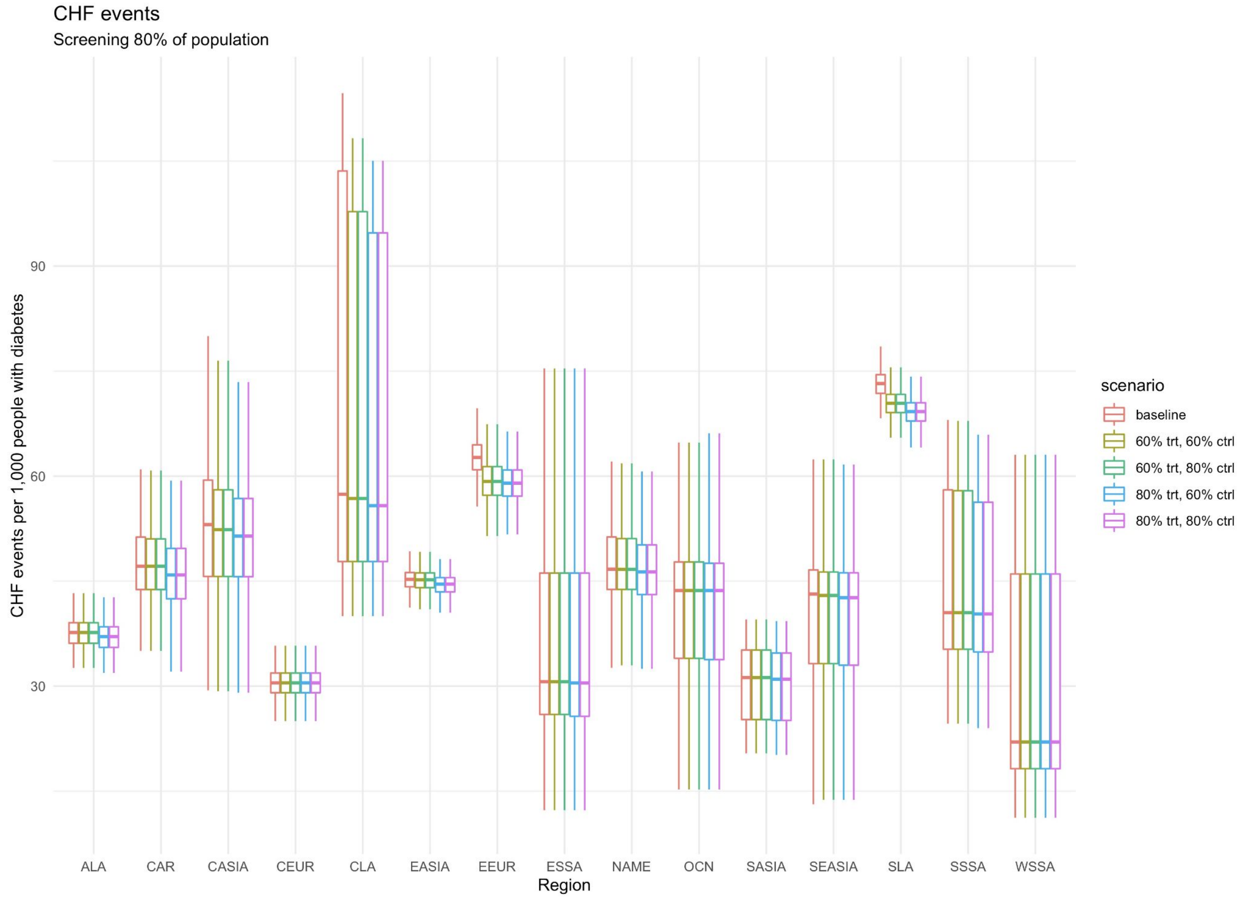
CHF events
Screening 80% of population
CHF events per 1,000 people with diabetes
90
60
30
ALA
CAR
CASIA
CEUR
CLA
EASIA
EEUR
ESSA
NAME
OCN
SASIA
SEASIA
SLA
SSSA
WSSA
Region
scenario
baseline
60% trt, 60% ctrl
60% trt, 80% ctrl
80% trt, 60% ctrl
80% trt, 80% ctrl

Appendix Figure 7: Estimated frequency of incident end-stage renal disease (ESRD) events without new screening but with increased treatment and control. The figure displays increases in treatment (trt) with blood pressure, glycaemic, or statin medicines to 60% or 80% of those diagnosed with hypertension or diabetes or indicated for statins (age 40 or older or >20% estimated 10-year risk of cardiovascular events), respectively; and control (ctrl) of blood pressure (systolic pressure <130 mmHg and diastolic pressure <80 mmHg) or glycaemia (haemoglobin A1c ≤7% or fasting plasma glucose <7 mmol/L [126 mg/dL]) to 60% or 80% of those treated for hypertension or diabetes, respectively. End-stage renal disease (ESRD) was defined as estimated glomerular filtration rate <15 mL/min/1.73m^2 or needing dialysis/transplant. Region abbreviations: CAR = Caribbean; CASIA = Central Asia; CLA = Central Latin America; EEUR = Eastern Europe; ESSA = Eastern Sub-Saharan Africa; NAME = North Africa/Middle East; OCN = Oceania; SASIA = South Asia; SEASIA = Southeast Asia; SLA = South Latin America; SSSA = South Sub-Saharan Africa; WSSA = Western Sub-Saharan Africa.

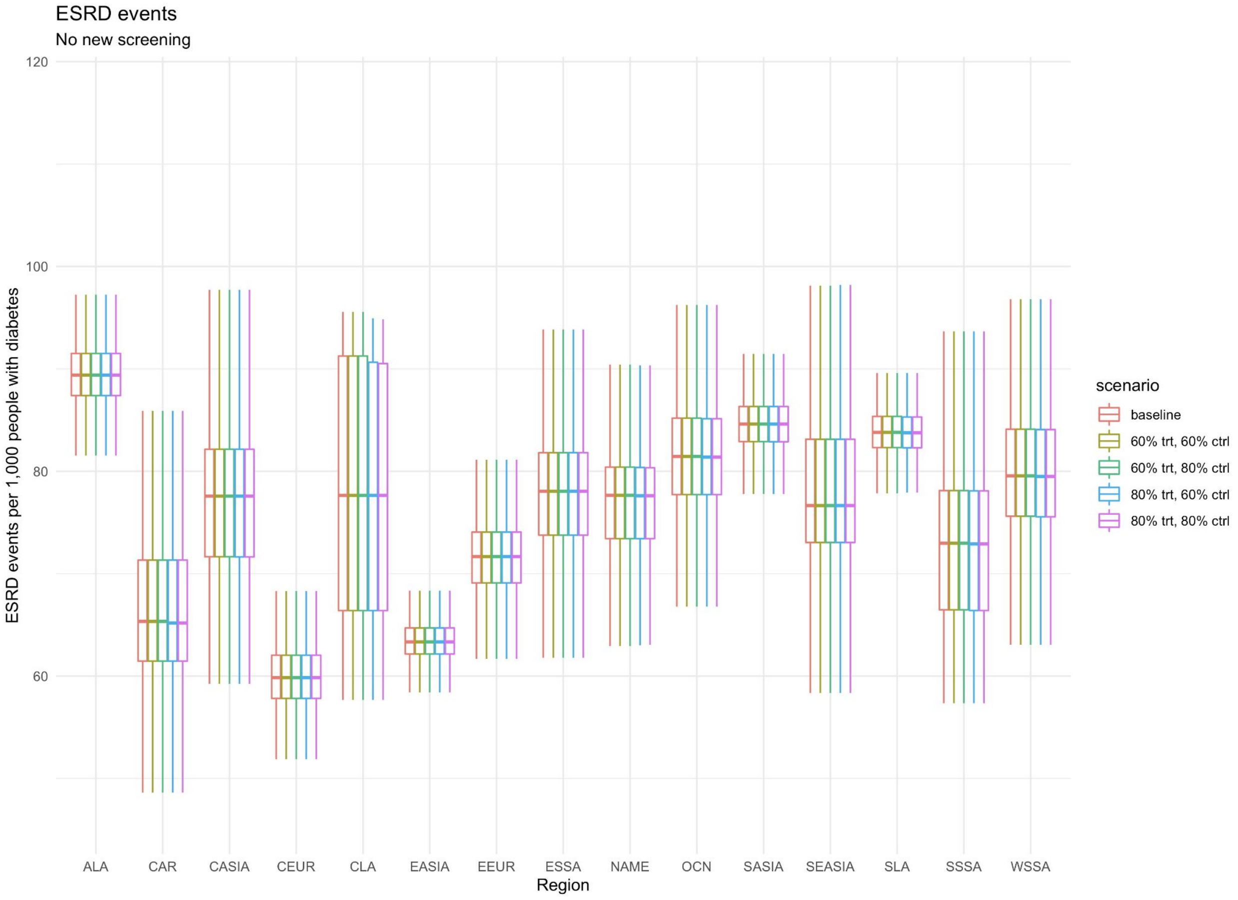

Appendix Figure 8: Estimated frequency of incident end-stage renal disease (ESRD) events with screening for 60% diagnosis and with increased treatment and control. We brought the proportion of people with diabetes who were diagnosed up to 60% within each country's population, and the portion of those with diabetes and hypertension who were diagnosed with hypertension up to 60%, leaving unaltered those countries with a baseline level above these proportions The figure displays increases in treatment (trt) with blood pressure, glycaemic, or statin medicines to 60% or 80% of those diagnosed with hypertension or diabetes or indicated for statins (age 40 or older or >20% estimated 10-year risk of cardiovascular events), respectively; and control (ctrl) of blood pressure (systolic pressure <130 mmHg and diastolic pressure <80 mmHg) or glycaemia (haemoglobin A1c ≤7% or fasting plasma glucose <7 mmol/L [126 mg/dL]) to 60% or 80% of those treated for hypertension or diabetes, respectively. End-stage renal disease (ESRD) was defined as estimated glomerular filtration rate <15 mL/min/1.73m^2 or needing dialysis/transplant. Region abbreviations: CAR = Caribbean; CASIA = Central Asia; CLA = Central Latin America; EEUR = Eastern Europe; ESSA = Eastern Sub-Saharan Africa; NAME = North Africa/Middle East; OCN = Oceania; SASIA = South Asia; SEASIA = Southeast Asia; SLA = South Latin America; SSSA = South Sub-Saharan Africa; WSSA = Western Sub-Saharan Africa.

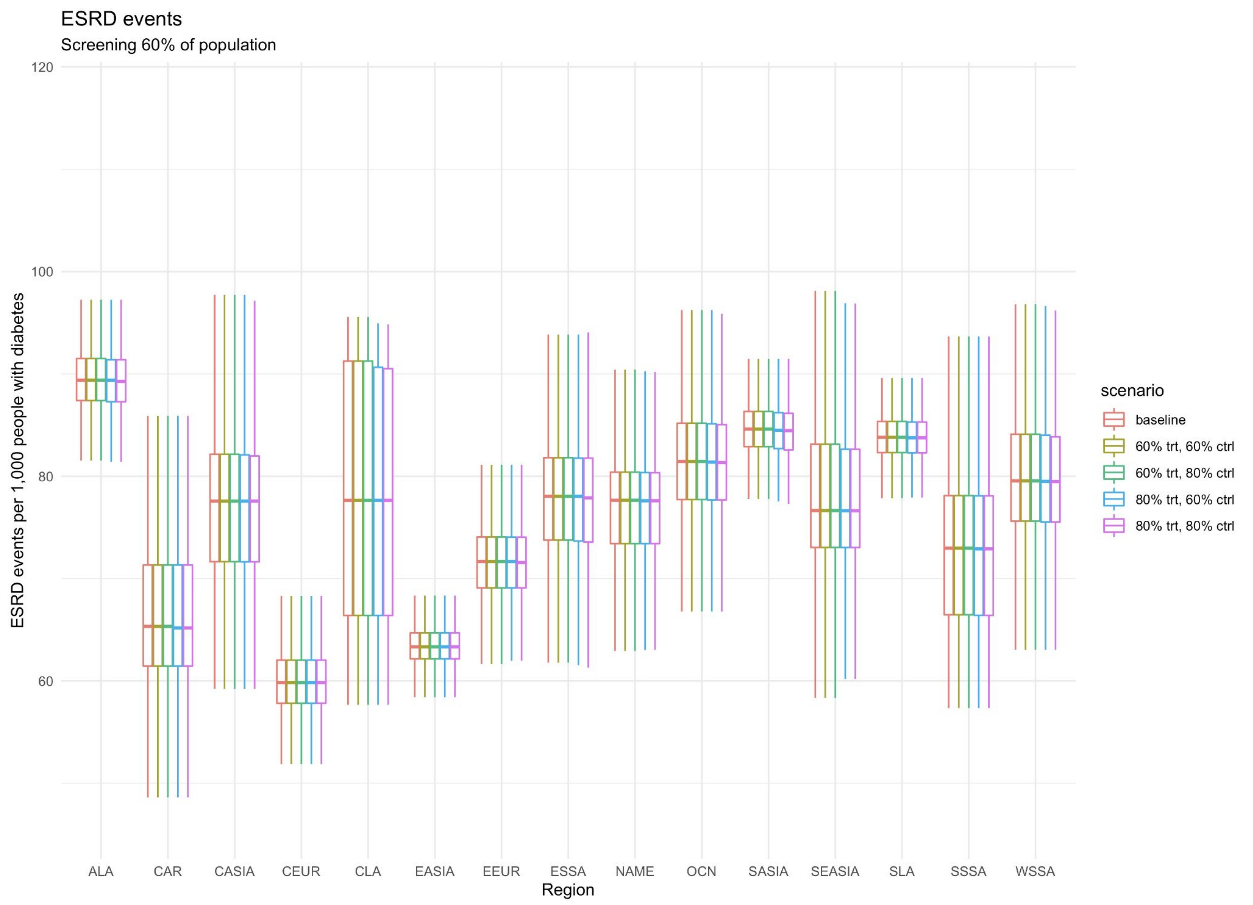
ESRD events
Screening 60% of population
ESRD events per 1,000 people with diabetes
120
100
80
60
ALA
CAR
CASIA
CEUR
CLA
EASIA
EEUR
ESSA
NAME
OCN
SASIA
SEASIA
SLA
SSSA
WSSA
Region
scenario
baseline
60% trt, 60% ctrl
60% trt, 80% ctrl
80% trt, 60% ctrl
80% trt, 80% ctrl

Appendix Figure 9: Estimated frequency of incident end-stage renal disease (ESRD) events with screening for 80% diagnosis and with increased treatment and control. We brought the proportion of people with diabetes who were diagnosed up to 80% within each country's population, and the portion of those with diabetes and hypertension who were diagnosed with hypertension up to 80%, leaving unaltered those countries with a baseline level above these proportions The figure displays increases in treatment (trt) with blood pressure, glycaemic, or statin medicines to 60% or 80% of those diagnosed with hypertension or diabetes or indicated for statins (age 40 or older or >20% estimated 10-year risk of cardiovascular events), respectively; and control (ctrl) of blood pressure (systolic pressure <130 mmHg and diastolic pressure <80 mmHg) or glycaemia (haemoglobin A1c ≤7% or fasting plasma glucose <7 mmol/L [126 mg/dL]) to 60% or 80% of those treated for hypertension or diabetes, respectively. End-stage renal disease (ESRD) was defined as estimated glomerular filtration rate <15 mL/min/1.73m^2 or needing dialysis/transplant. Region abbreviations: CAR = Caribbean; CASIA = Central Asia; CLA = Central Latin America; EEUR = Eastern Europe; ESSA = Eastern Sub-Saharan Africa; NAME = North Africa/Middle East; OCN = Oceania; SASIA = South Asia; SEASIA = Southeast Asia; SLA = South Latin America; SSSA = South Sub-Saharan Africa; WSSA = Western Sub-Saharan Africa.

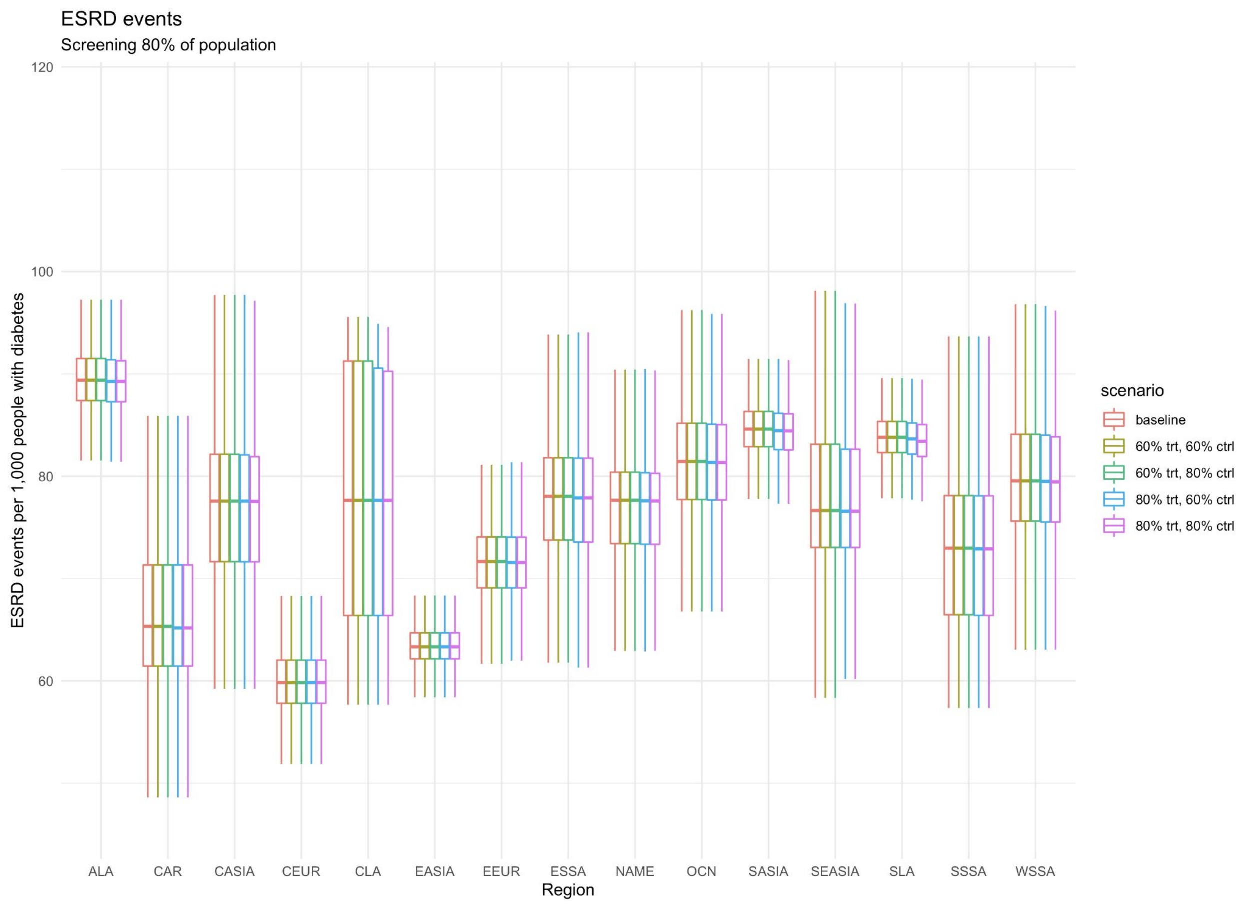
ESRD events
Screening 80% of population
ESRD events per 1,000 people with diabetes
120
100
80
60
ALA
CAR
CASIA
CEUR
CLA
EASIA
EEUR
ESSA
NAME
OCN
SASIA
SEASIA
SLA
SSSA
WSSA
Region
scenario
baseline
60% trt, 60% ctrl
60% trt, 80% ctrl
80% trt, 60% ctrl
80% trt, 80% ctrl

Appendix Figure 10: Estimated frequency of incident neuropathy events without new screening but with increased treatment and control. The figure displays increases in treatment (trt) with blood pressure, glycaemic, or statin medicines to 60% or 80% of those diagnosed with hypertension or diabetes or indicated for statins (age 40 or older or >20% estimated 10-year risk of cardiovascular events), respectively; and control (ctrl) of blood pressure (systolic pressure <130 mmHg and diastolic pressure <80 mmHg) or glycaemia (haemoglobin A1c ≤7% or fasting plasma glucose <7 mmol/L [126 mg/dL]) to 60% or 80% of those treated for hypertension or diabetes, respectively. Neuropathy was defined as pressure sensation loss by Semmes-Weinstein 5.07/10 gram monofilament exam. Region abbreviations: CAR = Caribbean; CASIA = Central Asia; CLA = Central Latin America; EEUR = Eastern Europe; ESSA = Eastern Sub-Saharan Africa; NAME = North Africa/Middle East; OCN = Oceania; SASIA = South Asia; SEASIA = Southeast Asia; SLA = South Latin America; SSSA = South Sub-Saharan Africa; WSSA = Western Sub-Saharan Africa.

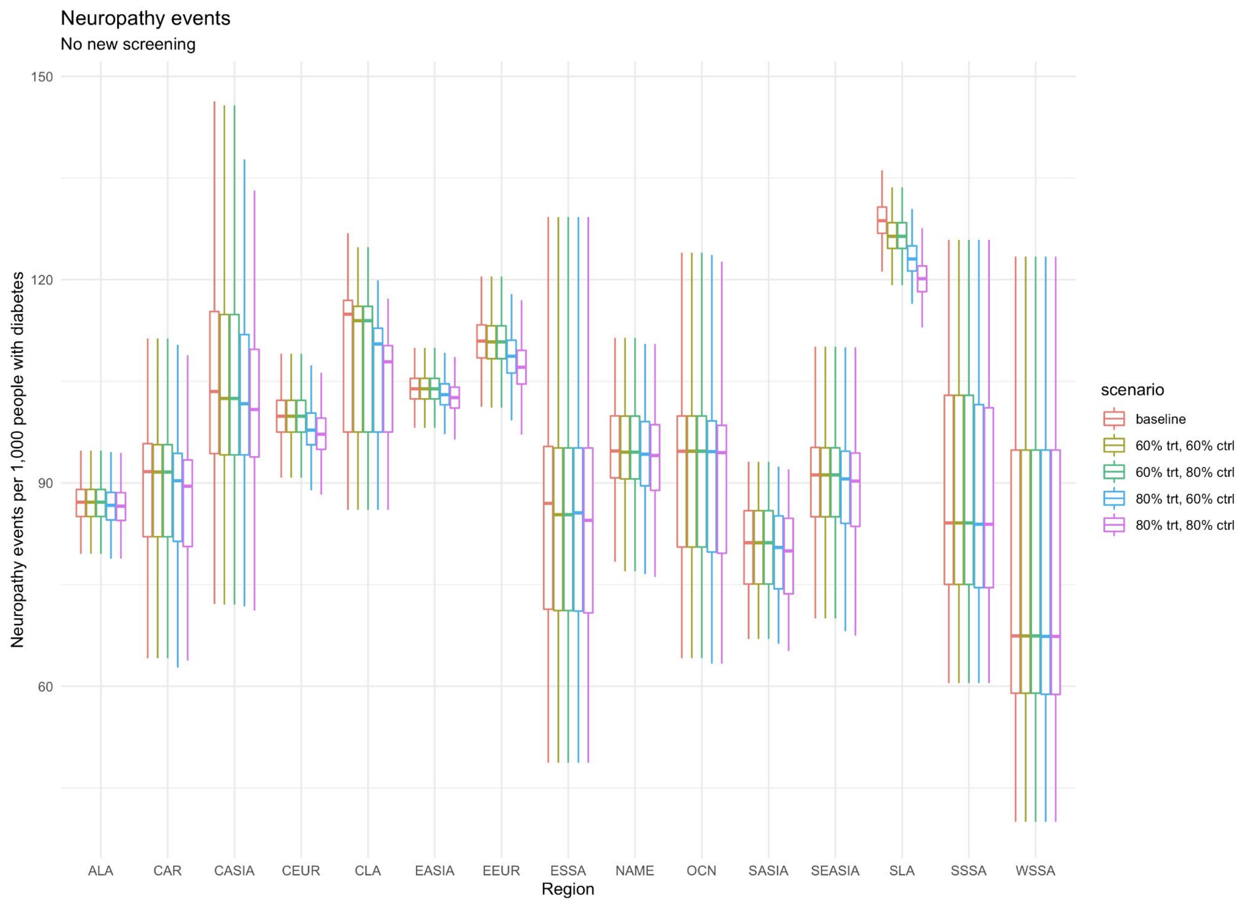

Appendix Figure 11: Estimated frequency of incident neuropathy events with screening for 60% diagnosis and with increased treatment and control. We brought the proportion of people with diabetes who were diagnosed up to 60% within each country's population, and the portion of those with diabetes and hypertension who were diagnosed with hypertension up to 60%, leaving unaltered those countries with a baseline level above these proportions The figure displays increases in treatment (trt) with blood pressure, glycaemic, or statin medicines to 60% or 80% of those diagnosed with hypertension or diabetes or indicated for statins (age 40 or older or >20% estimated 10-year risk of cardiovascular events), respectively; and control (ctrl) of blood pressure (systolic pressure <130 mmHg and diastolic pressure <80 mmHg) or glycaemia (haemoglobin A1c ≤7% or fasting plasma glucose <7 mmol/L [126 mg/dL]) to 60% or 80% of those treated for hypertension or diabetes, respectively. Neuropathy was defined as pressure sensation loss by Semmes-Weinstein 5.07/10 gram monofilament exam. Region abbreviations: CAR = Caribbean; CASIA = Central Asia; CLA = Central Latin America; EEUR = Eastern Europe; ESSA = Eastern Sub-Saharan Africa; NAME = North Africa/Middle East; OCN = Oceania; SASIA = South Asia; SEASIA = Southeast Asia; SLA = South Latin America; SSSA = South Sub-Saharan Africa; WSSA = Western Sub-Saharan Africa.

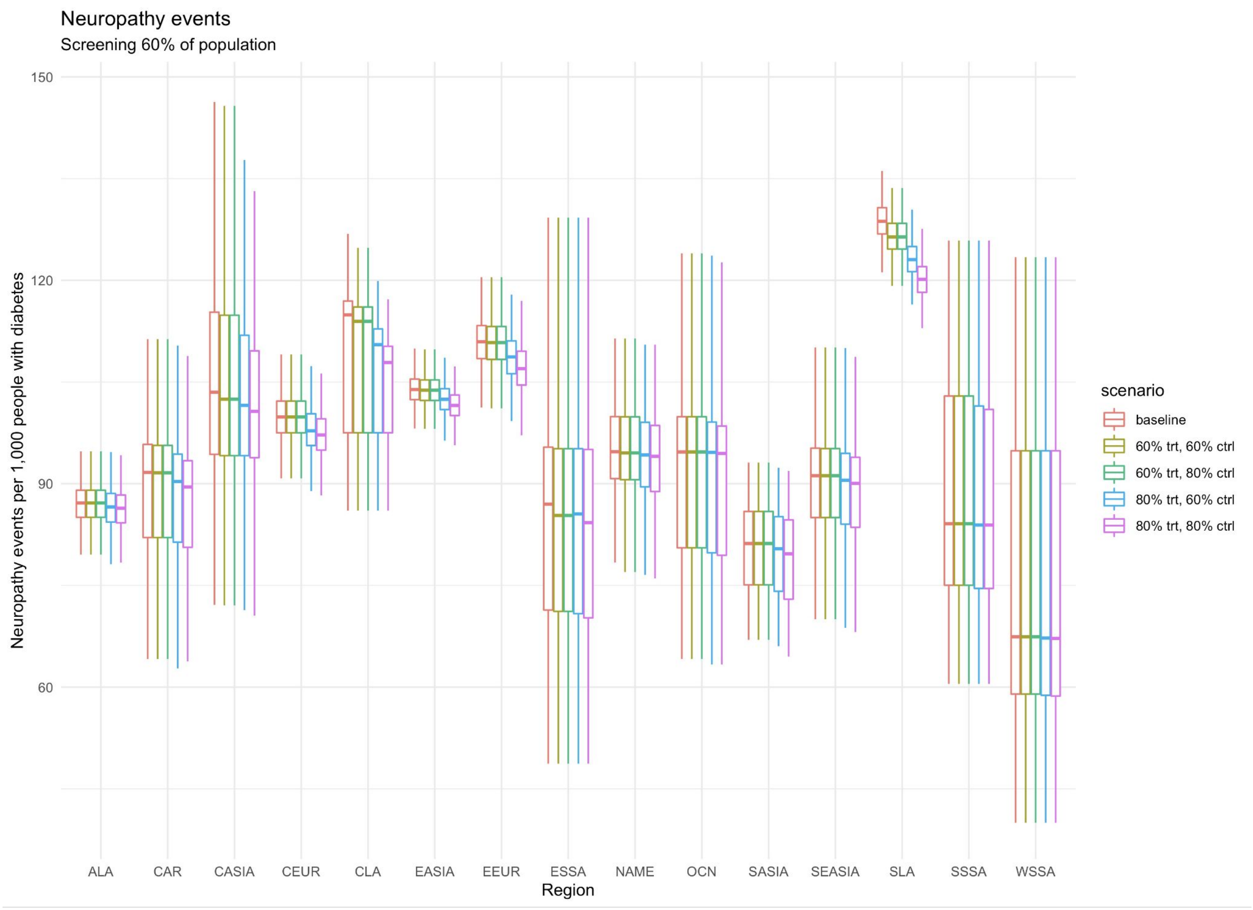

Neuropathy events
Screening 60% of population
Neuropathy events per 1,000 people with diabetes
150
120
90
60
ALA
CAR
CASIA
CEUR
CLA
EASIA
EEUR
ESSA
NAME
OCN
SASIA
SEASIA
SLA
SSSA
WSSA
Region
scenario
baseline
60% trt, 60% ctrl
60% trt, 80% ctrl
80% trt, 60% ctrl
80% trt, 80% ctrl

Appendix Figure 12: Estimated frequency of incident neuropathy events with screening for 80% diagnosis and with increased treatment and control. We brought the proportion of people with diabetes who were diagnosed up to 80% within each country's population, and the portion of those with diabetes and hypertension who were diagnosed with hypertension up to 80%, leaving unaltered those countries with a baseline level above these proportions The figure displays increases in treatment (trt) with blood pressure, glycaemic, or statin medicines to 60% or 80% of those diagnosed with hypertension or diabetes or indicated for statins (age 40 or older or >20% estimated 10-year risk of cardiovascular events), respectively; and control (ctrl) of blood pressure (systolic pressure <130 mmHg and diastolic pressure <80 mmHg) or glycaemia (haemoglobin A1c ≤7% or fasting plasma glucose <7 mmol/L [126 mg/dL]) to 60% or 80% of those treated for hypertension or diabetes, respectively. Neuropathy was defined as pressure sensation loss by Semmes-Weinstein 5.07/10 gram monofilament exam. Region abbreviations: CAR = Caribbean; CASIA = Central Asia; CLA = Central Latin America; EEUR = Eastern Europe; ESSA = Eastern Sub-Saharan Africa; NAME = North Africa/Middle East; OCN = Oceania; SASIA = South Asia; SEASIA = Southeast Asia; SLA = South Latin America; SSSA = South Sub-Saharan Africa; WSSA = Western Sub-Saharan Africa.

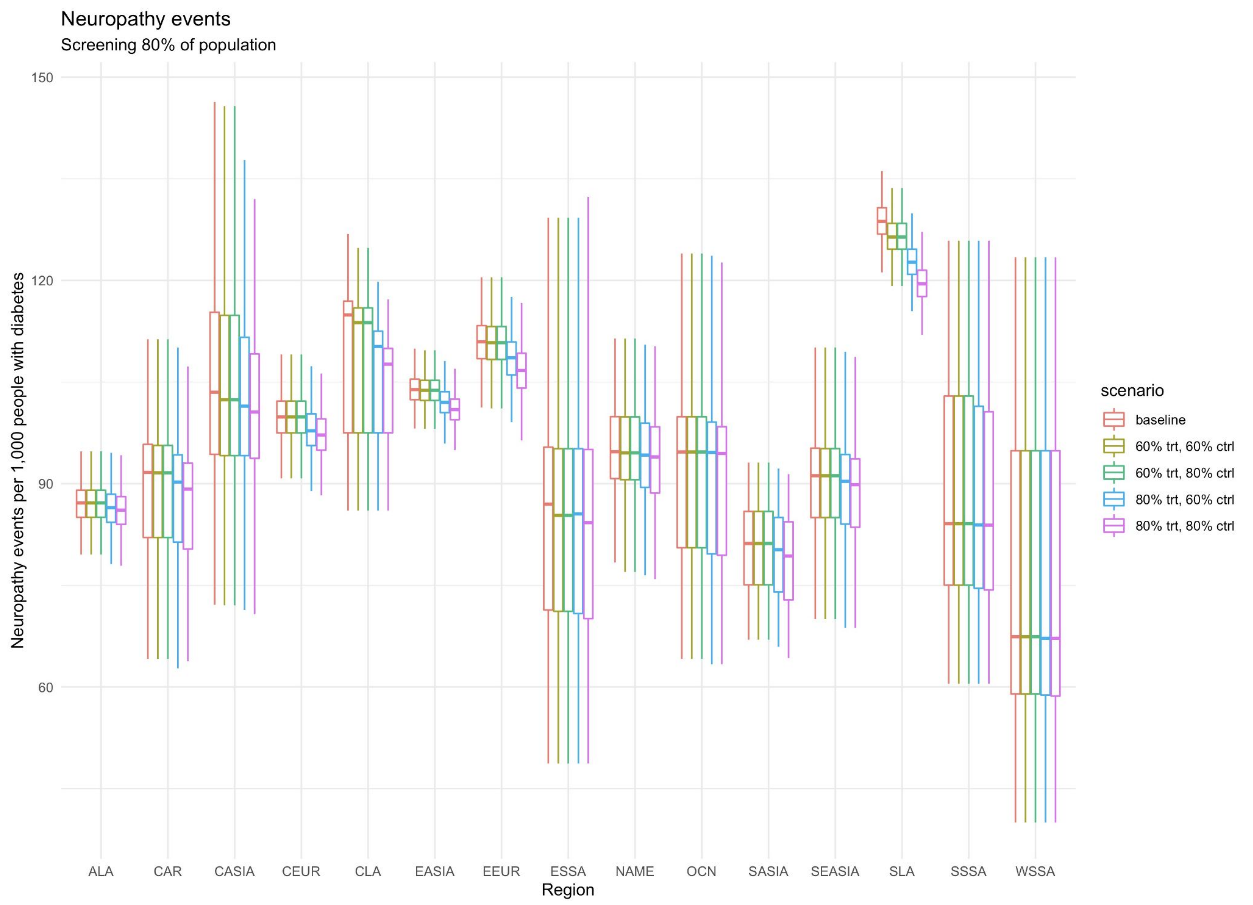

Neuropathy events
Screening 80% of population
Neuropathy events per 1,000 people with diabetes
150
120
90
60
ALA
CAR
CASIA
CEUR
CLA
EASIA
EEUR
ESSA
NAME
OCN
SASIA
SEASIA
SLA
SSSA
WSSA
Region
scenario
baseline
60% trt, 60% ctrl
60% trt, 80% ctrl
80% trt, 60% ctrl
80% trt, 80% ctrl

Appendix Figure 13: Estimated frequency of incident retinopathy events without new screening but with increased treatment and control. The figure displays increases in treatment (trt) with blood pressure, glycaemic, or statin medicines to 60% or 80% of those diagnosed with hypertension or diabetes or indicated for statins (age 40 or older or >20% estimated 10-year risk of cardiovascular events), respectively; and control (ctrl) of blood pressure (systolic pressure <130 mmHg and diastolic pressure <80 mmHg) or glycaemia (haemoglobin A1c ≤7% or fasting plasma glucose <7 mmol/L [126 mg/dL]) to 60% or 80% of those treated for hypertension or diabetes, respectively. The retinopathy end-point was severe vision loss, defined as <20/200 visual acuity by Snellen chart. Region abbreviations: CAR = Caribbean; CASIA = Central Asia; CLA = Central Latin America; EEUR = Eastern Europe; ESSA = Eastern Sub-Saharan Africa; NAME = North Africa/Middle East; OCN = Oceania; SASIA = South Asia; SEASIA = Southeast Asia; SLA = South Latin America; SSSA = South Sub-Saharan Africa; WSSA = Western Sub-Saharan Africa.

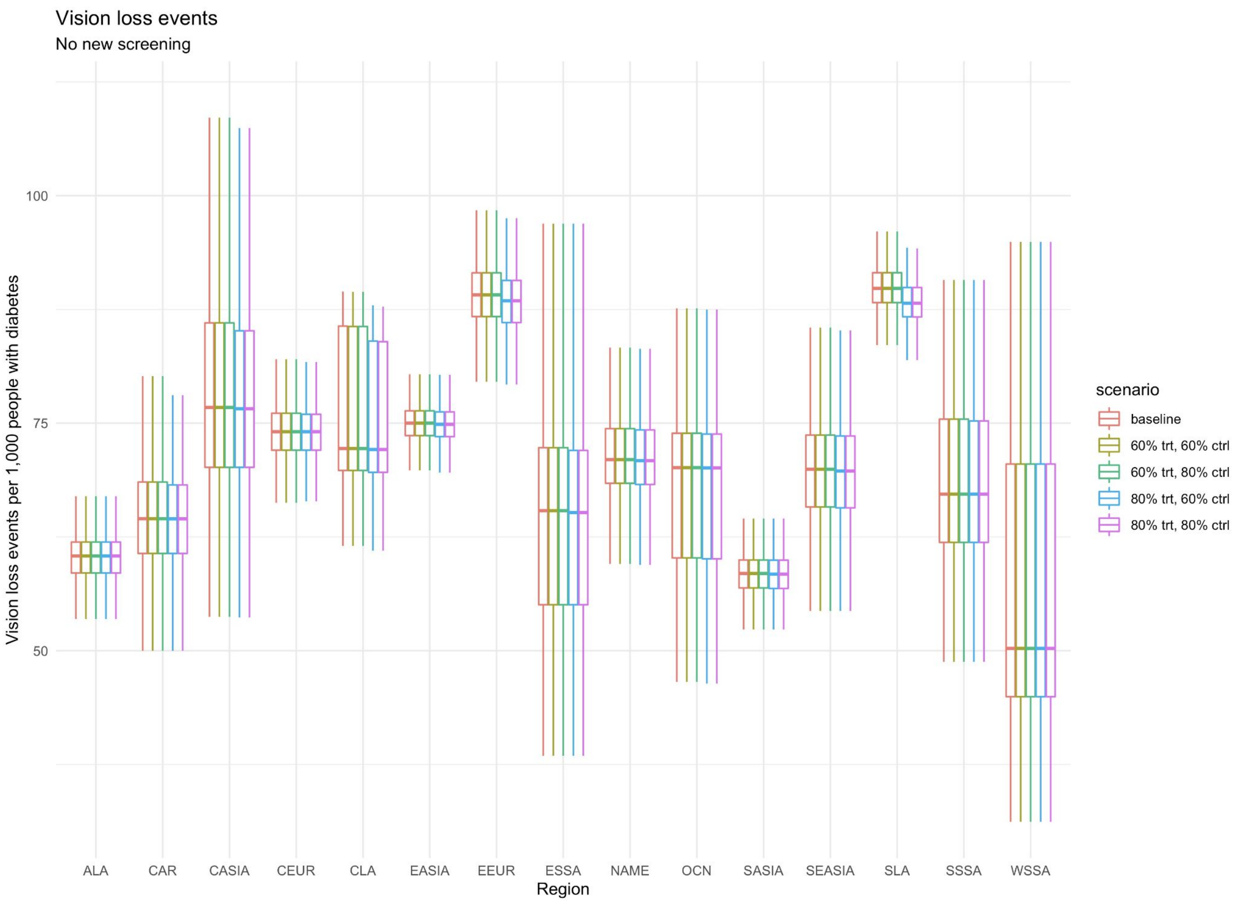

Appendix Figure 14: Estimated frequency of incident retinopathy events with screening for 60% diagnosis and with increased treatment and control. We brought the proportion of people with diabetes who were diagnosed up to 60% within each country's population, and the portion of those with diabetes and hypertension who were diagnosed with hypertension up to 60%, leaving unaltered those countries with a baseline level above these proportions The figure displays increases in treatment (trt) with blood pressure, glycaemic, or statin medicines to 60% or 80% of those diagnosed with hypertension or diabetes or indicated for statins (age 40 or older or >20% estimated 10-year risk of cardiovascular events), respectively; and control (ctrl) of blood pressure (systolic pressure <130 mmHg and diastolic pressure <80 mmHg) or glycaemia (haemoglobin A1c ≤7% or fasting plasma glucose <7 mmol/L [126 mg/dL]) to 60% or 80% of those treated for hypertension or diabetes, respectively. The retinopathy end-point was severe vision loss, defined as <20/200 visual acuity by Snellen chart. Region abbreviations: CAR = Caribbean; CASIA = Central Asia; CLA = Central Latin America; EEUR = Eastern Europe; ESSA = Eastern Sub-Saharan Africa; NAME = North Africa/Middle East; OCN = Oceania; SASIA = South Asia; SEASIA = Southeast Asia; SLA = South Latin America; SSSA = South Sub-Saharan Africa; WSSA = Western Sub-Saharan Africa.

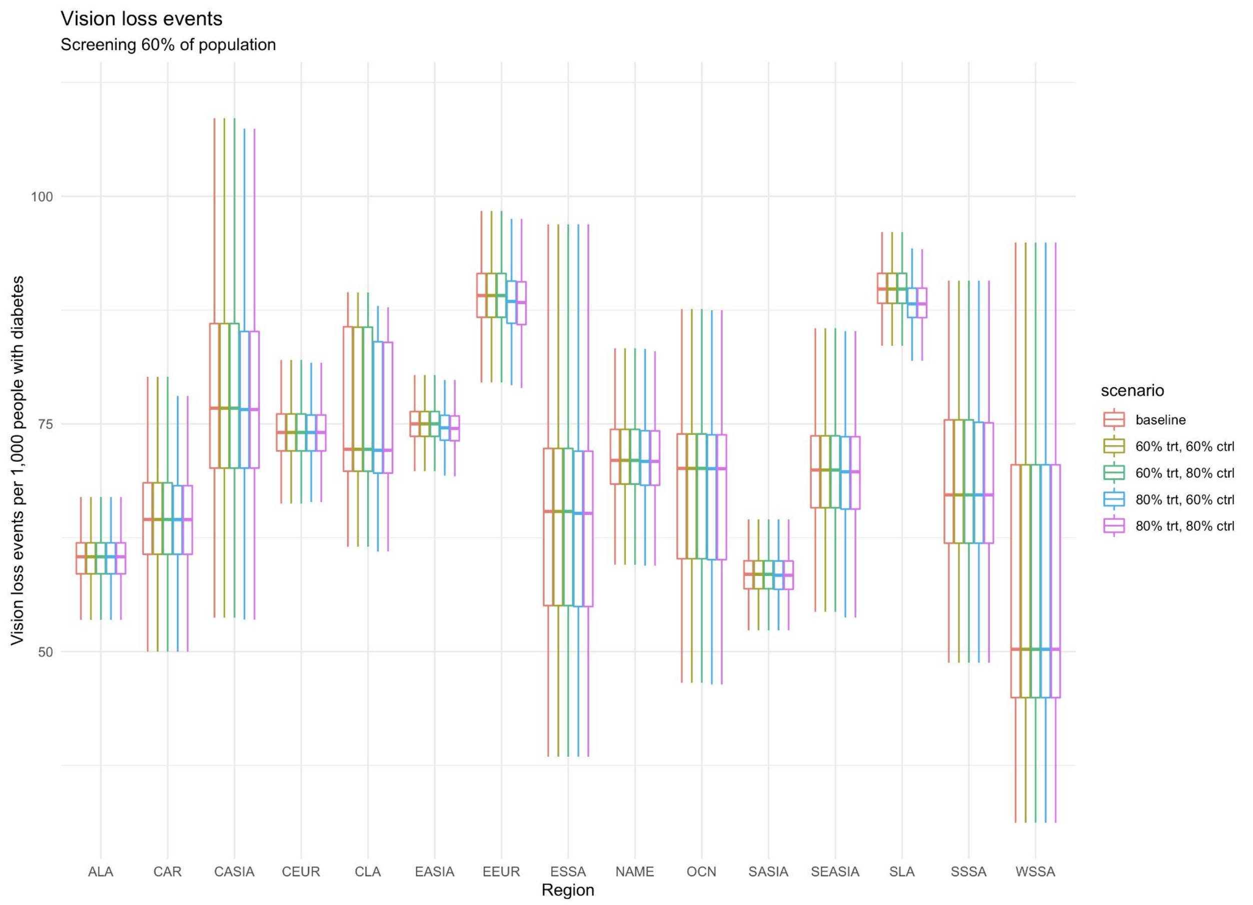
Vision loss events
Screening 60% of population
Vision loss events per 1,000 people with diabetes
100
75
50
ALA
CAR
CASIA
CEUR
CLA
EASIA
EEUR
ESSA
NAME
OCN
SASIA
SEASIA
SLA
SSSA
WSSA
Region
scenario
baseline
60% trt, 60% ctrl
60% trt, 80% ctrl
80% trt, 60% ctrl
80% trt, 80% ctrl

Appendix Figure 15: Estimated frequency of incident retinopathy events with screening for 80% diagnosis and with increased treatment and control. We brought the proportion of people with diabetes who were diagnosed up to 80% within each country's population, and the portion of those with diabetes and hypertension who were diagnosed with hypertension up to 80%, leaving unaltered those countries with a baseline level above these proportions The figure displays increases in treatment (trt) with blood pressure, glycaemic, or statin medicines to 60% or 80% of those diagnosed with hypertension or diabetes or indicated for statins (age 40 or older or >20% estimated 10-year risk of cardiovascular events), respectively; and control (ctrl) of blood pressure (systolic pressure <130 mmHg and diastolic pressure <80 mmHg) or glycaemia (haemoglobin A1c ≤7% or fasting plasma glucose <7 mmol/L [126 mg/dL]) to 60% or 80% of those treated for hypertension or diabetes, respectively. The retinopathy end-point was severe vision loss, defined as <20/200 visual acuity by Snellen chart. Region abbreviations: CAR = Caribbean; CASIA = Central Asia; CLA = Central Latin America; EEUR = Eastern Europe; ESSA = Eastern Sub-Saharan Africa; NAME = North Africa/Middle East; OCN = Oceania; SASIA = South Asia; SEASIA = Southeast Asia; SLA = South Latin America; SSSA = South Sub-Saharan Africa; WSSA = Western Sub-Saharan Africa.

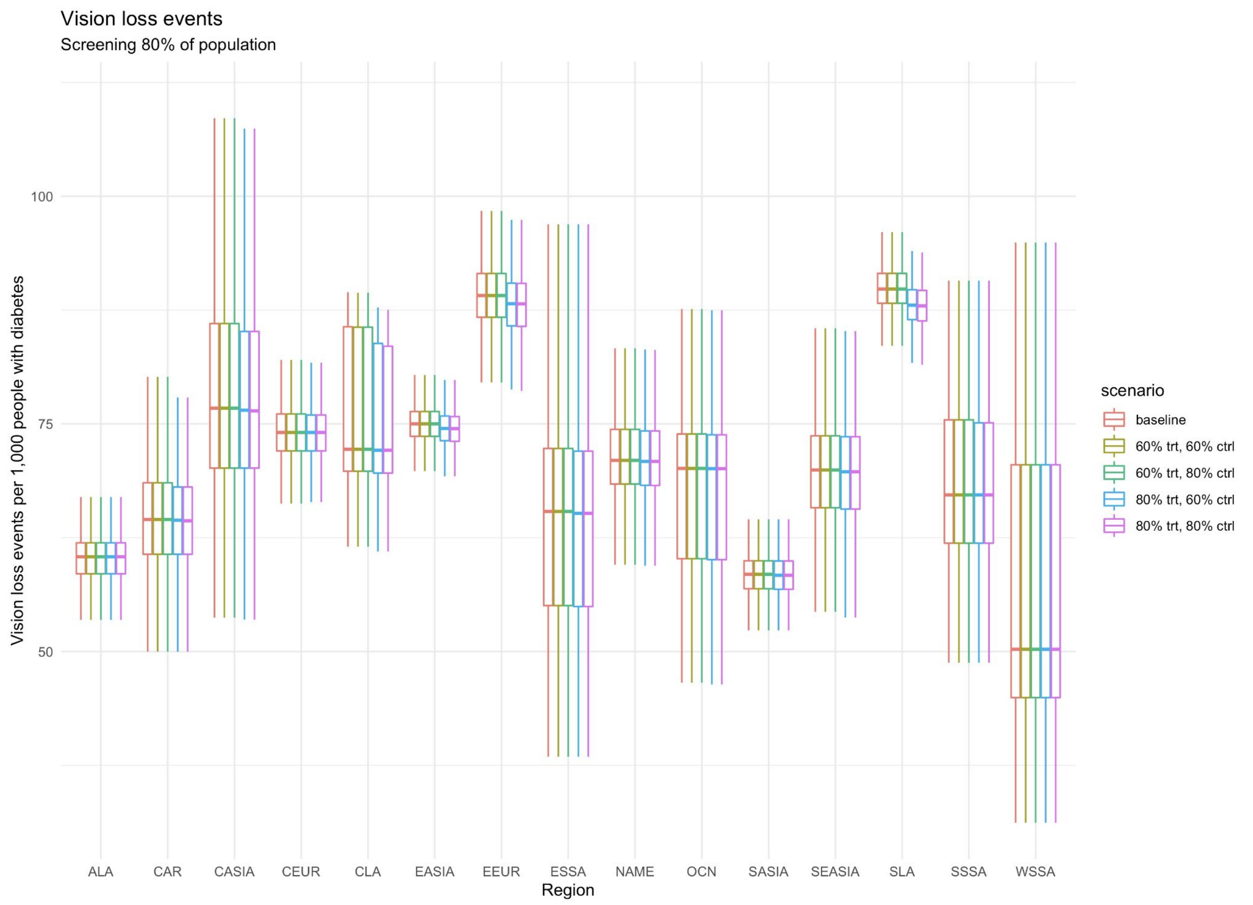
Vision loss events
Screening 80% of population
Vision loss events per 1,000 people with diabetes
100
75
50
ALA
CAR
CASIA
CEUR
CLA
EASIA
EEUR
ESSA
NAME
OCN
SASIA
SEASIA
SLA
SSSA
WSSA
Region
scenario
baseline
60% trt, 60% ctrl
60% trt, 80% ctrl
80% trt, 60% ctrl
80% trt, 80% ctrl

Appendix Figure 16: Estimated DALYs lost to cardiovascular disease (CVD) without new screening but with increased treatment and control. The figure displays increases in treatment (trt) with blood pressure, glycaemic, or statin medicines to 60% or 80% of those diagnosed with hypertension or diabetes or indicated for statins (age 40 or older or >20% estimated 10-year risk of cardiovascular events), respectively; and control (ctrl) of blood pressure (systolic pressure <130 mmHg and diastolic pressure <80 mmHg) or glycaemia (haemoglobin A1c ≤7% or fasting plasma glucose <7 mmol/L [126 mg/dL]) to 60% or 80% of those treated for hypertension or diabetes, respectively. Cardiovascular disease was defined as fatal and non-fatal myocardial infarction and stroke. Region abbreviations: CAR = Caribbean; CASIA = Central Asia; CLA = Central Latin America; EEUR = Eastern Europe; ESSA = Eastern Sub-Saharan Africa; NAME = North Africa/Middle East; OCN = Oceania; SASIA = South Asia; SEASIA = Southeast Asia; SLA = South Latin America; SSSA = South Sub-Saharan Africa; WSSA = Western Sub-Saharan Africa.

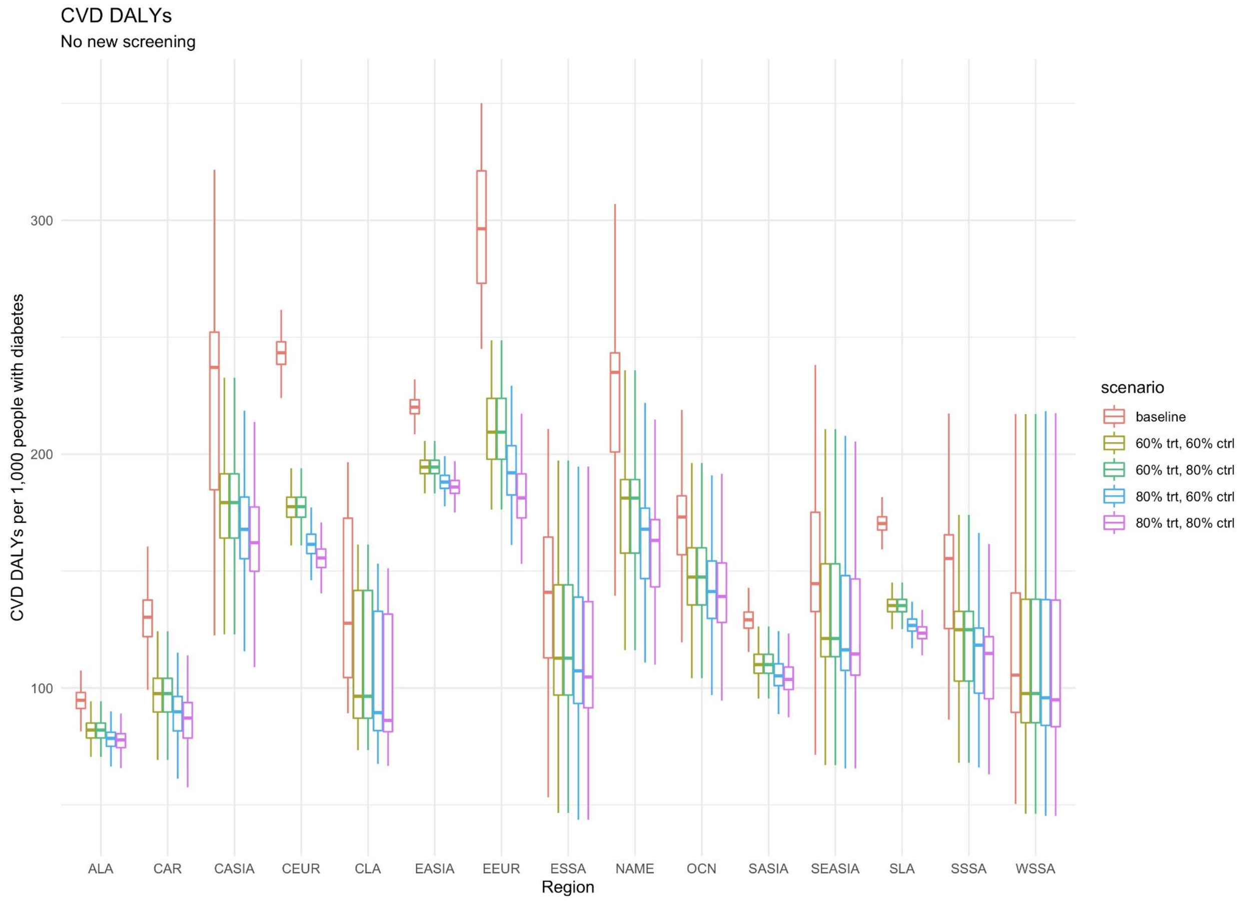

Appendix Figure 17: Estimated DALYs lost to cardiovascular disease (CVD) with screening for 60% diagnosis and with increased treatment and control. We brought the proportion of people with diabetes who were diagnosed up to 60% within each country's population, and the portion of those with diabetes and hypertension who were diagnosed with hypertension up to 60%, leaving unaltered those countries with a baseline level above these proportions The figure displays increases in treatment (trt) with blood pressure, glycaemic, or statin medicines to 60% or 80% of those diagnosed with hypertension or diabetes or indicated for statins (age 40 or older or >20% estimated 10-year risk of cardiovascular events), respectively; and control (ctrl) of blood pressure (systolic pressure <130 mmHg and diastolic pressure <80 mmHg) or glycaemia (haemoglobin A1c ≤7% or fasting plasma glucose <7 mmol/L [126 mg/dL]) to 60% or 80% of those treated for hypertension or diabetes, respectively. Cardiovascular disease was defined as fatal and non-fatal myocardial infarction and stroke. Region abbreviations: CAR = Caribbean; CASIA = Central Asia; CLA = Central Latin America; EEUR = Eastern Europe; ESSA = Eastern Sub-Saharan Africa; NAME = North Africa/Middle East; OCN = Oceania; SASIA = South Asia; SEASIA = Southeast Asia; SLA = South Latin America; SSSA = South Sub-Saharan Africa; WSSA = Western Sub-Saharan Africa.

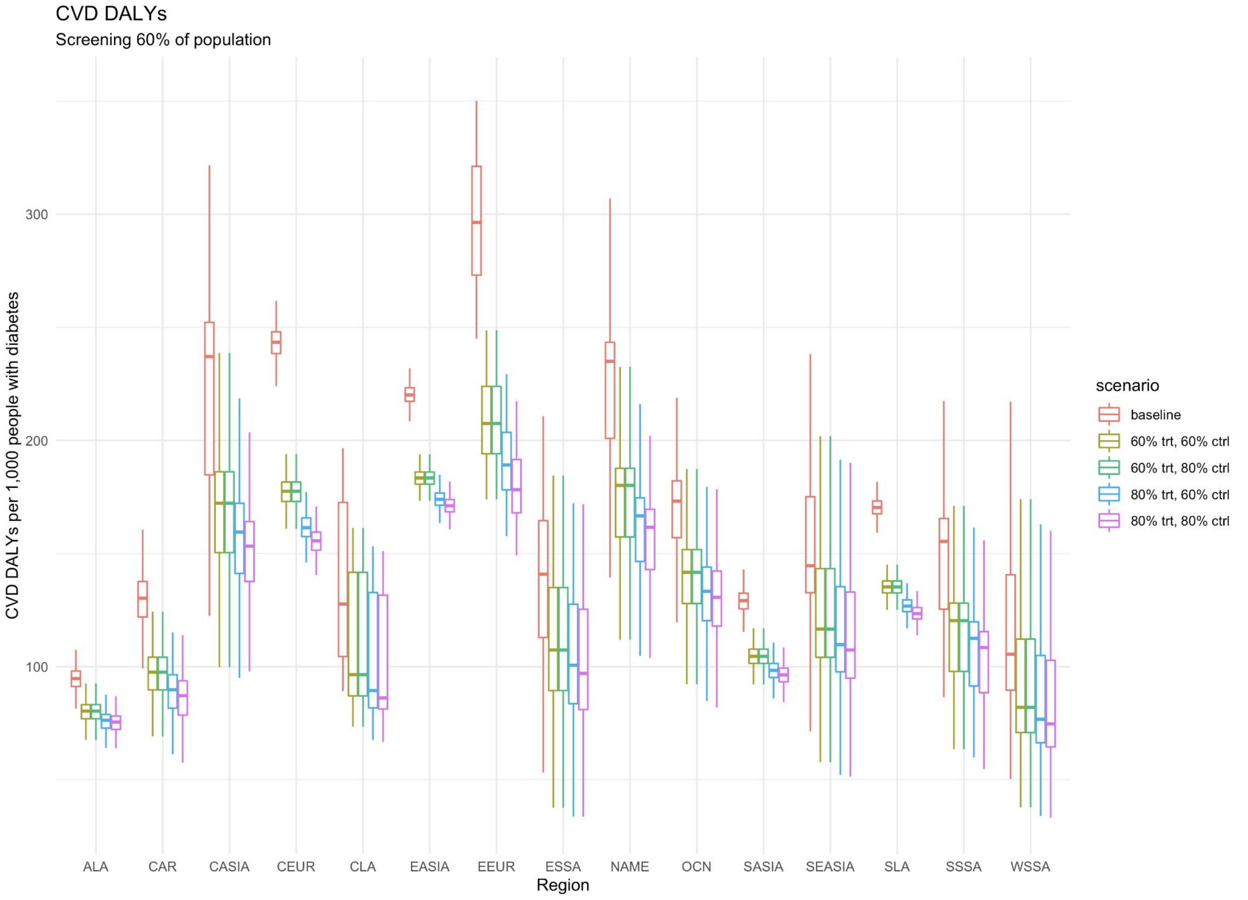
CVD DALYs
Screening 60% of population
CVD DALYs per 1,000 people with diabetes
300
200
100
ALA
CAR
CASIA
CEUR
CLA
EASIA
EEUR
ESSA
NAME
OCN
SASIA
SEASIA
SLA
SSSA
WSSA
Region
scenario
baseline
60% trt, 60% ctrl
60% trt, 80% ctrl
80% trt, 60% ctrl
80% trt, 80% ctrl

Appendix Figure 18: Estimated DALYs lost to cardiovascular disease (CVD) with screening for 80% diagnosis and with increased treatment and control. We brought the proportion of people with diabetes who were diagnosed up to 80% within each country's population, and the portion of those with diabetes and hypertension who were diagnosed with hypertension up to 80%, leaving unaltered those countries with a baseline level above these proportions The figure displays increases in treatment (trt) with blood pressure, glycaemic, or statin medicines to 60% or 80% of those diagnosed with hypertension or diabetes or indicated for statins (age 40 or older or >20% estimated 10-year risk of cardiovascular events), respectively; and control (ctrl) of blood pressure (systolic pressure <130 mmHg and diastolic pressure <80 mmHg) or glycaemia (haemoglobin A1c ≤7% or fasting plasma glucose <7 mmol/L [126 mg/dL]) to 60% or 80% of those treated for hypertension or diabetes, respectively. Cardiovascular disease was defined as fatal and non-fatal myocardial infarction and stroke. Region abbreviations: CAR = Caribbean; CASIA = Central Asia; CLA = Central Latin America; EEUR = Eastern Europe; ESSA = Eastern Sub-Saharan Africa; NAME = North Africa/Middle East; OCN = Oceania; SASIA = South Asia; SEASIA = Southeast Asia; SLA = South Latin America; SSSA = South Sub-Saharan Africa; WSSA = Western Sub-Saharan Africa.

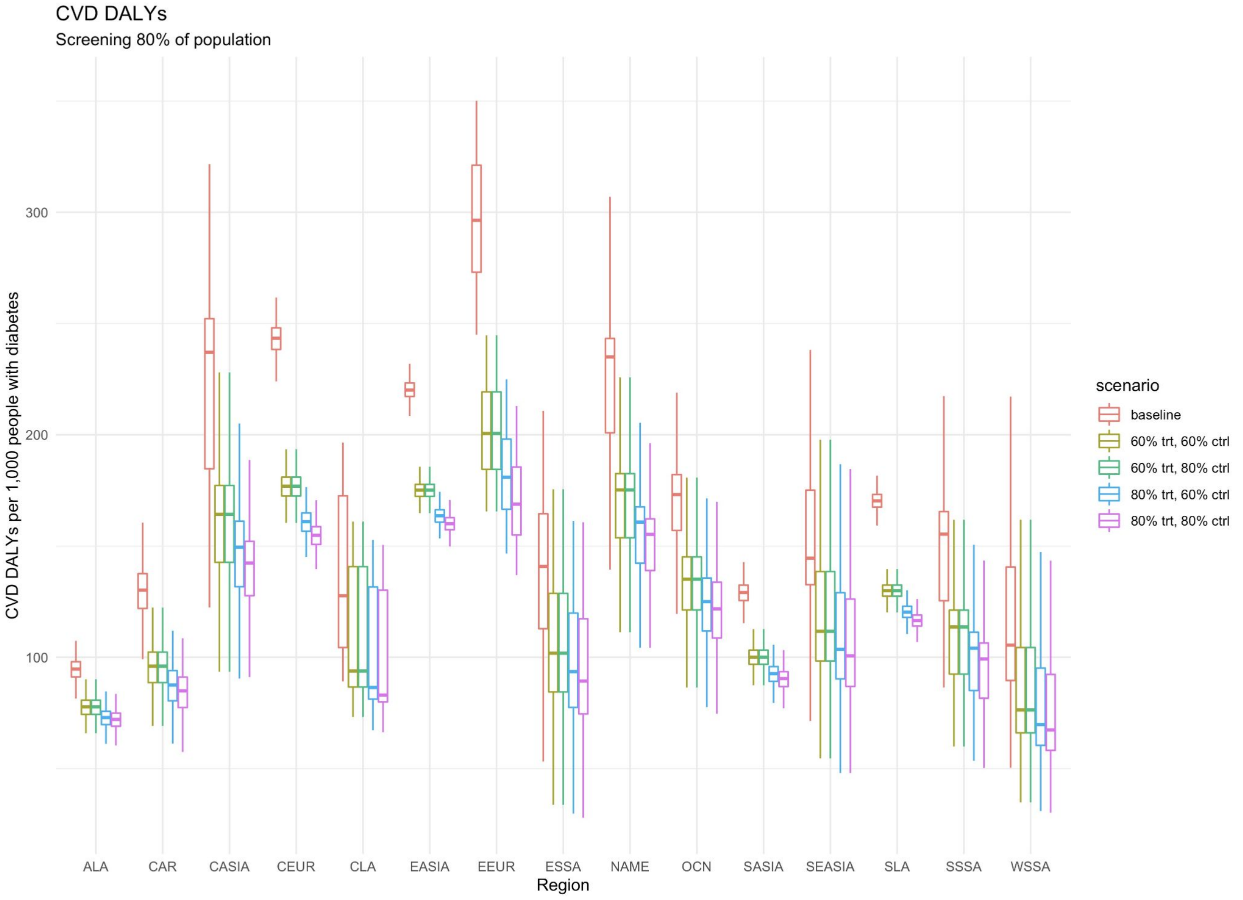
CVD DALYs
Screening 80% of population
CVD DALYs per 1,000 people with diabetes
300
200
100
ALA
CAR
CASIA
CEUR
CLA
EASIA
EEUR
ESSA
NAME
OCN
SASIA
SEASIA
SLA
SSSA
WSSA
Region
scenario
baseline
60% trt, 60% ctrl
60% trt, 80% ctrl
80% trt, 60% ctrl
80% trt, 80% ctrl

Appendix Figure 19: Estimated DALYs lost to congestive heart failure (CHF) without new screening but with increased treatment and control. The figure displays increases in treatment (trt) with blood pressure, glycaemic, or statin medicines to 60% or 80% of those diagnosed with hypertension or diabetes or indicated for statins (age 40 or older or >20% estimated 10-year risk of cardiovascular events), respectively; and control (ctrl) of blood pressure (systolic pressure <130 mmHg and diastolic pressure <80 mmHg) or glycaemia (haemoglobin A1c ≤7% or fasting plasma glucose <7 mmol/L [126 mg/dL]) to 60% or 80% of those treated for hypertension or diabetes, respectively. Congestive heart failure was defined as an ejection fraction of <40%, with New York Heart Association class III or IV functional limitations. Region abbreviations: CAR = Caribbean; CASIA = Central Asia; CLA = Central Latin America; EEUR = Eastern Europe; ESSA = Eastern Sub-Saharan Africa; NAME = North Africa/Middle East; OCN = Oceania; SASIA = South Asia; SEASIA = Southeast Asia; SLA = South Latin America; SSSA = South Sub-Saharan Africa; WSSA = Western Sub-Saharan Africa.

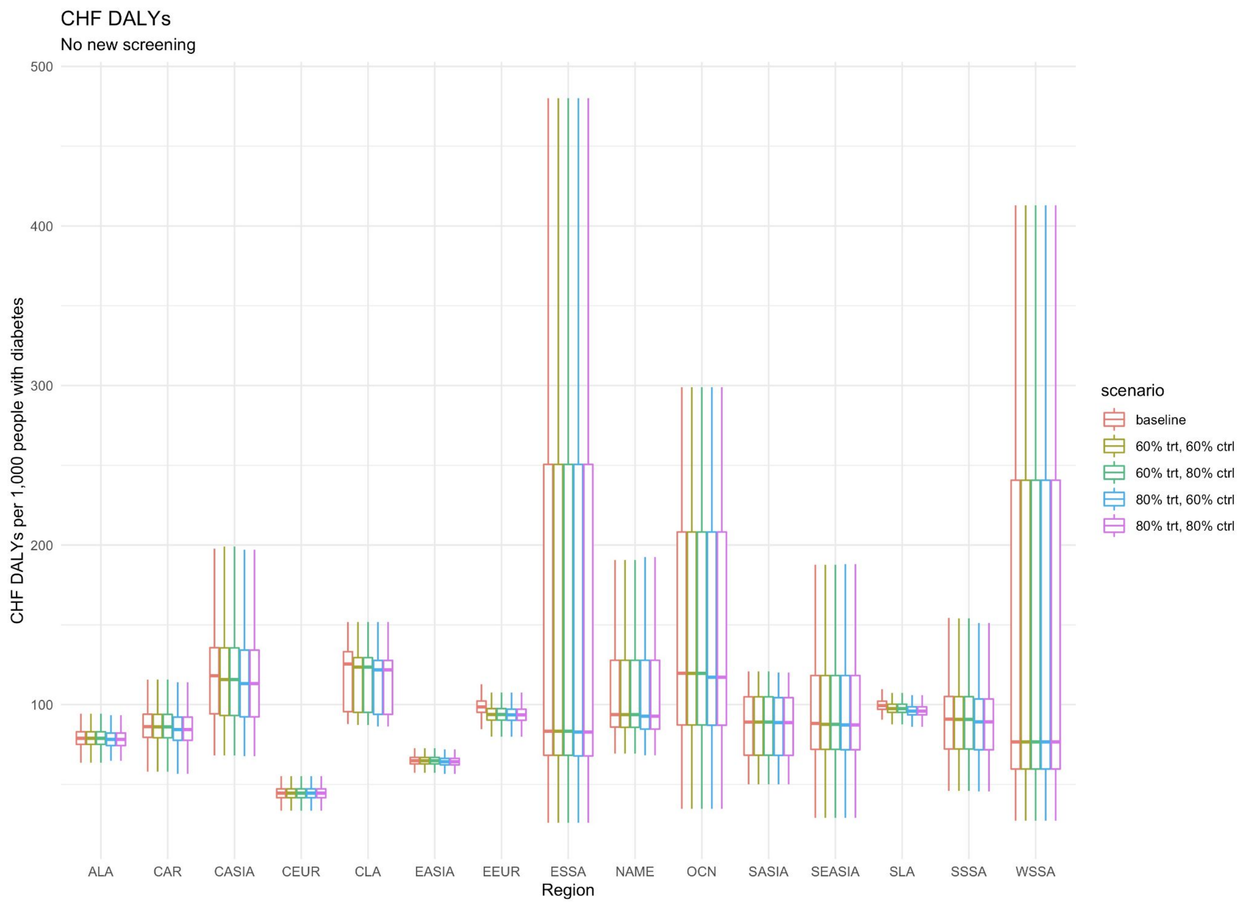
CHF DALYs
No new screening
500
400
300
200
100
CHF DALYs per 1,000 people with diabetes
ALA
CAR
CASIA
CEUR
CLA
EASIA
EEUR
ESSA
NAME
OCN
SASIA
SEASIA
SLA
SSSA
WSSA
Region
scenario
baseline
60% trt, 60% ctrl
60% trt, 80% ctrl
80% trt, 60% ctrl
80% trt, 80% ctrl

Appendix Figure 20: Estimated DALYs lost to congestive heart failure (CHF) with screening for 60% diagnosis and with increased treatment and control. We brought the proportion of people with diabetes who were diagnosed up to 60% within each country's population, and the portion of those with diabetes and hypertension who were diagnosed with hypertension up to 60%, leaving unaltered those countries with a baseline level above these proportions The figure displays increases in treatment (trt) with blood pressure, glycaemic, or statin medicines to 60% or 80% of those diagnosed with hypertension or diabetes or indicated for statins (age 40 or older or >20% estimated 10-year risk of cardiovascular events), respectively; and control (ctrl) of blood pressure (systolic pressure <130 mmHg and diastolic pressure <80 mmHg) or glycaemia (haemoglobin A1c ≤7% or fasting plasma glucose <7 mmol/L [126 mg/dL]) to 60% or 80% of those treated for hypertension or diabetes, respectively. Congestive heart failure was defined as an ejection fraction of <40%, with New York Heart Association class III or IV functional limitations. Region abbreviations: CAR = Caribbean; CASIA = Central Asia; CLA = Central Latin America; EEUR = Eastern Europe; ESSA = Eastern Sub-Saharan Africa; NAME = North Africa/Middle East; OCN = Oceania; SASIA = South Asia; SEASIA = Southeast Asia; SLA = South Latin America; SSSA = South Sub-Saharan Africa; WSSA = Western Sub-Saharan Africa.

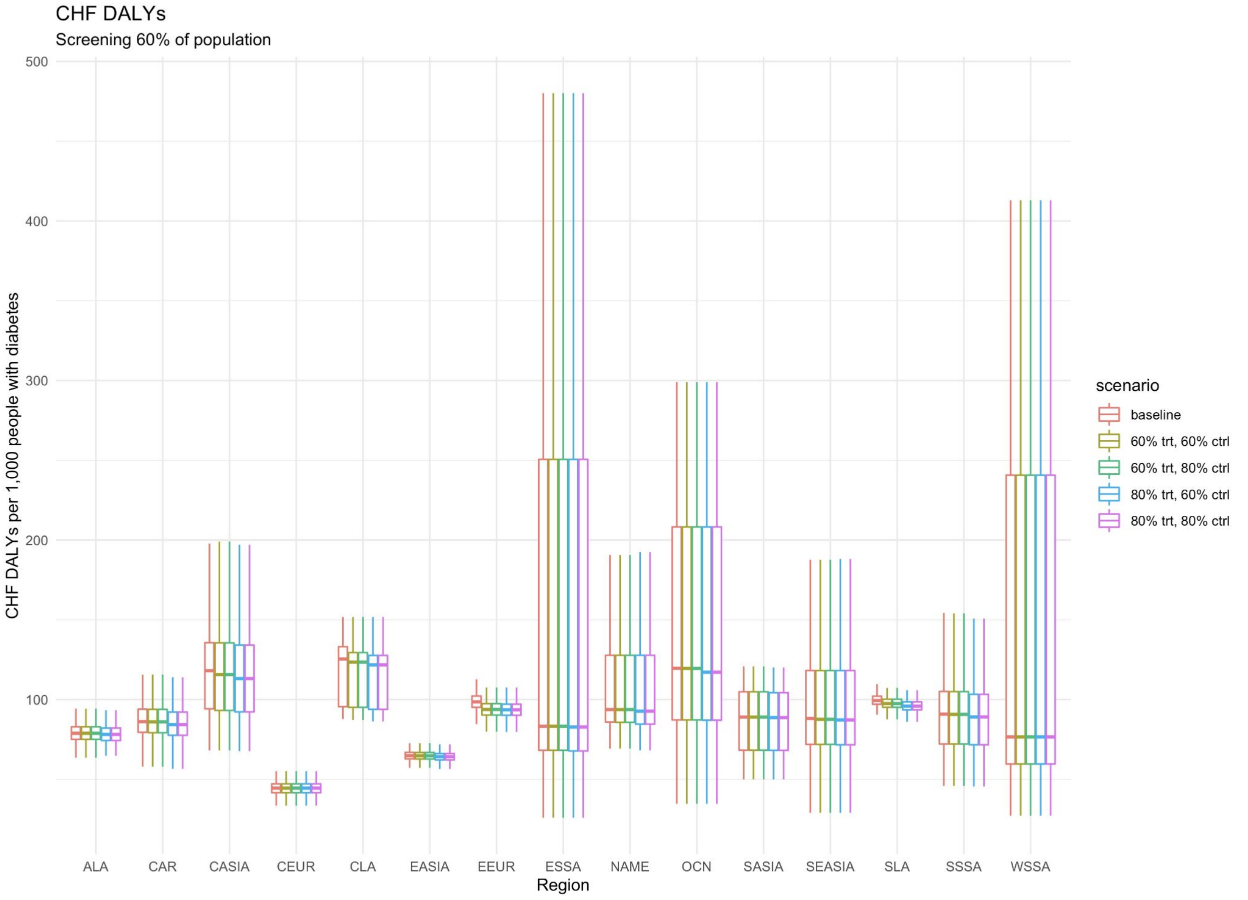

CHF DALYs
Screening 60% of population
CHF DALYs per 1,000 people with diabetes
500
400
300
200
100
ALA
CAR
CASIA
CEUR
CLA
EASIA
EEUR
ESSA
NAME
OCN
SASIA
SEASIA
SLA
SSSA
WSSA
Region
scenario
baseline
60% trt, 60% ctrl
60% trt, 80% ctrl
80% trt, 60% ctrl
80% trt, 80% ctrl

Appendix Figure 21: Estimated DALYs lost to congestive heart failure (CHF) with screening for 80% diagnosis and with increased treatment and control. We brought the proportion of people with diabetes who were diagnosed up to 80% within each country's population, and the portion of those with diabetes and hypertension who were diagnosed with hypertension up to 80%, leaving unaltered those countries with a baseline level above these proportions The figure displays increases in treatment (trt) with blood pressure, glycaemic, or statin medicines to 60% or 80% of those diagnosed with hypertension or diabetes or indicated for statins (age 40 or older or >20% estimated 10-year risk of cardiovascular events), respectively; and control (ctrl) of blood pressure (systolic pressure <130 mmHg and diastolic pressure <80 mmHg) or glycaemia (haemoglobin A1c ≤7% or fasting plasma glucose <7 mmol/L [126 mg/dL]) to 60% or 80% of those treated for hypertension or diabetes, respectively. Congestive heart failure was defined as an ejection fraction of <40%, with New York Heart Association class III or IV functional limitations. Region abbreviations: CAR = Caribbean; CASIA = Central Asia; CLA = Central Latin America; EEUR = Eastern Europe; ESSA = Eastern Sub-Saharan Africa; NAME = North Africa/Middle East; OCN = Oceania; SASIA = South Asia; SEASIA = Southeast Asia; SLA = South Latin America; SSSA = South Sub-Saharan Africa; WSSA = Western Sub-Saharan Africa.

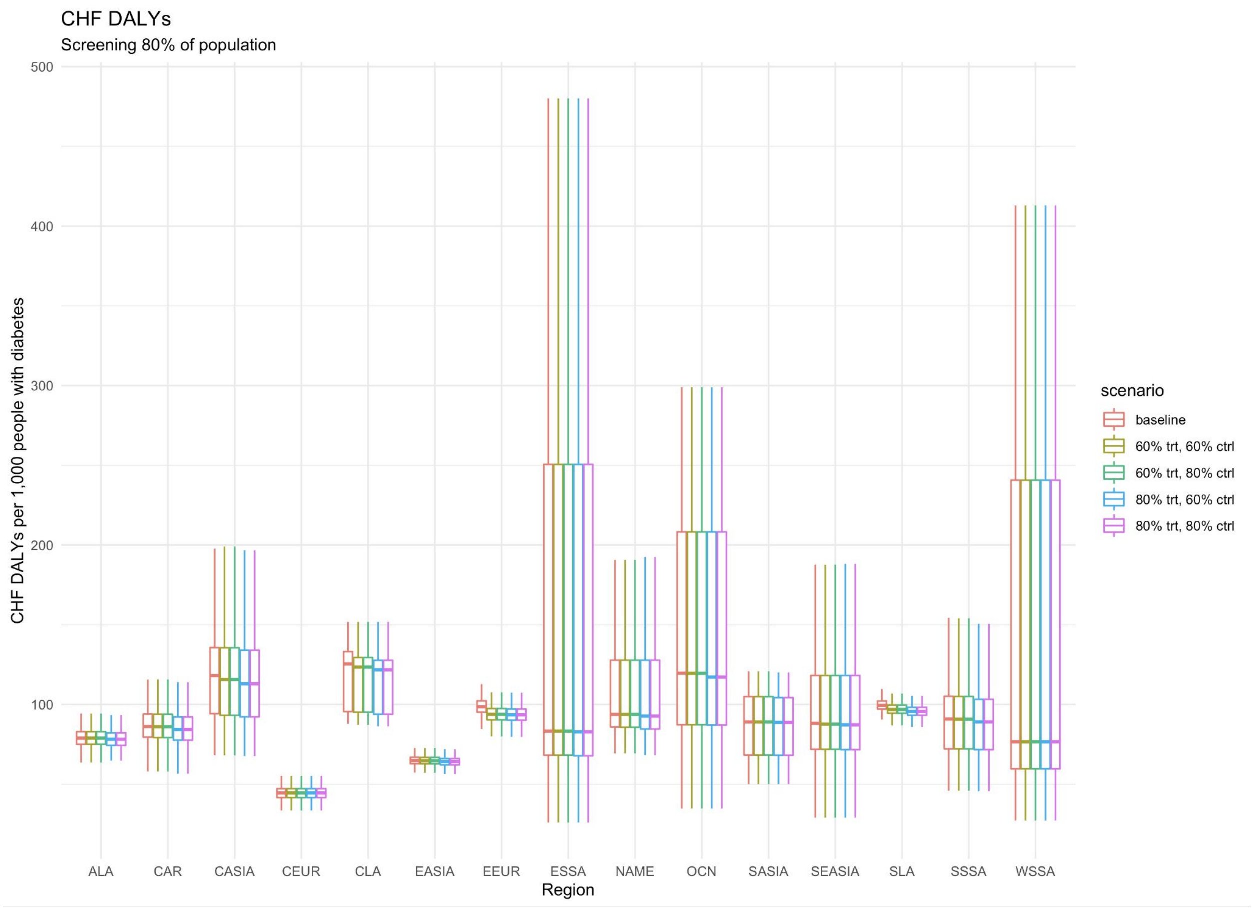
CHF DALYs
Screening 80% of population
CHF DALYs per 1,000 people with diabetes
500
400
300
200
100
ALA
CAR
CASIA
CEUR
CLA
EASIA
EEUR
ESSA
NAME
OCN
SASIA
SEASIA
SLA
SSSA
WSSA
Region
scenario
baseline
60% trt, 60% ctrl
60% trt, 80% ctrl
80% trt, 60% ctrl
80% trt, 80% ctrl

Appendix Figure 22: Estimated DALYs lost to end-stage renal disease (ESRD) without new screening but with increased treatment and control. The figure displays increases in treatment (trt) with blood pressure, glycaemic, or statin medicines to 60% or 80% of those diagnosed with hypertension or diabetes or indicated for statins (age 40 or older or >20% estimated 10-year risk of cardiovascular events), respectively; and control (ctrl) of blood pressure (systolic pressure <130 mmHg and diastolic pressure <80 mmHg) or glycaemia (haemoglobin A1c ≤7% or fasting plasma glucose <7 mmol/L [126 mg/dL]) to 60% or 80% of those treated for hypertension or diabetes, respectively. End-stage renal disease (ESRD) was defined as estimated glomerular filtration rate <15 mL/min/1.73m^2 or needing dialysis/transplant. Region abbreviations: CAR = Caribbean; CASIA = Central Asia; CLA = Central Latin America; EEUR = Eastern Europe; ESSA = Eastern Sub-Saharan Africa; NAME = North Africa/Middle East; OCN = Oceania; SASIA = South Asia; SEASIA = Southeast Asia; SLA = South Latin America; SSSA = South Sub-Saharan Africa; WSSA = Western Sub-Saharan Africa.

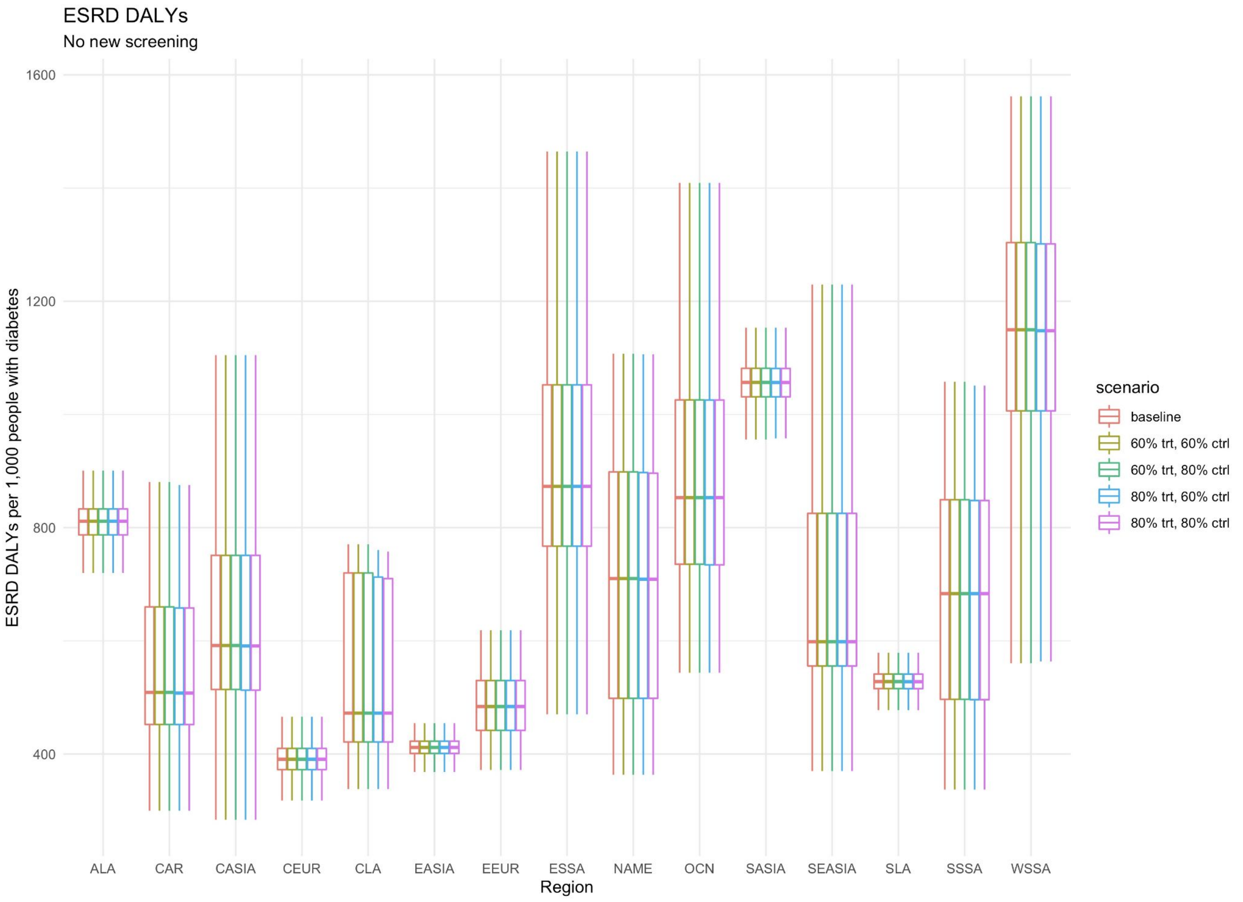

Appendix Figure 23: Estimated DALYs lost to incident end-stage renal disease (ESRD) events with screening for 60% diagnosis and with increased treatment and control. We brought the proportion of people with diabetes who were diagnosed up to 60% within each country's population, and the portion of those with diabetes and hypertension who were diagnosed with hypertension up to 60%, leaving unaltered those countries with a baseline level above these proportions The figure displays increases in treatment (trt) with blood pressure, glycaemic, or statin medicines to 60% or 80% of those diagnosed with hypertension or diabetes or indicated for statins (age 40 or older or >20% estimated 10-year risk of cardiovascular events), respectively; and control (ctrl) of blood pressure (systolic pressure <130 mmHg and diastolic pressure <80 mmHg) or glycaemia (haemoglobin A1c ≤7% or fasting plasma glucose <7 mmol/L [126 mg/dL]) to 60% or 80% of those treated for hypertension or diabetes, respectively. End-stage renal disease (ESRD) was defined as estimated glomerular filtration rate <15 mL/min/1.73m^2 or needing dialysis/transplant. Region abbreviations: CAR = Caribbean; CASIA = Central Asia; CLA = Central Latin America; EEUR = Eastern Europe; ESSA = Eastern Sub-Saharan Africa; NAME = North Africa/Middle East; OCN = Oceania; SASIA = South Asia; SEASIA = Southeast Asia; SLA = South Latin America; SSSA = South Sub-Saharan Africa; WSSA = Western Sub-Saharan Africa.

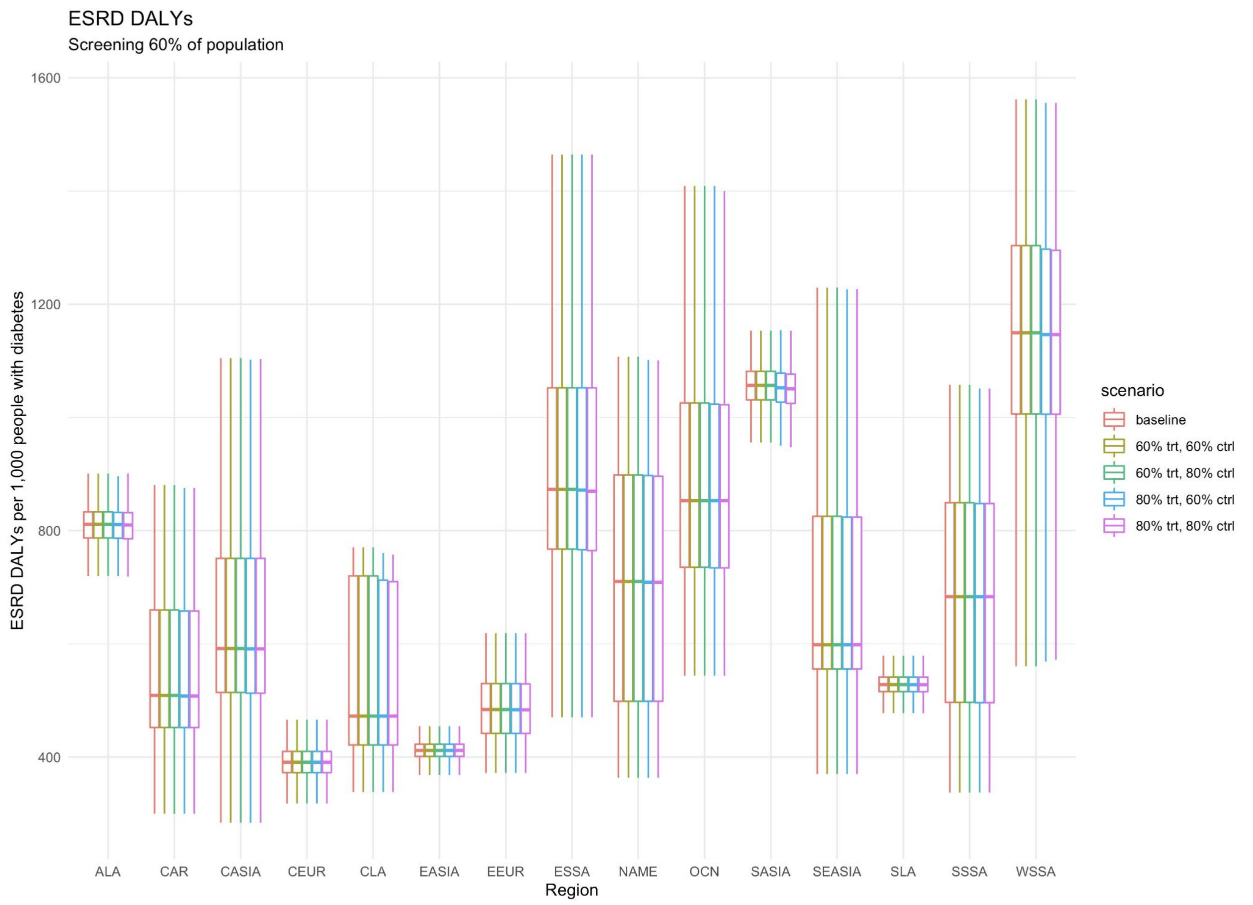
ESRD DALYs
Screening 60% of population
ESRD DALYs per 1,000 people with diabetes
1600
1200
800
400
ALA
CAR
CASIA
CEUR
CLA
EASIA
EEUR
ESSA
NAME
OCN
SASIA
SEASIA
SLA
SSSA
WSSA
Region
scenario
baseline
60% trt, 60% ctrl
60% trt, 80% ctrl
80% trt, 60% ctrl
80% trt, 80% ctrl

Appendix Figure 24: Estimated DALYs lost to end-stage renal disease (ESRD) with screening for 80% diagnosis and with increased treatment and control. We brought the proportion of people with diabetes who were diagnosed up to 80% within each country's population, and the portion of those with diabetes and hypertension who were diagnosed with hypertension up to 80%, leaving unaltered those countries with a baseline level above these proportions The figure displays increases in treatment (trt) with blood pressure, glycaemic, or statin medicines to 60% or 80% of those diagnosed with hypertension or diabetes or indicated for statins (age 40 or older or >20% estimated 10-year risk of cardiovascular events), respectively; and control (ctrl) of blood pressure (systolic pressure <130 mmHg and diastolic pressure <80 mmHg) or glycaemia (haemoglobin A1c ≤7% or fasting plasma glucose <7 mmol/L [126 mg/dL]) to 60% or 80% of those treated for hypertension or diabetes, respectively. End-stage renal disease (ESRD) was defined as estimated glomerular filtration rate <15 mL/min/1.73m^2 or needing dialysis/transplant. Region abbreviations: CAR = Caribbean; CASIA = Central Asia; CLA = Central Latin America; EEUR = Eastern Europe; ESSA = Eastern Sub-Saharan Africa; NAME = North Africa/Middle East; OCN = Oceania; SASIA = South Asia; SEASIA = Southeast Asia; SLA = South Latin America; SSSA = South Sub-Saharan Africa; WSSA = Western Sub-Saharan Africa.

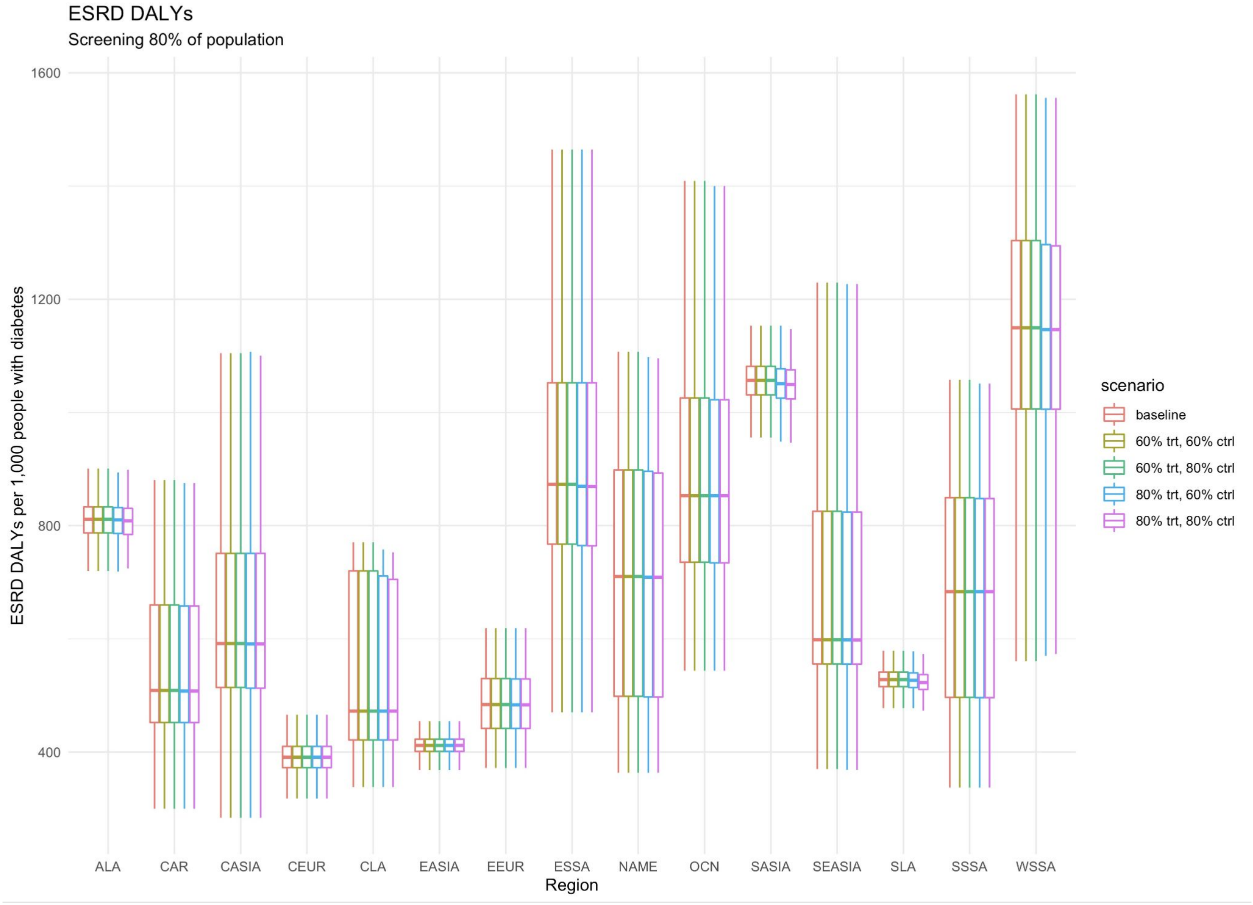
ESRD DALYs
Screening 80% of population
ESRD DALYs per 1,000 people with diabetes
1600
1200
800
400
ALA
CAR
CASIA
CEUR
CLA
EASIA
EEUR
ESSA
NAME
OCN
SASIA
SEASIA
SLA
SSSA
WSSA
Region
scenario
baseline
60% trt, 60% ctrl
60% trt, 80% ctrl
80% trt, 60% ctrl
80% trt, 80% ctrl

Appendix Figure 25: Estimated DALYs lost to neuropathy without new screening but with increased treatment and control. The figure displays increases in treatment (trt) with blood pressure, glycaemic, or statin medicines to 60% or 80% of those diagnosed with hypertension or diabetes or indicated for statins (age 40 or older or >20% estimated 10-year risk of cardiovascular events), respectively; and control (ctrl) of blood pressure (systolic pressure <130 mmHg and diastolic pressure <80 mmHg) or glycaemia (haemoglobin A1c ≤7% or fasting plasma glucose <7 mmol/L [126 mg/dL]) to 60% or 80% of those treated for hypertension or diabetes, respectively. Neuropathy was defined as pressure sensation loss by Semmes-Weinstein 5.07/10 gram monofilament exam. Region abbreviations: CAR = Caribbean; CASIA = Central Asia; CLA = Central Latin America; EEUR = Eastern Europe; ESSA = Eastern Sub-Saharan Africa; NAME = North Africa/Middle East; OCN = Oceania; SASIA = South Asia; SEASIA = Southeast Asia; SLA = South Latin America; SSSA = South Sub-Saharan Africa; WSSA = Western Sub-Saharan Africa.

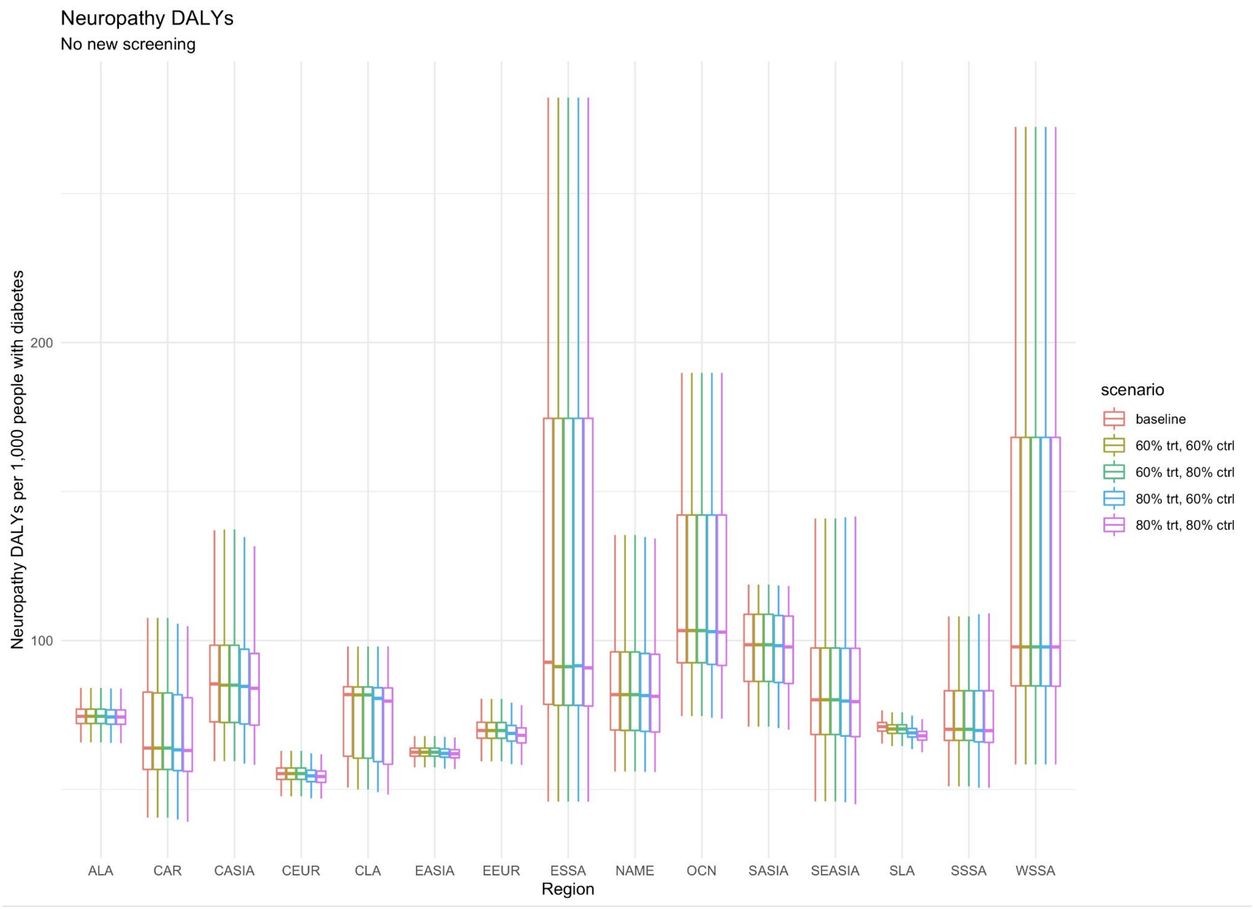

Appendix Figure 26: Estimated DALYs lost to neuropathy with screening for 60% diagnosis and with increased treatment and control. We brought the proportion of people with diabetes who were diagnosed up to 60% within each country's population, and the portion of those with diabetes and hypertension who were diagnosed with hypertension up to 60%, leaving unaltered those countries with a baseline level above these proportions The figure displays increases in treatment (trt) with blood pressure, glycaemic, or statin medicines to 60% or 80% of those diagnosed with hypertension or diabetes or indicated for statins (age 40 or older or >20% estimated 10-year risk of cardiovascular events), respectively; and control (ctrl) of blood pressure (systolic pressure <130 mmHg and diastolic pressure <80 mmHg) or glycaemia (haemoglobin A1c ≤7% or fasting plasma glucose <7 mmol/L [126 mg/dL]) to 60% or 80% of those treated for hypertension or diabetes, respectively. Neuropathy was defined as pressure sensation loss by Semmes-Weinstein 5.07/10 gram monofilament exam. Region abbreviations: CAR = Caribbean; CASIA = Central Asia; CLA = Central Latin America; EEUR = Eastern Europe; ESSA = Eastern Sub-Saharan Africa; NAME = North Africa/Middle East; OCN = Oceania; SASIA = South Asia; SEASIA = Southeast Asia; SLA = South Latin America; SSSA = South Sub-Saharan Africa; WSSA = Western Sub-Saharan Africa.

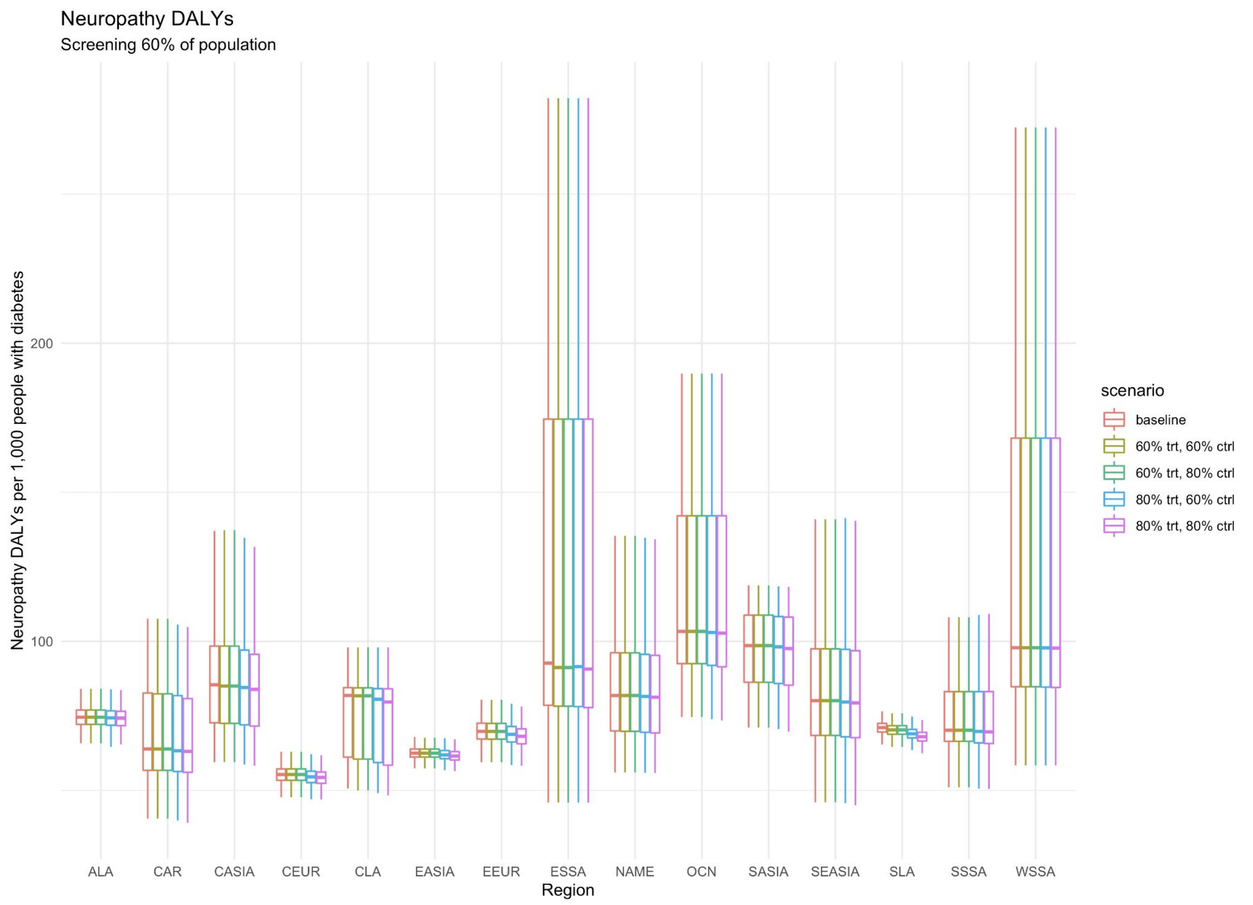
Neuropathy DALYs
Screening 60% of population
Neuropathy DALYs per 1,000 people with diabetes
200
100
ALA
CAR
CASIA
CEUR
CLA
EASIA
EEUR
ESSA
NAME
OCN
SASIA
SEASIA
SLA
SSSA
WSSA
Region
scenario
baseline
60% trt, 60% ctrl
60% trt, 80% ctrl
80% trt, 60% ctrl
80% trt, 80% ctrl

Appendix Figure 27: Estimated DALYs lost to neuropathy with screening for 80% diagnosis and with increased treatment and control. We brought the proportion of people with diabetes who were diagnosed up to 80% within each country's population, and the portion of those with diabetes and hypertension who were diagnosed with hypertension up to 80%, leaving unaltered those countries with a baseline level above these proportions The figure displays increases in treatment (trt) with blood pressure, glycaemic, or statin medicines to 60% or 80% of those diagnosed with hypertension or diabetes or indicated for statins (age 40 or older or >20% estimated 10-year risk of cardiovascular events), respectively; and control (ctrl) of blood pressure (systolic pressure <130 mmHg and diastolic pressure <80 mmHg) or glycaemia (haemoglobin A1c ≤7% or fasting plasma glucose <7 mmol/L [126 mg/dL]) to 60% or 80% of those treated for hypertension or diabetes, respectively. Neuropathy was defined as pressure sensation loss by Semmes-Weinstein 5.07/10 gram monofilament exam. Region abbreviations: CAR = Caribbean; CASIA = Central Asia; CLA = Central Latin America; EEUR = Eastern Europe; ESSA = Eastern Sub-Saharan Africa; NAME = North Africa/Middle East; OCN = Oceania; SASIA = South Asia; SEASIA = Southeast Asia; SLA = South Latin America; SSSA = South Sub-Saharan Africa; WSSA = Western Sub-Saharan Africa.

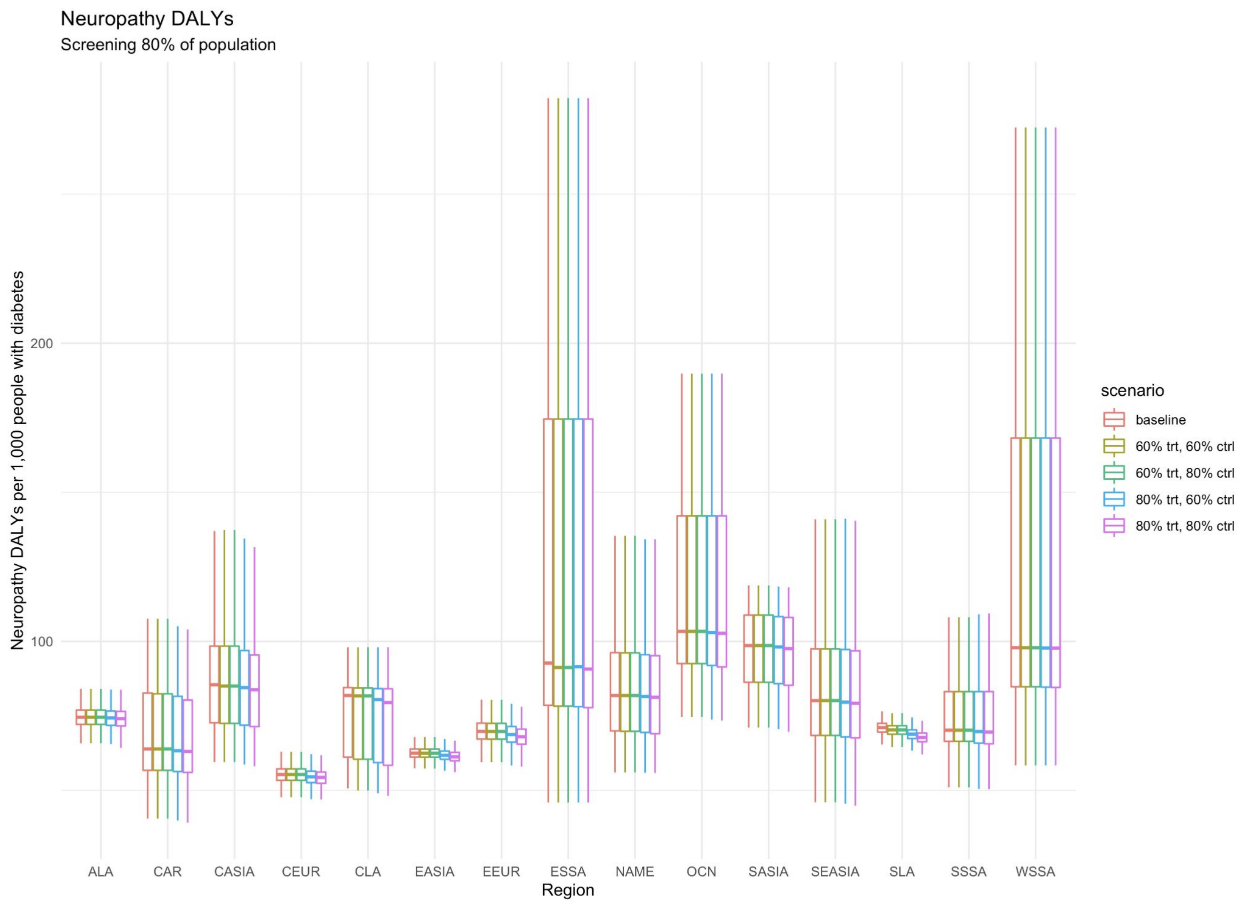
Neuropathy DALYs
Screening 80% of population
Neuropathy DALYs per 1,000 people with diabetes
200
100
ALA
CAR
CASIA
CEUR
CLA
EASIA
EEUR
ESSA
NAME
OCN
SASIA
SEASIA
SLA
SSSA
WSSA
Region
scenario
baseline
60% trt, 60% ctrl
60% trt, 80% ctrl
80% trt, 60% ctrl
80% trt, 80% ctrl

Appendix Figure 28: Estimated frequency DALYs lost to retinopathy without new screening but with increased treatment and control. The figure displays increases in treatment (trt) with blood pressure, glycaemic, or statin medicines to 60% or 80% of those diagnosed with hypertension or diabetes or indicated for statins (age 40 or older or >20% estimated 10-year risk of cardiovascular events), respectively; and control (ctrl) of blood pressure (systolic pressure <130 mmHg and diastolic pressure <80 mmHg) or glycaemia (haemoglobin A1c ≤7% or fasting plasma glucose <7 mmol/L [126 mg/dL]) to 60% or 80% of those treated for hypertension or diabetes, respectively. The retinopathy end-point was severe vision loss, defined as <20/200 visual acuity by Snellen chart. Region abbreviations: CAR = Caribbean; CASIA = Central Asia; CLA = Central Latin America; EEUR = Eastern Europe; ESSA = Eastern Sub-Saharan Africa; NAME = North Africa/Middle East; OCN = Oceania; SASIA = South Asia; SEASIA = Southeast Asia; SLA = South Latin America; SSSA = South Sub-Saharan Africa; WSSA = Western Sub-Saharan Africa.

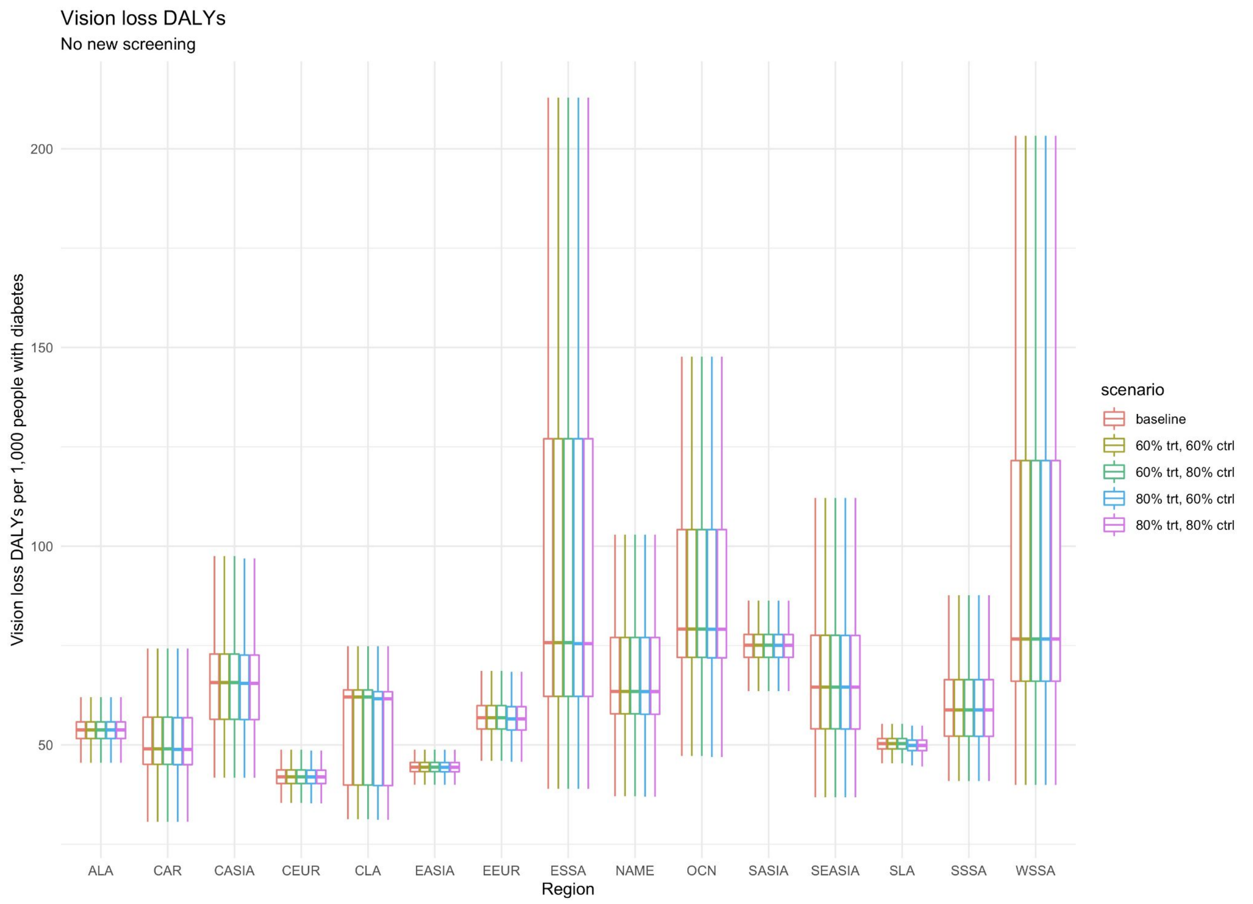

Appendix Figure 29: Estimated frequency of DALYs lost from retinopathy with screening for 60% diagnosis and with increased treatment and control. We brought the proportion of people with diabetes who were diagnosed up to 60% within each country's population, and the portion of those with diabetes and hypertension who were diagnosed with hypertension up to 60%, leaving unaltered those countries with a baseline level above these proportions The figure displays increases in treatment (trt) with blood pressure, glycaemic, or statin medicines to 60% or 80% of those diagnosed with hypertension or diabetes or indicated for statins (age 40 or older or >20% estimated 10-year risk of cardiovascular events), respectively; and control (ctrl) of blood pressure (systolic pressure <130 mmHg and diastolic pressure <80 mmHg) or glycaemia (haemoglobin A1c ≤7% or fasting plasma glucose <7 mmol/L [126 mg/dL]) to 60% or 80% of those treated for hypertension or diabetes, respectively. The retinopathy end-point was severe vision loss, defined as <20/200 visual acuity by Snellen chart. Region abbreviations: CAR = Caribbean; CASIA = Central Asia; CLA = Central Latin America; EEUR = Eastern Europe; ESSA = Eastern Sub-Saharan Africa; NAME = North Africa/Middle East; OCN = Oceania; SASIA = South Asia; SEASIA = Southeast Asia; SLA = South Latin America; SSSA = South Sub-Saharan Africa; WSSA = Western Sub-Saharan Africa.

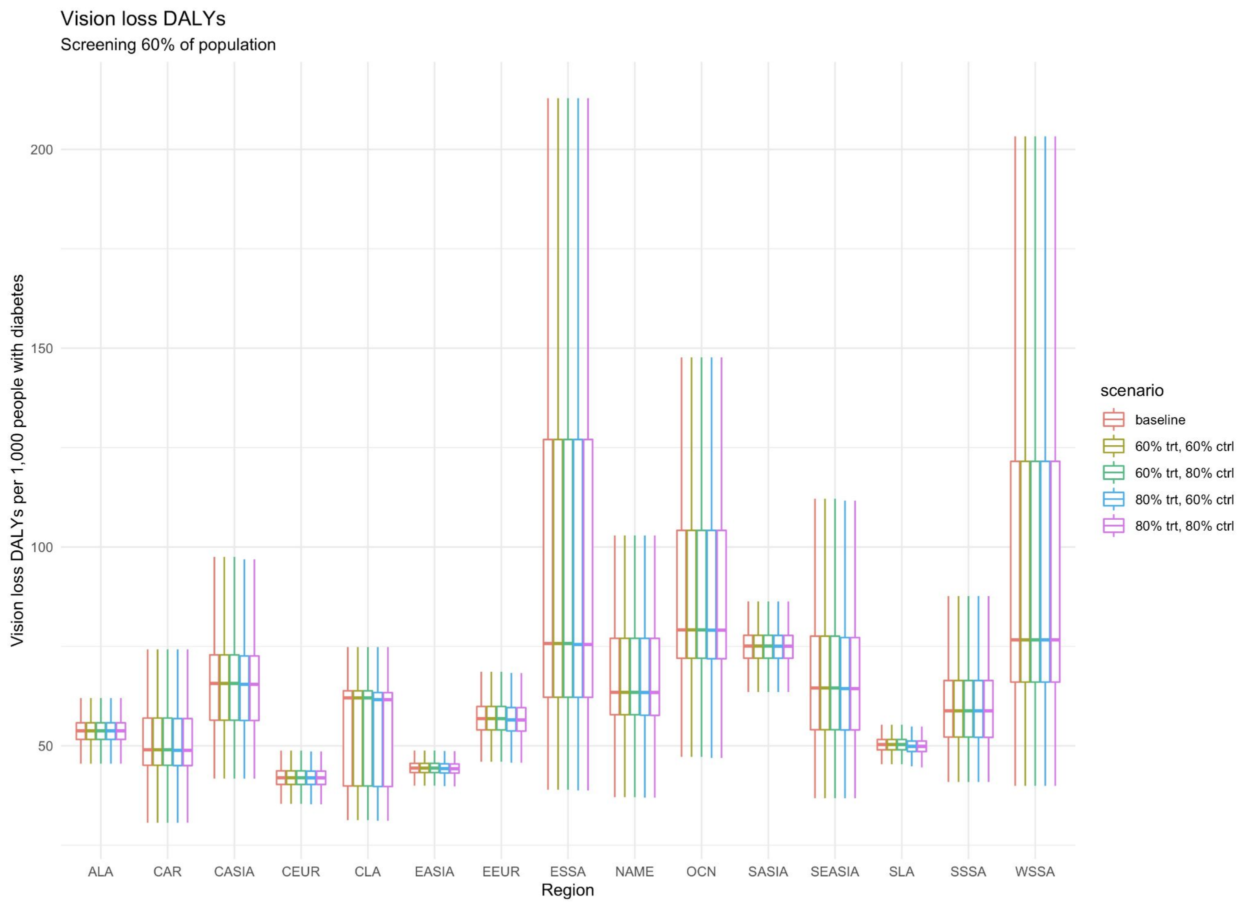

Vision loss DALYs
Screening 60% of population
Vision loss DALYs per 1,000 people with diabetes
200
150
100
50
ALA
CAR
CASIA
CEUR
CLA
EASIA
EEUR
ESSA
NAME
OCN
SASIA
SEASIA
SLA
SSSA
WSSA
Region
scenario
baseline
60% trt, 60% ctrl
60% trt, 80% ctrl
80% trt, 60% ctrl
80% trt, 80% ctrl

Appendix Figure 30: Estimated DALYs lost to retinopathy events with screening for 80% diagnosis and with increased treatment and control. We brought the proportion of people with diabetes who were diagnosed up to 80% within each country's population, and the portion of those with diabetes and hypertension who were diagnosed with hypertension up to 80%, leaving unaltered those countries with a baseline level above these proportions The figure displays increases in treatment (trt) with blood pressure, glycaemic, or statin medicines to 60% or 80% of those diagnosed with hypertension or diabetes or indicated for statins (age 40 or older or >20% estimated 10-year risk of cardiovascular events), respectively; and control (ctrl) of blood pressure (systolic pressure <130 mmHg and diastolic pressure <80 mmHg) or glycaemia (haemoglobin A1c ≤7% or fasting plasma glucose <7 mmol/L [126 mg/dL]) to 60% or 80% of those treated for hypertension or diabetes, respectively. The retinopathy end-point was severe vision loss, defined as <20/200 visual acuity by Snellen chart. Region abbreviations: CAR = Caribbean; CASIA = Central Asia; CLA = Central Latin America; EEUR = Eastern Europe; ESSA = Eastern Sub-Saharan Africa; NAME = North Africa/Middle East; OCN = Oceania; SASIA = South Asia; SEASIA = Southeast Asia; SLA = South Latin America; SSSA = South Sub-Saharan Africa; WSSA = Western Sub-Saharan Africa.

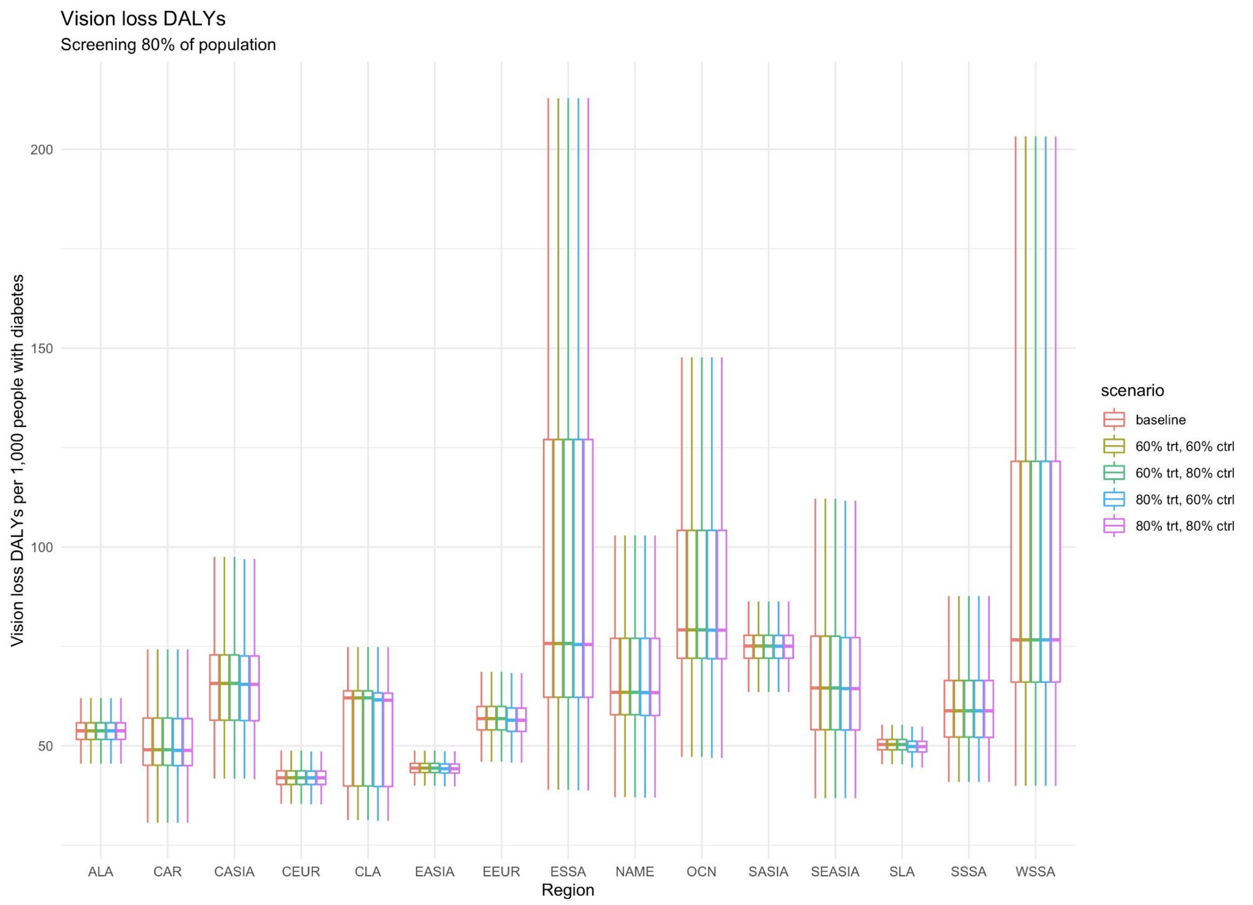

Vision loss DALYs
Screening 80% of population
Vision loss DALYs per 1,000 people with diabetes
200
150
100
50
ALA
CAR
CASIA
CEUR
CLA
EASIA
EEUR
ESSA
NAME
OCN
SASIA
SEASIA
SLA
SSSA
WSSA
Region
scenario
baseline
60% trt, 60% ctrl
60% trt, 80% ctrl
80% trt, 60% ctrl
80% trt, 80% ctrl

Appendix Figure 31: Estimated deaths from cardiovascular disease (CVD) without new screening but with increased treatment and control. The figure displays increases in treatment (trt) with blood pressure, glycaemic, or statin medicines to 60% or 80% of those diagnosed with hypertension or diabetes or indicated for statins (age 40 or older or >20% estimated 10-year risk of cardiovascular events), respectively; and control (ctrl) of blood pressure (systolic pressure <130 mmHg and diastolic pressure <80 mmHg) or glycaemia (haemoglobin A1c ≤7% or fasting plasma glucose <7 mmol/L [126 mg/dL]) to 60% or 80% of those treated for hypertension or diabetes, respectively. Cardiovascular disease was defined as fatal and non-fatal myocardial infarction and stroke. Region abbreviations: CAR = Caribbean; CASIA = Central Asia; CLA = Central Latin America; EEUR = Eastern Europe; ESSA = Eastern Sub-Saharan Africa; NAME = North Africa/Middle East; OCN = Oceania; SASIA = South Asia; SEASIA = Southeast Asia; SLA = South Latin America; SSSA = South Sub-Saharan Africa; WSSA = Western Sub-Saharan Africa.

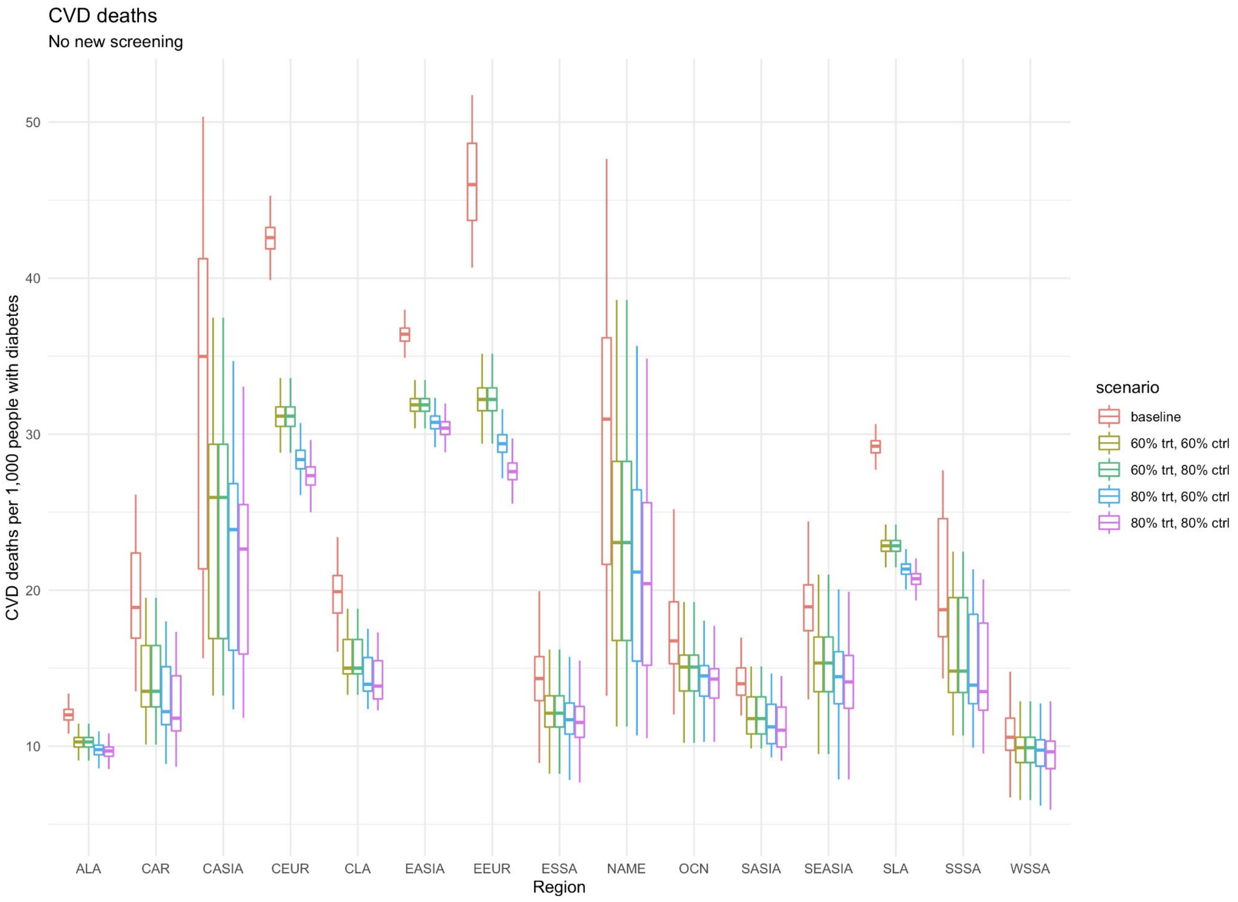

Appendix Figure 32: Estimated deaths from cardiovascular disease (CVD) with screening for 60% diagnosis and with increased treatment and control. We brought the proportion of people with diabetes who were diagnosed up to 60% within each country's population, and the portion of those with diabetes and hypertension who were diagnosed with hypertension up to 60%, leaving unaltered those countries with a baseline level above these proportions The figure displays increases in treatment (trt) with blood pressure, glycaemic, or statin medicines to 60% or 80% of those diagnosed with hypertension or diabetes or indicated for statins (age 40 or older or >20% estimated 10-year risk of cardiovascular events), respectively; and control (ctrl) of blood pressure (systolic pressure <130 mmHg and diastolic pressure <80 mmHg) or glycaemia (haemoglobin A1c ≤7% or fasting plasma glucose <7 mmol/L [126 mg/dL]) to 60% or 80% of those treated for hypertension or diabetes, respectively. Cardiovascular disease was defined as fatal and non-fatal myocardial infarction and stroke. Region abbreviations: CAR = Caribbean; CASIA = Central Asia; CLA = Central Latin America; EEUR = Eastern Europe; ESSA = Eastern Sub-Saharan Africa; NAME = North Africa/Middle East; OCN = Oceania; SASIA = South Asia; SEASIA = Southeast Asia; SLA = South Latin America; SSSA = South Sub-Saharan Africa; WSSA = Western Sub-Saharan Africa.

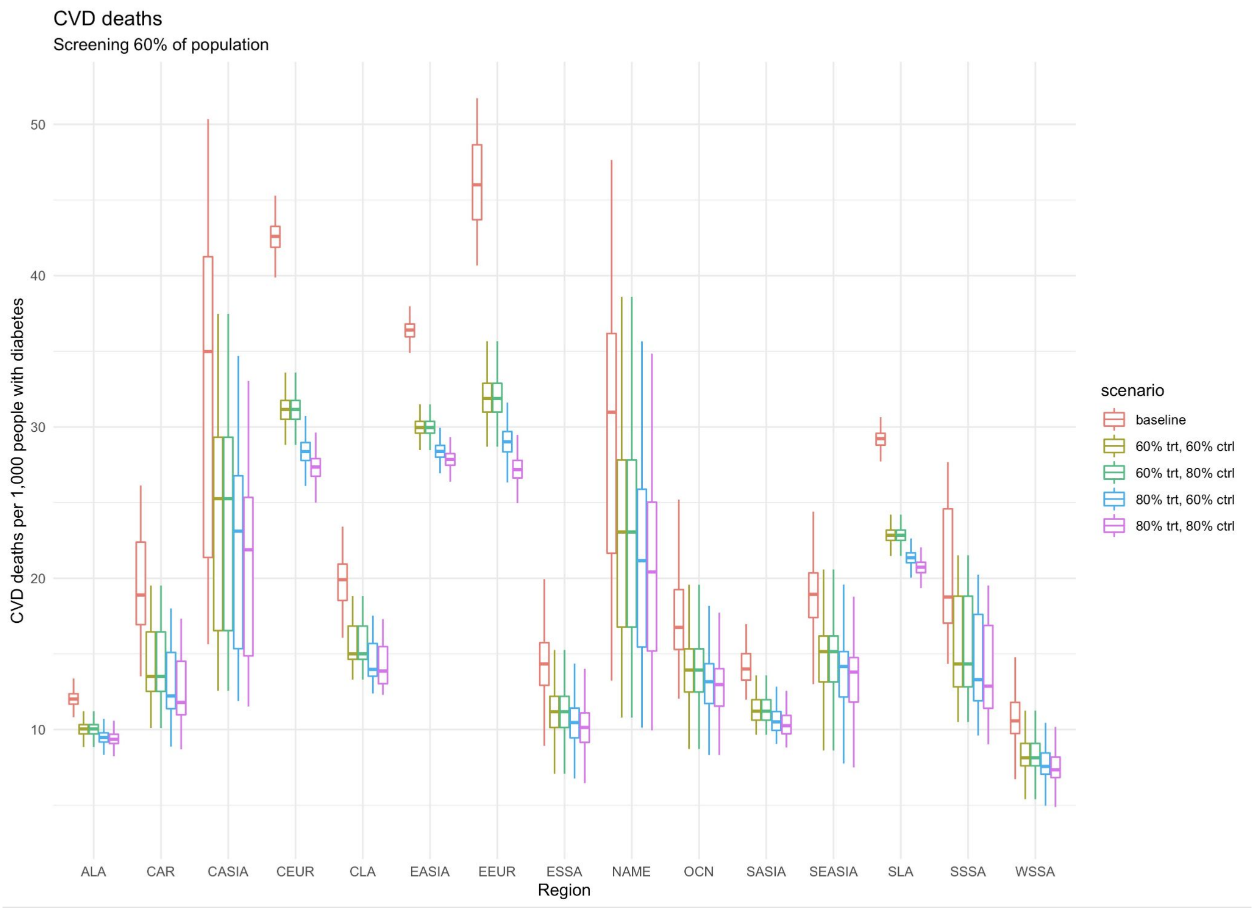

CVD deaths
Screening 60% of population
CVD deaths per 1,000 people with diabetes
50
40
30
20
10
ALA
CAR
CASIA
CEUR
CLA
EASIA
EEUR
ESSA
NAME
OCN
SASIA
SEASIA
SLA
SSSA
WSSA
Region
scenario
baseline
60% trt, 60% ctrl
60% trt, 80% ctrl
80% trt, 60% ctrl
80% trt, 80% ctrl

Appendix Figure 33: Estimated deaths from cardiovascular disease (CVD) with screening for 80% diagnosis and with increased treatment and control. We brought the proportion of people with diabetes who were diagnosed up to 80% within each country's population, and the portion of those with diabetes and hypertension who were diagnosed with hypertension up to 80%, leaving unaltered those countries with a baseline level above these proportions The figure displays increases in treatment (trt) with blood pressure, glycaemic, or statin medicines to 60% or 80% of those diagnosed with hypertension or diabetes or indicated for statins (age 40 or older or >20% estimated 10-year risk of cardiovascular events), respectively; and control (ctrl) of blood pressure (systolic pressure <130 mmHg and diastolic pressure <80 mmHg) or glycaemia (haemoglobin A1c ≤7% or fasting plasma glucose <7 mmol/L [126 mg/dL]) to 60% or 80% of those treated for hypertension or diabetes, respectively. Cardiovascular disease was defined as fatal and non-fatal myocardial infarction and stroke. Region abbreviations: CAR = Caribbean; CASIA = Central Asia; CLA = Central Latin America; EEUR = Eastern Europe; ESSA = Eastern Sub-Saharan Africa; NAME = North Africa/Middle East; OCN = Oceania; SASIA = South Asia; SEASIA = Southeast Asia; SLA = South Latin America; SSSA = South Sub-Saharan Africa; WSSA = Western Sub-Saharan Africa.

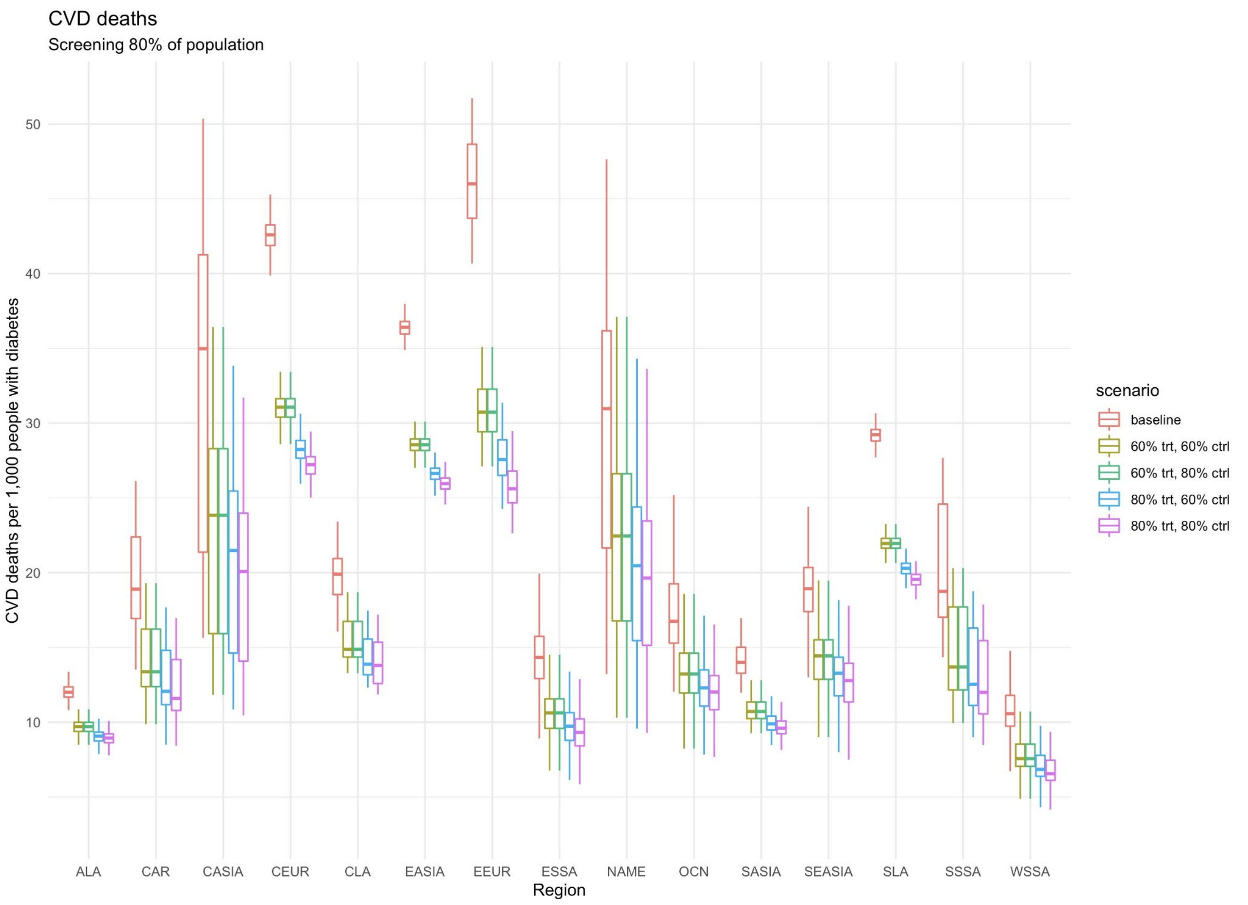
CVD deaths
Screening 80% of population
CVD deaths per 1,000 people with diabetes
50
40
30
20
10
ALA
CAR
CASIA
CEUR
CLA
EASIA
EEUR
ESSA
NAME
OCN
SASIA
SEASIA
SLA
SSSA
WSSA
Region
scenario
baseline
60% trt, 60% ctrl
60% trt, 80% ctrl
80% trt, 60% ctrl
80% trt, 80% ctrl

Appendix Figure 34: Estimated deaths from congestive heart failure (CHF) without new screening but with increased treatment and control. The figure displays increases in treatment (trt) with blood pressure, glycaemic, or statin medicines to 60% or 80% of those diagnosed with hypertension or diabetes or indicated for statins (age 40 or older or >20% estimated 10-year risk of cardiovascular events), respectively; and control (ctrl) of blood pressure (systolic pressure <130 mmHg and diastolic pressure <80 mmHg) or glycaemia (haemoglobin A1c ≤7% or fasting plasma glucose <7 mmol/L [126 mg/dL]) to 60% or 80% of those treated for hypertension or diabetes, respectively. Congestive heart failure was defined as an ejection fraction of <40%, with New York Heart Association class III or IV functional limitations. Region abbreviations: CAR = Caribbean; CASIA = Central Asia; CLA = Central Latin America; EEUR = Eastern Europe; ESSA = Eastern Sub-Saharan Africa; NAME = North Africa/Middle East; OCN = Oceania; SASIA = South Asia; SEASIA = Southeast Asia; SLA = South Latin America; SSSA = South Sub-Saharan Africa; WSSA = Western Sub-Saharan Africa.

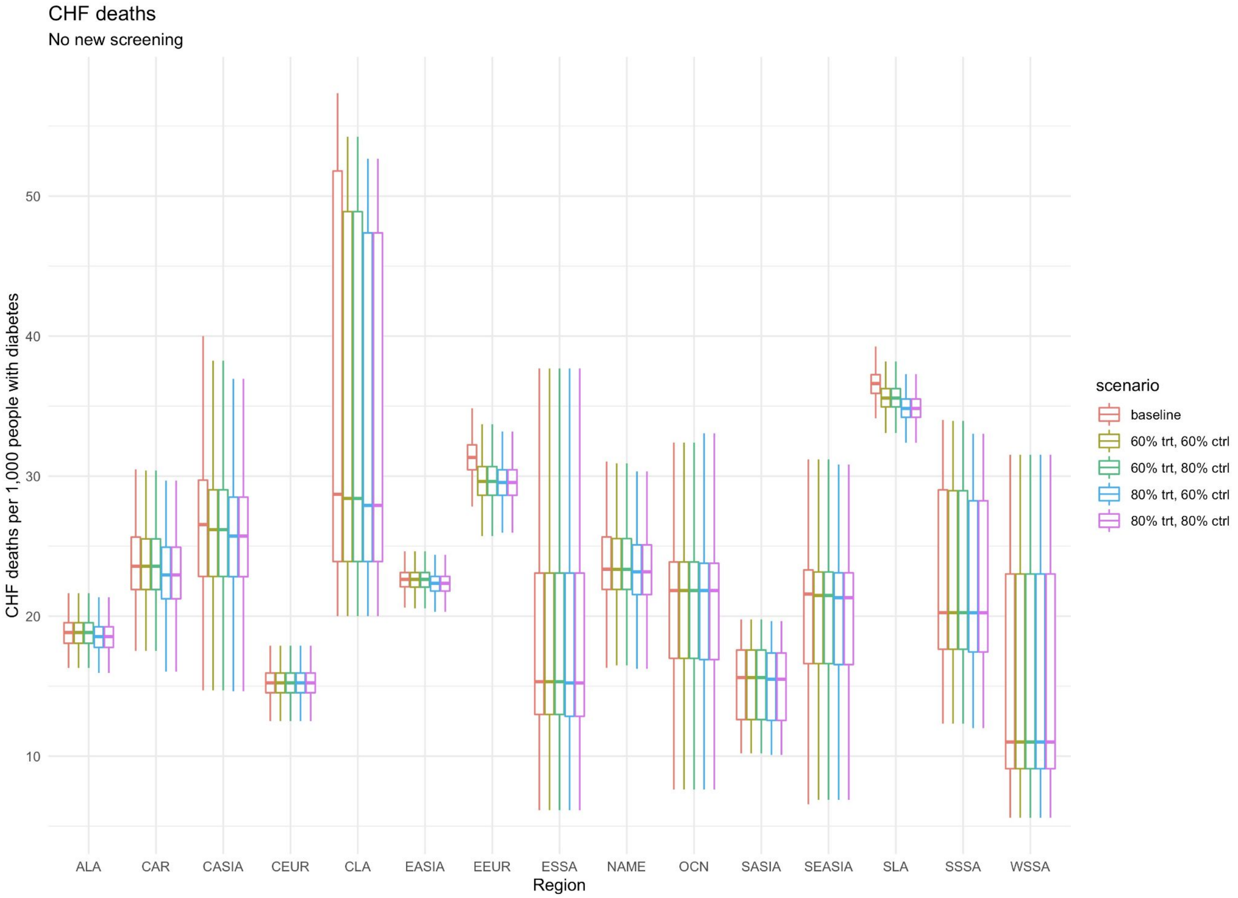

Appendix Figure 35: Estimated DALYs lost to congestive heart failure (CHF) with screening for 60% diagnosis and with increased treatment and control. We brought the proportion of people with diabetes who were diagnosed up to 60% within each country's population, and the portion of those with diabetes and hypertension who were diagnosed with hypertension up to 60%, leaving unaltered those countries with a baseline level above these proportions The figure displays increases in treatment (trt) with blood pressure, glycaemic, or statin medicines to 60% or 80% of those diagnosed with hypertension or diabetes or indicated for statins (age 40 or older or >20% estimated 10-year risk of cardiovascular events), respectively; and control (ctrl) of blood pressure (systolic pressure <130 mmHg and diastolic pressure <80 mmHg) or glycaemia (haemoglobin A1c ≤7% or fasting plasma glucose <7 mmol/L [126 mg/dL]) to 60% or 80% of those treated for hypertension or diabetes, respectively. Congestive heart failure was defined as an ejection fraction of <40%, with New York Heart Association class III or IV functional limitations. Region abbreviations: CAR = Caribbean; CASIA = Central Asia; CLA = Central Latin America; EEUR = Eastern Europe; ESSA = Eastern Sub-Saharan Africa; NAME = North Africa/Middle East; OCN = Oceania; SASIA = South Asia; SEASIA = Southeast Asia; SLA = South Latin America; SSSA = South Sub-Saharan Africa; WSSA = Western Sub-Saharan Africa.

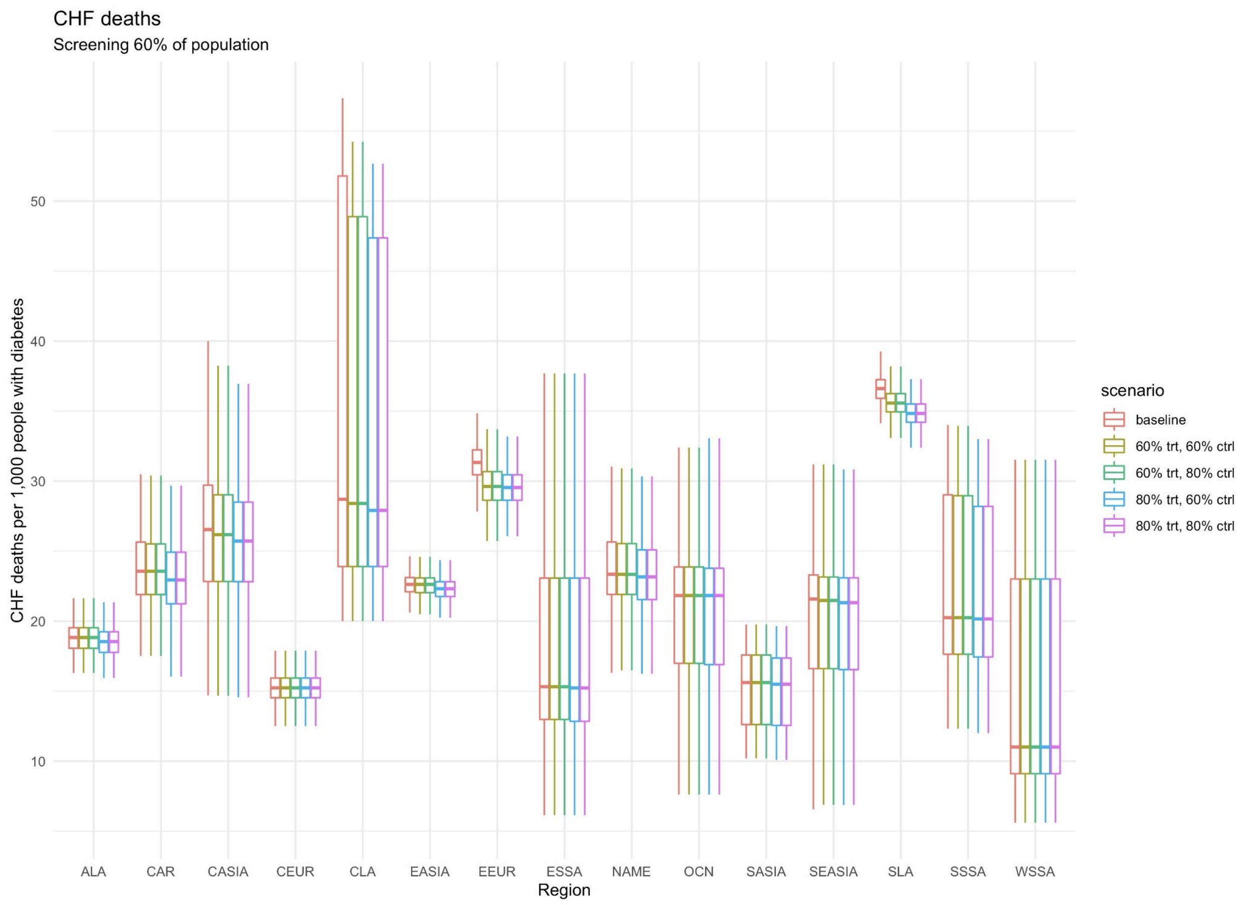
CHF deaths
Screening 60% of population
CHF deaths per 1,000 people with diabetes
50
40
30
20
10
ALA
CAR
CASIA
CEUR
CLA
EASIA
EEUR
ESSA
NAME
OCN
SASIA
SEASIA
SLA
SSSA
WSSA
Region
scenario
baseline
60% trt, 60% ctrl
60% trt, 80% ctrl
80% trt, 60% ctrl
80% trt, 80% ctrl

Appendix Figure 36: Estimated deaths from congestive heart failure (CHF) with screening for 80% diagnosis and with increased treatment and control. We brought the proportion of people with diabetes who were diagnosed up to 80% within each country's population, and the portion of those with diabetes and hypertension who were diagnosed with hypertension up to 80%, leaving unaltered those countries with a baseline level above these proportions The figure displays increases in treatment (trt) with blood pressure, glycaemic, or statin medicines to 60% or 80% of those diagnosed with hypertension or diabetes or indicated for statins (age 40 or older or >20% estimated 10-year risk of cardiovascular events), respectively; and control (ctrl) of blood pressure (systolic pressure <130 mmHg and diastolic pressure <80 mmHg) or glycaemia (haemoglobin A1c ≤7% or fasting plasma glucose <7 mmol/L [126 mg/dL]) to 60% or 80% of those treated for hypertension or diabetes, respectively. Congestive heart failure was defined as an ejection fraction of <40%, with New York Heart Association class III or IV functional limitations. Region abbreviations: CAR = Caribbean; CASIA = Central Asia; CLA = Central Latin America; EEUR = Eastern Europe; ESSA = Eastern Sub-Saharan Africa; NAME = North Africa/Middle East; OCN = Oceania; SASIA = South Asia; SEASIA = Southeast Asia; SLA = South Latin America; SSSA = South Sub-Saharan Africa; WSSA = Western Sub-Saharan Africa.

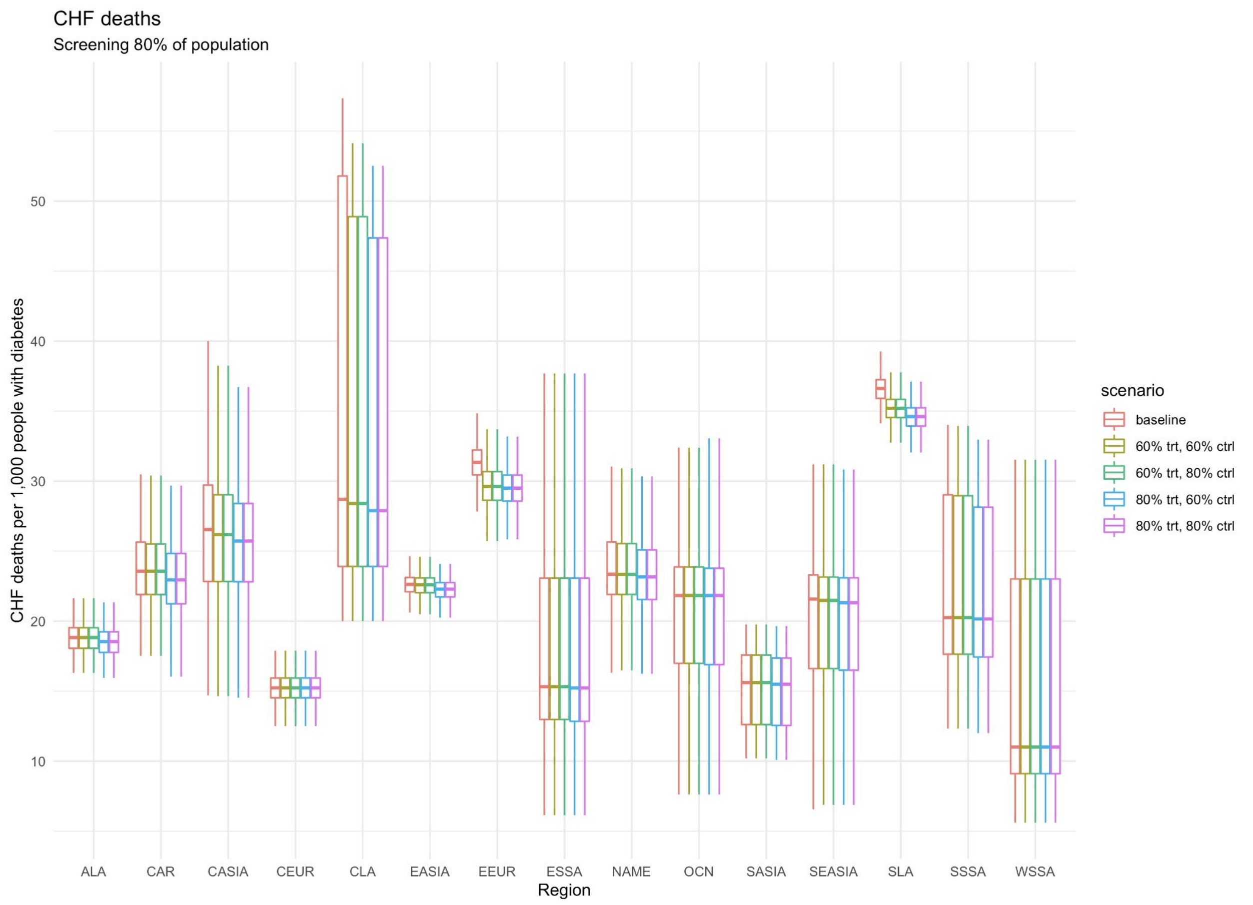

Appendix Figure 37: Estimated deaths from end-stage renal disease (ESRD) without new screening but with increased treatment and control. The figure displays increases in treatment (trt) with blood pressure, glycaemic, or statin medicines to 60% or 80% of those diagnosed with hypertension or diabetes or indicated for statins (age 40 or older or >20% estimated 10-year risk of cardiovascular events), respectively; and control (ctrl) of blood pressure (systolic pressure <130 mmHg and diastolic pressure <80 mmHg) or glycaemia

(haemoglobin A1c ≤7% or fasting plasma glucose <7 mmol/L [126 mg/dL]) to 60% or 80% of those treated for hypertension or diabetes, respectively. End-stage renal disease (ESRD) was defined as estimated glomerular filtration rate <15 mL/min/1.73m^2 or needing dialysis/transplant. Region abbreviations: CAR = Caribbean; CASIA = Central Asia; CLA = Central Latin America; EEUR = Eastern Europe; ESSA = Eastern Sub-Saharan Africa; NAME = North Africa/Middle East; OCN = Oceania; SASIA = South Asia; SEASIA = Southeast Asia; SLA = South Latin America; SSSA = South Sub-Saharan Africa; WSSA = Western Sub-Saharan Africa.

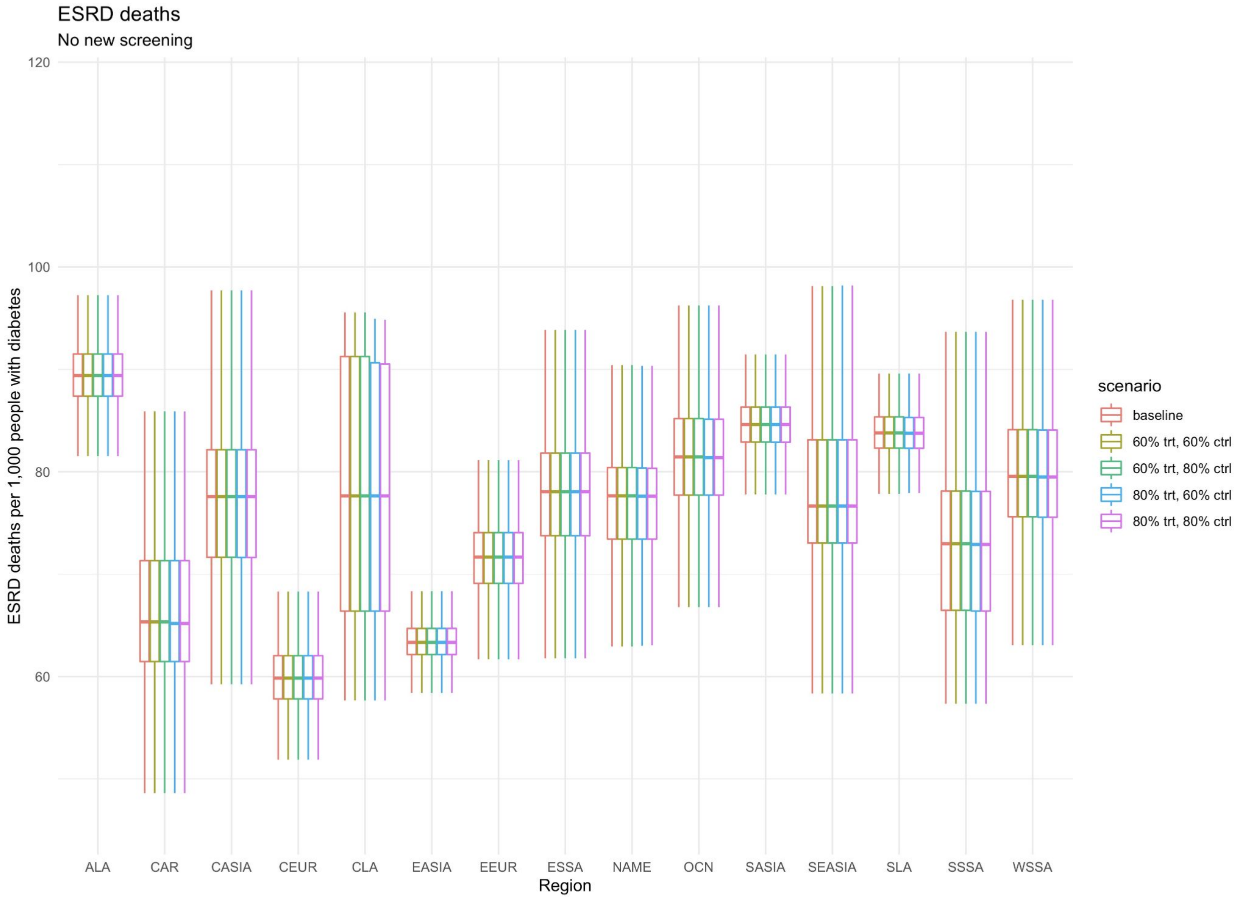

Appendix Figure 38: Estimated deaths from end-stage renal disease (ESRD) events with screening for 60% diagnosis and with increased treatment and control. We brought the proportion of people with diabetes who were diagnosed up to 60% within each country's population, and the portion of those with diabetes and hypertension who were diagnosed with hypertension up to 60%, leaving unaltered those countries with a baseline level above these proportions The figure displays increases in treatment (trt) with blood pressure, glycaemic, or statin medicines to 60% or 80% of those diagnosed with hypertension or diabetes or indicated for statins (age 40 or older or >20% estimated 10-year risk of cardiovascular events), respectively; and control (ctrl) of blood pressure (systolic pressure <130 mmHg and diastolic pressure <80 mmHg) or glycaemia (haemoglobin A1c ≤7% or fasting plasma glucose <7 mmol/L [126 mg/dL]) to 60% or 80% of those treated for hypertension or diabetes, respectively. End-stage renal disease (ESRD) was defined as estimated glomerular filtration rate <15 mL/min/1.73m^2 or needing dialysis/transplant. Region abbreviations: CAR = Caribbean; CASIA = Central Asia; CLA = Central Latin America; EEUR = Eastern Europe; ESSA = Eastern Sub-Saharan Africa; NAME = North Africa/Middle East; OCN = Oceania; SASIA = South Asia; SEASIA = Southeast Asia; SLA = South Latin America; SSSA = South Sub-Saharan Africa; WSSA = Western Sub-Saharan Africa.

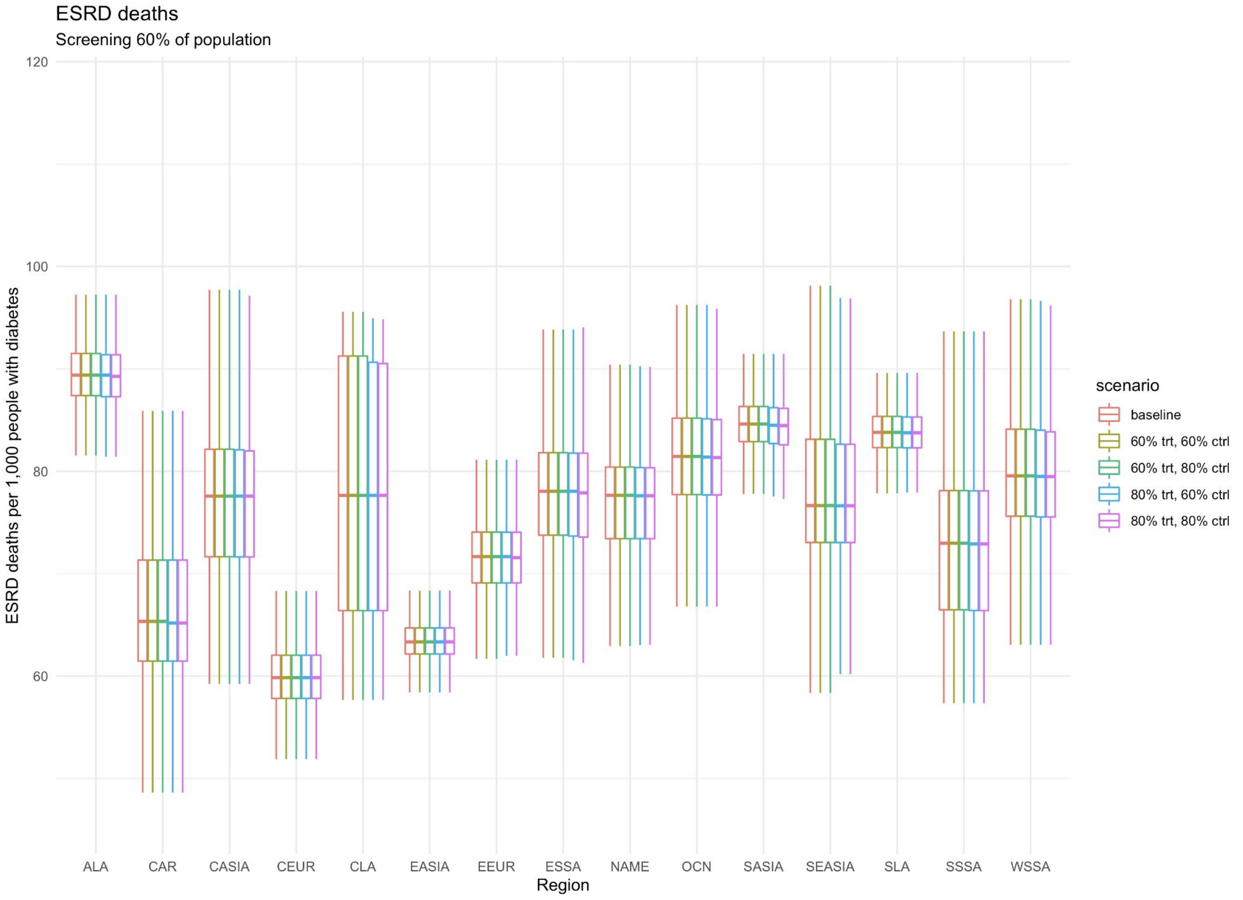
ESRD deaths
Screening 60% of population
ESRD deaths per 1,000 people with diabetes
120
100
80
60
ALA
CAR
CASIA
CEUR
CLA
EASIA
EEUR
ESSA
NAME
OCN
SASIA
SEASIA
SLA
SSSA
WSSA
Region
scenario
baseline
60% trt, 60% ctrl
60% trt, 80% ctrl
80% trt, 60% ctrl
80% trt, 80% ctrl

Appendix Figure 39: Estimated deaths from end-stage renal disease (ESRD) with screening for 80% diagnosis and with increased treatment and control. We brought the proportion of people with diabetes who were diagnosed up to 80% within each country's population, and the portion of those with diabetes and hypertension who were diagnosed with hypertension up to 80%, leaving unaltered those countries with a baseline level above these proportions The figure displays increases in treatment (trt) with blood pressure, glycaemic, or statin medicines to 60% or 80% of those diagnosed with hypertension or diabetes or indicated for statins (age 40 or older or >20% estimated 10-year risk of cardiovascular events), respectively; and control (ctrl) of blood pressure (systolic pressure <130 mmHg and diastolic pressure <80 mmHg) or glycaemia (haemoglobin A1c ≤7% or fasting plasma glucose <7 mmol/L [126 mg/dL]) to 60% or 80% of those treated for hypertension or diabetes, respectively. End-stage renal disease (ESRD) was defined as estimated glomerular filtration rate <15 mL/min/1.73m^2 or needing dialysis/transplant. Region abbreviations: CAR = Caribbean; CASIA = Central Asia; CLA = Central Latin America; EEUR = Eastern Europe; ESSA = Eastern Sub-Saharan Africa; NAME = North Africa/Middle East; OCN = Oceania; SASIA = South Asia; SEASIA = Southeast Asia; SLA = South Latin America; SSSA = South Sub-Saharan Africa; WSSA = Western Sub-Saharan Africa.

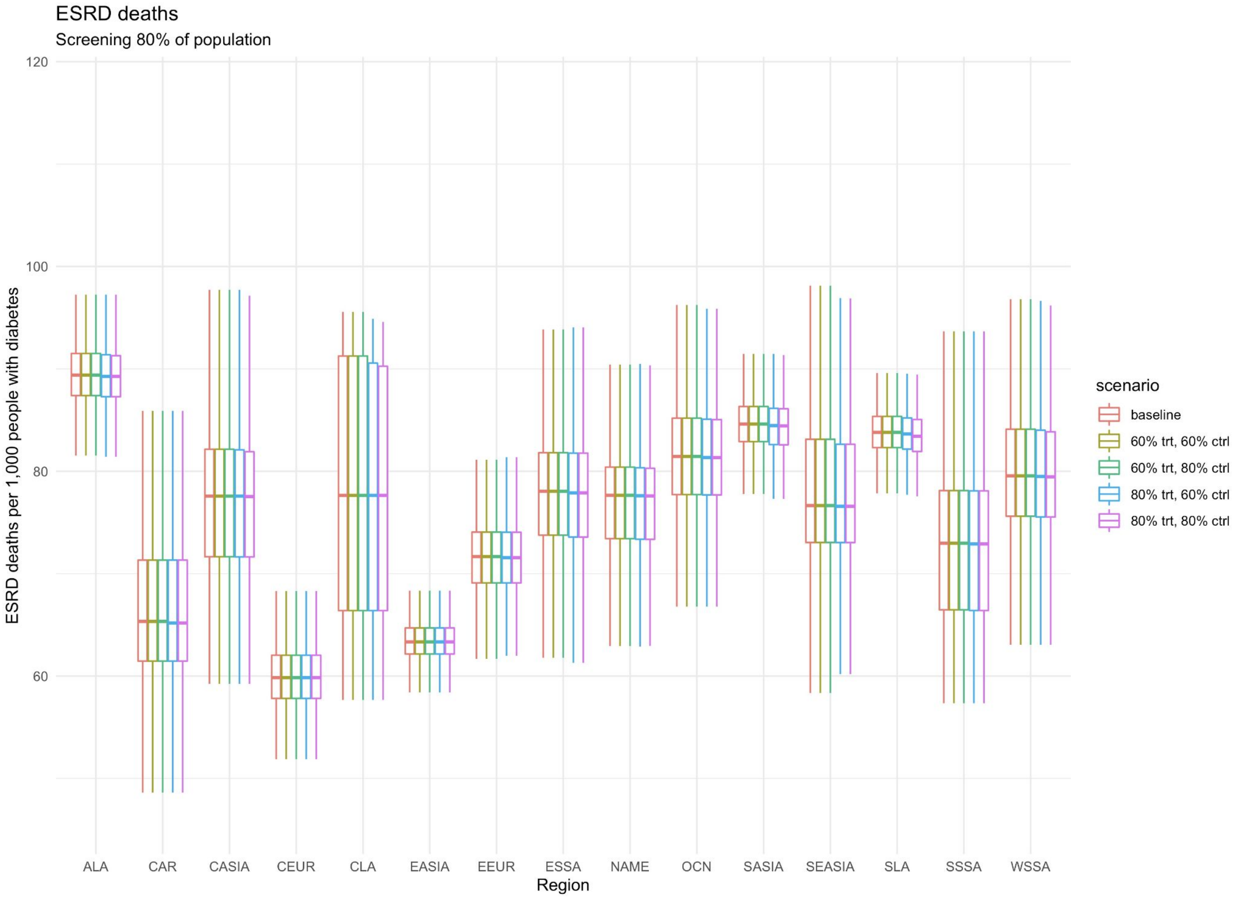
ESRD deaths
Screening 80% of population
ESRD deaths per 1,000 people with diabetes
120
100
80
60
ALA
CAR
CASIA
CEUR
CLA
EASIA
EEUR
ESSA
NAME
OCN
SASIA
SEASIA
SLA
SSSA
WSSA
Region
scenario
baseline
60% trt, 60% ctrl
60% trt, 80% ctrl
80% trt, 60% ctrl
80% trt, 80% ctrl

Appendix Figure 40: Estimated deaths from neuropathy without new screening but with increased treatment and control. The figure displays increases in treatment (trt) with blood pressure, glycaemic, or statin medicines to 60% or 80% of those diagnosed with hypertension or diabetes or indicated for statins (age 40 or older or >20% estimated 10-year risk of cardiovascular events), respectively; and control (ctrl) of blood pressure (systolic pressure <130 mmHg and diastolic pressure <80 mmHg) or glycaemia (haemoglobin A1c ≤7% or fasting plasma glucose <7 mmol/L [126 mg/dL]) to 60% or 80% of those treated for hypertension or diabetes, respectively. Neuropathy was defined as pressure sensation loss by Semmes-Weinstein 5.07/10 gram monofilament exam. Region abbreviations: CAR = Caribbean; CASIA = Central Asia; CLA = Central Latin America; EEUR = Eastern Europe; ESSA = Eastern Sub-Saharan Africa; NAME = North Africa/Middle East; OCN = Oceania; SASIA = South Asia; SEASIA = Southeast Asia; SLA = South Latin America; SSSA = South Sub-Saharan Africa; WSSA = Western Sub-Saharan Africa.

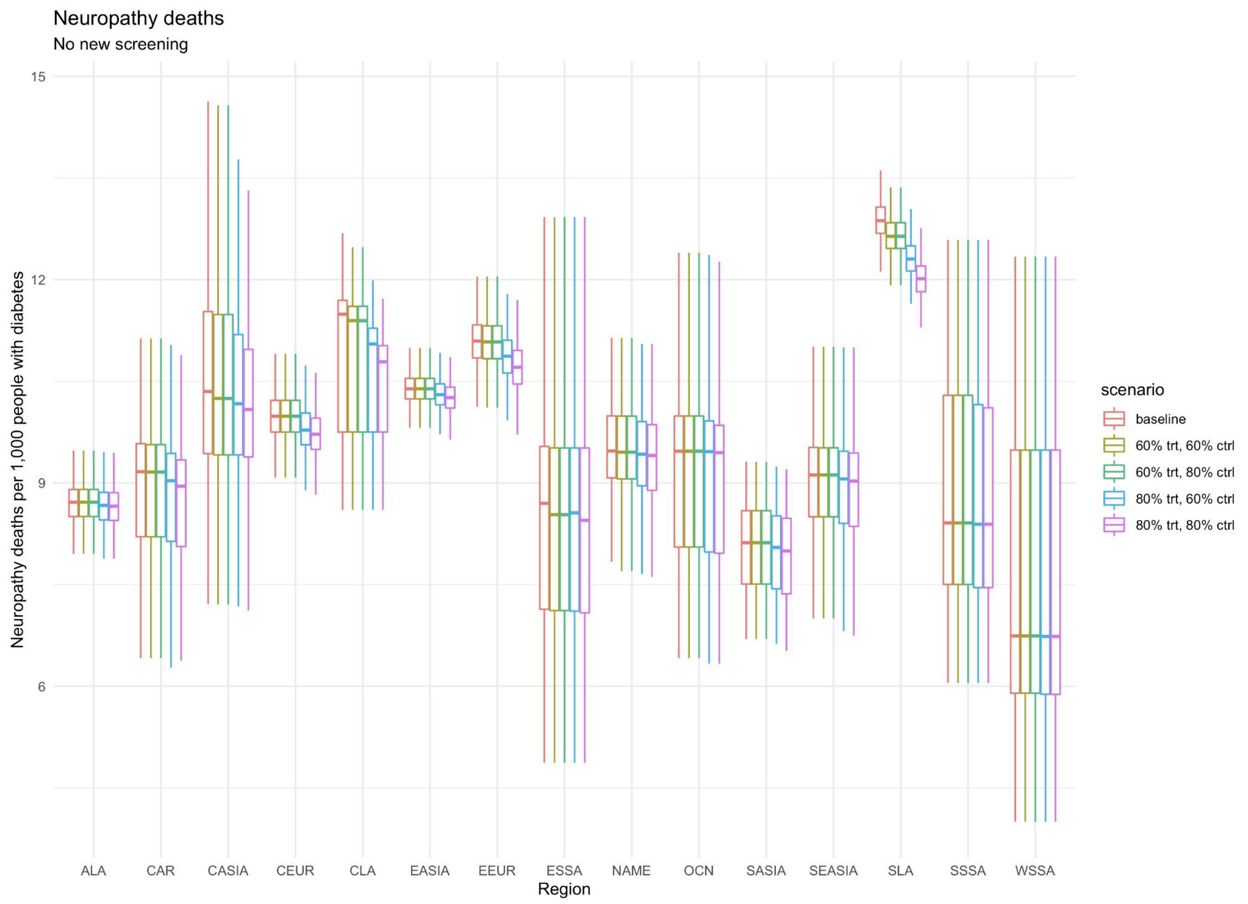

Appendix Figure 41: Estimated deaths from neuropathy with screening for 60% diagnosis and with increased treatment and control. We brought the proportion of people with diabetes who were diagnosed up to 60% within each country's population, and the portion of those with diabetes and hypertension who were diagnosed with hypertension up to 60%, leaving unaltered those countries with a baseline level above these proportions The figure displays increases in treatment (trt) with blood pressure, glycaemic, or statin medicines to 60% or 80% of those diagnosed with hypertension or diabetes or indicated for statins (age 40 or older or >20% estimated 10-year risk of cardiovascular events), respectively; and control (ctrl) of blood pressure (systolic pressure <130 mmHg and diastolic pressure <80 mmHg) or glycaemia (haemoglobin A1c ≤7% or fasting plasma glucose <7 mmol/L [126 mg/dL]) to 60% or 80% of those treated for hypertension or diabetes, respectively. Neuropathy was defined as pressure sensation loss by Semmes-Weinstein 5.07/10 gram monofilament exam. Region abbreviations: CAR = Caribbean; CASIA = Central Asia; CLA = Central Latin America; EEUR = Eastern Europe; ESSA = Eastern Sub-Saharan Africa; NAME = North Africa/Middle East; OCN = Oceania; SASIA = South Asia; SEASIA = Southeast Asia; SLA = South Latin America; SSSA = South Sub-Saharan Africa; WSSA = Western Sub-Saharan Africa.

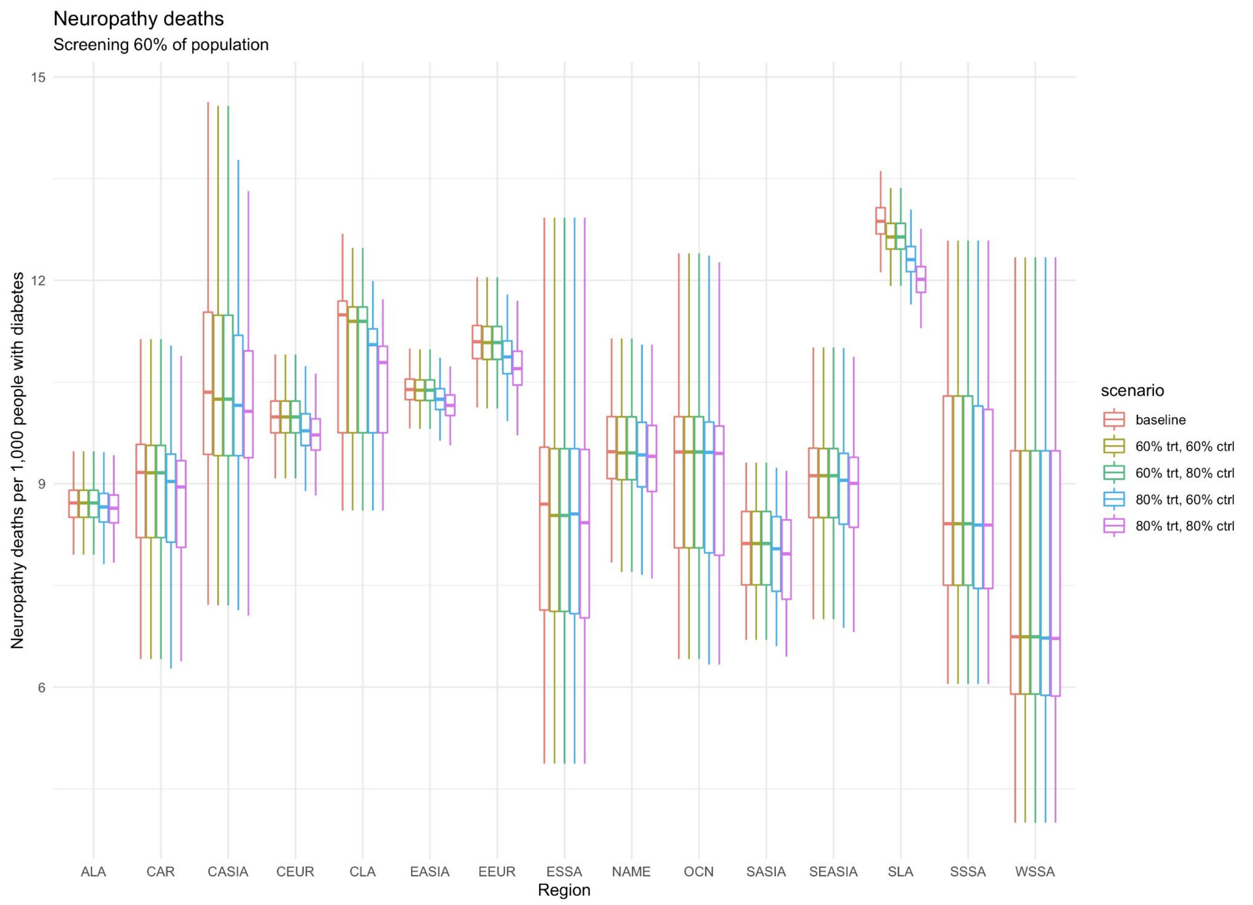
Neuropathy deaths
Screening 60% of population
Neuropathy deaths per 1,000 people with diabetes
15
12
9
6
ALA
CAR
CASIA
CEUR
CLA
EASIA
EEUR
ESSA
NAME
OCN
SASIA
SEASIA
SLA
SSSA
WSSA
Region
scenario
baseline
60% trt, 60% ctrl
60% trt, 80% ctrl
80% trt, 60% ctrl
80% trt, 80% ctrl

Appendix Figure 42: Estimated deaths from neuropathy with screening for 80% diagnosis and with increased treatment and control. We brought the proportion of people with diabetes who were diagnosed up to 80% within each country's population, and the portion of those with diabetes and hypertension who were diagnosed with hypertension up to 80%, leaving unaltered those countries with a baseline level above these proportions The figure displays increases in treatment (trt) with blood pressure, glycaemic, or statin medicines to 60% or 80% of those diagnosed with hypertension or diabetes or indicated for statins (age 40 or older or >20% estimated 10-year risk of cardiovascular events), respectively; and control (ctrl) of blood pressure (systolic pressure <130 mmHg and diastolic pressure <80 mmHg) or glycaemia (haemoglobin A1c ≤7% or fasting plasma glucose <7 mmol/L [126 mg/dL]) to 60% or 80% of those treated for hypertension or diabetes, respectively. Neuropathy was defined as pressure sensation loss by Semmes-Weinstein 5.07/10 gram monofilament exam. Region abbreviations: CAR = Caribbean; CASIA = Central Asia; CLA = Central Latin America; EEUR = Eastern Europe; ESSA = Eastern Sub-Saharan Africa; NAME = North Africa/Middle East; OCN = Oceania; SASIA = South Asia; SEASIA = Southeast Asia; SLA = South Latin America; SSSA = South Sub-Saharan Africa; WSSA = Western Sub-Saharan Africa.

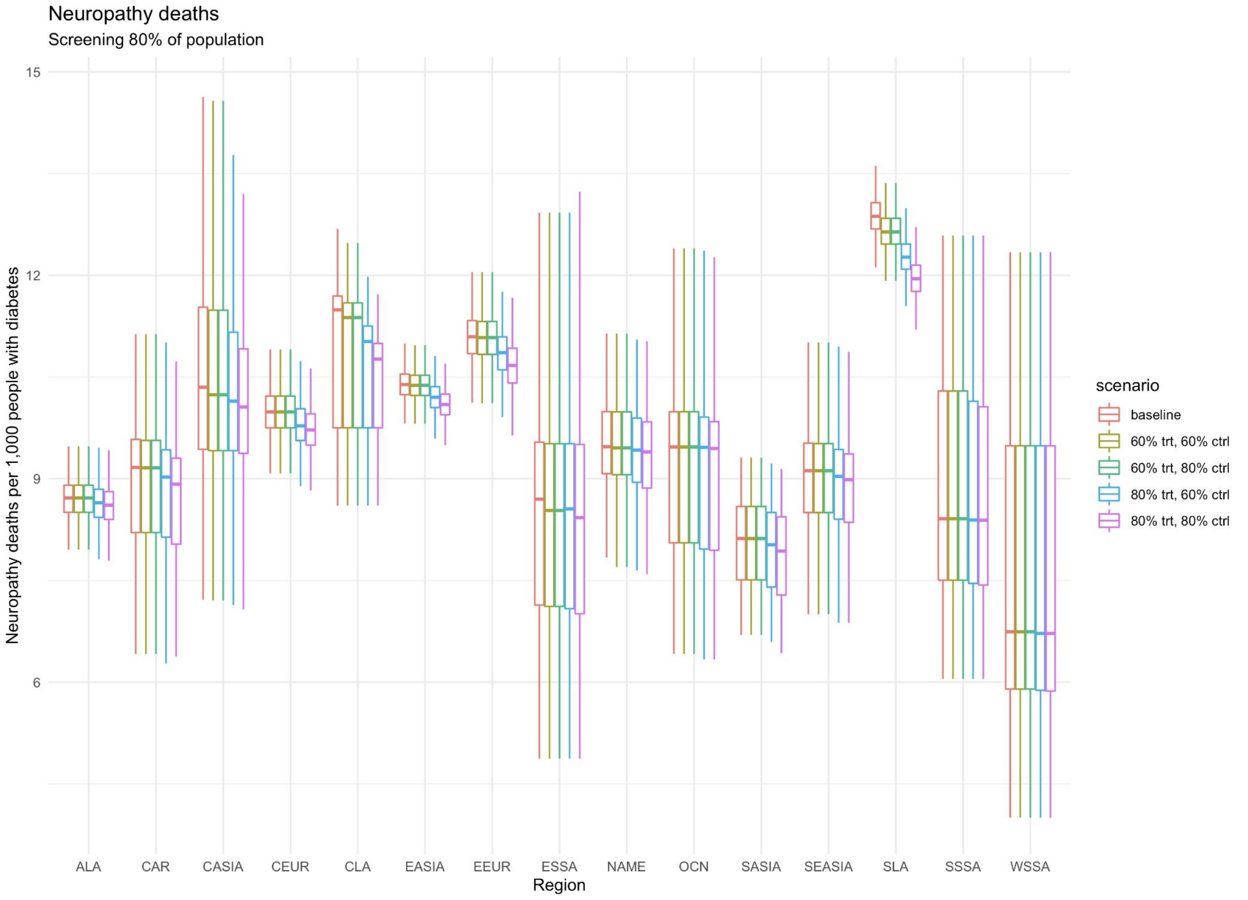
Neuropathy deaths
Screening 80% of population
Neuropathy deaths per 1,000 people with diabetes
15
12
9
6
ALA
CAR
CASIA
CEUR
CLA
EASIA
EEUR
ESSA
NAME
OCN
SASIA
SEASIA
SLA
SSSA
WSSA
Region
scenario
baseline
60% trt, 60% ctrl
60% trt, 80% ctrl
80% trt, 60% ctrl
80% trt, 80% ctrl

Appendix Figure 43: Survey flow chart.

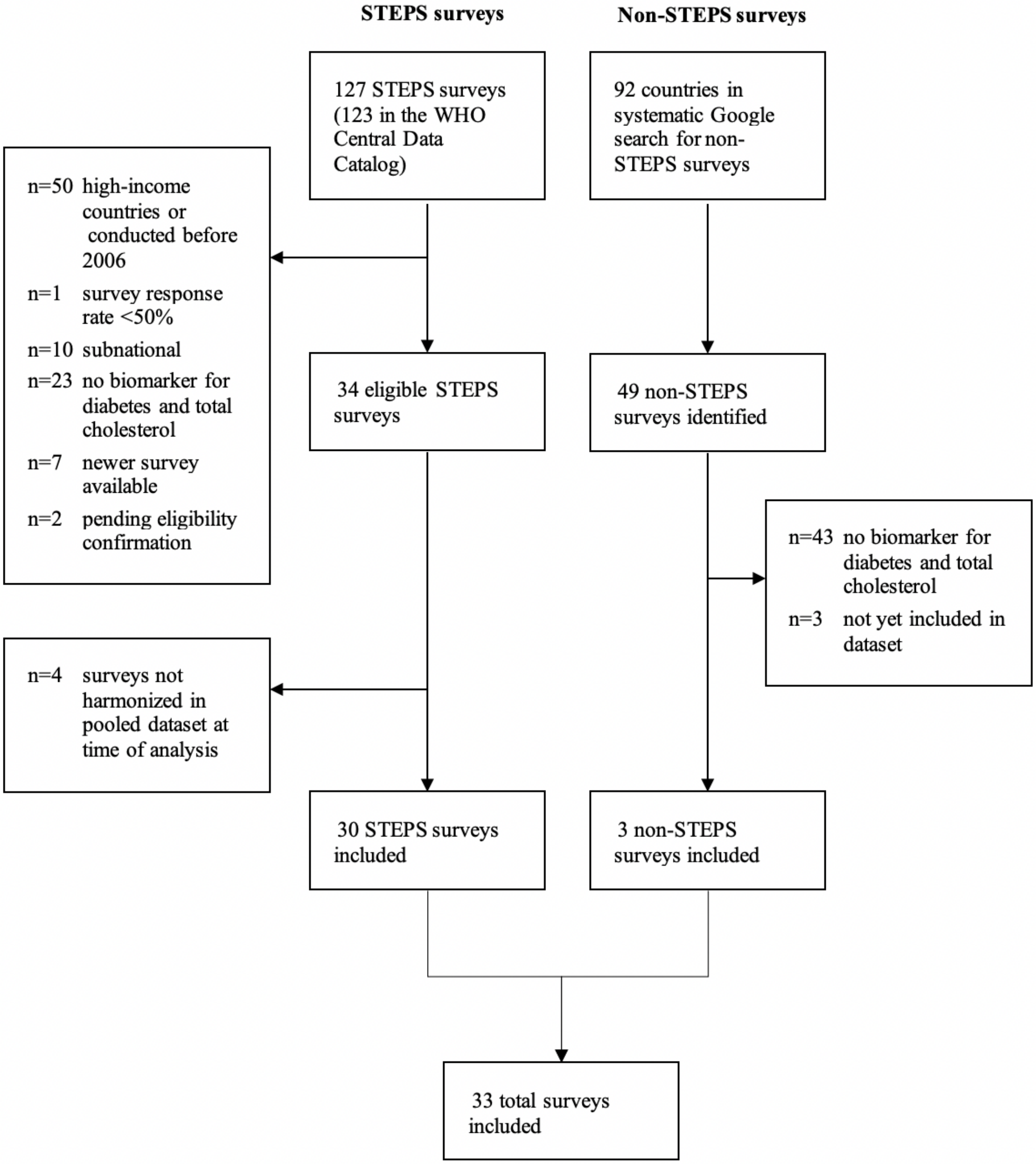

Appendix References

1 WHO CVD Risk Chart Working Group. World Health Organization cardiovascular disease risk charts: revised models to estimate risk in 21 global regions. *Lancet Glob Health* 2019; **7**: e1332–45.

2 Basu S, Sussman JB, Berkowitz SA, Hayward RA, Yudkin JS. Development and validation of Risk Equations for Complications Of type 2 Diabetes (RECODe) using individual participant data from randomised trials. *The lancet Diabetes & endocrinology* 2017; **5**. DOI:10.1016/S2213-8587(17)30221-8.

3 Basu S, Sussman JB, Berkowitz SA, *et al.* Validation of Risk Equations for Complications of Type 2 Diabetes (RECODe) Using Individual Participant Data From Diverse Longitudinal Cohorts in the U.S. *Diabetes Care* 2018; **41**: 586–95.

4 GBD Compare. http://vizhub.healthdata.org/gbd-compare (accessed March 3, 2021).

5 Li S, Vandvik PO, Lytvyn L, *et al.* SGLT-2 inhibitors or GLP-1 receptor agonists for adults with type 2 diabetes: a clinical practice guideline. *BMJ* 2021; **373**. DOI:10.1136/bmj.n1091.

6 Smith-Spangler CM, Juusola JL, Enns EA, Owens DK, Garber AM. Population strategies to decrease sodium intake and the burden of cardiovascular disease: a cost-effectiveness analysis. *Ann Intern Med* 2010; **152**: 481–7, W170–3.

7 Law MR, Wald NJ, Morris JK, Jordan RE. Value of low dose combination treatment with blood pressure lowering drugs: analysis of 354 randomised trials. *BMJ* 2003; **326**: 1427.

8 SOLVD Investigators, Yusuf S, Pitt B, Davis CE, Hood WB, Cohn JN. Effect of enalapril on survival in patients with reduced left ventricular ejection fractions and congestive heart failure. *N Engl J Med* 1991; **325**: 293–302.

9 Vijan S, Sussman JB, Yudkin JS, Hayward RA. Effect of Patients' Risks and Preferences on Health Gains With Plasma Glucose Level Lowering in Type 2 Diabetes Mellitus. JAMA Internal Medicine. 2014; **174**: 1227.

10 Cholesterol Treatment Trialists' (CTT) Collaboration, Fulcher J, O'Connell R, *et al.* Efficacy and safety of LDL-lowering therapy among men and women: meta-analysis of individual data from 174,000 participants in 27 randomised trials. *Lancet* 2015; **385**: 1397–405.

11 Inzucchi S. Diabetes Facts and Guidelines. Yale University School of Medicine, 2011 https://www.yumpu.com/en/document/read/5809026/diabetes-facts-and-guidelines-endocrinology-yale-university (accessed March 3, 2021).

12 Husereau D, Drummond M, Petrou S, *et al.* Consolidated Health Economic Evaluation

Reporting Standards (CHEERS)—Explanation and Elaboration: A Report of the ISPOR Health Economic Evaluation Publication Guidelines Good Reporting Practices Task Force. Value in Health. 2013; **16**: 231–50.

13 Common values in assessing health outcomes from disease and injury: disability weights measurement study for the Global Burden of Disease Study 2010. *Lancet* 2012; **380**: 2129–43.

14 Rushby JAF, Hanson K. Calculating and presenting disability adjusted life years (DALYs) in cost-effectiveness analysis. *Health Policy Plan* 2001; **16**: 326–31.

15 NCDs | Tools for implementing WHO PEN (Package of essential noncommunicable disease interventions). 2020; published online Sept 28. http://www.who.int/ncds/management/pen_tools/en/ (accessed Feb 1, 2021).

16 American Diabetes Association. 9. Pharmacologic Approaches to Glycemic Treatment: Standards of Medical Care in Diabetes—2021. *Diabetes Care* 2021; **44**: S111–24.

17 American Diabetes Association. 10. Cardiovascular Disease and Risk Management: Standards of Medical Care in Diabetes—2021. *Diabetes Care* 2021; **44**: S125–50.

18 Ponikowski P, Voors AA, Anker SD, *et al.* 2016 ESC Guidelines for the diagnosis and treatment of acute and chronic heart failure: The Task Force for the diagnosis and treatment of acute and chronic heart failure of the European Society of Cardiology (ESC)Developed with the special contribution of the Heart Failure Association (HFA) of the ESC. *Eur Heart J* 2016; **37**: 2129–200.

19 NCDs | Tools for implementing WHO PEN (Package of essential noncommunicable disease interventions). 2020; published online Sept 28. http://www.who.int/ncds/management/pen_tools/en/ (accessed Feb 1, 2021).

20 Mushi L, Marschall P, Fleßa S. The cost of dialysis in low and middle-income countries: a systematic review. *BMC Health Serv Res* 2015; **15**: 506.
